# Phenotypic and Genetic Correlations of Feed Efficiency Traits with Growth and Carcass Traits in Nellore Cattle Selected for Postweaning Weight

**DOI:** 10.1371/journal.pone.0161366

**Published:** 2016-08-18

**Authors:** Thais Matos Ceacero, Maria Eugênia Zerlotti Mercadante, Joslaine Noely dos Santos Gonçalves Cyrillo, Roberta Carrilho Canesin, Sarah Figueiredo Martins Bonilha, Lucia Galvão de Albuquerque

**Affiliations:** 1 Centro APTA Bovinos de Corte, Instituto de Zootecnia, Sertãozinho, SP, Brazil; 2 Faculdade de Ciências Agrárias e Veterinárias, Universidade Paulista “Júlio de Mesquita Filho”, Jaboticabal, SP, Brazil; University of Florida, UNITED STATES

## Abstract

This study evaluated phenotypic (r_ph_) and genetic correlations (r_g_) between 8 feed efficiency traits and other traits of economic interest including weight at selection (WS), loin-eye area (LEA), backfat thickness (BF), and rump fat thickness (RF) in Nellore cattle. Feed efficiency traits were gain:feed, residual feed intake (RFI), residual feed intake adjusted for backfat thickness (RFI_b_) and for backfat and rump fat thickness (RFI_sf_), residual body weight gain (RG), residual intake and body weight gain (RIG), and residual intake and body weight gain using RFI_b_ (RIG_b_) and RFI_sf_ (RIG_sf_). The variance components were estimated by the restricted maximum likelihood method using a two-trait animal model. The heritability estimates (h^2^) were 0.14, 0.24, 0.20, 0.22, 0.19, 0.15, 0.11 and 0.11 for gain:feed, RFI, RFI_b_, RFI_sf_, RG, RIG, RIG_b_ and RIG_sf_, respectively. All r_ph_ values between traits were close to zero, except for the correlation of feed efficiency traits with dry matter intake and average daily gain. High r_g_ values were observed for the correlation of dry matter intake, average daily gain and metabolic weight with WS and hip height (>0.61) and low to medium values (0.15 to 0.48) with the carcass traits (LEA, BF, RF). Among the feed efficiency traits, RG showed the highest r_g_ with WS and hip height (0.34 and 0.25) and the lowest r_g_ with subcutaneous fat thickness (-0.17 to 0.18). The r_g_ values of RFI, RFI_b_ and RFI_sf_ with WS (0.17, 0.23 and 0.22), BF (0.37, 0.33 and 0.33) and RF (0.30, 0.31 and 0.32) were unfavorable. The r_g_ values of gain:feed, RIG, RIG_b_ and RIG_sf_ with WS were low and favorable (0.07 to 0.22), while medium and unfavorable (-0.22 to -0.45) correlations were observed with fat thickness. The inclusion of subcutaneous fat thickness in the models used to calculate RFI did not reduce the r_g_ between these traits. Selecting animals for higher feed efficiency will result in little or no genetic change in growth and will decrease subcutaneous fat thickness in the carcass.

## Introduction

The Brazilian meat industry is one of the most dynamic in the world. The average annual genetic trends for growth traits reported by Nellore breeding programs in Brazil ranges from 0.15 to 0.61% of the average/year for weaning weight and from 0.15 to 0.77% of the average/year for postweaning weights, while higher genetic trends are obtained for postweaning weight gain (0.11 to 1.02% of the average/year). As a correlated response, this selection has resulted in a genetic trend of 0.33% of the average/year in birth weight and of 0.35% of the average/year in mature cow weight [1]. These figures highlight the intense improvement work of technicians, breeders and companies commercializing genetic material to increase meat production of Nellore cattle, the most representative breed in the Brazilian cattle herd. However, although selection for higher weight at a specific age, associated with muscling and fertility traits, can be used to increase production efficiency, this selection approach disregards the main economic factor of animal production, i.e., feeding and consequently feed efficiency.

In *Bos taurus*, feed efficiency traits exhibit moderate heritability [2], low to high genetic correlations with growth (depending on the feed efficiency trait analyzed), and moderate genetic antagonism with carcass fat thickness. However, in addition to the fact that some genetic parameter estimates reported in the literature have high standard errors, fewer than a dozen studies on Zebu cattle raised in tropical production systems are available [3, 4]. The objective of this study was to estimate genetic correlations of different feed efficiency traits with growth and carcass traits in Nellore cattle. The selection of beef cattle for feed efficiency will lead to a reduction in production costs, but should not have a negative impact on weight or carcass quality.

## Material and Methods

The experiment was conducted in accordance with animal welfare guidelines according to State Law No. 11.977 of the State of São Paulo, Brazil. All animal procedures were approved by the Ethics and Animal Handling Committee of the Instituto de Zootecnia, Nova Odessa, SP, Brazil.

Records from 8,078 Nellore animals born between 1978 and 2013 to 320 bulls and 2,078 dams from Centro APTA Bovinos de Corte, Sertãozinho, São Paulo, Brazil (21°10’ S; 47°57’ W) were used. This Nellore herd consists of three selection lines started in 1978, two of them selected for higher postweaning weight and one control line selected for average postweaning weight [5, 6]. In the three lines, males are selected for yearling weight adjusted to 378 days of age (W378) obtained after feedlot performance testing for 168 days, and females are selected for postweaning weight adjusted to 550 days of age (W550) obtained on pasture.

Dry matter intake (DMI) and average daily gain (ADG) were obtained for intact males (n = 600) and females (n = 355), born between 2004 and 2013 to 78 bulls and 542 dams, in 21 performance tests after weaning on pasture. Prior to the test, the animals were allowed to adapt to the diet and facilities for a minimum period of 21 days. The animals started the test at a body weight of 237 ± 50 kg and age of 287 ± 38 days and remained in individual (n = 683) or collective pens equipped with the GrowSafe^®^ System (GrowSafe Systems Ltd., Airdrie, Alberta, Canada) (n = 272) for 84 ± 15 days. The animals had *ad libitum* access to diet and water. The feed composition of the diet offered was modified over the years, but was equivalent in the content of total digestible nutrients (62%) and crude protein (13%) [7]. Diet samples were obtained every week for the determination of dry matter. Daily DMI values were excluded if there were no leftovers in the individual pens or when the GrowSafe^®^ System indicated daily assigned feed disappearance ≤ 90%.

The animals were weighed every 14 days after fasting for 12 h (2005 and 2006) and every 28 days for males born in 2007 and 2008 and for females born from 2009 to 2011 after fasting. From 2009 to 2012, males were weighed weekly without fasting, with three weekly weight recordings on consecutive days in 2009 and 2010, two weekly weight recordings on consecutive days in 2011, and one weight recording per week in 2012. In 2013 and 2014, males were weighed without fasting every 14 days. In 2012, females were weighed on two consecutive days every 15 days. Therefore, each animal was weighed at least 4 times with prior fasting and at least 6 times without prior fasting.

Dry matter intake was obtained as the mean of all valid days of feed intake during the test period. Average daily gain was estimated by linear regression of weights on days on test (DOT):

$${\text{y}}_{\text{i}}=\alpha +\beta *{\text{DOT}}_{\text{i}}\;+\varepsilon_{\text{i}}$$

where y_i_ is the animal’s weight in the i^th^ observation; α is the intercept that represents the initial weight; β is the linear regression coefficient that represents ADG; DOT_i_ is the days on test in the i^th^ observation, and ε_i_ is the random error associated with each observation. The mid-test metabolic body weight (BW^0.75^) was calculated as BW^0.75^ = [α + β * (DOT/2)]^0.75^.

Gain:feed was calculated as the ratio between ADG and DMI. Residual feed intake (RFI) was estimated as the residual of the linear regression equation of DMI on ADG and BW^0.75^, and residual body weight gain (RG) was estimated as the residual of the regression equation of ADG on DMI and BW^0.75^ [8] within each contemporary group. Residual intake and body weight gain (RIG) was calculated from RFI and RG [9].

Three linear regression models were fitted to estimate RFI:

$$\text{DMI}=\beta_{0}+\beta_{1}\text{ADG}+\beta_{2}{\text{BW}}^{0.75}+\varepsilon$$ (1)

$$\text{DMI}=\beta_{0}+\beta_{1}\text{ADG}+\beta_{2}{\text{BW}}^{0.75}+\beta_{3}\text{BF}+\varepsilon$$ (2)

$$\text{DMI}=\beta_{0}+\beta_{1}\text{ADG}+\beta_{2}{\text{BW}}^{0.75}+\beta_{3}\text{BF}+\beta_{4}\text{RF}+\varepsilon$$ (3)

where β_0_ is the intercept and β_1_, β_2_, β_3_ and β_4_ are the partial regression coefficients of DMI on ADG, BW^0.75^, backfat thickness (BF) and rump fat thickness (RF), respectively, and *ε* in models 1, 2 and 3 is RFI, RFI adjusted for BF (RFI_b_) and RFI adjusted for BF and RF (RFI_sf_), respectively.

The following linear regression model was fitted to estimate RG:

$$\text{ADG}=\beta_{0}+\beta_{1}\text{DMI}+\beta_{2}{\text{BW}}^{0.75}+\varepsilon$$

where β_0_ is the intercept and β_1_ and β_2_ are the partial regression coefficients of ADG on DMI and BW^0.75^, respectively, and ε is RG.

Residual intake and gain was calculated as:

$$\text{RIG}=\frac{\text{RG}}{\sigma_{\text{RG}}}-\frac{\text{RFI}}{\sigma_{\text{RFI}}}$$

where σ_RG_ is the standard deviation of the mean RG of the contemporary group and σ_RFI_ is the standard deviation of the mean RFI of the contemporary group. The residual intakes and gains using RFI_b_ (RIG_b_) and RFI_sf_ (RIG_sf_) were also calculated considering the standard deviations of the mean of the respective traits for the contemporary group.

Weight at selection (WS), defined as W378 (males) and W550 (females), was analyzed as a single trait. Since hip height (HH) was obtained in males and females at yearling and postweaning, respectively, it was also analyzed as a single trait. The same was done for chest circumference (CC). Weight at selection was obtained for animals born from 1978 to 2013, HH for animals born from 1985 to 2013, and CC for animals born from 1989 to 2013.

The carcass traits were measured by ultrasound [Pie Medical 401347-Aquila, 3.5-MHz, 18-cm linear probe; Pie Medical Equipment B.V., Maastricht, The Netherlands] in males (yearling) born from 1996 to 2002 (except for the years 1998, 2000 and 2003) and in females (postweaning) born from 2004 to 2013. For the measurement of loin-eye area (LEA) and BF, the transducer was placed perpendicular to the spine in a transverse position over the longissimus dorsi muscle between the 12^th^ and 13^th^ rib on the left side of the animal using a standoff pad. Rump fat thickness was measured by placing the transducer at the junction of the gluteus medius and biceps femoris muscles located between the hook and pin bone [10]. The images were saved and subsequently analyzed using the Echo Image Viewer 1.0 (Pie Medical Equipment B.V., Maastricht, The Netherlands).

Animals with more than one record outside the interval of ±3.5 standard deviations from the mean of the contemporary group were eliminated from the database. For the growth and carcass traits, the contemporary groups were defined as year of birth of the animal, selection line, and sex. For the feed efficiency traits, the contemporary group was defined as year of birth (2004 to 2013), test facility [3 facilities with at least 32 individual pens or one collective pen], and sex (intact male or female). Table 1 shows the data structure and descriptive statistics of the traits.

**Table 1:** Description of the final data set of feed efficiency, growth and carcass traits.

Trait	Mean ± SD	No. of animals with records	No. of contemporary groups
DMI (kg/day)	6.83 ± 1.33	955	21
ADG (kg)	1.00 ± 0.26	955	21
BW^0.75^ (kg)	68.2 ± 10.1	955	21
Gain:feed (kg gain/kg DM)	0.148 ± 0.033	955	21
RFI (kg/day)	-0.011 ± 0.60	955	21
RFI_b_ (kg/day)	-0.0091 ± 0.60	894	21
RFI_sf_ (kg/day)	-0.0090 ± 0.59	891	21
RG (kg/day)	0.002 ± 0.12	955	21
RIG	0.044 ± 1.71	955	21
RIG_b_	0.042 ± 1.70	894	21
RIG_sf_	0.043 ± 1.69	891	21
WS (kg)	300 ± 48.8	8,078	201
HH (cm)	132 ± 5.43	6,548	169
CC (cm)	164 ± 8.72	3,876	100
LEA (cm^2^)	51.4 ± 8.89	2,283	65
BF (mm)	1.75 ± 1.42	2,285	65
RF (mm)	5.08 ± 2.54	1,817	51

DMI, dry matter intake; ADG, average daily gain; BW^0.75^, metabolic body weight; RFI, residual feed intake; RFI_b_, residual feed intake adjusted for backfat thickness; RFI_sf_, residual feed intake adjusted for backfat and rump fat thickness; RG, residual body weight gain; RIG, residual intake and gain; RIG_b_, residual intake and gain using RFI_b_; RIG_sf_, residual intake and gain using RFI_sf_; WS, weight at selection; HH, hip height of males and females at selection; CC, chest circumference of males and females at selection; LEA, loin-eye area; BF, backfat thickness; RF, rump fat thickness.

The (co)variance components were estimated by the restricted maximum likelihood method under a two-trait animal model using the WOMBAT program [11]. For the feed efficiency traits, DMI, ADG and BW^0.75^, the model included random direct additive genetic effects, the fixed effects of contemporary group, and age of animal (linear effect) and age of dam (linear and quadratic effects) as covariates. For the other traits (growth and carcass), the model included random direct additive genetic and maternal permanent environmental effects (only for WS), in addition to the fixed effects of contemporary group and month of birth of the animal, age of animal (linear effect), and age of dam (linear and quadratic effects). A pedigree file containing 8,478 animals was used in all analyses.

The general model can be written in matrix form as:

y=Xβ+Zα+Wc+ε

where y is the vector of observations; β is the vector of fixed effects associated with the incidence matrix X; α is the vector of random direct additive genetic effects associated with the incidence matrix Z; c is the vector of maternal permanent environmental effects associated with the incidence matrix W, and ε is the vector of residuals.

In this study, it was assumed that:

$$\text{E}\left[\text{y}\right]=\text{X}\beta ;\text{ Var}\left(\alpha \right)\;=\;\text{A}⊕{\text{S}}_{\alpha };\;\text{Var}\left(\text{c}\right)=\text{I}⊕{\text{S}}_{\text{c}}\;\text{and}\;\text{Var}\left(\text{e}\right)=\text{I}⊕{\text{S}}_{\text{e}}$$

where S_α_ is the matrix of direct additive genetic (co)variances; S_c_ is the matrix of maternal permanent environmental variance; S_e_ is the matrix of residual (co)variances; A is the numerator of the additive genetic relationship matrix; I is an identity matrix, and ⊕ is the direct product between matrices. It was also assumed that α, c and e are not correlated.

## Results

The general equations of predicted DMI and ADG were: DMIp = 2.112 (±0.134) x ADG + 0.084 (±0.003) x BW^0.75^ (R^2^ = 0.80) and ADGp = 0.099 (±0.006) x DMI + 0.002 (±0.001) x BW^0.75^ (R^2^ = 0.75), respectively. The mean RFI was -0.012 ± 0.712 kg/day (-2.314 to 4.964 kg/day) in males and -0.010 ± 0.375 kg/day (-1.176 to 1.287 kg/day) in females. The mean RG was 0.002 ± 0.136 kg/day (-0.707 to 0.332 kg/day) in males and 0.003 ± 0.097 kg/day (-0.283 to 0.329 kg/day) in females.

**Fig 1:**
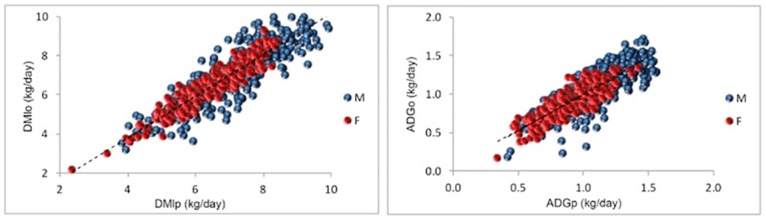
Relationship between observed (DMIo and ADGo) and predicted (DMIp and ADGp) dry matter intake (left) and average daily gain (right).

The coefficient of variation of DMI, ADG and BW^0.75^ was higher (19%, 26% and 15%, respectively) than the coefficient of variation of RFI and RG (8.7% and 12%), which were calculated using mean DMI and ADG, respectively. Coefficients of variation of 4% to 8% for RFI and of 10% to 14% for RG were reported in studies on *Bos taurus* [2]. The coefficients of variation were 7% and 10% for RFI and 20% for RG in Nellore cattle (*Bos indicus*) [3, 4]. These data indicate considerable variation in RFI and RG. Fig 1 illustrates the dispersion of observed and predicted DMI and ADG. The standard deviations of RFI, RFI_b_ and RFI_sf_ were similar.

Dry matter intake corresponded to 2.41% and 2.54% of the average body weight of males and females, respectively, during the test. A crude estimate of the percentage of feed consumed for maintenance, implicit in the RFI equation, can be obtained by (100 x β_1_ x BW^0.75^)/(β_1_ x BW^0.75^ x β_2_ x ADG), where β_1_ and β_2_ are the partial regression coefficients of DMI on ADG and BW^0.75^, respectively, and BW^0.75^ and ADG are the means shown in Table 1 [12]. Considering all animals, 47% of DMI was required for maintenance. This percentage was 44.76% for males and 49.48% for females, suggesting that females were less efficient than males. In fact, a study [13] analyzing part of the same database as used here, reported a better gain:feed ratio for males compared to females. Finishing *Bos taurus* steers attending the Australian, Korean and Japanese markets required 67%, 72% and 61% of DMI for maintenance [12]. These results support the view that *Bos indicus* animals have lower maintenance requirements than *Bos taurus* [14].

The variance components and heritability estimates of the feed efficiency traits were obtained in a series of two-trait analyses between these traits. The mean values are shown in Table 2. The heritability estimates for component traits of RFI and for the growth (WS, HH, and CC) and ultrasound-measured carcass traits (LEA, BF, and RF) were also obtained by two-trait analyses and the mean values are shown in Table 3. This table also displays the correlations between these traits. Table 4 shows the phenotypic and genetic correlations between the feed efficiency traits and the growth and carcass traits.

**Table 2:** Variance components and heritability estimates for the feed efficiency traits.

Trait^1^	σ^2^_a_	σ^2^_e_	h^2^ ± SE
Gain:feed	0.06	0.38	0.14 ± 0.06
RFI	0.08	0.28	0.24 ± 0.06
RFI_b_	0.07	0.29	0.20 ± 0.06
RFI_sf_	0.07	0.28	0.22 ± 0.06
RG	0.003	0.01	0.19 ± 0.06
RIG	0.45	2.44	0.15 ± 0.05
RIG_b_	0.34	2.53	0.11 ± 0.05
RIG_sf_	0.31	2.51	0.11 ± 0.05

RFI, residual feed intake; RFI_b_, residual feed intake adjusted for backfat thickness; RFI_sf_, residual feed intake adjusted for backfat and rump fat thickness; RG, residual body weight gain; RIG, residual intake and gain; RIG_b_, residual intake and gain using RFI_b_; RIG_sf_, residual intake and gain using RFI_sf_; σ^2^_a_, additive genetic variance component; σ^2^_e_, residual variance component; h^2^, heritability; SE, standard error.

**Table 3:** Heritability (diagonal), phenotypic correlation (below the diagonal) and genetic correlation (above the diagonal) between dry matter intake, average daily gain, metabolic body weight, growth and carcass traits.

Trait	DMI	ADG	BW^0.75^	WS	HH	CC	LEA	BF	RF
DMI	0.45 ± 0.05	0.83 ± 0.06	0.82 ± 0.05	0.8 ± 0.06	0.61 ± 0.07	0.79 ± 0.07	0.48 ± 0.10	0.29 ± 0.13	0.39 ± 0.11
ADG	0.56	0.41 ± 0.06	0.73 ± 0.08	0.84 ± 0.05	0.62 ± 0.08	0.80 ± 0.08	0.41 ± 0.11	0.15 ± 0.13	0.24 ± 0.12
BW^0.75^	0.65	0.42	0.53 ± 0.06	0.84 ± 0.05	0.80 ± 0.04	0.90 ± 0.03	0.63 ± 0.07	0.14 ± 0.12	0.30 ± 0.11
WS	0.60	0.61	0.83	0.40 ± 0.03	0.75 ± 0.02	0.87 ± 0.02	0.48 ± 0.06	-0.02 ± 0.10	0.11 ± 0.10
HH	0.45	0.36	0.69	0.67	0.61 ± 0.02	0.64 ± 0.04	0.19 ± 0.07	-0.15 ± 0.09	-0.03 ± 0.09
CC	0.55	0.60	0.75	0.73	0.57	0.31 ± 0.03	0.38 ± 0.08	0.17 ± 0.11	0.29 ± 0.10
LEA	0.44	0.36	0.59	0.49	0.31	0.45	0.44 ± 0.05	0.09 ± 0.10	0.18 ± 0.10
BF	0.23	0.15	0.29	0.22	0.65	0.28	0.20	0.30 ± 0.04	0.79 ± 0.05
RF	0.25	0.21	0.29	0.26	0.07	0.30	0.25	0.61	0.38 ± 0.05

DMI, dry matter intake; ADG, average daily gain; BW^0.75^, metabolic body weight; WS, weight at selection; HH, hip height of males and females at selection; CC, chest circumference of males and females at selection; LEA, loin-eye area; BF, backfat thickness; RF, rump fat thickness.

**Table 4:** Phenotypic and genetic correlations of dry matter intake, average daily gain, weight at selection, hip height, chest circumference, loin-eye area, backfat thickness and rump fat thickness with the feed efficiency traits.

Trait	DMI	ADG	WS	HH	CC	LEA	BF	RF
Phenotypic correlation								
Gain:feed	-0.28	0.58	0.10	-0.03	0.07	0.04	-0.02	0.01
RFI	0.73	0.04	0.06	0.04	0.06	0.03	0.05	0.06
RFI_b_	0.71	0.02	0.05	0.03	0.04	0.03	0.03	0.04
RFI_sf_	0.70	0.02	0.05	0.04	0.04	0.01	0.02	0.03
RG	-0.10	0.68	0.17	0.00	0.09	0.03	-0.03	0.01
RIG	-0.44	0.34	0.04	-0.03	0.00	0.01	-0.08	-0.05
RIG_b_	-0.41	0.35	0.05	-0.03	0.01	0.00	-0.04	-0.04
RIG_sf_	-0.41	0.35	0.05	-0.04	0.01	0.00	-0.05	-0.02
Genetic correlation								
Gain:feed	-0.11 ± 0.18	0.39 ± 0.15	0.09 ± 0.15	0.08 ± 0.15	0.15 ± 0.20	0.01 ± 0.19	-0.22 ± 0.20	-0.23 ± 0.20
RFI	0.68 ± 0.08	0.33 ± 0.16	0.17 ± 0.14	0.06 ± 0.13	0.14 ± 0.17	0.00 ± 0.16	0.37 ± 0.17	0.30 ± 0.16
RFI_b_	0.73 ± 0.09	0.40 ± 0.17	0.23 ± 0.16	0.06 ± 0.14	0.14 ± 0.18	-0.06 ± 0.17	0.33 ± 0.19	0.31 ± 0.17
RFI_sf_	0.71 ± 0.09	0.38 ± 0.17	0.22 ± 0.15	0.08 ± 0.14	0.14 ± 0.18	-0.04 ± 0.19	0.33 ± 0.18	0.32 ± 0.17
RG	0.21 ± 0.17	0.66 ± 0.09	0.34 ± 0.14	0.25 ± 0.14	0.34 ± 0.18	0.09 ± 0.17	-0.17 ± 0.17	-0.18 ± 0.17
RIG	-0.27 ± 0.16	0.19 ± 0.17	0.10 ± 0.15	0.13 ± 0.14	0.17 ± 0.19	0.16 ± 0.19	-0.38 ± 0.20	-0.31 ± 0.20
RIG_b_	-0.27 ± 0.19	0.14 ± 0.20	0.08 ±0.17	0.13 ± 0.14	0.22 ± 0.22	0.28 ± 0.24	-0.45 ± 0.26	-0.42 ± 0.25
RIG_sf_	-0.28 ± 0.18	0.12 ± 0.20	0.07 ± 0.17	0.15 ± 0.16	0.20 ± 0.22	0.29 ± 0.23	-0.45 ± 0.26	-0.43 ± 0.25

DMI, dry matter intake; ADG, average daily gain; RFI, residual feed intake; RFI_b_, residual feed intake adjusted for backfat thickness; RFI_sf_, residual feed intake adjusted for backfat and rump thickness; RG, residual body weight gain; RIG, residual intake and gain; RIG_b_, residual intake and gain using RFI_b_; RIG_sf_, residual intake and gain using RFI_sf_; WS, weight at selection; HH, hip height of males and females at selection; CC, chest circumference of males and females at selection; LEA, loin-eye area; BF, backfat thickness; RF, rump fat thickness.

## Discussion

The heritability estimates for the feed efficiency traits ranged from 0.11 to 0.24 (Table 2). The lowest estimate was obtained for the feed efficiency trait whose concept comprised a larger number of traits (RIG_b_ and RIG_sf_), while the highest estimate was found for RFI. Pooled heritabilities higher than those estimated in the present study (0.23 ± 0.013, 0.33 ± 0.013 and 0.28 ± 0.030 for feed:gain, RFI and RG, respectively) were reported in a comprehensive meta-analysis [2]. However, this meta-analysis mainly included studies on *Bos taurus* or composites. Also, a meta-analysis comprising 39 heritability estimates [15] found a pooled heritability of 0.255 ± 0.008 for RFI, a value very similar to that estimated in the present study. Studies investigating Nellore cattle raised in Brazil reported higher heritabilities for feed:gain, RFI, RG and RIG (0.313 ± 0.110, 0.375 ± 0.162, 0.406 ± 0.147 and 0.544 ± 0,173, respectively) [4] and higher “genomic heritabilities” (the proportion of phenotypic variance explained by genome markers) for feed:gain, gain:feed and RFI (0.39, 0.47 and 0.33, respectively) [3].

Although RG has been proposed concomitantly with RFI [8], genetic parameter estimates for this trait have only been reported in recent years. In contrast to RFI in which negative values indicate more efficient animals, positive values of RG indicate more efficient animals. Residual gain is essentially a growth trait since ADG is adjusted for DMI and BW^0.75^. However, in the present study the heritability for RG (0.19 ± 0.06) was substantially lower than that estimated for ADG which was 0.41 (Table 3), probably because a large part of the genetic variation in ADG is due to genetic differences in DMI between animals. The RIG trait was proposed later [9]. The authors justified the proposal of this trait by the fact that the phenotypic independence between RFI and ADG is a reason why breeders do not adopt the former in selection programs, since animals with slow growth (undesirable trait) can be classified as efficient (negative RFI). The phenotypic independence between RG and DMI creates a similar situation. One solution to this problem was the combination of RFI and RG (i.e., RIG), which guarantees the independence of this trait from BW^0.75^, as well as a negative phenotypic correlation with DMI and a positive phenotypic correlation with ADG. Consequently, RIG reduces (but does not eliminate) the probability of a slow-growing animal to be highly ranked based on RIG [2].

The heritabilities for the component traits of feed efficiency (DMI, ADG, and BW^0.75^), growth traits and ultrasound-measured carcass traits (Table 3) were higher than the estimates obtained for the feed efficiency traits. These results agree with those reported in meta-analyses of heritabilities for feed efficiency traits in cattle [2, 15]. In general, the component traits of feed efficiency have a higher heritability than feed efficiency traits. This finding might be explained by the fact that the residuals (in the case of RFI, RG, and RIG) include not only the variation that is not captured by the independent variables of the prediction equation, but also random measurement errors of DMI, ADG (and BF and RF in the case of RFI_b_, RFI_sf_, RIG_b_ and RIG_sf_), rather than simply the separate measurement error of each trait as is the case for DMI, ADG, BF and RF [2].

The use of different models for predicting DMI can provide different heritabilities for RFI [16]. The inclusion of subcutaneous fat thickness in the prediction model of DMI slightly reduced the h^2^ estimate of RFI_b_, RFI_sf_, RIG_b_ and RIG_sf_. In other words, genetic differences in feed efficiency can also be due to differences in the composition of weight gain. However, this may depend on the age at which the animals are evaluated since more energy is used for fat deposition than for protein deposition, while fat maintenance requires less energy than protein maintenance [17]. Lower heritabilities of RFI adjusted for fat thickness, compared to the heritability of RFI estimated with the traditional model, were also reported for Brangus heifers [18] and Charolais steers [19]. However, no difference between the heritabilities for RFI and RFI_sf_ was observed in different *Bos taurus* breeds [20] or in Angus steers [19].

Table 3 shows the genetic and phenotypic correlations between the component traits of feed efficiency and the growth and carcass traits. Selection for growth (WS), a selection criterion that has been used in the herd of the present study since 1980 [5], leads to an increase in ADG, BW^0.75^, HH, CC, and LEA. This selection exerts little effect on fat deposition in the carcass, but invariably increases feed intake (DMI). The results are consistent with the findings of other studies [8, 12, 21]. High phenotypic and genetic correlations between growth traits and DMI have been widely described and discussed in the literature for different domestic animal species other than beef cattle [22].

The phenotypic relationships created by construction of the traits RFI, RG and RIG, i.e., zero correlations between RFI and ADG and between RG and DMI, a negative correlation between RIG and DMI and a positive correlation between RIG and ADG, do not necessarily imply similar genetic relationships (Table 4). The genetic correlation estimates had high standard errors due to the small number of animals with DMI records, a trait that is difficult to measure. Despite the high standard errors of the genetic correlation estimates, the selection of low RFI animals will result in a decrease in DMI but also in ADG, which is unfavorable, while the selection of high RFI animals will increase both ADG and DMI, which is also unfavorable. On the other hand, although the selection of high RIG animals leads to a decrease in DMI and to an increase in ADG, selection exclusively based on RIG would not be justified (Table 4) since the genetic correlations between these traits are so low and RIG is the least heritable feed efficiency trait (Table 2). High and favorable genetic correlations between RIG and DMI (-0.35 ± 0.10) and between RIG and ADG (0.47 ± 0.10), which are higher than those estimated in the present study (Table 4), have been reported [9].

One may recommend the inclusion of carcass fat thickness in the prediction model of DMI for calculating RFI so that RFI is phenotypically independent of BF and RF [18–20, 23]. However, this procedure may not guarantee a zero genetic correlation between these traits, although in the present study the genetic correlation of RFI_b_ and RFI_sf_ with carcass fat thickness was slightly lower (Table 4). A highly consistent result obtained in the present study was that the genetic correlations between the eight feed efficiency traits and carcass fat thickness were invariably antagonistic, i.e., selecting animals that are genetically more efficient in terms of feed utilization will lead to a decrease in the breeding value for fat thickness, as observed in other studies [4, 12, 19]. A meta-analysis [2] reported a low positive pooled genetic correlation (0.20) between RFI and fat thickness in the carcass. However, an antagonistic correlation between RFI and carcass fat was observed in Charolais cattle that were finished late, but not in Angus cattle that were finished early [19]. Logically, lower feed intake for the same ADG and the same metabolic body weight (low RFI) must have costs for the animals. Lower fat deposition, which requires more energy than muscle deposition, might be one of them. However, the genetic correlations were of low to medium magnitude and associated with high standard errors; thus, this genetic relationship, which may differ between breeds, diets and phases of life of the animal, is not well established. The decrease in the breeding value of animals for carcass fat deposition may represent a problem for *Bos indicus* beef breeds. In fact, a relationship between the myofibrillar fragmentation index and tenderness, measured as shear force, was described in Nellore carcasses with greater BF compared to those with lower BF, suggesting possible tenderness problems in animals with a leaner carcass [24]. However, it is well established that the retail beef yield is lower for animals with a greater fat cover in the carcass [25], suggesting that it is desirable to select animals with moderate but not high breeding values for BF and RF.

Although selection for higher growth (WS) results in an increase in DMI and ADG (Table 3), it seems to have little effect on feed efficiency traits since the correlations with these traits were low (0.07 to 0.23), even with gain:feed (Table 4). An exception was RG whose genetic correlation with WS was slightly higher (0.34). Similar feed conversion was reported in Angus cattle (*Bos taurus*) lines selected for high and low growth rates compared to the unselected control line, with no evidence of the loss of feed efficiency after 15 years of selection for growth [26]. In Nellore cattle (*Bos indicus*), these results were confirmed by the similar RFI of animals from two lines selected for growth (high postweaning weight versus control) [27].

In conclusion, feed efficiency, growth and carcass traits of Nellore cattle obtained postweaning show sufficient genetic variability to respond to selection; however, the genetic progress will be slower for the feed efficiency traits than for the other traits. In this breed, RFI is the most heritable feed efficiency trait. Additionally, there is evidence of low genetic antagonism between RFI and growth and of high genetic synergism between RFI and DMI. This fact makes the trait eligible for selection for higher feed efficiency. Selecting animals that convert feed more efficiently will imply a certain opposite correlated response in fat thickness, i.e., an antagonism of medium magnitude exists between the effect of genes for feed efficiency and the genes for carcass fat deposition in Nellore cattle.

## Supporting Information

S1 File Pedigree information, model of analysis, parameter estimates, and the resulting covariance matrices for Tables 2 and 4. (DOCX)

S2 File Pedigree information, model of analysis, parameter estimates, and the resulting covariance matrices for Table 3. (DOCX)
