# Supplementary material for: Phenotypic and Genetic Correlations of Feed Efficiency Traits with Growth and Carcass Traits in Nellore Cattle Selected for Postweaning Weight
Source: PLoS One. 2016 Aug 18;11(8):e0161366. doi: 10.1371/journal.pone.0161366 (PMC4990259; doi:10.1371/journal.pone.0161366)
Supplement: S1 File — (DOCX) [file pone.0161366.s001.docx]

**S1 File. Supporting Information. Pedigree information, model of analysis, parameter estimates, and the resulting covariance matrices for Table 2 and Table 4.**

======= Version 30-08-2013 ======================================= **KM** ====

Program WOMBAT: Summary of Pedigree Information

==============================================================================

Two traits analysis (RFI x DMI)

Analysis type : "muv 2"

Data file : "DADOS.dat"

Pedigree file : "ReducedPedFile.dat"

Parameter file : "wombat.par"

No. of animal IDs in data file = = 955

No. of animal IDs in total = = 2288

*****Pedigree Structure for random effect : 1 ****************************

Original no. of animals = 2288

No. of animals after pruning = 2204

... proportion (%) remaining = 96.3

No. of levels w/out records = 1249

No. of levels with records = 955 100.0%

... 2 record(s) = 955 100.0%

No. of animals w/out offspring = 828 37.6%

No. of animals with offspring = 1376 62.4%

... and records = 127 5.8%

No. of animals with unknown sire = 59

No. of animals with unknown dam = 145

No. of animals with both parents unknown = 58

No. of animals with records =

... and unknown sire = 0

... and unknown dam = 0

... and both parents unknown = 0

No. of sires = 296

... with progeny in the data = 78

... with records & progeny in data = 18

No. of dams = 1080

... with progeny in the data = 542

... with records & progeny in data = 98

No. of animals with known/unpruned grand-parents

... with paternal grandsire = 2083

... with paternal granddam = 1957

... with maternal grandsire = 1959

... with maternal granddam = 1872

Inbreeding coefficients for random effect 1 computed

No. of inbred animals = 1768

Average inbreeding coefficient = 2.1450 (in %)

... amongst inbred animals = 2.6740 (in %)

random effect no. = 1 NRM

no. of elements in NRM/GIN inverse 8375

log determinant = -1487.8684515369519

======== end of file ============================01-04-2014==========17:36====

======= Version 30-08-2013 ======================================= **KM** ====

Program WOMBAT: Summary of information from Set-up step

==============================================================================

Analysis type : "muv 2"

Data file : "DADOS.dat"

Pedigree file : "ReducedPedFile.dat"

Parameter file : "wombat.par"

No. of traits = 2

nrec mean sdev min. max.

1 "RFI" 955 -0.112496E-01 0.608835 -2.31380 4.96420

2 "DMI" 955 6.83104 1.33500 2.15500 12.6400

Numbers of individuals/records for pairs of traits

1 2

1 "RFI" 955 955

2 "DMI" 955 955

Covariables

1"RFI" nrec mean sdev min. max.

1 "idv(2)" 955 6.15079 3.05304 3.00000 17.0000

2 "idade(1)" 955 369.980 37.3543 267.000 511.000

2"DMI" nrec mean sdev min. max.

1 "idv(2)" 955 6.15079 3.05304 3.00000 17.0000

2 "idade(1)" 955 369.980 37.3543 267.000 511.000

Fixed effects

1 "RFI" nlev

1 "gc" 21

2 "DMI" nlev

1 "gc" 21

Random effects nlev

1 "animal" 2204 NRM

======== end of file ============================01-04-2014==========17:36====

======= Version 30-08-2013 ======================================= **KM** ====

Program WOMBAT: Estimates of covariance components

==============================================================================

Analysis type : "muv 2"

Data file : "DADOS.dat"

Pedigree file : "ReducedPedFile.dat"

Parameter file : "wombat.par"

No. of traits = 2 RFI DMI

No. of records = 1910 955 955

No. of parameters = 6

Maximum log L = -9.574

-1/2 AIC & AICC = -15.574 -15.596

-1/2 BIC = -32.162 "Penalty factor" = 3.765

Parameter estimates with approx. sampling erors

1 CHOL Z 1 1 -0.445895 0.576833E-01

2 CHOL Z 1 2 0.431473 0.281810E-01

3 CHOL Z 2 2 -1.14319 0.607699E-01

4 CHOL A 1 1 -0.492020 0.869837E-01

5 CHOL A 1 2 0.199037 0.436589E-01

6 CHOL A 2 2 -1.55550 0.159290

Convergence criteria for last 3 iterates

Change in log likelihood = 9.824473 0.075191 0.000252

Change in parameter vector = 0.053103 0.009440 0.001264

Norm of gradient vector = 250.7318 18.2443 0.2654

Newton decrement = -18.6993 -0.1529 -0.0007

***** Estimates of residual covariances ************************************

Order of fit = 2

Covariance matrix

1 0.28780

2 0.27625 0.40992

Eigenvalues of covariance matrix

Value 0.63 0.07

(%) 90.55 9.45

Trace 0.70

Matrix of correlations and variance ratios

1 0.7737

2 0.8043 0.5230

Covariances & correlations with approximate sampling errors

1 COVS Z 1 1 0.287803 0.235058E-01 vrat 0.774 0.064

2 COVS Z 1 2 0.276251 0.287924E-01 corr 0.804 0.029

3 COVS Z 2 2 0.409921 0.472912E-01 vrat 0.523 0.068

***** Estimates for RE 1 "animal" ***************************************

No. of levels = 2204

Covariance structure = NRM

Order of fit = 2

Covariance matrix

1 0.84172E-01

2 0.12169 0.37380

Eigenvalues of covariance matrix

Value 0.42 0.04

(%) 91.30 8.70

Trace 0.46

Matrix of correlations and variance ratios

1 0.2263

2 0.6860 0.4770

Covariances & correlations with approximate sampling errors

4 COVS A 1 1 0.841721E-01 0.254369E-01 vrat 0.226 0.064

5 COVS A 1 2 0.121689 0.344455E-01 corr 0.686 0.084

6 COVS A 2 2 0.373798 0.650287E-01 vrat 0.477 0.068

***** Estimates of phenotypic covariances ***********************************

Covariance matrix

1 0.37197

2 0.39794 0.78372

Eigenvalues of covariance matrix

Value 1.03 0.13

(%) 88.77 11.23

Trace 1.16

Correlation matrix

1 1.0000

2 0.7370 1.0000

Covariances & correlations with approximate sampling errors

7 COVS T 1 1 0.371975 0.181772E-01

8 COVS T 1 2 0.397940 0.236141E-01 corr 0.737 0.016

9 COVS T 2 2 0.783719 0.411501E-01

======== end of file ============================01-04-2014==========17:36====

======= Version 30-08-2013 ======================================= **KM** ====

Program WOMBAT: Summary of Pedigree Information

==============================================================================

Two traits analysis (RFI x ADG)

Analysis type : "muv 2"

Data file : "DADOS.dat"

Pedigree file : "ReducedPedFile.dat"

Parameter file : "wombat.par"

No. of animal IDs in data file = = 955

No. of animal IDs in total = = 2288

*****Pedigree Structure for random effect : 1 ****************************

Original no. of animals = 2288

No. of animals after pruning = 2204

... proportion (%) remaining = 96.3

No. of levels w/out records = 1249

No. of levels with records = 955 100.0%

... 2 record(s) = 955 100.0%

No. of animals w/out offspring = 828 37.6%

No. of animals with offspring = 1376 62.4%

... and records = 127 5.8%

No. of animals with unknown sire = 59

No. of animals with unknown dam = 145

No. of animals with both parents unknown = 58

No. of animals with records =

... and unknown sire = 0

... and unknown dam = 0

... and both parents unknown = 0

No. of sires = 296

... with progeny in the data = 78

... with records & progeny in data = 18

No. of dams = 1080

... with progeny in the data = 542

... with records & progeny in data = 98

No. of animals with known/unpruned grand-parents

... with paternal grandsire = 2083

... with paternal granddam = 1957

... with maternal grandsire = 1959

... with maternal granddam = 1872

Inbreeding coefficients for random effect 1 computed

No. of inbred animals = 1768

Average inbreeding coefficient = 2.1450 (in %)

... amongst inbred animals = 2.6740 (in %)

random effect no. = 1 NRM

no. of elements in NRM/GIN inverse 8375

log determinant = -1487.8684515369519

======== end of file ============================01-04-2014==========17:41====

======= Version 30-08-2013 ======================================= **KM** ====

Program WOMBAT: Summary of information from Set-up step

==============================================================================

Analysis type : "muv 2"

Data file : "DADOS.dat"

Pedigree file : "ReducedPedFile.dat"

Parameter file : "wombat.par"

No. of traits = 2

nrec mean sdev min. max.

1 "RFI" 955 -0.112496E-01 0.608835 -2.31380 4.96420

2 "ADG" 955 1.00846 0.260202 0.176000 1.71800

Numbers of individuals/records for pairs of traits

1 2

1 "RFI" 955 955

2 "ADG" 955 955

Covariables

1"RFI" nrec mean sdev min. max.

1 "idv(2)" 955 6.15079 3.05304 3.00000 17.0000

2 "idade(1)" 955 369.980 37.3543 267.000 511.000

2"ADG" nrec mean sdev min. max.

1 "idv(2)" 955 6.15079 3.05304 3.00000 17.0000

2 "idade(1)" 955 369.980 37.3543 267.000 511.000

Fixed effects

1 "RFI" nlev

1 "gc" 21

2 "ADG" nlev

1 "gc" 21

Random effects nlev

1 "animal" 2204 NRM

======== end of file ============================01-04-2014==========17:41====

======= Version 30-08-2013 ======================================= **KM** ====

Program WOMBAT: Estimates of covariance components

==============================================================================

Analysis type : "muv 2"

Data file : "DADOS.dat"

Pedigree file : "ReducedPedFile.dat"

Parameter file : "wombat.par"

No. of traits = 2 RFI ADG

No. of records = 1910 955 955

No. of parameters = 6

Maximum log L = 1208.312

-1/2 AIC & AICC = 1202.312 1202.290

-1/2 BIC = 1185.724 "Penalty factor" = 3.765

Parameter estimates with approx. sampling erors

1 CHOL Z 1 1 -0.622497 0.406064E-01

2 CHOL Z 1 2 -0.111548E-01 0.769172E-02

3 CHOL Z 2 2 -2.13340 0.537226E-01

4 CHOL A 1 1 -1.24087 0.150888

5 CHOL A 1 2 0.347262E-01 0.170778E-01

6 CHOL A 2 2 -2.33992 0.113083

Convergence criteria for last 3 iterates

Change in log likelihood = 7.406684 0.065530 0.000081

Change in parameter vector = 0.017573 0.003100 0.000495

Norm of gradient vector = 657.0074 44.7499 0.7477

Newton decrement = -14.1723 -0.1313 -0.0002

***** Estimates of residual covariances ************************************

Order of fit = 2

Covariance matrix

1 0.28794

2 -0.59857E-02 0.14151E-01

Eigenvalues of covariance matrix

Value 0.29 0.01

(%) 95.36 4.64

Trace 0.30

Matrix of correlations and variance ratios

1 0.7750

2 -0.0938 0.5744

Covariances & correlations with approximate sampling errors

1 COVS Z 1 1 0.287942 0.233846E-01 vrat 0.775 0.064

2 COVS Z 1 2 -0.598570E-02 0.411636E-02 corr -0.094 0.065

3 COVS Z 2 2 0.141511E-01 0.148822E-02 vrat 0.574 0.067

***** Estimates for RE 1 "animal" ***************************************

No. of levels = 2204

Covariance structure = NRM

Order of fit = 2

Covariance matrix

1 0.83598E-01

2 0.10041E-01 0.10486E-01

Eigenvalues of covariance matrix

Value 0.08 0.01

(%) 90.29 9.71

Trace 0.09

Matrix of correlations and variance ratios

1 0.2250

2 0.3391 0.4256

Covariances & correlations with approximate sampling errors

4 COVS A 1 1 0.835984E-01 0.252280E-01 vrat 0.225 0.064

5 COVS A 1 2 0.100405E-01 0.490663E-02 corr 0.339 0.164

6 COVS A 2 2 0.104864E-01 0.195685E-02 vrat 0.426 0.067

***** Estimates of phenotypic covariances ***********************************

Covariance matrix

1 0.37154

2 0.40548E-02 0.24638E-01

Eigenvalues of covariance matrix

Value 0.37 0.02

(%) 93.79 6.21

Trace 0.40

Correlation matrix

1 1.0000

2 0.0424 1.0000

Covariances & correlations with approximate sampling errors

7 COVS T 1 1 0.371541 0.181340E-01

8 COVS T 1 2 0.405482E-02 0.337740E-02 corr 0.042 0.035

9 COVS T 2 2 0.246375E-01 0.127145E-02

======== end of file ============================01-04-2014==========17:41====

======= Version 30-08-2013 ======================================= **KM** ====

Program WOMBAT: Summary of Pedigree Information

==============================================================================

Two traits analysis (RFI x WS)

Analysis type : "muv 2"

Data file : "DADOS.dat"

Pedigree file : "ReducedPedFile.dat"

Parameter file : "wombat.par"

No. of animal IDs in data file = = 8091

No. of animal IDs in total = = 8490

*****Pedigree Structure for random effect : 1 ****************************

Original no. of animals = 8490

No. of animals after pruning = 8433

... proportion (%) remaining = 99.3

No. of levels w/out records = 342

No. of levels with records = 8091 100.0%

... 1 record(s) = 7149 88.4%

... 2 record(s) = 942 11.6%

No. of animals w/out offspring = 6052 71.8%

No. of animals with offspring = 2381 28.2%

... and records = 2039 24.2%

No. of animals with unknown sire = 313

No. of animals with unknown dam = 384

No. of animals with both parents unknown = 312

No. of animals with records =

... and unknown sire = 1

... and unknown dam = 53

... and both parents unknown = 0

No. of sires = 325

... with progeny in the data = 320

... with records & progeny in data = 288

No. of dams = 2056

... with progeny in the data = 2056

... with records & progeny in data = 1751

No. of animals with known/unpruned grand-parents

... with paternal grandsire = 7358

... with paternal granddam = 7056

... with maternal grandsire = 6535

... with maternal granddam = 6468

Inbreeding coefficients for random effect 1 computed

No. of inbred animals = 5144

Average inbreeding coefficient = 1.5417 (in %)

... amongst inbred animals = 2.5275 (in %)

random effect no. = 1 NRM

no. of elements in NRM/GIN inverse 31838

log determinant = -5688.7609053187680

random effect no. = 2 IDE

no. of elements in NRM/GIN inverse 0

log determinant = 0.0000000000000000

======== end of file ============================06-02-2015==========11:12====

======= Version 30-08-2013 ======================================= **KM** ====

Program WOMBAT: Summary of information from Set-up step

==============================================================================

Analysis type : "muv 2"

Data file : "DADOS.dat"

Pedigree file : "ReducedPedFile.dat"

Parameter file : "wombat.par"

No. of traits = 2

nrec mean sdev min. max.

1 "WS" 8078 299.703 49.6462 160.030 489.760

2 "RFI" 955 -0.112496E-01 0.608835 -2.31380 4.96420

Numbers of individuals/records for pairs of traits

1 2

1 "WS" 8078 942

2 "RFI" 942 955

Covariables

1"WS" nrec mean sdev min. max.

1 "idv(2)" 8078 6.63568 3.07616 2.00000 18.0000

2 "idade(1)" 8078 473.414 105.028 293.000 645.000

2"RFI" nrec mean sdev min. max.

1 "idv(2)" 955 6.15079 3.05304 3.00000 17.0000

2 "idade(1)" 955 369.980 37.3543 267.000 511.000

Fixed effects

1 "WS" nlev

1 "gc" 201

2 "mn" 4

2 "RFI" nlev

1 "gc" 21

Random effects nlev

1 "animal" 8433 NRM

2 "peanim" 2107 IDE

======== end of file ============================06-02-2015==========11:12====

======= Version 30-08-2013 ======================================= **KM** ====

Program WOMBAT: Estimates of covariance components

==============================================================================

Analysis type : "muv 2"

Data file : "DADOS.dat"

Pedigree file : "ReducedPedFile.dat"

Parameter file : "wombat.par"

No. of traits = 2 WS RFI

No. of records = 9033 8078 955

No. of parameters = 7

Maximum log L = -30200.015

-1/2 AIC & AICC = -30207.015 -30207.021

-1/2 BIC = -30231.805 "Penalty factor" = 4.541

Parameter estimates with approx. sampling erors

1 CHOL Z 1 1 3.02588 0.211281E-01

2 CHOL Z 1 2 0.127247E-01 0.304400E-01

3 CHOL Z 2 2 -0.623909 0.411267E-01

4 CHOL A 1 1 2.88223 0.479639E-01

5 CHOL A 1 2 0.514119E-01 0.407034E-01

6 CHOL A 2 2 -1.25068 0.159214

7 CHOL B 1 1 2.32941 0.507286E-01

Convergence criteria for last 3 iterates

Change in log likelihood = 0.153784 0.003746 0.000234

Change in parameter vector = 0.005222 0.000921 0.000222

Norm of gradient vector = 18.5338 1.4791 0.3647

Newton decrement = -0.2794 -0.0062 -0.0004

***** Estimates of residual covariances ************************************

Order of fit = 2

Covariance matrix

1 424.86

2 0.26228 0.28729

Eigenvalues of covariance matrix

Value 424.86 0.29

(%) 99.93 0.07

Trace 425.15

Matrix of correlations and variance ratios

1 0.5003

2 0.0237 0.7725

Covariances & correlations with approximate sampling errors

1 COVS Z 1 1 424.860 17.9530 vrat 0.500 0.027

2 COVS Z 1 2 0.262282 0.627550 corr 0.024 0.057

3 COVS Z 2 2 0.287293 0.235938E-01 vrat 0.772 0.065

***** Estimates for RE 1 "animal" ***************************************

No. of levels = 8433

Covariance structure = NRM

Order of fit = 2

Covariance matrix

1 318.77

2 0.91791 0.84617E-01

Eigenvalues of covariance matrix

Value 318.77 0.08

(%) 99.97 0.03

Trace 318.85

Matrix of correlations and variance ratios

1 0.3754

2 0.1767 0.2275

Covariances & correlations with approximate sampling errors

4 COVS A 1 1 318.766 30.5785 vrat 0.375 0.030

5 COVS A 1 2 0.917909 0.728142 corr 0.177 0.143

6 COVS A 2 2 0.846174E-01 0.256339E-01 vrat 0.228 0.065

***** Estimates for RE 2 "peanim" ***************************************

No. of levels = 2107

Covariance structure = IDE

Order of fit = 1

Covariance matrix

1 105.51

Matrix of correlations and variance ratios

1 0.1243

Covariances & correlations with approximate sampling errors

7 COVS B 1 1 105.511 10.7048 vrat 0.124 0.012

***** Estimates of phenotypic covariances ***********************************

Covariance matrix

1 849.14

2 1.1802 0.37191

Eigenvalues of covariance matrix

Value 849.14 0.37

(%) 99.96 0.04

Trace 849.51

Correlation matrix

1 1.0000

2 0.0664 1.0000

Covariances & correlations with approximate sampling errors

8 COVS T 1 1 849.136 18.5602

9 COVS T 1 2 1.18019 0.579015 corr 0.066 0.032

10 COVS T 2 2 0.371910 0.181944E-01

======== end of file ============================06-02-2015==========11:13====

======= Version 30-08-2013 ======================================= **KM** ====

Program WOMBAT: Summary of Pedigree Information

==============================================================================

Two traits analysis (HH x RFI)

Analysis type : "muv 2"

Data file : "DADOS.dat"

Pedigree file : "ReducedPedFile.dat"

Parameter file : "wombat.par"

No. of animal IDs in data file = = 6560

No. of animal IDs in total = = 7100

*****Pedigree Structure for random effect : 1 ****************************

Original no. of animals = 7100

No. of animals after pruning = 7017

... proportion (%) remaining = 98.8

No. of levels w/out records = 457

No. of levels with records = 6560 100.0%

... 1 record(s) = 5617 85.6%

... 2 record(s) = 943 14.4%

No. of animals w/out offspring = 4829 68.8%

No. of animals with offspring = 2188 31.2%

... and records = 1731 24.7%

No. of animals with unknown sire = 148

No. of animals with unknown dam = 237

No. of animals with both parents unknown = 148

No. of animals with records =

... and unknown sire = 0

... and unknown dam = 18

... and both parents unknown = 0

No. of sires = 320

... with progeny in the data = 283

... with records & progeny in data = 240

No. of dams = 1868

... with progeny in the data = 1837

... with records & progeny in data = 1491

No. of animals with known/unpruned grand-parents

... with paternal grandsire = 6738

... with paternal granddam = 6484

... with maternal grandsire = 6339

... with maternal granddam = 6111

Inbreeding coefficients for random effect 1 computed

No. of inbred animals = 5149

Average inbreeding coefficient = 1.8504 (in %)

... amongst inbred animals = 2.5218 (in %)

random effect no. = 1 NRM

no. of elements in NRM/GIN inverse 26822

log determinant = -4814.8039049931958

======== end of file ============================06-02-2015==========11:15====

======= Version 30-08-2013 ======================================= **KM** ====

Program WOMBAT: Summary of information from Set-up step

==============================================================================

Analysis type : "muv 2"

Data file : "DADOS.dat"

Pedigree file : "ReducedPedFile.dat"

Parameter file : "wombat.par"

No. of traits = 2

nrec mean sdev min. max.

1 "HH" 6548 132.269 5.43028 100.000 149.000

2 "RFI" 955 -0.112496E-01 0.608835 -2.31380 4.96420

Numbers of individuals/records for pairs of traits

1 2

1 "HH" 6548 943

2 "RFI" 943 955

Covariables

1"HH" nrec mean sdev min. max.

1 "idv(2)" 6548 6.40043 2.93455 2.00000 17.0000

2 "idade(1)" 6548 472.701 107.333 293.000 645.000

2"RFI" nrec mean sdev min. max.

1 "idv(2)" 955 6.15079 3.05304 3.00000 17.0000

2 "idade(1)" 955 369.980 37.3543 267.000 511.000

Fixed effects

1 "HH" nlev

1 "gc" 169

2 "mn" 4

2 "RFI" nlev

1 "gc" 21

Random effects nlev

1 "animal" 7017 NRM

======== end of file ============================06-02-2015==========11:15====

======= Version 30-08-2013 ======================================= **KM** ====

Program WOMBAT: Estimates of covariance components

==============================================================================

Analysis type : "muv 2"

Data file : "DADOS.dat"

Pedigree file : "ReducedPedFile.dat"

Parameter file : "wombat.par"

No. of traits = 2 HH RFI

No. of records = 7503 6548 955

No. of parameters = 6

Maximum log L = -11451.878

-1/2 AIC & AICC = -11457.878 -11457.883

-1/2 BIC = -11478.566 "Penalty factor" = 4.448

Parameter estimates with approx. sampling erors

1 CHOL Z 1 1 0.897233 0.266288E-01

2 CHOL Z 1 2 0.214313E-01 0.376436E-01

3 CHOL Z 2 2 -0.632626 0.419231E-01

4 CHOL A 1 1 1.12004 0.316239E-01

5 CHOL A 1 2 0.200441E-01 0.402054E-01

6 CHOL A 2 2 -1.19907 0.144682

Convergence criteria for last 3 iterates

Change in log likelihood = 0.090854 0.001472 0.000032

Change in parameter vector = 0.007213 0.001030 0.000153

Norm of gradient vector = 18.0621 1.3741 0.1903

Newton decrement = -0.1644 -0.0026 -0.0001

***** Estimates of residual covariances ************************************

Order of fit = 2

Covariance matrix

1 6.0163

2 0.52567E-01 0.28263

Eigenvalues of covariance matrix

Value 6.02 0.28

(%) 95.52 4.48

Trace 6.30

Matrix of correlations and variance ratios

1 0.3904

2 0.0403 0.7559

Covariances & correlations with approximate sampling errors

1 COVS Z 1 1 6.01626 0.320412 vrat 0.390 0.026

2 COVS Z 1 2 0.525668E-01 0.923502E-01 corr 0.040 0.071

3 COVS Z 2 2 0.282627 0.235791E-01 vrat 0.756 0.065

***** Estimates for RE 1 "animal" ***************************************

No. of levels = 7017

Covariance structure = NRM

Order of fit = 2

Covariance matrix

1 9.3941

2 0.61435E-01 0.91288E-01

Eigenvalues of covariance matrix

Value 9.39 0.09

(%) 99.04 0.96

Trace 9.49

Matrix of correlations and variance ratios

1 0.6096

2 0.0663 0.2441

Covariances & correlations with approximate sampling errors

4 COVS A 1 1 9.39415 0.594159 vrat 0.610 0.026

5 COVS A 1 2 0.614351E-01 0.123278 corr 0.066 0.134

6 COVS A 2 2 0.912883E-01 0.261664E-01 vrat 0.244 0.065

***** Estimates of phenotypic covariances ***********************************

Covariance matrix

1 15.410

2 0.11400 0.37392

Eigenvalues of covariance matrix

Value 15.41 0.37

(%) 97.64 2.36

Trace 15.78

Correlation matrix

1 1.0000

2 0.0475 1.0000

Covariances & correlations with approximate sampling errors

7 COVS T 1 1 15.4104 0.382707

8 COVS T 1 2 0.114002 0.879269E-01 corr 0.047 0.037

9 COVS T 2 2 0.373916 0.184121E-01

======== end of file ============================06-02-2015==========11:15====

======= Version 30-08-2013 ======================================= **KM** ====

Program WOMBAT: Summary of Pedigree Information

==============================================================================

Two traits analysis (CC x RFI)

Analysis type : "muv 2"

Data file : "DADOS.dat"

Pedigree file : "ReducedPedFile.dat"

Parameter file : "wombat.par"

No. of animal IDs in data file = = 3943

No. of animal IDs in total = = 5418

*****Pedigree Structure for random effect : 1 ****************************

Original no. of animals = 5418

No. of animals after pruning = 5330

... proportion (%) remaining = 98.4

No. of levels w/out records = 1387

No. of levels with records = 3943 100.0%

... 1 record(s) = 3055 77.5%

... 2 record(s) = 888 22.5%

No. of animals w/out offspring = 3311 62.1%

No. of animals with offspring = 2019 37.9%

... and records = 632 11.9%

No. of animals with unknown sire = 94

No. of animals with unknown dam = 188

No. of animals with both parents unknown = 94

No. of animals with records =

... and unknown sire = 0

... and unknown dam = 0

... and both parents unknown = 0

No. of sires = 318

... with progeny in the data = 255

... with records & progeny in data = 217

No. of dams = 1701

... with progeny in the data = 1508

... with records & progeny in data = 412

No. of animals with known/unpruned grand-parents

... with paternal grandsire = 5142

... with paternal granddam = 4954

... with maternal grandsire = 4953

... with maternal granddam = 4738

Inbreeding coefficients for random effect 1 computed

No. of inbred animals = 4324

Average inbreeding coefficient = 2.1293 (in %)

... amongst inbred animals = 2.6247 (in %)

random effect no. = 1 NRM

no. of elements in NRM/GIN inverse 20464

log determinant = -3671.8274443710275

======== end of file ============================06-02-2015==========11:17====

======= Version 30-08-2013 ======================================= **KM** ====

Program WOMBAT: Summary of information from Set-up step

==============================================================================

Analysis type : "muv 2"

Data file : "DADOS.dat"

Pedigree file : "ReducedPedFile.dat"

Parameter file : "wombat.par"

No. of traits = 2

nrec mean sdev min. max.

1 "CC" 3876 164.140 8.72028 128.000 192.000

2 "RFI" 955 -0.112496E-01 0.608835 -2.31380 4.96420

Numbers of individuals/records for pairs of traits

1 2

1 "CC" 3876 888

2 "RFI" 888 955

Covariables

1"CC" nrec mean sdev min. max.

1 "idv(2)" 3876 6.27632 2.87660 2.00000 17.0000

2 "idade(1)" 3876 431.403 109.187 293.000 725.000

2"RFI" nrec mean sdev min. max.

1 "idv(2)" 955 6.15079 3.05304 3.00000 17.0000

2 "idade(1)" 955 369.980 37.3543 267.000 511.000

Fixed effects

1 "CC" nlev

1 "gc" 100

2 "mn" 4

2 "RFI" nlev

1 "gc" 21

Random effects nlev

1 "animal" 5330 NRM

======== end of file ============================06-02-2015==========11:17====

======= Version 30-08-2013 ======================================= **KM** ====

Program WOMBAT: Estimates of covariance components

==============================================================================

Analysis type : "muv 2"

Data file : "DADOS.dat"

Pedigree file : "ReducedPedFile.dat"

Parameter file : "wombat.par"

No. of traits = 2 CC RFI

No. of records = 4831 3876 955

No. of parameters = 6

Maximum log L = -8743.521

-1/2 AIC & AICC = -8749.521 -8749.530

-1/2 BIC = -8768.887 "Penalty factor" = 4.228

Parameter estimates with approx. sampling erors

1 CHOL Z 1 1 1.61712 0.234126E-01

2 CHOL Z 1 2 0.214866E-01 0.301317E-01

3 CHOL Z 2 2 -0.630014 0.413837E-01

4 CHOL A 1 1 1.18616 0.727547E-01

5 CHOL A 1 2 0.440513E-01 0.505870E-01

6 CHOL A 2 2 -1.21865 0.149521

Convergence criteria for last 3 iterates

Change in log likelihood = 1.704959 0.019217 0.000490

Change in parameter vector = 0.036505 0.005144 0.000886

Norm of gradient vector = 93.5501 5.9177 0.3911

Newton decrement = -3.2933 -0.0340 -0.0008

***** Estimates of residual covariances ************************************

Order of fit = 2

Covariance matrix

1 25.387

2 0.10826 0.28411

Eigenvalues of covariance matrix

Value 25.39 0.28

(%) 98.90 1.10

Trace 25.67

Matrix of correlations and variance ratios

1 0.7031

2 0.0403 0.7608

Covariances & correlations with approximate sampling errors

1 COVS Z 1 1 25.3873 1.18876 vrat 0.703 0.039

2 COVS Z 1 2 0.108262 0.151911 corr 0.040 0.057

3 COVS Z 2 2 0.284108 0.234811E-01 vrat 0.761 0.064

***** Estimates for RE 1 "animal" ***************************************

No. of levels = 5330

Covariance structure = NRM

Order of fit = 2

Covariance matrix

1 10.722

2 0.14425 0.89337E-01

Eigenvalues of covariance matrix

Value 10.72 0.09

(%) 99.19 0.81

Trace 10.81

Matrix of correlations and variance ratios

1 0.2969

2 0.1474 0.2392

Covariances & correlations with approximate sampling errors

4 COVS A 1 1 10.7223 1.56019 vrat 0.297 0.039

5 COVS A 1 2 0.144245 0.166133 corr 0.147 0.170

6 COVS A 2 2 0.893365E-01 0.258278E-01 vrat 0.239 0.064

***** Estimates of phenotypic covariances ***********************************

Covariance matrix

1 36.110

2 0.25251 0.37344

Eigenvalues of covariance matrix

Value 36.11 0.37

(%) 98.98 1.02

Trace 36.48

Correlation matrix

1 1.0000

2 0.0688 1.0000

Covariances & correlations with approximate sampling errors

7 COVS T 1 1 36.1096 0.973271

8 COVS T 1 2 0.252508 0.131050 corr 0.069 0.036

9 COVS T 2 2 0.373445 0.183364E-01

======== end of file ============================06-02-2015==========11:17====

======= Version 30-08-2013 ======================================= **KM** ====

Program WOMBAT: Summary of Pedigree Information

==============================================================================

Two traits analysis (LEA x RFI)

Analysis type : "muv 2"

Data file : "DADOS.dat"

Pedigree file : "ReducedPedFile.dat"

Parameter file : "wombat.par"

No. of animal IDs in data file = = 2396

No. of animal IDs in total = = 3756

*****Pedigree Structure for random effect : 1 ****************************

Original no. of animals = 3756

No. of animals after pruning = 3665

... proportion (%) remaining = 97.6

No. of levels w/out records = 1269

No. of levels with records = 2396 100.0%

... 1 record(s) = 1554 64.9%

... 2 record(s) = 842 35.1%

No. of animals w/out offspring = 1913 52.2%

No. of animals with offspring = 1752 47.8%

... and records = 483 13.2%

No. of animals with unknown sire = 64

No. of animals with unknown dam = 161

No. of animals with both parents unknown = 64

No. of animals with records =

... and unknown sire = 0

... and unknown dam = 0

... and both parents unknown = 0

No. of sires = 313

... with progeny in the data = 180

... with records & progeny in data = 85

No. of dams = 1439

... with progeny in the data = 1042

... with records & progeny in data = 392

No. of animals with known/unpruned grand-parents

... with paternal grandsire = 3520

... with paternal granddam = 3382

... with maternal grandsire = 3391

... with maternal granddam = 3267

random effect no. = 1 NRM

no. of elements in NRM/GIN inverse 14038

log determinant = -2522.5521618264124

======== end of file ============================06-02-2015==========11:25====

======= Version 30-08-2013 ======================================= **KM** ====

Program WOMBAT: Summary of information from Set-up step

==============================================================================

Analysis type : "muv 2"

Data file : "DADOS.dat"

Pedigree file : "ReducedPedFile.dat"

Parameter file : "wombat.par"

No. of traits = 2

nrec mean sdev min. max.

1 "LEA" 2283 51.4305 8.89390 21.4000 83.4000

2 "RFI" 955 -0.112496E-01 0.608835 -2.31380 4.96420

Numbers of individuals/records for pairs of traits

1 2

1 "LEA" 2283 842

2 "RFI" 842 955

Covariables

1"LEA" nrec mean sdev min. max.

1 "idv(2)" 2283 6.11082 2.92852 2.00000 17.0000

2 "idade(1)" 2283 448.951 98.8881 306.000 609.000

2"RFI" nrec mean sdev min. max.

1 "idv(2)" 955 6.15079 3.05304 3.00000 17.0000

2 "idade(1)" 955 369.980 37.3543 267.000 511.000

Fixed effects

1 "LEA" nlev

1 "gc" 65

2 "mn" 4

2 "RFI" nlev

1 "gc" 21

Random effects nlev

1 "animal" 3665 NRM

======== end of file ============================06-02-2015==========11:25====

======= Version 30-08-2013 ======================================= **KM** ====

Program WOMBAT: Estimates of covariance components

==============================================================================

Analysis type : "muv 2"

Data file : "DADOS.dat"

Pedigree file : "ReducedPedFile.dat"

Parameter file : "wombat.par"

No. of traits = 2 LEA RFI

No. of records = 3238 2283 955

No. of parameters = 6

Maximum log L = -5117.185

-1/2 AIC & AICC = -5123.185 -5123.198

-1/2 BIC = -5141.343 "Penalty factor" = 4.026

Parameter estimates with approx. sampling erors

1 CHOL Z 1 1 1.49149 0.394546E-01

2 CHOL Z 1 2 0.293371E-01 0.365469E-01

3 CHOL Z 2 2 -0.635380 0.418021E-01

4 CHOL A 1 1 1.41550 0.678692E-01

5 CHOL A 1 2 0.113249E-02 0.489015E-01

6 CHOL A 2 2 -1.18864 0.140812

Convergence criteria for last 3 iterates

Change in log likelihood = 0.842832 0.000941 0.000003

Change in parameter vector = 0.018673 0.000718 0.000106

Norm of gradient vector = 48.0037 1.5933 0.0339

Newton decrement = -1.6531 -0.0019 -0.0000

***** Estimates of residual covariances ************************************

Order of fit = 2

Covariance matrix

1 19.747

2 0.13037 0.28148

Eigenvalues of covariance matrix

Value 19.75 0.28

(%) 98.60 1.40

Trace 20.03

Matrix of correlations and variance ratios

1 0.5379

2 0.0553 0.7520

Covariances & correlations with approximate sampling errors

1 COVS Z 1 1 19.7467 1.55820 vrat 0.538 0.051

2 COVS Z 1 2 0.130366 0.162530 corr 0.055 0.069

3 COVS Z 2 2 0.281479 0.234736E-01 vrat 0.752 0.065

***** Estimates for RE 1 "animal" ***************************************

No. of levels = 3665

Covariance structure = NRM

Order of fit = 2

Covariance matrix

1 16.962

2 0.46642E-02 0.92804E-01

Eigenvalues of covariance matrix

Value 16.96 0.09

(%) 99.46 0.54

Trace 17.06

Matrix of correlations and variance ratios

1 0.4621

2 0.0037 0.2480

Covariances & correlations with approximate sampling errors

4 COVS A 1 1 16.9624 2.30245 vrat 0.462 0.051

5 COVS A 1 2 0.466421E-02 0.201414 corr 0.004 0.161

6 COVS A 2 2 0.928038E-01 0.261381E-01 vrat 0.248 0.065

***** Estimates of phenotypic covariances ***********************************

Covariance matrix

1 36.709

2 0.13503 0.37428

Eigenvalues of covariance matrix

Value 36.71 0.37

(%) 98.99 1.01

Trace 37.08

Correlation matrix

1 1.0000

2 0.0364 1.0000

Covariances & correlations with approximate sampling errors

7 COVS T 1 1 36.7091 1.36594

8 COVS T 1 2 0.135030 0.141402 corr 0.036 0.038

9 COVS T 2 2 0.374283 0.184455E-01

======== end of file ============================06-02-2015==========11:25====

======= Version 30-08-2013 ======================================= **KM** ====

Program WOMBAT: Summary of Pedigree Information

==============================================================================

Two traits analysis (BF x RFI)

Analysis type : "muv 2"

Data file : "DADOS.dat"

Pedigree file : "ReducedPedFile.dat"

Parameter file : "wombat.par"

No. of animal IDs in data file = = 2396

No. of animal IDs in total = = 3756

*****Pedigree Structure for random effect : 1 ****************************

Original no. of animals = 3756

No. of animals after pruning = 3665

... proportion (%) remaining = 97.6

No. of levels w/out records = 1269

No. of levels with records = 2396 100.0%

... 1 record(s) = 1552 64.8%

... 2 record(s) = 844 35.2%

No. of animals w/out offspring = 1913 52.2%

No. of animals with offspring = 1752 47.8%

... and records = 483 13.2%

No. of animals with unknown sire = 64

No. of animals with unknown dam = 161

No. of animals with both parents unknown = 64

No. of animals with records =

... and unknown sire = 0

... and unknown dam = 0

... and both parents unknown = 0

No. of sires = 313

... with progeny in the data = 180

... with records & progeny in data = 85

No. of dams = 1439

... with progeny in the data = 1042

... with records & progeny in data = 392

No. of animals with known/unpruned grand-parents

... with paternal grandsire = 3520

... with paternal granddam = 3382

... with maternal grandsire = 3391

... with maternal granddam = 3267

random effect no. = 1 NRM

no. of elements in NRM/GIN inverse 14038

log determinant = -2522.5521618264124

======== end of file ============================06-02-2015==========11:30====

======= Version 30-08-2013 ======================================= **KM** ====

Program WOMBAT: Summary of information from Set-up step

==============================================================================

Analysis type : "muv 2"

Data file : "DADOS.dat"

Pedigree file : "ReducedPedFile.dat"

Parameter file : "wombat.par"

No. of traits = 2

nrec mean sdev min. max.

1 "BF" 2285 1.75922 1.42798 0.00000 10.5000

2 "RFI" 955 -0.112496E-01 0.608835 -2.31380 4.96420

Numbers of individuals/records for pairs of traits

1 2

1 "BF" 2285 844

2 "RFI" 844 955

Covariables

1"BF" nrec mean sdev min. max.

1 "idv(2)" 2285 6.10810 2.92868 2.00000 17.0000

2 "idade(1)" 2285 449.081 98.9421 306.000 609.000

2"RFI" nrec mean sdev min. max.

1 "idv(2)" 955 6.15079 3.05304 3.00000 17.0000

2 "idade(1)" 955 369.980 37.3543 267.000 511.000

Fixed effects

1 "BF" nlev

1 "gc" 65

2 "mn" 4

2 "RFI" nlev

1 "gc" 21

Random effects nlev

1 "animal" 3665 NRM

======== end of file ============================06-02-2015==========11:30====

======= Version 30-08-2013 ======================================= **KM** ====

Program WOMBAT: Estimates of covariance components

==============================================================================

analise bi

Analysis type : "muv 2"

Data file : "DADOS.dat"

Pedigree file : "ReducedPedFile.dat"

Parameter file : "wombat.par"

No. of traits = 2 BF RFI

No. of records = 3240 2285 955

No. of parameters = 6

Maximum log L = -1107.491

-1/2 AIC & AICC = -1113.491 -1113.504

-1/2 BIC = -1131.652 "Penalty factor" = 4.027

Parameter estimates with approx. sampling erors

1 CHOL Z 1 1 -0.220573 0.291425E-01

2 CHOL Z 1 2 -0.288459E-01 0.308953E-01

3 CHOL Z 2 2 -0.625090 0.404842E-01

4 CHOL A 1 1 -0.642614 0.853754E-01

5 CHOL A 1 2 0.109541 0.499563E-01

6 CHOL A 2 2 -1.30981 0.177603

Convergence criteria for last 3 iterates

Change in log likelihood = 12.172820 0.027414 0.000043

Change in parameter vector = 0.100683 0.008670 0.000748

Norm of gradient vector = 263.7572 10.9610 0.2617

Newton decrement = -23.3410 -0.0559 -0.0001

***** Estimates of residual covariances ************************************

Order of fit = 2

Covariance matrix

1 0.64330

2 -0.23136E-01 0.28729

Eigenvalues of covariance matrix

Value 0.64 0.29

(%) 69.29 30.71

Trace 0.93

Matrix of correlations and variance ratios

1 0.6993

2 -0.0538 0.7720

Covariances & correlations with approximate sampling errors

1 COVS Z 1 1 0.643299 0.374947E-01 vrat 0.699 0.046

2 COVS Z 1 2 -0.231362E-01 0.247539E-01 corr -0.054 0.058

3 COVS Z 2 2 0.287286 0.231115E-01 vrat 0.772 0.062

***** Estimates for RE 1 "animal" ***************************************

No. of levels = 3665

Covariance structure = NRM

Order of fit = 2

Covariance matrix

1 0.27659

2 0.57610E-01 0.84830E-01

Eigenvalues of covariance matrix

Value 0.29 0.07

(%) 80.95 19.05

Trace 0.36

Matrix of correlations and variance ratios

1 0.3007

2 0.3761 0.2280

Covariances & correlations with approximate sampling errors

4 COVS A 1 1 0.276587 0.472276E-01 vrat 0.301 0.046

5 COVS A 1 2 0.576096E-01 0.265106E-01 corr 0.376 0.173

6 COVS A 2 2 0.848303E-01 0.248445E-01 vrat 0.228 0.062

***** Estimates of phenotypic covariances ***********************************

Covariance matrix

1 0.91989

2 0.34473E-01 0.37212

Eigenvalues of covariance matrix

Value 0.92 0.37

(%) 71.37 28.63

Trace 1.29

Correlation matrix

1 1.0000

2 0.0589 1.0000

Covariances & correlations with approximate sampling errors

7 COVS T 1 1 0.919887 0.311574E-01

8 COVS T 1 2 0.344734E-01 0.207100E-01 corr 0.059 0.035

9 COVS T 2 2 0.372116 0.181382E-01

======== end of file ============================06-02-2015==========11:30====

======= Version 30-08-2013 ======================================= **KM** ====

Program WOMBAT: Summary of Pedigree Information

==============================================================================

Two traits analysis (RF x RFI)

Analysis type : "muv 2"

Data file : "DADOS.dat"

Pedigree file : "ReducedPedFile.dat"

Parameter file : "wombat.par"

No. of animal IDs in data file = = 1930

No. of animal IDs in total = = 3174

*****Pedigree Structure for random effect : 1 ****************************

Original no. of animals = 3174

No. of animals after pruning = 3089

... proportion (%) remaining = 97.3

No. of levels w/out records = 1159

No. of levels with records = 1930 100.0%

... 1 record(s) = 1088 56.4%

... 2 record(s) = 842 43.6%

No. of animals w/out offspring = 1497 48.5%

No. of animals with offspring = 1592 51.5%

... and records = 433 14.0%

No. of animals with unknown sire = 60

No. of animals with unknown dam = 147

No. of animals with both parents unknown = 59

No. of animals with records =

... and unknown sire = 0

... and unknown dam = 0

... and both parents unknown = 0

No. of sires = 307

... with progeny in the data = 114

... with records & progeny in data = 44

No. of dams = 1285

... with progeny in the data = 792

... with records & progeny in data = 389

No. of animals with known/unpruned grand-parents

... with paternal grandsire = 2965

... with paternal granddam = 2839

... with maternal grandsire = 2840

... with maternal granddam = 2751

random effect no. = 1 NRM

no. of elements in NRM/GIN inverse 11801

log determinant = -2124.3928089367182

======== end of file ============================06-02-2015==========11:33====

======= Version 30-08-2013 ======================================= **KM** ====

Program WOMBAT: Summary of information from Set-up step

==============================================================================

Analysis type : "muv 2"

Data file : "DADOS.dat"

Pedigree file : "ReducedPedFile.dat"

Parameter file : "wombat.par"

No. of traits = 2

nrec mean sdev min. max.

1 "RF" 1817 5.08663 2.54341 0.00000 19.2000

2 "RFI" 955 -0.112496E-01 0.608835 -2.31380 4.96420

Numbers of individuals/records for pairs of traits

1 2

1 "RF" 1817 842

2 "RFI" 842 955

Covariables

1"RF" nrec mean sdev min. max.

1 "idv(2)" 1817 6.09191 3.03138 2.00000 17.0000

2 "idade(1)" 1817 469.100 100.703 306.000 609.000

2"RFI" nrec mean sdev min. max.

1 "idv(2)" 955 6.15079 3.05304 3.00000 17.0000

2 "idade(1)" 955 369.980 37.3543 267.000 511.000

Fixed effects

1 "RF" nlev

1 "gc" 51

2 "mn" 3

2 "RFI" nlev

1 "gc" 21

Random effects nlev

1 "animal" 3089 NRM

======== end of file ============================06-02-2015==========11:33====

======= Version 30-08-2013 ======================================= **KM** ====

Program WOMBAT: Estimates of covariance components

==============================================================================

Analysis type : "muv 2"

Data file : "DADOS.dat"

Pedigree file : "ReducedPedFile.dat"

Parameter file : "wombat.par"

No. of traits = 2 RF RFI

No. of records = 2772 1817 955

No. of parameters = 6

Maximum log L = -1780.231

-1/2 AIC & AICC = -1786.231 -1786.246

-1/2 BIC = -1803.925 "Penalty factor" = 3.949

Parameter estimates with approx. sampling erors

1 CHOL Z 1 1 0.235201 0.365365E-01

2 CHOL Z 1 2 -0.241257E-01 0.333460E-01

3 CHOL Z 2 2 -0.628287 0.412286E-01

4 CHOL A 1 1 0.145960E-02 0.804316E-01

5 CHOL A 1 2 0.895686E-01 0.478935E-01

6 CHOL A 2 2 -1.26505 0.162597

Convergence criteria for last 3 iterates

Change in log likelihood = 7.457050 0.020076 0.000013

Change in parameter vector = 0.075630 0.007248 0.000545

Norm of gradient vector = 176.0749 8.4582 0.1021

Newton decrement = -14.4083 -0.0404 -0.0000

***** Estimates of residual covariances ************************************

Order of fit = 2

Covariance matrix

1 1.6006

2 -0.30523E-01 0.28521

Eigenvalues of covariance matrix

Value 1.60 0.28

(%) 84.91 15.09

Trace 1.89

Matrix of correlations and variance ratios

1 0.6148

2 -0.0452 0.7649

Covariances & correlations with approximate sampling errors

1 COVS Z 1 1 1.60064 0.116963 vrat 0.615 0.053

2 COVS Z 1 2 -0.305229E-01 0.421168E-01 corr -0.045 0.063

3 COVS Z 2 2 0.285210 0.233590E-01 vrat 0.765 0.064

***** Estimates for RE 1 "animal" ***************************************

No. of levels = 3089

Covariance structure = NRM

Order of fit = 2

Covariance matrix

1 1.0029

2 0.89699E-01 0.87673E-01

Eigenvalues of covariance matrix

Value 1.01 0.08

(%) 92.76 7.24

Trace 1.09

Matrix of correlations and variance ratios

1 0.3852

2 0.3025 0.2351

Covariances & correlations with approximate sampling errors

4 COVS A 1 1 1.00292 0.161333 vrat 0.385 0.053

5 COVS A 1 2 0.896995E-01 0.485346E-01 corr 0.302 0.162

6 COVS A 2 2 0.876731E-01 0.254986E-01 vrat 0.235 0.064

***** Estimates of phenotypic covariances ***********************************

Covariance matrix

1 2.6036

2 0.59177E-01 0.37288

Eigenvalues of covariance matrix

Value 2.61 0.37

(%) 87.52 12.48

Trace 2.98

Correlation matrix

1 1.0000

2 0.0601 1.0000

Covariances & correlations with approximate sampling errors

7 COVS T 1 1 2.60356 0.103010

8 COVS T 1 2 0.591766E-01 0.360789E-01 corr 0.060 0.036

9 COVS T 2 2 0.372883 0.182618E-01

======== end of file ============================06-02-2015==========11:33====

======= Version 30-08-2013 ======================================= **KM** ====

Program WOMBAT: Summary of Pedigree Information

==============================================================================

Two traits analysis (GAIN:FEED x DMI)

Analysis type : "muv 2"

Data file : "DADOS.dat"

Pedigree file : "ReducedPedFile.dat"

Parameter file : "wombat.par"

No. of animal IDs in data file = = 955

No. of animal IDs in total = = 2288

*****Pedigree Structure for random effect : 1 ****************************

Original no. of animals = 2288

No. of animals after pruning = 2204

... proportion (%) remaining = 96.3

No. of levels w/out records = 1249

No. of levels with records = 955 100.0%

... 2 record(s) = 955 100.0%

No. of animals w/out offspring = 828 37.6%

No. of animals with offspring = 1376 62.4%

... and records = 127 5.8%

No. of animals with unknown sire = 59

No. of animals with unknown dam = 145

No. of animals with both parents unknown = 58

No. of animals with records =

... and unknown sire = 0

... and unknown dam = 0

... and both parents unknown = 0

No. of sires = 296

... with progeny in the data = 78

... with records & progeny in data = 18

No. of dams = 1080

... with progeny in the data = 542

... with records & progeny in data = 98

No. of animals with known/unpruned grand-parents

... with paternal grandsire = 2083

... with paternal granddam = 1957

... with maternal grandsire = 1959

... with maternal granddam = 1872

random effect no. = 1 NRM

no. of elements in NRM/GIN inverse 8375

log determinant = -1487.8684515369519

======== end of file ============================25-02-2015==========18:25====

======= Version 30-08-2013 ======================================= **KM** ====

Program WOMBAT: Summary of information from Set-up step

==============================================================================

Analysis type : "muv 2"

Data file : "DADOS.dat"

Pedigree file : "ReducedPedFile.dat"

Parameter file : "wombat.par"

No. of traits = 2

nrec mean sdev min. max.

1 "G:F" 955 148.845 32.9032 40.4900 273.954

2 "DMI" 955 6.83104 1.33500 2.15500 12.6400

Numbers of individuals/records for pairs of traits

1 2

1 "G:F" 955 955

2 "DMI" 955 955

Covariables

1"G:F" nrec mean sdev min. max.

1 "idv(2)" 955 6.15079 3.05304 3.00000 17.0000

2 "idade(1)" 955 369.980 37.3543 267.000 511.000

2"DMI" nrec mean sdev min. max.

1 "idv(2)" 955 6.15079 3.05304 3.00000 17.0000

2 "idade(1)" 955 369.980 37.3543 267.000 511.000

Fixed effects

1 "G:F" nlev

1 "gc" 21

2 "DMI" nlev

1 "gc" 21

Random effects nlev

1 "animal" 2204 NRM

======== end of file ============================25-02-2015==========18:25====

======= Version 30-08-2013 ======================================= **KM** ====

Program WOMBAT: Estimates of covariance components

==============================================================================

Analysis type : "muv 2"

Data file : "DADOS.dat"

Pedigree file : "ReducedPedFile.dat"

Parameter file : "wombat.par"

No. of traits = 2 G:F DMI

No. of records = 1910 955 955

No. of parameters = 6

Maximum log L = -3661.724

-1/2 AIC & AICC = -3667.724 -3667.746

-1/2 BIC = -3684.312 "Penalty factor" = 3.765

Parameter estimates with approx. sampling erors

1 CHOL Z 1 1 19.5454 0.729183

2 CHOL Z 1 2 -0.247562 0.400344E-01

3 CHOL Z 2 2 0.594391 0.369014E-01

4 CHOL A 1 1 2.11424 0.205301

5 CHOL A 1 2 -0.705544E-01 0.110570

6 CHOL A 2 2 -0.507497 0.856376E-01

Convergence criteria for last 3 iterates

Change in log likelihood = 8.493630 0.092989 0.000029

Change in parameter vector = 0.099811 0.013161 0.000121

Norm of gradient vector = 33.1966 2.1972 0.0735

Newton decrement = -16.1875 -0.1860 -0.0001

***** Estimates of residual covariances ************************************

Order of fit = 2

Covariance matrix

1 382.02

2 -4.8387 0.41459

Eigenvalues of covariance matrix

Value 382.09 0.35

(%) 99.91 0.09

Trace 382.44

Matrix of correlations and variance ratios

1 0.8477

2 -0.3845 0.5302

Covariances & correlations with approximate sampling errors

1 COVS Z 1 1 382.025 28.5044 vrat 0.848 0.060

2 COVS Z 1 2 -4.83870 0.839264 corr -0.384 0.057

3 COVS Z 2 2 0.414588 0.475834E-01 vrat 0.530 0.069

***** Estimates for RE 1 "animal" ***************************************

No. of levels = 2204

Covariance structure = NRM

Order of fit = 2

Covariance matrix

1 68.613

2 -0.58442 0.36738

Eigenvalues of covariance matrix

Value 68.62 0.36

(%) 99.47 0.53

Trace 68.98

Matrix of correlations and variance ratios

1 0.1523

2 -0.1164 0.4698

Covariances & correlations with approximate sampling errors

4 COVS A 1 1 68.6126 28.1724 vrat 0.152 0.060

5 COVS A 1 2 -0.584421 0.949901 corr -0.116 0.180

6 COVS A 2 2 0.367382 0.650536E-01 vrat 0.470 0.069

***** Estimates of phenotypic covariances ***********************************

Covariance matrix

1 450.64

2 -5.4231 0.78197

Eigenvalues of covariance matrix

Value 450.70 0.72

(%) 99.84 0.16

Trace 451.42

Correlation matrix

1 1.0000

2 -0.2889 1.0000

Covariances & correlations with approximate sampling errors

7 COVS T 1 1 450.637 21.6380

8 COVS T 1 2 -5.42312 0.673363 corr -0.289 0.032

9 COVS T 2 2 0.781970 0.410349E-01

======== end of file ============================25-02-2015==========18:25====

======= Version 30-08-2013 ======================================= **KM** ====

Program WOMBAT: Summary of Pedigree Information

==============================================================================

Two traits analysis (GAIN:FEED x ADG)

Analysis type : "muv 2"

Data file : "DADOS.dat"

Pedigree file : "ReducedPedFile.dat"

Parameter file : "wombat.par"

No. of animal IDs in data file = = 955

No. of animal IDs in total = = 2288

*****Pedigree Structure for random effect : 1 ****************************

Original no. of animals = 2288

No. of animals after pruning = 2204

... proportion (%) remaining = 96.3

No. of levels w/out records = 1249

No. of levels with records = 955 100.0%

... 2 record(s) = 955 100.0%

No. of animals w/out offspring = 828 37.6%

No. of animals with offspring = 1376 62.4%

... and records = 127 5.8%

No. of animals with unknown sire = 59

No. of animals with unknown dam = 145

No. of animals with both parents unknown = 58

No. of animals with records =

... and unknown sire = 0

... and unknown dam = 0

... and both parents unknown = 0

No. of sires = 296

... with progeny in the data = 78

... with records & progeny in data = 18

No. of dams = 1080

... with progeny in the data = 542

... with records & progeny in data = 98

No. of animals with known/unpruned grand-parents

... with paternal grandsire = 2083

... with paternal granddam = 1957

... with maternal grandsire = 1959

... with maternal granddam = 1872

random effect no. = 1 NRM

no. of elements in NRM/GIN inverse 8375

log determinant = -1487.8684515369519

======== end of file ============================25-02-2015==========18:27====

======= Version 30-08-2013 ======================================= **KM** ====

Program WOMBAT: Summary of information from Set-up step

==============================================================================

Analysis type : "muv 2"

Data file : "DADOS.dat"

Pedigree file : "ReducedPedFile.dat"

Parameter file : "wombat.par"

No. of traits = 2

nrec mean sdev min. max.

1 "G:F" 955 148.845 32.9032 40.4900 273.954

2 "ADG" 955 1.00846 0.260202 0.176000 1.71800

Numbers of individuals/records for pairs of traits

1 2

1 "G:F" 955 955

2 "ADG" 955 955

Covariables

1"G:F" nrec mean sdev min. max.

1 "idv(2)" 955 6.15079 3.05304 3.00000 17.0000

2 "idade(1)" 955 369.980 37.3543 267.000 511.000

2"ADG" nrec mean sdev min. max.

1 "idv(2)" 955 6.15079 3.05304 3.00000 17.0000

2 "idade(1)" 955 369.980 37.3543 267.000 511.000

Fixed effects

1 "G:F" nlev

1 "gc" 21

2 "ADG" nlev

1 "gc" 21

Random effects nlev

1 "animal" 2204 NRM

======== end of file ============================25-02-2015==========18:27====

======= Version 30-08-2013 ======================================= **KM** ====

Program WOMBAT: Estimates of covariance components

==============================================================================

Analysis type : "muv 2"

Data file : "DADOS.dat"

Pedigree file : "ReducedPedFile.dat"

Parameter file : "wombat.par"

No. of traits = 2 G:F ADG

No. of records = 1910 955 955

No. of parameters = 6

Maximum log L = -1896.176

-1/2 AIC & AICC = -1902.176 -1902.198

-1/2 BIC = -1918.765 "Penalty factor" = 3.765

Parameter estimates with approx. sampling erors

1 CHOL Z 1 1 2.97309 0.372659E-01

2 CHOL Z 1 2 0.820679E-01 0.655708E-02

3 CHOL Z 2 2 -2.42151 0.611206E-01

4 CHOL A 1 1 2.11188 0.205901

5 CHOL A 1 2 0.389643E-01 0.171374E-01

6 CHOL A 2 2 -2.39657 0.826148E-01

Convergence criteria for last 3 iterates

Change in log likelihood = 6.403221 0.023012 0.000012

Change in parameter vector = 0.023285 0.002726 0.000178

Norm of gradient vector = 264.6444 37.5715 0.3865

Newton decrement = -12.2157 -0.0463 -0.0000

***** Estimates of residual covariances ************************************

Order of fit = 2

Covariance matrix

1 382.29

2 1.6046 0.14618E-01

Eigenvalues of covariance matrix

Value 382.29 0.01

(%) 100.00 0.00

Trace 382.30

Matrix of correlations and variance ratios

1 0.8484

2 0.6788 0.5986

Covariances & correlations with approximate sampling errors

1 COVS Z 1 1 382.287 28.4925 vrat 0.848 0.060

2 COVS Z 1 2 1.60460 0.164720 corr 0.679 0.037

3 COVS Z 2 2 0.146184E-01 0.144842E-02 vrat 0.599 0.065

***** Estimates for RE 1 "animal" ***************************************

No. of levels = 2204

Covariance structure = NRM

Order of fit = 2

Covariance matrix

1 68.289

2 0.32199 0.98046E-02

Eigenvalues of covariance matrix

Value 68.29 0.01

(%) 99.99 0.01

Trace 68.30

Matrix of correlations and variance ratios

1 0.1516

2 0.3935 0.4014

Covariances & correlations with approximate sampling errors

4 COVS A 1 1 68.2894 28.1217 vrat 0.152 0.060

5 COVS A 1 2 0.321991 0.176891 corr 0.394 0.151

6 COVS A 2 2 0.980457E-02 0.184168E-02 vrat 0.401 0.065

***** Estimates of phenotypic covariances ***********************************

Covariance matrix

1 450.58

2 1.9266 0.24423E-01

Eigenvalues of covariance matrix

Value 450.58 0.02

(%) 100.00 0.00

Trace 450.60

Correlation matrix

1 1.0000

2 0.5808 1.0000

Covariances & correlations with approximate sampling errors

7 COVS T 1 1 450.576 21.6290

8 COVS T 1 2 1.92660 0.131060 corr 0.581 0.023

9 COVS T 2 2 0.244230E-01 0.123840E-02

======== end of file ============================25-02-2015==========18:27====

======= Version 30-08-2013 ======================================= **KM** ====

Program WOMBAT: Summary of Pedigree Information

==============================================================================

Two traits analysis (WS x GAIN:FEED)

Analysis type : "muv 2"

Data file : "DADOS.dat"

Pedigree file : "ReducedPedFile.dat"

Parameter file : "wombat.par"

No. of animal IDs in data file = = 8091

No. of animal IDs in total = = 8490

*****Pedigree Structure for random effect : 1 ****************************

Original no. of animals = 8490

No. of animals after pruning = 8433

... proportion (%) remaining = 99.3

No. of levels w/out records = 342

No. of levels with records = 8091 100.0%

... 1 record(s) = 7149 88.4%

... 2 record(s) = 942 11.6%

No. of animals w/out offspring = 6052 71.8%

No. of animals with offspring = 2381 28.2%

... and records = 2039 24.2%

No. of animals with unknown sire = 313

No. of animals with unknown dam = 384

No. of animals with both parents unknown = 312

No. of animals with records =

... and unknown sire = 1

... and unknown dam = 53

... and both parents unknown = 0

No. of sires = 325

... with progeny in the data = 320

... with records & progeny in data = 288

No. of dams = 2056

... with progeny in the data = 2056

... with records & progeny in data = 1751

No. of animals with known/unpruned grand-parents

... with paternal grandsire = 7358

... with paternal granddam = 7056

... with maternal grandsire = 6535

... with maternal granddam = 6468

random effect no. = 1 NRM

no. of elements in NRM/GIN inverse 31838

log determinant = -5688.7609053187680

random effect no. = 2 IDE

no. of elements in NRM/GIN inverse 0

log determinant = 0.0000000000000000

======== end of file ============================05-02-2015==========10:07====

======= Version 30-08-2013 ======================================= **KM** ====

Program WOMBAT: Summary of information from Set-up step

==============================================================================

Analysis type : "muv 2"

Data file : "DADOS.dat"

Pedigree file : "ReducedPedFile.dat"

Parameter file : "wombat.par"

No. of traits = 2

nrec mean sdev min. max.

1 "WS" 8078 299.703 49.6462 160.030 489.760

2 "G:F" 955 148.845 32.9032 40.4900 273.954

Numbers of individuals/records for pairs of traits

1 2

1 "WS" 8078 942

2 "G:F" 942 955

Covariables

1"WS" nrec mean sdev min. max.

1 "idv(2)" 8078 6.63568 3.07616 2.00000 18.0000

2 "idade(1)" 8078 473.414 105.028 293.000 645.000

2"G:F" nrec mean sdev min. max.

1 "idv(2)" 955 6.15079 3.05304 3.00000 17.0000

2 "idade(1)" 955 369.980 37.3543 267.000 511.000

Fixed effects

1 "WS" nlev

1 "gc" 201

2 "mn" 4

2 "G:F" nlev

1 "gc" 21

Random effects nlev

1 "animal" 8433 NRM

2 "peanim" 2107 IDE

======== end of file ============================05-02-2015==========10:07====

======= Version 30-08-2013 ======================================= **KM** ====

Program WOMBAT: Estimates of covariance components

==============================================================================

Analysis type : "muv 2"

Data file : "DADOS.dat"

Pedigree file : "ReducedPedFile.dat"

Parameter file : "wombat.par"

No. of traits = 2 WS G:F

No. of records = 9033 8078 955

No. of parameters = 7

Maximum log L = -33507.832

-1/2 AIC & AICC = -33514.832 -33514.838

-1/2 BIC = -33539.622 "Penalty factor" = 4.541

Parameter estimates with approx. sampling erors

1 CHOL Z 1 1 20.6400 0.434130

2 CHOL Z 1 2 2.49802 1.03557

3 CHOL Z 2 2 19.3684 0.725533

4 CHOL A 1 1 17.7344 0.858711

5 CHOL A 1 2 0.812975 1.30473

6 CHOL A 2 2 8.19567 1.69419

7 CHOL B 1 1 2.34127 0.499271E-01

Convergence criteria for last 3 iterates

Change in log likelihood = 0.023659 0.001814 0.000146

Change in parameter vector = 0.005233 0.001417 0.000406

Norm of gradient vector = 0.3156 0.1540 0.0603

Newton decrement = -0.0376 -0.0028 -0.0002

***** Estimates of residual covariances ************************************

Order of fit = 2

Covariance matrix

1 426.01

2 51.559 381.38

Eigenvalues of covariance matrix

Value 459.87 347.51

(%) 56.96 43.04

Trace 807.38

Matrix of correlations and variance ratios

1 0.5020

2 0.1279 0.8490

Covariances & correlations with approximate sampling errors

1 COVS Z 1 1 426.009 17.9208 vrat 0.502 0.027

2 COVS Z 1 2 51.5590 21.4162 corr 0.128 0.053

3 COVS Z 2 2 381.376 28.3432 vrat 0.849 0.060

***** Estimates for RE 1 "animal" ***************************************

No. of levels = 8433

Covariance structure = NRM

Order of fit = 2

Covariance matrix

1 314.51

2 14.418 67.830

Eigenvalues of covariance matrix

Value 315.35 66.99

(%) 82.48 17.52

Trace 382.34

Matrix of correlations and variance ratios

1 0.3706

2 0.0987 0.1510

Covariances & correlations with approximate sampling errors

4 COVS A 1 1 314.508 30.4574 vrat 0.371 0.030

5 COVS A 1 2 14.4176 23.1743 corr 0.099 0.157

6 COVS A 2 2 67.8299 27.9344 vrat 0.151 0.060

***** Estimates for RE 2 "peanim" ***************************************

No. of levels = 2107

Covariance structure = IDE

Order of fit = 1

Covariance matrix

1 108.04

Matrix of correlations and variance ratios

1 0.1273

Covariances & correlations with approximate sampling errors

7 COVS B 1 1 108.044 10.7886 vrat 0.127 0.012

***** Estimates of phenotypic covariances ***********************************

Covariance matrix

1 848.56

2 65.977 449.21

Eigenvalues of covariance matrix

Value 859.18 438.59

(%) 66.20 33.80

Trace 1297.77

Correlation matrix

1 1.0000

2 0.1069 1.0000

Covariances & correlations with approximate sampling errors

8 COVS T 1 1 848.560 18.5150

9 COVS T 1 2 65.9766 19.7233 corr 0.107 0.032

10 COVS T 2 2 449.206 21.5281

======== end of file ============================05-02-2015==========10:07====

======= Version 30-08-2013 ======================================= **KM** ====

Program WOMBAT: Summary of Pedigree Information

==============================================================================

Two traits analysis (HH x GAIN:FEED)

Analysis type : "muv 2"

Data file : "DADOS.dat"

Pedigree file : "ReducedPedFile.dat"

Parameter file : "wombat.par"

No. of animal IDs in data file = = 6560

No. of animal IDs in total = = 7100

*****Pedigree Structure for random effect : 1 ****************************

Original no. of animals = 7100

No. of animals after pruning = 7017

... proportion (%) remaining = 98.8

No. of levels w/out records = 457

No. of levels with records = 6560 100.0%

... 1 record(s) = 5617 85.6%

... 2 record(s) = 943 14.4%

No. of animals w/out offspring = 4829 68.8%

No. of animals with offspring = 2188 31.2%

... and records = 1731 24.7%

No. of animals with unknown sire = 148

No. of animals with unknown dam = 237

No. of animals with both parents unknown = 148

No. of animals with records =

... and unknown sire = 0

... and unknown dam = 18

... and both parents unknown = 0

No. of sires = 320

... with progeny in the data = 283

... with records & progeny in data = 240

No. of dams = 1868

... with progeny in the data = 1837

... with records & progeny in data = 1491

No. of animals with known/unpruned grand-parents

... with paternal grandsire = 6738

... with paternal granddam = 6484

... with maternal grandsire = 6339

... with maternal granddam = 6111

random effect no. = 1 NRM

no. of elements in NRM/GIN inverse 26822

log determinant = -4814.8039049931958

======== end of file ============================05-02-2015==========10:03====

======= Version 30-08-2013 ======================================= **KM** ====

Program WOMBAT: Summary of information from Set-up step

==============================================================================

Analysis type : "muv 2"

Data file : "DADOS.dat"

Pedigree file : "ReducedPedFile.dat"

Parameter file : "wombat.par"

No. of traits = 2

nrec mean sdev min. max.

1 "HH" 6548 132.269 5.43028 100.000 149.000

2 "G:F" 955 148.845 32.9032 40.4900 273.954

Numbers of individuals/records for pairs of traits

1 2

1 "HH" 6548 943

2 "G:F" 943 955

Covariables

1"HH" nrec mean sdev min. max.

1 "idv(2)" 6548 6.40043 2.93455 2.00000 17.0000

2 "idade(1)" 6548 472.701 107.333 293.000 645.000

2"G:F" nrec mean sdev min. max.

1 "idv(2)" 955 6.15079 3.05304 3.00000 17.0000

2 "idade(1)" 955 369.980 37.3543 267.000 511.000

Fixed effects

1 "HH" nlev

1 "gc" 169

2 "mn" 4

2 "G:F" nlev

1 "gc" 21

Random effects nlev

1 "animal" 7017 NRM

======== end of file ============================05-02-2015==========10:03====

======= Version 30-08-2013 ======================================= **KM** ====

Program WOMBAT: Estimates of covariance components

==============================================================================

Analysis type : "muv 2"

Data file : "DADOS.dat"

Pedigree file : "ReducedPedFile.dat"

Parameter file : "wombat.par"

No. of traits = 2 HH G:F

No. of records = 7503 6548 955

No. of parameters = 6

Maximum log L = -14763.724

-1/2 AIC & AICC = -14769.724 -14769.729

-1/2 BIC = -14790.412 "Penalty factor" = 4.448

Parameter estimates with approx. sampling erors

1 CHOL Z 1 1 19.5233 0.730345

2 CHOL Z 1 2 -0.262901 0.161566

3 CHOL Z 2 2 2.43688 0.680593E-01

4 CHOL A 1 1 8.35354 1.69483

5 CHOL A 1 2 0.258379 0.477391

6 CHOL A 2 2 3.05682 0.104477

Convergence criteria for last 3 iterates

Change in log likelihood = 7.570033 0.081144 0.000118

Change in parameter vector = 0.087661 0.011223 0.000402

Norm of gradient vector = 10.3469 1.1113 0.1170

Newton decrement = -14.4490 -0.1609 -0.0002

***** Estimates of residual covariances ************************************

Order of fit = 2

Covariance matrix

1 6.0075

2 -5.1327 381.16

Eigenvalues of covariance matrix

Value 381.23 5.94

(%) 98.47 1.53

Trace 387.17

Matrix of correlations and variance ratios

1 0.3896

2 -0.1073 0.8453

Covariances & correlations with approximate sampling errors

1 COVS Z 1 1 6.00748 0.320538 vrat 0.390 0.026

2 COVS Z 1 2 -5.13270 3.15310 corr -0.107 0.066

3 COVS Z 2 2 381.160 28.5175 vrat 0.845 0.060

***** Estimates for RE 1 "animal" ***************************************

No. of levels = 7017

Covariance structure = NRM

Order of fit = 2

Covariance matrix

1 9.4109

2 2.1584 69.782

Eigenvalues of covariance matrix

Value 69.86 9.33

(%) 88.21 11.79

Trace 79.19

Matrix of correlations and variance ratios

1 0.6104

2 0.0842 0.1547

Covariances & correlations with approximate sampling errors

4 COVS A 1 1 9.41093 0.594886 vrat 0.610 0.026

5 COVS A 1 2 2.15838 3.99155 corr 0.084 0.156

6 COVS A 2 2 69.7817 28.3156 vrat 0.155 0.060

***** Estimates of phenotypic covariances ***********************************

Covariance matrix

1 15.418

2 -2.9743 450.94

Eigenvalues of covariance matrix

Value 450.96 15.40

(%) 96.70 3.30

Trace 466.36

Correlation matrix

1 1.0000

2 -0.0357 1.0000

Covariances & correlations with approximate sampling errors

7 COVS T 1 1 15.4184 0.383147

8 COVS T 1 2 -2.97431 2.96626 corr -0.036 0.036

9 COVS T 2 2 450.941 21.6768

======== end of file ============================05-02-2015==========10:03====

======= Version 30-08-2013 ======================================= **KM** ====

Program WOMBAT: Summary of Pedigree Information

==============================================================================

Two traits analysis (CC x GAIN:FEED)

Analysis type : "muv 2"

Data file : "DADOS.dat"

Pedigree file : "ReducedPedFile.dat"

Parameter file : "wombat.par"

No. of animal IDs in data file = = 3943

No. of animal IDs in total = = 5418

*****Pedigree Structure for random effect : 1 ****************************

Original no. of animals = 5418

No. of animals after pruning = 5330

... proportion (%) remaining = 98.4

No. of levels w/out records = 1387

No. of levels with records = 3943 100.0%

... 1 record(s) = 3055 77.5%

... 2 record(s) = 888 22.5%

No. of animals w/out offspring = 3311 62.1%

No. of animals with offspring = 2019 37.9%

... and records = 632 11.9%

No. of animals with unknown sire = 94

No. of animals with unknown dam = 188

No. of animals with both parents unknown = 94

No. of animals with records =

... and unknown sire = 0

... and unknown dam = 0

... and both parents unknown = 0

No. of sires = 318

... with progeny in the data = 255

... with records & progeny in data = 217

No. of dams = 1701

... with progeny in the data = 1508

... with records & progeny in data = 412

No. of animals with known/unpruned grand-parents

... with paternal grandsire = 5142

... with paternal granddam = 4954

... with maternal grandsire = 4953

... with maternal granddam = 4738

Inbreeding coefficients for random effect 1 computed

No. of inbred animals = 4324

Average inbreeding coefficient = 2.1293 (in %)

... amongst inbred animals = 2.6247 (in %)

random effect no. = 1 NRM

no. of elements in NRM/GIN inverse 20464

log determinant = -3671.8274443710275

======== end of file ============================05-02-2015==========10:13====

======= Version 30-08-2013 ======================================= **KM** ====

Program WOMBAT: Summary of information from Set-up step

==============================================================================

Analysis type : "muv 2"

Data file : "DADOS.dat"

Pedigree file : "ReducedPedFile.dat"

Parameter file : "wombat.par"

No. of traits = 2

nrec mean sdev min. max.

1 "CC" 3876 164.140 8.72028 128.000 192.000

2 "G:F" 955 148.845 32.9032 40.4900 273.954

Numbers of individuals/records for pairs of traits

1 2

1 "CC" 3876 888

2 "G:F" 888 955

Covariables

1"CC" nrec mean sdev min. max.

1 "idv(2)" 3876 6.27632 2.87660 2.00000 17.0000

2 "idade(1)" 3876 431.403 109.187 293.000 725.000

2"G:F" nrec mean sdev min. max.

1 "idv(2)" 955 6.15079 3.05304 3.00000 17.0000

2 "idade(1)" 955 369.980 37.3543 267.000 511.000

Fixed effects

1 "CC" nlev

1 "gc" 100

2 "mn" 4

2 "G:F" nlev

1 "gc" 21

Random effects nlev

1 "animal" 5330 NRM

======== end of file ============================05-02-2015==========10:13====

======= Version 30-08-2013 ======================================= **KM** ====

Program WOMBAT: Estimates of covariance components

==============================================================================

Analysis type : "muv 2"

Data file : "DADOS.dat"

Pedigree file : "ReducedPedFile.dat"

Parameter file : "wombat.par"

No. of traits = 2 CC G:F

No. of records = 4831 3876 955

No. of parameters = 6

Maximum log L = -12055.709

-1/2 AIC & AICC = -12061.709 -12061.718

-1/2 BIC = -12081.076 "Penalty factor" = 4.228

Parameter estimates with approx. sampling erors

1 CHOL Z 1 1 19.5724 0.725897

2 CHOL Z 1 2 0.259267 0.267910

3 CHOL Z 2 2 5.03502 0.118113

4 CHOL A 1 1 8.20498 1.69987

5 CHOL A 1 2 0.500473 0.657437

6 CHOL A 2 2 3.22860 0.254271

Convergence criteria for last 3 iterates

Change in log likelihood = 7.424502 0.077420 0.000031

Change in parameter vector = 0.087327 0.011613 0.000406

Norm of gradient vector = 8.9480 0.8080 0.0200

Newton decrement = -14.1848 -0.1545 -0.0001

***** Estimates of residual covariances ************************************

Order of fit = 2

Covariance matrix

1 25.419

2 5.0745 383.08

Eigenvalues of covariance matrix

Value 383.15 25.35

(%) 93.80 6.20

Trace 408.50

Matrix of correlations and variance ratios

1 0.7043

2 0.0514 0.8505

Covariances & correlations with approximate sampling errors

1 COVS Z 1 1 25.4187 1.18895 vrat 0.704 0.039

2 COVS Z 1 2 5.07448 5.25701 corr 0.051 0.053

3 COVS Z 2 2 383.079 28.4151 vrat 0.851 0.060

***** Estimates for RE 1 "animal" ***************************************

No. of levels = 5330

Covariance structure = NRM

Order of fit = 2

Covariance matrix

1 10.674

2 4.1064 67.322

Eigenvalues of covariance matrix

Value 67.62 10.38

(%) 86.69 13.31

Trace 78.00

Matrix of correlations and variance ratios

1 0.2957

2 0.1532 0.1495

Covariances & correlations with approximate sampling errors

4 COVS A 1 1 10.6744 1.55843 vrat 0.296 0.039

5 COVS A 1 2 4.10637 5.41793 corr 0.153 0.201

6 COVS A 2 2 67.3218 27.8947 vrat 0.149 0.060

***** Estimates of phenotypic covariances ***********************************

Covariance matrix

1 36.093

2 9.1808 450.40

Eigenvalues of covariance matrix

Value 450.60 35.89

(%) 92.62 7.38

Trace 486.49

Correlation matrix

1 1.0000

2 0.0720 1.0000

Covariances & correlations with approximate sampling errors

7 COVS T 1 1 36.0930 0.972202

8 COVS T 1 2 9.18085 4.48639 corr 0.072 0.035

9 COVS T 2 2 450.400 21.5967

======== end of file ============================05-02-2015==========10:13====

======= Version 30-08-2013 ======================================= **KM** ====

Program WOMBAT: Summary of Pedigree Information

==============================================================================

Two traits analysis (LEA x GAIN:FEED)

Analysis type : "muv 2"

Data file : "DADOS.dat"

Pedigree file : "ReducedPedFile.dat"

Parameter file : "wombat.par"

No. of animal IDs in data file = = 2396

No. of animal IDs in total = = 3756

*****Pedigree Structure for random effect : 1 ****************************

Original no. of animals = 3756

No. of animals after pruning = 3665

... proportion (%) remaining = 97.6

No. of levels w/out records = 1269

No. of levels with records = 2396 100.0%

... 1 record(s) = 1554 64.9%

... 2 record(s) = 842 35.1%

No. of animals w/out offspring = 1913 52.2%

No. of animals with offspring = 1752 47.8%

... and records = 483 13.2%

No. of animals with unknown sire = 64

No. of animals with unknown dam = 161

No. of animals with both parents unknown = 64

No. of animals with records =

... and unknown sire = 0

... and unknown dam = 0

... and both parents unknown = 0

No. of sires = 313

... with progeny in the data = 180

... with records & progeny in data = 85

No. of dams = 1439

... with progeny in the data = 1042

... with records & progeny in data = 392

No. of animals with known/unpruned grand-parents

... with paternal grandsire = 3520

... with paternal granddam = 3382

... with maternal grandsire = 3391

... with maternal granddam = 3267

random effect no. = 1 NRM

no. of elements in NRM/GIN inverse 14038

log determinant = -2522.5521618264124

======== end of file ============================01-04-2014==========10:18====

======= Version 30-08-2013 ======================================= **KM** ====

Program WOMBAT: Summary of information from Set-up step

==============================================================================

Analysis type : "muv 2"

Data file : "DADOS.dat"

Pedigree file : "ReducedPedFile.dat"

Parameter file : "wombat.par"

No. of traits = 2

nrec mean sdev min. max.

1 "LEA" 2283 51.4305 8.89390 21.4000 83.4000

2 "G:F" 955 148.845 32.9032 40.4900 273.954

Numbers of individuals/records for pairs of traits

1 2

1 "LEA" 2283 842

2 "G:F" 842 955

Covariables

1"LEA" nrec mean sdev min. max.

1 "idv(2)" 2283 6.11082 2.92852 2.00000 17.0000

2 "idade(1)" 2283 448.951 98.8881 306.000 609.000

2"G:F" nrec mean sdev min. max.

1 "idv(2)" 955 6.15079 3.05304 3.00000 17.0000

2 "idade(1)" 955 369.980 37.3543 267.000 511.000

Fixed effects

1 "LEA" nlev

1 "gc" 65

2 "mn" 4

2 "G:F" nlev

1 "gc" 21

Random effects nlev

1 "animal" 3665 NRM

======== end of file ============================01-04-2014==========10:18====

======= Version 30-08-2013 ======================================= **KM** ====

Program WOMBAT: Estimates of covariance components

==============================================================================

Analysis type : "muv 2"

Data file : "DADOS.dat"

Pedigree file : "ReducedPedFile.dat"

Parameter file : "wombat.par"

No. of traits = 2 LEA G:F

No. of records = 3238 2283 955

No. of parameters = 6

Maximum log L = -8429.463

-1/2 AIC & AICC = -8435.463 -8435.476

-1/2 BIC = -8453.622 "Penalty factor" = 4.026

Parameter estimates with approx. sampling erors

1 CHOL Z 1 1 19.5425 0.728923

2 CHOL Z 1 2 0.264983 0.291350

3 CHOL Z 2 2 4.43236 0.176352

4 CHOL A 1 1 8.29540 1.69762

5 CHOL A 1 2 0.554573E-01 0.804002

6 CHOL A 2 2 4.12418 0.279604

Convergence criteria for last 3 iterates

Change in log likelihood = 7.355563 0.078991 0.000132

Change in parameter vector = 0.086071 0.011661 0.000557

Norm of gradient vector = 8.8808 0.7970 0.0334

Newton decrement = -14.0590 -0.1565 -0.0002

***** Estimates of residual covariances ************************************

Order of fit = 2

Covariance matrix

1 19.716

2 5.1784 381.91

Eigenvalues of covariance matrix

Value 381.98 19.64

(%) 95.11 4.89

Trace 401.62

Matrix of correlations and variance ratios

1 0.5368

2 0.0597 0.8473

Covariances & correlations with approximate sampling errors

1 COVS Z 1 1 19.7161 1.55867 vrat 0.537 0.051

2 COVS Z 1 2 5.17843 5.69153 corr 0.060 0.066

3 COVS Z 2 2 381.908 28.4899 vrat 0.847 0.060

***** Estimates for RE 1 "animal" ***************************************

No. of levels = 3665

Covariance structure = NRM

Order of fit = 2

Covariance matrix

1 17.012

2 0.46004 68.814

Eigenvalues of covariance matrix

Value 68.82 17.01

(%) 80.18 19.82

Trace 85.83

Matrix of correlations and variance ratios

1 0.4632

2 0.0134 0.1527

Covariances & correlations with approximate sampling errors

4 COVS A 1 1 17.0119 2.30553 vrat 0.463 0.051

5 COVS A 1 2 0.460040 6.66443 corr 0.013 0.195

6 COVS A 2 2 68.8136 28.1649 vrat 0.153 0.060

***** Estimates of phenotypic covariances ***********************************

Covariance matrix

1 36.728

2 5.6385 450.72

Eigenvalues of covariance matrix

Value 450.80 36.65

(%) 92.48 7.52

Trace 487.45

Correlation matrix

1 1.0000

2 0.0438 1.0000

Covariances & correlations with approximate sampling errors

7 COVS T 1 1 36.7280 1.36755

8 COVS T 1 2 5.63847 4.85081 corr 0.044 0.038

9 COVS T 2 2 450.722 21.6443

======== end of file ============================01-04-2014==========10:18====

======= Version 30-08-2013 ======================================= **KM** ====

Program WOMBAT: Summary of Pedigree Information

==============================================================================

Two traits analysis (BF x GAIN:FEED)

Analysis type : "muv 2"

Data file : "DADOS.dat"

Pedigree file : "ReducedPedFile.dat"

Parameter file : "wombat.par"

No. of animal IDs in data file = = 2396

No. of animal IDs in total = = 3756

*****Pedigree Structure for random effect : 1 ****************************

Original no. of animals = 3756

No. of animals after pruning = 3665

... proportion (%) remaining = 97.6

No. of levels w/out records = 1269

No. of levels with records = 2396 100.0%

... 1 record(s) = 1552 64.8%

... 2 record(s) = 844 35.2%

No. of animals w/out offspring = 1913 52.2%

No. of animals with offspring = 1752 47.8%

... and records = 483 13.2%

No. of animals with unknown sire = 64

No. of animals with unknown dam = 161

No. of animals with both parents unknown = 64

No. of animals with records =

... and unknown sire = 0

... and unknown dam = 0

... and both parents unknown = 0

No. of sires = 313

... with progeny in the data = 180

... with records & progeny in data = 85

No. of dams = 1439

... with progeny in the data = 1042

... with records & progeny in data = 392

No. of animals with known/unpruned grand-parents

... with paternal grandsire = 3520

... with paternal granddam = 3382

... with maternal grandsire = 3391

... with maternal granddam = 3267

Inbreeding coefficients for random effect 1 computed

No. of inbred animals = 3134

Average inbreeding coefficient = 2.3793 (in %)

... amongst inbred animals = 2.7824 (in %)

random effect no. = 1 NRM

no. of elements in NRM/GIN inverse 14038

log determinant = -2522.5521618264124

======== end of file ============================01-04-2014==========10:22====

======= Version 30-08-2013 ======================================= **KM** ====

Program WOMBAT: Summary of information from Set-up step

==============================================================================

Analysis type : "muv 2"

Data file : "DADOS.dat"

Pedigree file : "ReducedPedFile.dat"

Parameter file : "wombat.par"

No. of traits = 2

nrec mean sdev min. max.

1 "BF" 2285 1.75922 1.42798 0.00000 10.5000

2 "G:F" 955 148.845 32.9032 40.4900 273.954

Numbers of individuals/records for pairs of traits

1 2

1 "BF" 2285 844

2 "G:F" 844 955

Covariables

1"BF" nrec mean sdev min. max.

1 "idv(2)" 2285 6.10810 2.92868 2.00000 17.0000

2 "idade(1)" 2285 449.081 98.9421 306.000 609.000

2"G:F" nrec mean sdev min. max.

1 "idv(2)" 955 6.15079 3.05304 3.00000 17.0000

2 "idade(1)" 955 369.980 37.3543 267.000 511.000

Fixed effects

1 "BF" nlev

1 "gc" 65

2 "mn" 4

2 "G:F" nlev

1 "gc" 21

Random effects nlev

1 "animal" 3665 NRM

======== end of file ============================01-04-2014==========10:22====

======= Version 30-08-2013 ======================================= **KM** ====

Program WOMBAT: Estimates of covariance components

==============================================================================

Analysis type : "muv 2"

Data file : "DADOS.dat"

Pedigree file : "ReducedPedFile.dat"

Parameter file : "wombat.par"

No. of traits = 2 BF G:F

No. of records = 3240 2285 955

No. of parameters = 6

Maximum log L = -4422.009

-1/2 AIC & AICC = -4428.009 -4428.022

-1/2 BIC = -4446.170 "Penalty factor" = 4.027

Parameter estimates with approx. sampling erors

1 CHOL Z 1 1 19.6413 0.720907

2 CHOL Z 1 2 0.267558E-01 0.438962E-01

3 CHOL Z 2 2 0.798674 0.235867E-01

4 CHOL A 1 1 2.07822 0.214497

5 CHOL A 1 2 -0.117997 0.110002

6 CHOL A 2 2 -0.655755 0.984442E-01

Convergence criteria for last 3 iterates

Change in log likelihood = 7.104559 0.068296 0.000048

Change in parameter vector = 0.093976 0.011556 0.000081

Norm of gradient vector = 33.7532 2.9880 0.2795

Newton decrement = -13.5682 -0.1359 -0.0001

***** Estimates of residual covariances ************************************

Order of fit = 2

Covariance matrix

1 0.63860

2 0.52552 385.78

Eigenvalues of covariance matrix

Value 385.78 0.64

(%) 99.83 0.17

Trace 386.42

Matrix of correlations and variance ratios

1 0.6927

2 0.0335 0.8580

Covariances & correlations with approximate sampling errors

1 COVS Z 1 1 0.638596 0.376023E-01 vrat 0.693 0.046

2 COVS Z 1 2 0.525519 0.862255 corr 0.033 0.055

3 COVS Z 2 2 385.781 28.3191 vrat 0.858 0.059

***** Estimates for RE 1 "animal" ***************************************

No. of levels = 3665

Covariance structure = NRM

Order of fit = 2

Covariance matrix

1 0.28334

2 -0.94283 63.844

Eigenvalues of covariance matrix

Value 63.86 0.27

(%) 99.58 0.42

Trace 64.13

Matrix of correlations and variance ratios

1 0.3073

2 -0.2217 0.1420

Covariances & correlations with approximate sampling errors

4 COVS A 1 1 0.283337 0.478000E-01 vrat 0.307 0.046

5 COVS A 1 2 -0.942830 0.863680 corr -0.222 0.206

6 COVS A 2 2 63.8444 27.3889 vrat 0.142 0.059

***** Estimates of phenotypic covariances ***********************************

Covariance matrix

1 0.92193

2 -0.41731 449.63

Eigenvalues of covariance matrix

Value 449.63 0.92

(%) 99.80 0.20

Trace 450.55

Correlation matrix

1 1.0000

2 -0.0205 1.0000

Covariances & correlations with approximate sampling errors

7 COVS T 1 1 0.921933 0.313671E-01

8 COVS T 1 2 -0.417312 0.716889 corr -0.020 0.035

9 COVS T 2 2 449.625 21.4927

======== end of file ============================01-04-2014==========10:22====

======= Version 30-08-2013 ======================================= **KM** ====

Program WOMBAT: Summary of Pedigree Information

==============================================================================

Two traits analysis (RFI x GAIN:FEED)

Analysis type : "muv 2"

Data file : "DADOS.dat"

Pedigree file : "ReducedPedFile.dat"

Parameter file : "wombat.par"

No. of animal IDs in data file = = 1930

No. of animal IDs in total = = 3174

*****Pedigree Structure for random effect : 1 ****************************

Original no. of animals = 3174

No. of animals after pruning = 3089

... proportion (%) remaining = 97.3

No. of levels w/out records = 1159

No. of levels with records = 1930 100.0%

... 1 record(s) = 1088 56.4%

... 2 record(s) = 842 43.6%

No. of animals w/out offspring = 1497 48.5%

No. of animals with offspring = 1592 51.5%

... and records = 433 14.0%

No. of animals with unknown sire = 60

No. of animals with unknown dam = 147

No. of animals with both parents unknown = 59

No. of animals with records =

... and unknown sire = 0

... and unknown dam = 0

... and both parents unknown = 0

No. of sires = 307

... with progeny in the data = 114

... with records & progeny in data = 44

No. of dams = 1285

... with progeny in the data = 792

... with records & progeny in data = 389

No. of animals with known/unpruned grand-parents

... with paternal grandsire = 2965

... with paternal granddam = 2839

... with maternal grandsire = 2840

... with maternal granddam = 2751

Inbreeding coefficients for random effect 1 computed

No. of inbred animals = 2643

Average inbreeding coefficient = 2.5643 (in %)

... amongst inbred animals = 2.9971 (in %)

random effect no. = 1 NRM

no. of elements in NRM/GIN inverse 11801

log determinant = -2124.3928089367182

======== end of file ============================01-04-2014==========10:27====

======= Version 30-08-2013 ======================================= **KM** ====

Program WOMBAT: Summary of information from Set-up step

==============================================================================

Analysis type : "muv 2"

Data file : "DADOS.dat"

Pedigree file : "ReducedPedFile.dat"

Parameter file : "wombat.par"

No. of traits = 2

nrec mean sdev min. max.

1 "RF" 1817 5.08663 2.54341 0.00000 19.2000

2 "G:F" 955 148.845 32.9032 40.4900 273.954

Numbers of individuals/records for pairs of traits

1 2

1 "RF" 1817 842

2 "G:F" 842 955

Covariables

1"RF" nrec mean sdev min. max.

1 "idv(2)" 1817 6.09191 3.03138 2.00000 17.0000

2 "idade(1)" 1817 469.100 100.703 306.000 609.000

2"G:F" nrec mean sdev min. max.

1 "idv(2)" 955 6.15079 3.05304 3.00000 17.0000

2 "idade(1)" 955 369.980 37.3543 267.000 511.000

Fixed effects

1 "RF" nlev

1 "gc" 51

2 "mn" 3

2 "G:F" nlev

1 "gc" 21

Random effects nlev

1 "animal" 3089 NRM

======== end of file ============================01-04-2014==========10:27====

======= Version 30-08-2013 ======================================= **KM** ====

Program WOMBAT: Estimates of covariance components

==============================================================================

Analysis type : "muv 2"

Data file : "DADOS.dat"

Pedigree file : "ReducedPedFile.dat"

Parameter file : "wombat.par"

No. of traits = 2 RF G:F

No. of records = 2772 1817 955

No. of parameters = 6

Maximum log L = -5093.479

-1/2 AIC & AICC = -5099.479 -5099.494

-1/2 BIC = -5117.173 "Penalty factor" = 3.949

Parameter estimates with approx. sampling erors

1 CHOL Z 1 1 19.6522 0.723206

2 CHOL Z 1 2 0.119361 0.747328E-01

3 CHOL Z 2 2 1.26071 0.467846E-01

4 CHOL A 1 1 7.95583 1.72842

5 CHOL A 1 2 -0.235776 0.203434

6 CHOL A 2 2 0.971002 0.939485E-01

Convergence criteria for last 3 iterates

Change in log likelihood = 7.530087 0.086949 0.000095

Change in parameter vector = 0.093385 0.014842 0.000473

Norm of gradient vector = 17.4630 2.1911 0.2516

Newton decrement = -14.3648 -0.1718 -0.0002

***** Estimates of residual covariances ************************************

Order of fit = 2

Covariance matrix

1 1.6036

2 2.3457 386.21

Eigenvalues of covariance matrix

Value 386.23 1.59

(%) 99.59 0.41

Trace 387.81

Matrix of correlations and variance ratios

1 0.6163

2 0.0943 0.8592

Covariances & correlations with approximate sampling errors

1 COVS Z 1 1 1.60364 0.116900 vrat 0.616 0.053

2 COVS Z 1 2 2.34572 1.47031 corr 0.094 0.059

3 COVS Z 2 2 386.211 28.4252 vrat 0.859 0.059

***** Estimates for RE 1 "animal" ***************************************

No. of levels = 3089

Covariance structure = NRM

Order of fit = 2

Covariance matrix

1 0.99844

2 -1.8758 63.295

Eigenvalues of covariance matrix

Value 63.35 0.94

(%) 98.53 1.47

Trace 64.29

Matrix of correlations and variance ratios

1 0.3837

2 -0.2360 0.1408

Covariances & correlations with approximate sampling errors

4 COVS A 1 1 0.998436 0.160959 vrat 0.384 0.053

5 COVS A 1 2 -1.87580 1.57729 corr -0.236 0.203

6 COVS A 2 2 63.2952 27.5020 vrat 0.141 0.059

***** Estimates of phenotypic covariances ***********************************

Covariance matrix

1 2.6021

2 0.46993 449.51

Eigenvalues of covariance matrix

Value 449.51 2.60

(%) 99.42 0.58

Trace 452.11

Correlation matrix

1 1.0000

2 0.0137 1.0000

Covariances & correlations with approximate sampling errors

7 COVS T 1 1 2.60207 0.102850

8 COVS T 1 2 0.469926 1.23806 corr 0.014 0.036

9 COVS T 2 2 449.506 21.4859

======== end of file ============================01-04-2014==========10:27====

======= Version 30-08-2013 ======================================= **KM** ====

Program WOMBAT: Summary of Pedigree Information

==============================================================================

Two traits analysis (RFIb x DMI)

Analysis type : "muv 2"

Data file : "DADOS.dat"

Pedigree file : "ReducedPedFile.dat"

Parameter file : "wombat.par"

No. of animal IDs in data file = = 955

No. of animal IDs in total = = 2288

*****Pedigree Structure for random effect : 1 ****************************

Original no. of animals = 2288

No. of animals after pruning = 2204

... proportion (%) remaining = 96.3

No. of levels w/out records = 1249

No. of levels with records = 955 100.0%

... 1 record(s) = 61 6.4%

... 2 record(s) = 894 93.6%

No. of animals w/out offspring = 828 37.6%

No. of animals with offspring = 1376 62.4%

... and records = 127 5.8%

No. of animals with unknown sire = 59

No. of animals with unknown dam = 145

No. of animals with both parents unknown = 58

No. of animals with records =

... and unknown sire = 0

... and unknown dam = 0

... and both parents unknown = 0

No. of sires = 296

... with progeny in the data = 78

... with records & progeny in data = 18

No. of dams = 1080

... with progeny in the data = 542

... with records & progeny in data = 98

No. of animals with known/unpruned grand-parents

... with paternal grandsire = 2083

... with paternal granddam = 1957

... with maternal grandsire = 1959

... with maternal granddam = 1872

random effect no. = 1 NRM

no. of elements in NRM/GIN inverse 8375

log determinant = -1487.8684515369519

======== end of file ============================01-04-2014==========17:44====

======= Version 30-08-2013 ======================================= **KM** ====

Program WOMBAT: Summary of information from Set-up step

==============================================================================

Analysis type : "muv 2"

Data file : "DADOS.dat"

Pedigree file : "ReducedPedFile.dat"

Parameter file : "wombat.par"

No. of traits = 2

nrec mean sdev min. max.

1 "RFIb" 894 -0.916555E-02 0.602128 -2.36330 4.96970

2 "DMI" 955 6.83104 1.33500 2.15500 12.6400

Numbers of individuals/records for pairs of traits

1 2

1 "RFIb" 894 894

2 "DMI" 894 955

Covariables

1"RFIb" nrec mean sdev min. max.

1 "idv(2)" 894 6.16443 3.04994 3.00000 17.0000

2 "idade(1)" 894 368.964 35.9309 267.000 511.000

2"DMI" nrec mean sdev min. max.

1 "idv(2)" 955 6.15079 3.05304 3.00000 17.0000

2 "idade(1)" 955 369.980 37.3543 267.000 511.000

Fixed effects

1 "RFIb" nlev

1 "gc" 21

2 "DMI" nlev

1 "gc" 21

Random effects nlev

1 "animal" 2204 NRM

======== end of file ============================01-04-2014==========17:44====

======= Version 30-08-2013 ======================================= **KM** ====

Program WOMBAT: Estimates of covariance components

==============================================================================

Analysis type : "muv 2"

Data file : "DADOS.dat"

Pedigree file : "ReducedPedFile.dat"

Parameter file : "wombat.par"

No. of traits = 2 RFIb DMI

No. of records = 1849 894 955

No. of parameters = 6

Maximum log L = -71.917

-1/2 AIC & AICC = -77.917 -77.940

-1/2 BIC = -94.406 "Penalty factor" = 3.748

Parameter estimates with approx. sampling erors

1 CHOL Z 1 1 -0.437816 0.563082E-01

2 CHOL Z 1 2 0.421192 0.281855E-01

3 CHOL Z 2 2 -1.02973 0.508638E-01

4 CHOL A 1 1 -0.505157 0.874991E-01

5 CHOL A 1 2 0.175930 0.409439E-01

6 CHOL A 2 2 -1.81369 0.244979

Convergence criteria for last 3 iterates

Change in log likelihood = 5.754050 0.023999 0.000239

Change in parameter vector = 0.046382 0.005476 0.001043

Norm of gradient vector = 151.3669 6.6668 0.5437

Newton decrement = -11.0804 -0.0503 -0.0007

***** Estimates of residual covariances ************************************

Order of fit = 2

Covariance matrix

1 0.30493

2 0.27186 0.41660

Eigenvalues of covariance matrix

Value 0.64 0.08

(%) 88.46 11.54

Trace 0.72

Matrix of correlations and variance ratios

1 0.8413

2 0.7628 0.5336

Covariances & correlations with approximate sampling errors

1 COVS Z 1 1 0.304925 0.231574E-01 vrat 0.841 0.060

2 COVS Z 1 2 0.271856 0.278507E-01 corr 0.763 0.031

3 COVS Z 2 2 0.416599 0.469158E-01 vrat 0.534 0.068

***** Estimates for RE 1 "animal" ***************************************

No. of levels = 2204

Covariance structure = NRM

Order of fit = 2

Covariance matrix

1 0.57537E-01

2 0.10616 0.36410

Eigenvalues of covariance matrix

Value 0.40 0.02

(%) 94.22 5.78

Trace 0.42

Matrix of correlations and variance ratios

1 0.1587

2 0.7334 0.4664

Covariances & correlations with approximate sampling errors

4 COVS A 1 1 0.575370E-01 0.224139E-01 vrat 0.159 0.060

5 COVS A 1 2 0.106158 0.313603E-01 corr 0.733 0.094

6 COVS A 2 2 0.364105 0.637177E-01 vrat 0.466 0.068

***** Estimates of phenotypic covariances ***********************************

Covariance matrix

1 0.36246

2 0.37801 0.78070

Eigenvalues of covariance matrix

Value 1.00 0.14

(%) 87.79 12.21

Trace 1.14

Correlation matrix

1 1.0000

2 0.7106 1.0000

Covariances & correlations with approximate sampling errors

7 COVS T 1 1 0.362462 0.176919E-01

8 COVS T 1 2 0.378014 0.226580E-01 corr 0.711 0.017

9 COVS T 2 2 0.780703 0.407046E-01

======== end of file ============================01-04-2014==========17:44====

======= Version 30-08-2013 ======================================= **KM** ====

Program WOMBAT: Summary of Pedigree Information

==============================================================================

Two traits analysis (RFIb x ADG)

Analysis type : "muv 2"

Data file : "DADOS.dat"

Pedigree file : "ReducedPedFile.dat"

Parameter file : "wombat.par"

No. of animal IDs in data file = = 955

No. of animal IDs in total = = 2288

*****Pedigree Structure for random effect : 1 ****************************

Original no. of animals = 2288

No. of animals after pruning = 2204

... proportion (%) remaining = 96.3

No. of levels w/out records = 1249

No. of levels with records = 955 100.0%

... 1 record(s) = 61 6.4%

... 2 record(s) = 894 93.6%

No. of animals w/out offspring = 828 37.6%

No. of animals with offspring = 1376 62.4%

... and records = 127 5.8%

No. of animals with unknown sire = 59

No. of animals with unknown dam = 145

No. of animals with both parents unknown = 58

No. of animals with records =

... and unknown sire = 0

... and unknown dam = 0

... and both parents unknown = 0

No. of sires = 296

... with progeny in the data = 78

... with records & progeny in data = 18

No. of dams = 1080

... with progeny in the data = 542

... with records & progeny in data = 98

No. of animals with known/unpruned grand-parents

... with paternal grandsire = 2083

... with paternal granddam = 1957

... with maternal grandsire = 1959

... with maternal granddam = 1872

random effect no. = 1 NRM

no. of elements in NRM/GIN inverse 8375

log determinant = -1487.8684515369519

======== end of file ============================01-04-2014==========17:46====

======= Version 30-08-2013 ======================================= **KM** ====

Program WOMBAT: Summary of information from Set-up step

==============================================================================

Analysis type : "muv 2"

Data file : "DADOS.dat"

Pedigree file : "ReducedPedFile.dat"

Parameter file : "wombat.par"

No. of traits = 2

nrec mean sdev min. max.

1 "RFIb" 894 -0.916555E-02 0.602128 -2.36330 4.96970

2 "ADG" 955 1.00846 0.260202 0.176000 1.71800

Numbers of individuals/records for pairs of traits

1 2

1 "RFIb" 894 894

2 "ADG" 894 955

Covariables

1"RFIb" nrec mean sdev min. max.

1 "idv(2)" 894 6.16443 3.04994 3.00000 17.0000

2 "idade(1)" 894 368.964 35.9309 267.000 511.000

2"ADG" nrec mean sdev min. max.

1 "idv(2)" 955 6.15079 3.05304 3.00000 17.0000

2 "idade(1)" 955 369.980 37.3543 267.000 511.000

Fixed effects

1 "RFIb" nlev

1 "gc" 21

2 "ADG" nlev

1 "gc" 21

Random effects nlev

1 "animal" 2204 NRM

======== end of file ============================01-04-2014==========17:46====

======= Version 30-08-2013 ======================================= **KM** ====

Program WOMBAT: Estimates of covariance components

==============================================================================

Analysis type : "muv 2"

Data file : "DADOS.dat"

Pedigree file : "ReducedPedFile.dat"

Parameter file : "wombat.par"

No. of traits = 2 RFIb ADG

No. of records = 1849 894 955

No. of parameters = 6

Maximum log L = 1212.518

-1/2 AIC & AICC = 1206.518 1206.495

-1/2 BIC = 1190.029 "Penalty factor" = 3.748

Parameter estimates with approx. sampling erors

1 CHOL Z 1 1 -0.606611 0.394744E-01

2 CHOL Z 1 2 -0.152873E-01 0.758346E-02

3 CHOL Z 2 2 -2.13771 0.544130E-01

4 CHOL A 1 1 -1.34780 0.175462

5 CHOL A 1 2 0.416866E-01 0.184932E-01

6 CHOL A 2 2 -2.36889 0.126975

Convergence criteria for last 3 iterates

Change in log likelihood = 7.767462 0.054597 0.000093

Change in parameter vector = 0.020327 0.003257 0.000630

Norm of gradient vector = 656.1141 33.0493 0.5155

Newton decrement = -14.9318 -0.1101 -0.0003

***** Estimates of residual covariances ************************************

Order of fit = 2

Covariance matrix

1 0.29724

2 -0.83346E-02 0.14140E-01

Eigenvalues of covariance matrix

Value 0.30 0.01

(%) 95.54 4.46

Trace 0.31

Matrix of correlations and variance ratios

1 0.8149

2 -0.1286 0.5740

Covariances & correlations with approximate sampling errors

1 COVS Z 1 1 0.297238 0.234666E-01 vrat 0.815 0.062

2 COVS Z 1 2 -0.833459E-02 0.411884E-02 corr -0.129 0.064

3 COVS Z 2 2 0.141400E-01 0.148352E-02 vrat 0.574 0.067

***** Estimates for RE 1 "animal" ***************************************

No. of levels = 2204

Covariance structure = NRM

Order of fit = 2

Covariance matrix

1 0.67502E-01

2 0.10831E-01 0.10496E-01

Eigenvalues of covariance matrix

Value 0.07 0.01

(%) 89.09 10.91

Trace 0.08

Matrix of correlations and variance ratios

1 0.1851

2 0.4069 0.4260

Covariances & correlations with approximate sampling errors

4 COVS A 1 1 0.675023E-01 0.236882E-01 vrat 0.185 0.062

5 COVS A 1 2 0.108307E-01 0.472584E-02 corr 0.407 0.177

6 COVS A 2 2 0.104958E-01 0.195031E-02 vrat 0.426 0.067

***** Estimates of phenotypic covariances ***********************************

Covariance matrix

1 0.36474

2 0.24961E-02 0.24636E-01

Eigenvalues of covariance matrix

Value 0.36 0.02

(%) 93.68 6.32

Trace 0.39

Correlation matrix

1 1.0000

2 0.0263 1.0000

Covariances & correlations with approximate sampling errors

7 COVS T 1 1 0.364740 0.181180E-01

8 COVS T 1 2 0.249610E-02 0.338127E-02 corr 0.026 0.036

9 COVS T 2 2 0.246358E-01 0.127043E-02

======== end of file ============================01-04-2014==========17:46====

======= Version 30-08-2013 ======================================= **KM** ====

Program WOMBAT: Summary of Pedigree Information

==============================================================================

Two traits analysis (WS x RFIb)

Analysis type : "muv 2"

Data file : "DADOS.dat"

Pedigree file : "ReducedPedFile.dat"

Parameter file : "wombat.par"

No. of animal IDs in data file = = 8090

No. of animal IDs in total = = 8489

*****Pedigree Structure for random effect : 1 ****************************

Original no. of animals = 8489

No. of animals after pruning = 8432

... proportion (%) remaining = 99.3

No. of levels w/out records = 342

No. of levels with records = 8090 100.0%

... 1 record(s) = 7208 89.1%

... 2 record(s) = 882 10.9%

No. of animals w/out offspring = 6051 71.8%

No. of animals with offspring = 2381 28.2%

... and records = 2039 24.2%

No. of animals with unknown sire = 313

No. of animals with unknown dam = 384

No. of animals with both parents unknown = 312

No. of animals with records =

... and unknown sire = 1

... and unknown dam = 53

... and both parents unknown = 0

No. of sires = 325

... with progeny in the data = 320

... with records & progeny in data = 288

No. of dams = 2056

... with progeny in the data = 2056

... with records & progeny in data = 1751

No. of animals with known/unpruned grand-parents

... with paternal grandsire = 7357

... with paternal granddam = 7055

... with maternal grandsire = 6534

... with maternal granddam = 6467

Inbreeding coefficients for random effect 1 computed

No. of inbred animals = 5143

Average inbreeding coefficient = 1.5416 (in %)

... amongst inbred animals = 2.5274 (in %)

random effect no. = 1 NRM

no. of elements in NRM/GIN inverse 31835

log determinant = -5688.0517714250827

random effect no. = 2 IDE

no. of elements in NRM/GIN inverse 0

log determinant = 0.0000000000000000

======== end of file ============================06-02-2015==========11:39====

======= Version 30-08-2013 ======================================= **KM** ====

Program WOMBAT: Summary of information from Set-up step

==============================================================================

Analysis type : "muv 2"

Data file : "DADOS.dat"

Pedigree file : "ReducedPedFile.dat"

Parameter file : "wombat.par"

No. of traits = 2

nrec mean sdev min. max.

1 "WS" 8078 299.703 49.6462 160.030 489.760

2 "RFIb" 894 -0.916555E-02 0.602128 -2.36330 4.96970

Numbers of individuals/records for pairs of traits

1 2

1 "WS" 8078 882

2 "RFIb" 882 894

Covariables

1"WS" nrec mean sdev min. max.

1 "idv(2)" 8078 6.63568 3.07616 2.00000 18.0000

2 "idade(1)" 8078 473.414 105.028 293.000 645.000

2"RFIb" nrec mean sdev min. max.

1 "idv(2)" 894 6.16443 3.04994 3.00000 17.0000

2 "idade(1)" 894 368.964 35.9309 267.000 511.000

Fixed effects

1 "WS" nlev

1 "gc" 201

2 "mn" 4

2 "RFIb" nlev

1 "gc" 21

Random effects nlev

1 "animal" 8432 NRM

2 "peanim" 2107 IDE

======== end of file ============================06-02-2015==========11:39====

======= Version 30-08-2013 ======================================= **KM** ====

Program WOMBAT: Estimates of covariance components

==============================================================================

Analysis type : "muv 2"

Data file : "DADOS.dat"

Pedigree file : "ReducedPedFile.dat"

Parameter file : "wombat.par"

No. of traits = 2 WS RFIb

No. of records = 8972 8078 894

No. of parameters = 7

Maximum log L = -30197.059

-1/2 AIC & AICC = -30204.059 -30204.065

-1/2 BIC = -30228.824 "Penalty factor" = 4.538

Parameter estimates with approx. sampling erors

1 CHOL Z 1 1 3.02595 0.211216E-01

2 CHOL Z 1 2 -0.188896E-02 0.308594E-01

3 CHOL Z 2 2 -0.604166 0.396883E-01

4 CHOL A 1 1 2.88189 0.479794E-01

5 CHOL A 1 2 0.596684E-01 0.394700E-01

6 CHOL A 2 2 -1.38485 0.195655

7 CHOL B 1 1 2.33002 0.506872E-01

Convergence criteria for last 3 iterates

Change in log likelihood = 0.152381 0.003819 0.000246

Change in parameter vector = 0.005257 0.000992 0.000258

Norm of gradient vector = 18.4912 1.5680 0.3984

Newton decrement = -0.2779 -0.0065 -0.0004

***** Estimates of residual covariances ************************************

Order of fit = 2

Covariance matrix

1 424.92

2 -0.38938E-01 0.29870

Eigenvalues of covariance matrix

Value 424.92 0.30

(%) 99.93 0.07

Trace 425.21

Matrix of correlations and variance ratios

1 0.5004

2 -0.0035 0.8185

Covariances & correlations with approximate sampling errors

1 COVS Z 1 1 424.916 17.9498 vrat 0.500 0.027

2 COVS Z 1 2 -0.389381E-01 0.636099 corr -0.003 0.056

3 COVS Z 2 2 0.298699 0.237118E-01 vrat 0.818 0.063

***** Estimates for RE 1 "animal" ***************************************

No. of levels = 8432

Covariance structure = NRM

Order of fit = 2

Covariance matrix

1 318.55

2 1.0650 0.66241E-01

Eigenvalues of covariance matrix

Value 318.55 0.06

(%) 99.98 0.02

Trace 318.62

Matrix of correlations and variance ratios

1 0.3752

2 0.2318 0.1815

Covariances & correlations with approximate sampling errors

4 COVS A 1 1 318.549 30.5676 vrat 0.375 0.030

5 COVS A 1 2 1.06496 0.706559 corr 0.232 0.160

6 COVS A 2 2 0.662410E-01 0.239092E-01 vrat 0.182 0.063

***** Estimates for RE 2 "peanim" ***************************************

No. of levels = 2107

Covariance structure = IDE

Order of fit = 1

Covariance matrix

1 105.64

Matrix of correlations and variance ratios

1 0.1244

Covariances & correlations with approximate sampling errors

7 COVS B 1 1 105.640 10.7092 vrat 0.124 0.012

***** Estimates of phenotypic covariances ***********************************

Covariance matrix

1 849.11

2 1.0260 0.36494

Eigenvalues of covariance matrix

Value 849.11 0.36

(%) 99.96 0.04

Trace 849.47

Correlation matrix

1 1.0000

2 0.0583 1.0000

Covariances & correlations with approximate sampling errors

8 COVS T 1 1 849.105 18.5571

9 COVS T 1 2 1.02602 0.584174 corr 0.058 0.033

10 COVS T 2 2 0.364940 0.181313E-01

======== end of file ============================06-02-2015==========11:39====

======= Version 30-08-2013 ======================================= **KM** ====

Program WOMBAT: Summary of Pedigree Information

==============================================================================

Two traits analysis (HH x RFIb)

Analysis type : "muv 2"

Data file : "DADOS.dat"

Pedigree file : "ReducedPedFile.dat"

Parameter file : "wombat.par"

No. of animal IDs in data file = = 6559

No. of animal IDs in total = = 7099

*****Pedigree Structure for random effect : 1 ****************************

Original no. of animals = 7099

No. of animals after pruning = 7016

... proportion (%) remaining = 98.8

No. of levels w/out records = 457

No. of levels with records = 6559 100.0%

... 1 record(s) = 5676 86.5%

... 2 record(s) = 883 13.5%

No. of animals w/out offspring = 4828 68.8%

No. of animals with offspring = 2188 31.2%

... and records = 1731 24.7%

No. of animals with unknown sire = 148

No. of animals with unknown dam = 237

No. of animals with both parents unknown = 148

No. of animals with records =

... and unknown sire = 0

... and unknown dam = 18

... and both parents unknown = 0

No. of sires = 320

... with progeny in the data = 283

... with records & progeny in data = 240

No. of dams = 1868

... with progeny in the data = 1837

... with records & progeny in data = 1491

No. of animals with known/unpruned grand-parents

... with paternal grandsire = 6737

... with paternal granddam = 6483

... with maternal grandsire = 6338

... with maternal granddam = 6110

Inbreeding coefficients for random effect 1 computed

No. of inbred animals = 5148

Average inbreeding coefficient = 1.8503 (in %)

... amongst inbred animals = 2.5217 (in %)

random effect no. = 1 NRM

no. of elements in NRM/GIN inverse 26819

log determinant = -4814.0947710995106

======== end of file ============================06-02-2015==========11:41====

======= Version 30-08-2013 ======================================= **KM** ====

Program WOMBAT: Summary of information from Set-up step

==============================================================================

Analysis type : "muv 2"

Data file : "DADOS.dat"

Pedigree file : "ReducedPedFile.dat"

Parameter file : "wombat.par"

No. of traits = 2

nrec mean sdev min. max.

1 "HH" 6548 132.269 5.43028 100.000 149.000

2 "RFIb" 894 -0.916555E-02 0.602128 -2.36330 4.96970

Numbers of individuals/records for pairs of traits

1 2

1 "HH" 6548 883

2 "RFIb" 883 894

Covariables

1"HH" nrec mean sdev min. max.

1 "idv(2)" 6548 6.40043 2.93455 2.00000 17.0000

2 "idade(1)" 6548 472.701 107.333 293.000 645.000

2"RFIb" nrec mean sdev min. max.

1 "idv(2)" 894 6.16443 3.04994 3.00000 17.0000

2 "idade(1)" 894 368.964 35.9309 267.000 511.000

Fixed effects

1 "HH" nlev

1 "gc" 169

2 "mn" 4

2 "RFIb" nlev

1 "gc" 21

Random effects nlev

1 "animal" 7016 NRM

======== end of file ============================06-02-2015==========11:41====

======= Version 30-08-2013 ======================================= **KM** ====

Program WOMBAT: Estimates of covariance components

==============================================================================

Analysis type : "muv 2"

Data file : "DADOS.dat"

Pedigree file : "ReducedPedFile.dat"

Parameter file : "wombat.par"

No. of traits = 2 HH RFIb

No. of records = 7442 6548 894

No. of parameters = 6

Maximum log L = -11448.985

-1/2 AIC & AICC = -11454.985 -11454.990

-1/2 BIC = -11475.648 "Penalty factor" = 4.444

Parameter estimates with approx. sampling erors

1 CHOL Z 1 1 0.897274 0.266285E-01

2 CHOL Z 1 2 0.137757E-01 0.384540E-01

3 CHOL Z 2 2 -0.616149 0.408647E-01

4 CHOL A 1 1 1.11999 0.316279E-01

5 CHOL A 1 2 0.178670E-01 0.401775E-01

6 CHOL A 2 2 -1.29355 0.166368

Convergence criteria for last 3 iterates

Change in log likelihood = 0.091453 0.001495 0.000033

Change in parameter vector = 0.007029 0.000989 0.000147

Norm of gradient vector = 18.1566 1.3869 0.1925

Newton decrement = -0.1653 -0.0026 -0.0001

***** Estimates of residual covariances ************************************

Order of fit = 2

Covariance matrix

1 6.0167

2 0.33790E-01 0.29181

Eigenvalues of covariance matrix

Value 6.02 0.29

(%) 95.38 4.62

Trace 6.31

Matrix of correlations and variance ratios

1 0.3904

2 0.0255 0.7943

Covariances & correlations with approximate sampling errors

1 COVS Z 1 1 6.01675 0.320433 vrat 0.390 0.026

2 COVS Z 1 2 0.337904E-01 0.943296E-01 corr 0.026 0.071

3 COVS Z 2 2 0.291811 0.237690E-01 vrat 0.794 0.064

***** Estimates for RE 1 "animal" ***************************************

No. of levels = 7016

Covariance structure = NRM

Order of fit = 2

Covariance matrix

1 9.3932

2 0.54759E-01 0.75557E-01

Eigenvalues of covariance matrix

Value 9.39 0.08

(%) 99.21 0.79

Trace 9.47

Matrix of correlations and variance ratios

1 0.6096

2 0.0650 0.2057

Covariances & correlations with approximate sampling errors

4 COVS A 1 1 9.39320 0.594174 vrat 0.610 0.026

5 COVS A 1 2 0.547594E-01 0.123170 corr 0.065 0.148

6 COVS A 2 2 0.755572E-01 0.248545E-01 vrat 0.206 0.064

***** Estimates of phenotypic covariances ***********************************

Covariance matrix

1 15.410

2 0.88550E-01 0.36737

Eigenvalues of covariance matrix

Value 15.41 0.37

(%) 97.67 2.33

Trace 15.78

Correlation matrix

1 1.0000

2 0.0372 1.0000

Covariances & correlations with approximate sampling errors

7 COVS T 1 1 15.4099 0.382701

8 COVS T 1 2 0.885498E-01 0.895203E-01 corr 0.037 0.038

9 COVS T 2 2 0.367368 0.184158E-01

======== end of file ============================06-02-2015==========11:41====

======= Version 30-08-2013 ======================================= **KM** ====

Program WOMBAT: Summary of Pedigree Information

==============================================================================

Two traits analysis (CC x RFIb)

Analysis type : "muv 2"

Data file : "DADOS.dat"

Pedigree file : "ReducedPedFile.dat"

Parameter file : "wombat.par"

No. of animal IDs in data file = = 3941

No. of animal IDs in total = = 5416

*****Pedigree Structure for random effect : 1 ****************************

Original no. of animals = 5416

No. of animals after pruning = 5328

... proportion (%) remaining = 98.4

No. of levels w/out records = 1387

No. of levels with records = 3941 100.0%

... 1 record(s) = 3112 79.0%

... 2 record(s) = 829 21.0%

No. of animals w/out offspring = 3309 62.1%

No. of animals with offspring = 2019 37.9%

... and records = 632 11.9%

No. of animals with unknown sire = 94

No. of animals with unknown dam = 188

No. of animals with both parents unknown = 94

No. of animals with records =

... and unknown sire = 0

... and unknown dam = 0

... and both parents unknown = 0

No. of sires = 318

... with progeny in the data = 255

... with records & progeny in data = 217

No. of dams = 1701

... with progeny in the data = 1508

... with records & progeny in data = 412

No. of animals with known/unpruned grand-parents

... with paternal grandsire = 5140

... with paternal granddam = 4952

... with maternal grandsire = 4951

... with maternal granddam = 4736

Inbreeding coefficients for random effect 1 computed

No. of inbred animals = 4322

Average inbreeding coefficient = 2.1289 (in %)

... amongst inbred animals = 2.6244 (in %)

random effect no. = 1 NRM

no. of elements in NRM/GIN inverse 20457

log determinant = -3670.4017279806212

======== end of file ============================06-02-2015==========11:43====

======= Version 30-08-2013 ======================================= **KM** ====

Program WOMBAT: Summary of information from Set-up step

==============================================================================

Analysis type : "muv 2"

Data file : "DADOS.dat"

Pedigree file : "ReducedPedFile.dat"

Parameter file : "wombat.par"

No. of traits = 2

nrec mean sdev min. max.

1 "CC" 3876 164.140 8.72028 128.000 192.000

2 "RFIb" 894 -0.916555E-02 0.602128 -2.36330 4.96970

Numbers of individuals/records for pairs of traits

1 2

1 "CC" 3876 829

2 "RFIb" 829 894

Covariables

1"CC" nrec mean sdev min. max.

1 "idv(2)" 3876 6.27632 2.87660 2.00000 17.0000

2 "idade(1)" 3876 431.403 109.187 293.000 725.000

2"RFIb" nrec mean sdev min. max.

1 "idv(2)" 894 6.16443 3.04994 3.00000 17.0000

2 "idade(1)" 894 368.964 35.9309 267.000 511.000

Fixed effects

1 "CC" nlev

1 "gc" 100

2 "mn" 4

2 "RFIb" nlev

1 "gc" 21

Random effects nlev

1 "animal" 5328 NRM

======== end of file ============================06-02-2015==========11:43====

======= Version 30-08-2013 ======================================= **KM** ====

Program WOMBAT: Estimates of covariance components

==============================================================================

Analysis type : "muv 2"

Data file : "DADOS.dat"

Pedigree file : "ReducedPedFile.dat"

Parameter file : "wombat.par"

No. of traits = 2 CC RFIb

No. of records = 4770 3876 894

No. of parameters = 6

Maximum log L = -8741.284

-1/2 AIC & AICC = -8747.284 -8747.293

-1/2 BIC = -8766.611 "Penalty factor" = 4.221

Parameter estimates with approx. sampling erors

1 CHOL Z 1 1 1.61727 0.234050E-01

2 CHOL Z 1 2 0.836122E-02 0.309310E-01

3 CHOL Z 2 2 -0.613934 0.403626E-01

4 CHOL A 1 1 1.18567 0.728018E-01

5 CHOL A 1 2 0.382209E-01 0.508945E-01

6 CHOL A 2 2 -1.31139 0.171574

Convergence criteria for last 3 iterates

Change in log likelihood = 1.713837 0.019330 0.000493

Change in parameter vector = 0.036001 0.005085 0.000875

Norm of gradient vector = 93.7877 5.9429 0.3923

Newton decrement = -3.3107 -0.0342 -0.0008

***** Estimates of residual covariances ************************************

Order of fit = 2

Covariance matrix

1 25.395

2 0.42135E-01 0.29299

Eigenvalues of covariance matrix

Value 25.39 0.29

(%) 98.86 1.14

Trace 25.69

Matrix of correlations and variance ratios

1 0.7033

2 0.0154 0.7982

Covariances & correlations with approximate sampling errors

1 COVS Z 1 1 25.3948 1.18873 vrat 0.703 0.039

2 COVS Z 1 2 0.421349E-01 0.155895 corr 0.015 0.057

3 COVS Z 2 2 0.292986 0.236395E-01 vrat 0.798 0.063

***** Estimates for RE 1 "animal" ***************************************

No. of levels = 5328

Covariance structure = NRM

Order of fit = 2

Covariance matrix

1 10.712

2 0.12509 0.74061E-01

Eigenvalues of covariance matrix

Value 10.71 0.07

(%) 99.33 0.67

Trace 10.79

Matrix of correlations and variance ratios

1 0.2967

2 0.1404 0.2018

Covariances & correlations with approximate sampling errors

4 COVS A 1 1 10.7117 1.55967 vrat 0.297 0.039

5 COVS A 1 2 0.125092 0.166893 corr 0.140 0.189

6 COVS A 2 2 0.740612E-01 0.244932E-01 vrat 0.202 0.063

***** Estimates of phenotypic covariances ***********************************

Covariance matrix

1 36.107

2 0.16723 0.36705

Eigenvalues of covariance matrix

Value 36.11 0.37

(%) 99.00 1.00

Trace 36.47

Correlation matrix

1 1.0000

2 0.0459 1.0000

Covariances & correlations with approximate sampling errors

7 COVS T 1 1 36.1066 0.973036

8 COVS T 1 2 0.167227 0.134230 corr 0.046 0.037

9 COVS T 2 2 0.367047 0.183570E-01

======== end of file ============================06-02-2015==========11:43====

======= Version 30-08-2013 ======================================= **KM** ====

Program WOMBAT: Summary of Pedigree Information

==============================================================================

Two traits analysis (LEA x RFIb)

Analysis type : "muv 2"

Data file : "DADOS.dat"

Pedigree file : "ReducedPedFile.dat"

Parameter file : "wombat.par"

No. of animal IDs in data file = = 2350

No. of animal IDs in total = = 3729

*****Pedigree Structure for random effect : 1 ****************************

Original no. of animals = 3729

No. of animals after pruning = 3638

... proportion (%) remaining = 97.6

No. of levels w/out records = 1288

No. of levels with records = 2350 100.0%

... 1 record(s) = 1523 64.8%

... 2 record(s) = 827 35.2%

No. of animals w/out offspring = 1889 51.9%

No. of animals with offspring = 1749 48.1%

... and records = 461 12.7%

No. of animals with unknown sire = 64

No. of animals with unknown dam = 161

No. of animals with both parents unknown = 64

No. of animals with records =

... and unknown sire = 0

... and unknown dam = 0

... and both parents unknown = 0

No. of sires = 313

... with progeny in the data = 180

... with records & progeny in data = 84

No. of dams = 1436

... with progeny in the data = 1036

... with records & progeny in data = 371

No. of animals with known/unpruned grand-parents

... with paternal grandsire = 3493

... with paternal granddam = 3355

... with maternal grandsire = 3364

... with maternal granddam = 3240

Inbreeding coefficients for random effect 1 computed

No. of inbred animals = 3107

Average inbreeding coefficient = 2.3747 (in %)

... amongst inbred animals = 2.7805 (in %)

random effect no. = 1 NRM

no. of elements in NRM/GIN inverse 13937

log determinant = -2503.1878537727198

======== end of file ============================06-02-2015==========11:46====

======= Version 30-08-2013 ======================================= **KM** ====

Program WOMBAT: Summary of information from Set-up step

==============================================================================

Analysis type : "muv 2"

Data file : "DADOS.dat"

Pedigree file : "ReducedPedFile.dat"

Parameter file : "wombat.par"

No. of traits = 2

nrec mean sdev min. max.

1 "LEA" 2283 51.4305 8.89390 21.4000 83.4000

2 "RFIb" 894 -0.916555E-02 0.602128 -2.36330 4.96970

Numbers of individuals/records for pairs of traits

1 2

1 "LEA" 2283 827

2 "RFIb" 827 894

Covariables

1"LEA" nrec mean sdev min. max.

1 "idv(2)" 2283 6.11082 2.92852 2.00000 17.0000

2 "idade(1)" 2283 448.951 98.8881 306.000 609.000

2"RFIb" nrec mean sdev min. max.

1 "idv(2)" 894 6.16443 3.04994 3.00000 17.0000

2 "idade(1)" 894 368.964 35.9309 267.000 511.000

Fixed effects

1 "LEA" nlev

1 "gc" 65

2 "mn" 4

2 "RFIb" nlev

1 "gc" 21

Random effects nlev

1 "animal" 3638 NRM

======== end of file ============================06-02-2015==========11:46====

======= Version 30-08-2013 ======================================= **KM** ====

Program WOMBAT: Estimates of covariance components

==============================================================================

Analysis type : "muv 2"

Data file : "DADOS.dat"

Pedigree file : "ReducedPedFile.dat"

Parameter file : "wombat.par"

No. of traits = 2 LEA RFIb

No. of records = 3177 2283 894

No. of parameters = 6

Maximum log L = -5114.287

-1/2 AIC & AICC = -5120.287 -5120.300

-1/2 BIC = -5138.387 "Penalty factor" = 4.017

Parameter estimates with approx. sampling erors

1 CHOL Z 1 1 1.49188 0.394289E-01

2 CHOL Z 1 2 0.256496E-01 0.369462E-01

3 CHOL Z 2 2 -0.620251 0.408290E-01

4 CHOL A 1 1 1.41481 0.679381E-01

5 CHOL A 1 2 -0.169358E-01 0.493525E-01

6 CHOL A 2 2 -1.27725 0.159585

Convergence criteria for last 3 iterates

Change in log likelihood = 0.689362 0.000587 0.000001

Change in parameter vector = 0.014849 0.000772 0.000092

Norm of gradient vector = 41.9927 1.0845 0.0244

Newton decrement = -1.3527 -0.0012 -0.0000

***** Estimates of residual covariances ************************************

Order of fit = 2

Covariance matrix

1 19.762

2 0.11402 0.28990

Eigenvalues of covariance matrix

Value 19.76 0.29

(%) 98.56 1.44

Trace 20.05

Matrix of correlations and variance ratios

1 0.5385

2 0.0476 0.7879

Covariances & correlations with approximate sampling errors

1 COVS Z 1 1 19.7619 1.55838 vrat 0.538 0.051

2 COVS Z 1 2 0.114024 0.164256 corr 0.048 0.069

3 COVS Z 2 2 0.289897 0.235821E-01 vrat 0.788 0.063

***** Estimates for RE 1 "animal" ***************************************

No. of levels = 3638

Covariance structure = NRM

Order of fit = 2

Covariance matrix

1 16.939

2 -0.69703E-01 0.78019E-01

Eigenvalues of covariance matrix

Value 16.94 0.08

(%) 99.54 0.46

Trace 17.02

Matrix of correlations and variance ratios

1 0.4615

2 -0.0606 0.2121

Covariances & correlations with approximate sampling errors

4 COVS A 1 1 16.9391 2.30161 vrat 0.462 0.051

5 COVS A 1 2 -0.697029E-01 0.203063 corr -0.061 0.177

6 COVS A 2 2 0.780186E-01 0.248043E-01 vrat 0.212 0.063

***** Estimates of phenotypic covariances ***********************************

Covariance matrix

1 36.701

2 0.44321E-01 0.36792

Eigenvalues of covariance matrix

Value 36.70 0.37

(%) 99.01 0.99

Trace 37.07

Correlation matrix

1 1.0000

2 0.0121 1.0000

Covariances & correlations with approximate sampling errors

7 COVS T 1 1 36.7010 1.36533

8 COVS T 1 2 0.443209E-01 0.142226 corr 0.012 0.039

9 COVS T 2 2 0.367916 0.184693E-01

======== end of file ============================06-02-2015==========11:46====

======= Version 30-08-2013 ======================================= **KM** ====

Program WOMBAT: Summary of Pedigree Information

==============================================================================

Two traits analysis (BF x RFIb)

Analysis type : "muv 2"

Data file : "DADOS.dat"

Pedigree file : "ReducedPedFile.dat"

Parameter file : "wombat.par"

No. of animal IDs in data file = = 2350

No. of animal IDs in total = = 3729

*****Pedigree Structure for random effect : 1 ****************************

Original no. of animals = 3729

No. of animals after pruning = 3638

... proportion (%) remaining = 97.6

No. of levels w/out records = 1288

No. of levels with records = 2350 100.0%

... 1 record(s) = 1521 64.7%

... 2 record(s) = 829 35.3%

No. of animals w/out offspring = 1889 51.9%

No. of animals with offspring = 1749 48.1%

... and records = 461 12.7%

No. of animals with unknown sire = 64

No. of animals with unknown dam = 161

No. of animals with both parents unknown = 64

No. of animals with records =

... and unknown sire = 0

... and unknown dam = 0

... and both parents unknown = 0

No. of sires = 313

... with progeny in the data = 180

... with records & progeny in data = 84

No. of dams = 1436

... with progeny in the data = 1036

... with records & progeny in data = 371

No. of animals with known/unpruned grand-parents

... with paternal grandsire = 3493

... with paternal granddam = 3355

... with maternal grandsire = 3364

... with maternal granddam = 3240

Inbreeding coefficients for random effect 1 computed

No. of inbred animals = 3107

Average inbreeding coefficient = 2.3747 (in %)

... amongst inbred animals = 2.7805 (in %)

random effect no. = 1 NRM

no. of elements in NRM/GIN inverse 13937

log determinant = -2503.1878537727198

======== end of file ============================06-02-2015==========13:05====

======= Version 30-08-2013 ======================================= **KM** ====

Program WOMBAT: Summary of information from Set-up step

==============================================================================

Analysis type : "muv 2"

Data file : "DADOS.dat"

Pedigree file : "ReducedPedFile.dat"

Parameter file : "wombat.par"

No. of traits = 2

nrec mean sdev min. max.

1 "BF" 2285 1.75922 1.42798 0.00000 10.5000

2 "RFIb" 894 -0.916555E-02 0.602128 -2.36330 4.96970

Numbers of individuals/records for pairs of traits

1 2

1 "BF" 2285 829

2 "RFIb" 829 894

Covariables

1"BF" nrec mean sdev min. max.

1 "idv(2)" 2285 6.10810 2.92868 2.00000 17.0000

2 "idade(1)" 2285 449.081 98.9421 306.000 609.000

2"RFIb" nrec mean sdev min. max.

1 "idv(2)" 894 6.16443 3.04994 3.00000 17.0000

2 "idade(1)" 894 368.964 35.9309 267.000 511.000

Fixed effects

1 "BF" nlev

1 "gc" 65

2 "mn" 4

2 "RFIb" nlev

1 "gc" 21

Random effects nlev

1 "animal" 3638 NRM

======== end of file ============================06-02-2015==========13:05====

======= Version 30-08-2013 ======================================= **KM** ====

Program WOMBAT: Estimates of covariance components

==============================================================================

Analysis type : "muv 2"

Data file : "DADOS.dat"

Pedigree file : "ReducedPedFile.dat"

Parameter file : "wombat.par"

No. of traits = 2 BF RFIb

No. of records = 3179 2285 894

No. of parameters = 6

Maximum log L = -1105.224

-1/2 AIC & AICC = -1111.224 -1111.238

-1/2 BIC = -1129.326 "Penalty factor" = 4.017

Parameter estimates with approx. sampling erors

1 CHOL Z 1 1 -0.221249 0.292146E-01

2 CHOL Z 1 2 -0.359124E-01 0.313281E-01

3 CHOL Z 2 2 -0.612678 0.397316E-01

4 CHOL A 1 1 -0.640335 0.852430E-01

5 CHOL A 1 2 0.899550E-01 0.499921E-01

6 CHOL A 2 2 -1.38047 0.194780

Convergence criteria for last 3 iterates

Change in log likelihood = 12.066875 0.027711 0.000026

Change in parameter vector = 0.096680 0.006766 0.000631

Norm of gradient vector = 264.2186 11.3281 0.2001

Newton decrement = -23.1335 -0.0562 -0.0001

***** Estimates of residual covariances ************************************

Order of fit = 2

Covariance matrix

1 0.64243

2 -0.28784E-01 0.29494

Eigenvalues of covariance matrix

Value 0.64 0.29

(%) 68.79 31.21

Trace 0.94

Matrix of correlations and variance ratios

1 0.6981

2 -0.0661 0.8053

Covariances & correlations with approximate sampling errors

1 COVS Z 1 1 0.642429 0.375366E-01 vrat 0.698 0.046

2 COVS Z 1 2 -0.287844E-01 0.250953E-01 corr -0.066 0.058

3 COVS Z 2 2 0.294943 0.232800E-01 vrat 0.805 0.061

***** Estimates for RE 1 "animal" ***************************************

No. of levels = 3638

Covariance structure = NRM

Order of fit = 2

Covariance matrix

1 0.27785

2 0.47417E-01 0.71324E-01

Eigenvalues of covariance matrix

Value 0.29 0.06

(%) 82.54 17.46

Trace 0.35

Matrix of correlations and variance ratios

1 0.3019

2 0.3368 0.1947

Covariances & correlations with approximate sampling errors

4 COVS A 1 1 0.277851 0.473697E-01 vrat 0.302 0.046

5 COVS A 1 2 0.474167E-01 0.264579E-01 corr 0.337 0.191

6 COVS A 2 2 0.713236E-01 0.236674E-01 vrat 0.195 0.061

***** Estimates of phenotypic covariances ***********************************

Covariance matrix

1 0.92028

2 0.18632E-01 0.36627

Eigenvalues of covariance matrix

Value 0.92 0.37

(%) 71.58 28.42

Trace 1.29

Correlation matrix

1 1.0000

2 0.0321 1.0000

Covariances & correlations with approximate sampling errors

7 COVS T 1 1 0.920280 0.312018E-01

8 COVS T 1 2 0.186323E-01 0.210104E-01 corr 0.032 0.036

9 COVS T 2 2 0.366267 0.182332E-01

======== end of file ============================06-02-2015==========13:05====

======= Version 30-08-2013 ======================================= **KM** ====

Program WOMBAT: Summary of Pedigree Information

==============================================================================

Two traits analysis (RF x RFIb)

Analysis type : "muv 2"

Data file : "DADOS.dat"

Pedigree file : "ReducedPedFile.dat"

Parameter file : "wombat.par"

No. of animal IDs in data file = = 1884

No. of animal IDs in total = = 3147

*****Pedigree Structure for random effect : 1 ****************************

Original no. of animals = 3147

No. of animals after pruning = 3062

... proportion (%) remaining = 97.3

No. of levels w/out records = 1178

No. of levels with records = 1884 100.0%

... 1 record(s) = 1057 56.1%

... 2 record(s) = 827 43.9%

No. of animals w/out offspring = 1473 48.1%

No. of animals with offspring = 1589 51.9%

... and records = 411 13.4%

No. of animals with unknown sire = 60

No. of animals with unknown dam = 147

No. of animals with both parents unknown = 59

No. of animals with records =

... and unknown sire = 0

... and unknown dam = 0

... and both parents unknown = 0

No. of sires = 307

... with progeny in the data = 114

... with records & progeny in data = 43

No. of dams = 1282

... with progeny in the data = 783

... with records & progeny in data = 368

No. of animals with known/unpruned grand-parents

... with paternal grandsire = 2938

... with paternal granddam = 2812

... with maternal grandsire = 2813

... with maternal granddam = 2724

Inbreeding coefficients for random effect 1 computed

No. of inbred animals = 2616

Average inbreeding coefficient = 2.5605 (in %)

... amongst inbred animals = 2.9970 (in %)

random effect no. = 1 NRM

no. of elements in NRM/GIN inverse 11700

log determinant = -2105.0285008830233

======== end of file ============================06-02-2015==========13:49====

======= Version 30-08-2013 ======================================= **KM** ====

Program WOMBAT: Summary of information from Set-up step

==============================================================================

Analysis type : "muv 2"

Data file : "DADOS.dat"

Pedigree file : "ReducedPedFile.dat"

Parameter file : "wombat.par"

No. of traits = 2

nrec mean sdev min. max.

1 "RF" 1817 5.08663 2.54341 0.00000 19.2000

2 "RFIb" 894 -0.916555E-02 0.602128 -2.36330 4.96970

Numbers of individuals/records for pairs of traits

1 2

1 "RF" 1817 827

2 "RFIb" 827 894

Covariables

1"RF" nrec mean sdev min. max.

1 "idv(2)" 1817 6.09191 3.03138 2.00000 17.0000

2 "idade(1)" 1817 469.100 100.703 306.000 609.000

2"RFIb" nrec mean sdev min. max.

1 "idv(2)" 894 6.16443 3.04994 3.00000 17.0000

2 "idade(1)" 894 368.964 35.9309 267.000 511.000

Fixed effects

1 RF" nlev

1 "gc" 51

2 "mn" 3

2 "RFIb" nlev

1 "gc" 21

Random effects nlev

1 "animal" 3062 NRM

======== end of file ============================06-02-2015==========13:49====

======= Version 30-08-2013 ======================================= **KM** ====

Program WOMBAT: Estimates of covariance components

==============================================================================

Analysis type : "muv 2"

Data file : "DADOS.dat"

Pedigree file : "ReducedPedFile.dat"

Parameter file : "wombat.par"

No. of traits = 2 RF RFIb

No. of records = 2711 1817 894

No. of parameters = 6

Maximum log L = -1777.323

-1/2 AIC & AICC = -1783.323 -1783.338

-1/2 BIC = -1800.948 "Penalty factor" = 3.938

Parameter estimates with approx. sampling erors

1 CHOL Z 1 1 0.235130 0.365388E-01

2 CHOL Z 1 2 -0.347603E-01 0.336855E-01

3 CHOL Z 2 2 -0.614913 0.404034E-01

4 CHOL A 1 1 0.168837E-02 0.803979E-01

5 CHOL A 1 2 0.851590E-01 0.475826E-01

6 CHOL A 2 2 -1.36028 0.187268

Convergence criteria for last 3 iterates

Change in log likelihood = 7.293313 0.020015 0.000010

Change in parameter vector = 0.072098 0.006106 0.000494

Norm of gradient vector = 175.1271 8.6621 0.0947

Newton decrement = -14.0903 -0.0402 -0.0000

***** Estimates of residual covariances ************************************

Order of fit = 2

Covariance matrix

1 1.6004

2 -0.43974E-01 0.29355

Eigenvalues of covariance matrix

Value 1.60 0.29

(%) 84.58 15.42

Trace 1.89

Matrix of correlations and variance ratios

1 0.6146

2 -0.0642 0.8007

Covariances & correlations with approximate sampling errors

1 COVS Z 1 1 1.60041 0.116954 vrat 0.615 0.053

2 COVS Z 1 2 -0.439743E-01 0.425259E-01 corr -0.064 0.062

3 COVS Z 2 2 0.293552 0.234790E-01 vrat 0.801 0.062

***** Estimates for RE 1 "animal" ***************************************

No. of levels = 3062

Covariance structure = NRM

Order of fit = 2

Covariance matrix

1 1.0034

2 0.85303E-01 0.73090E-01

Eigenvalues of covariance matrix

Value 1.01 0.07

(%) 93.93 6.07

Trace 1.08

Matrix of correlations and variance ratios

1 0.3854

2 0.3150 0.1993

Covariances & correlations with approximate sampling errors

4 COVS A 1 1 1.00338 0.161340 vrat 0.385 0.053

5 COVS A 1 2 0.853029E-01 0.481707E-01 corr 0.315 0.178

6 COVS A 2 2 0.730900E-01 0.241663E-01 vrat 0.199 0.062

***** Estimates of phenotypic covariances ***********************************

Covariance matrix

1 2.6038

2 0.41329E-01 0.36664

Eigenvalues of covariance matrix

Value 2.60 0.37

(%) 87.68 12.32

Trace 2.97

Correlation matrix

1 1.0000

2 0.0423 1.0000

Covariances & correlations with approximate sampling errors

7 COVS T 1 1 2.60379 0.103024

8 COVS T 1 2 0.413286E-01 0.363559E-01 corr 0.042 0.037

9 COVS T 2 2 0.366642 0.183035E-01

======== end of file ============================06-02-2015==========13:49====

======= Version 30-08-2013 ======================================= **KM** ====

Program WOMBAT: Summary of Pedigree Information

==============================================================================

Two traits analysis (RFIsf x DMI)

Analysis type : "muv 2"

Data file : "DADOS.dat"

Pedigree file : "ReducedPedFile.dat"

Parameter file : "wombat.par"

No. of animal IDs in data file = = 955

No. of animal IDs in total = = 2288

*****Pedigree Structure for random effect : 1 ****************************

Original no. of animals = 2288

No. of animals after pruning = 2204

... proportion (%) remaining = 96.3

No. of levels w/out records = 1249

No. of levels with records = 955 100.0%

... 1 record(s) = 64 6.7%

... 2 record(s) = 891 93.3%

No. of animals w/out offspring = 828 37.6%

No. of animals with offspring = 1376 62.4%

... and records = 127 5.8%

No. of animals with unknown sire = 59

No. of animals with unknown dam = 145

No. of animals with both parents unknown = 58

No. of animals with records =

... and unknown sire = 0

... and unknown dam = 0

... and both parents unknown = 0

No. of sires = 296

... with progeny in the data = 78

... with records & progeny in data = 18

No. of dams = 1080

... with progeny in the data = 542

... with records & progeny in data = 98

No. of animals with known/unpruned grand-parents

... with paternal grandsire = 2083

... with paternal granddam = 1957

... with maternal grandsire = 1959

... with maternal granddam = 1872

random effect no. = 1 NRM

no. of elements in NRM/GIN inverse 8375

log determinant = -1487.8684515369519

======== end of file ============================01-04-2014==========17:48====

======= Version 30-08-2013 ======================================= **KM** ====

Program WOMBAT: Summary of information from Set-up step

==============================================================================

Analysis type : "muv 2"

Data file : "DADOS.dat"

Pedigree file : "ReducedPedFile.dat"

Parameter file : "wombat.par"

No. of traits = 2

nrec mean sdev min. max.

1 "RFIsf" 891 -0.904938E-02 0.596324 -2.24260 4.91730

2 "DMI" 955 6.83104 1.33500 2.15500 12.6400

Numbers of individuals/records for pairs of traits

1 2

1 "RFIsf" 891 891

2 "DMI" 891 955

Covariables

1"RFIsf" nrec mean sdev min. max.

1 "idv(2)" 891 6.16611 3.04967 3.00000 17.0000

2 "idade(1)" 891 368.979 35.9742 267.000 511.000

2"DMI" nrec mean sdev min. max.

1 "idv(2)" 955 6.15079 3.05304 3.00000 17.0000

2 "idade(1)" 955 369.980 37.3543 267.000 511.000

Fixed effects

1 "RFIsf" nlev

1 "gc" 21

2 "DMI" nlev

1 "gc" 21

Random effects nlev

1 "animal" 2204 NRM

======== end of file ============================01-04-2014==========17:48====

======= Version 30-08-2013 ======================================= **KM** ====

Program WOMBAT: Estimates of covariance components

==============================================================================

Analysis type : "muv 2"

Data file : "DADOS.dat"

Pedigree file : "ReducedPedFile.dat"

Parameter file : "wombat.par"

No. of traits = 2 RFIsf DMI

No. of records = 1846 891 955

No. of parameters = 6

Maximum log L = -72.922

-1/2 AIC & AICC = -78.922 -78.945

-1/2 BIC = -95.405 "Penalty factor" = 3.747

Parameter estimates with approx. sampling erors

1 CHOL Z 1 1 -0.437374 0.565988E-01

2 CHOL Z 1 2 0.404582 0.285991E-01

3 CHOL Z 2 2 -1.03209 0.501770E-01

4 CHOL A 1 1 -0.506065 0.882284E-01

5 CHOL A 1 2 0.182262 0.419388E-01

6 CHOL A 2 2 -1.71835 0.208305

Convergence criteria for last 3 iterates

Change in log likelihood = 4.758907 0.017972 0.000107

Change in parameter vector = 0.040762 0.004485 0.000783

Norm of gradient vector = 136.8772 5.5073 0.3101

Newton decrement = -9.1681 -0.0370 -0.0003

***** Estimates of residual covariances ************************************

Order of fit = 2

Covariance matrix

1 0.29061

2 0.26125 0.41697

Eigenvalues of covariance matrix

Value 0.62 0.09

(%) 87.99 12.01

Trace 0.71

Matrix of correlations and variance ratios

1 0.8163

2 0.7505 0.5343

Covariances & correlations with approximate sampling errors

1 COVS Z 1 1 0.290609 0.230048E-01 vrat 0.816 0.062

2 COVS Z 1 2 0.261250 0.278843E-01 corr 0.751 0.032

3 COVS Z 2 2 0.416967 0.471996E-01 vrat 0.534 0.068

***** Estimates for RE 1 "animal" ***************************************

No. of levels = 2204

Covariance structure = NRM

Order of fit = 2

Covariance matrix

1 0.65390E-01

2 0.10988 0.36344

Eigenvalues of covariance matrix

Value 0.40 0.03

(%) 93.18 6.82

Trace 0.43

Matrix of correlations and variance ratios

1 0.1837

2 0.7128 0.4657

Covariances & correlations with approximate sampling errors

4 COVS A 1 1 0.653902E-01 0.232884E-01 vrat 0.184 0.062

5 COVS A 1 2 0.109879 0.321897E-01 corr 0.713 0.090

6 COVS A 2 2 0.363444 0.641321E-01 vrat 0.466 0.068

***** Estimates of phenotypic covariances ***********************************

Covariance matrix

1 0.35600

2 0.37113 0.78041

Eigenvalues of covariance matrix

Value 1.00 0.14

(%) 87.62 12.38

Trace 1.14

Correlation matrix

1 1.0000

2 0.7041 1.0000

Covariances & correlations with approximate sampling errors

7 COVS T 1 1 0.355999 0.175511E-01

8 COVS T 1 2 0.371130 0.226069E-01 corr 0.704 0.018

9 COVS T 2 2 0.780411 0.407550E-01

======== end of file ============================01-04-2014==========17:48====

======= Version 30-08-2013 ======================================= **KM** ====

Program WOMBAT: Summary of Pedigree Information

==============================================================================

Two traits analysis (RFIsf x ADG)

Analysis type : "muv 2"

Data file : "DADOS.dat"

Pedigree file : "ReducedPedFile.dat"

Parameter file : "wombat.par"

No. of animal IDs in data file = = 955

No. of animal IDs in total = = 2288

*****Pedigree Structure for random effect : 1 ****************************

Original no. of animals = 2288

No. of animals after pruning = 2204

... proportion (%) remaining = 96.3

No. of levels w/out records = 1249

No. of levels with records = 955 100.0%

... 1 record(s) = 64 6.7%

... 2 record(s) = 891 93.3%

No. of animals w/out offspring = 828 37.6%

No. of animals with offspring = 1376 62.4%

... and records = 127 5.8%

No. of animals with unknown sire = 59

No. of animals with unknown dam = 145

No. of animals with both parents unknown = 58

No. of animals with records =

... and unknown sire = 0

... and unknown dam = 0

... and both parents unknown = 0

No. of sires = 296

... with progeny in the data = 78

... with records & progeny in data = 18

No. of dams = 1080

... with progeny in the data = 542

... with records & progeny in data = 98

No. of animals with known/unpruned grand-parents

... with paternal grandsire = 2083

... with paternal granddam = 1957

... with maternal grandsire = 1959

... with maternal granddam = 1872

random effect no. = 1 NRM

no. of elements in NRM/GIN inverse 8375

log determinant = -1487.8684515369519

======== end of file ============================01-04-2014==========17:49====

======= Version 30-08-2013 ======================================= **KM** ====

Program WOMBAT: Summary of information from Set-up step

==============================================================================

Analysis type : "muv 2"

Data file : "DADOS.dat"

Pedigree file : "ReducedPedFile.dat"

Parameter file : "wombat.par"

No. of traits = 2

nrec mean sdev min. max.

1 "RFIsf" 891 -0.904938E-02 0.596324 -2.24260 4.91730

2 "ADG" 955 1.00846 0.260202 0.176000 1.71800

Numbers of individuals/records for pairs of traits

1 2

1 "RFIsf" 891 891

2 "ADG" 891 955

Covariables

1"RFIsf" nrec mean sdev min. max.

1 "idv(2)" 891 6.16611 3.04967 3.00000 17.0000

2 "idade(1)" 891 368.979 35.9742 267.000 511.000

2"ADG" nrec mean sdev min. max.

1 "idv(2)" 955 6.15079 3.05304 3.00000 17.0000

2 "idade(1)" 955 369.980 37.3543 267.000 511.000

Fixed effects

1 "RFIsf" nlev

1 "gc" 21

2 "ADG" nlev

1 "gc" 21

Random effects nlev

1 "animal" 2204 NRM

======== end of file ============================01-04-2014==========17:49====

======= Version 30-08-2013 ======================================= **KM** ====

Program WOMBAT: Estimates of covariance components

==============================================================================

Analysis type : "muv 2"

Data file : "DADOS.dat"

Pedigree file : "ReducedPedFile.dat"

Parameter file : "wombat.par"

No. of traits = 2 RFIsf ADG

No. of records = 1846 891 955

No. of parameters = 6

Maximum log L = 1221.905

-1/2 AIC & AICC = 1215.905 1215.882

-1/2 BIC = 1199.422 "Penalty factor" = 3.747

Parameter estimates with approx. sampling erors

1 CHOL Z 1 1 -0.627251 0.407761E-01

2 CHOL Z 1 2 -0.153128E-01 0.773060E-02

3 CHOL Z 2 2 -2.13903 0.546625E-01

4 CHOL A 1 1 -1.30748 0.165391

5 CHOL A 1 2 0.398733E-01 0.178194E-01

6 CHOL A 2 2 -2.35764 0.120878

Convergence criteria for last 3 iterates

Change in log likelihood = 7.188385 0.048955 0.000076

Change in parameter vector = 0.018629 0.002708 0.000533

Norm of gradient vector = 635.8471 31.4583 0.4554

Newton decrement = -13.8540 -0.0987 -0.0002

***** Estimates of residual covariances ************************************

Order of fit = 2

Covariance matrix

1 0.28522

2 -0.81779E-02 0.14104E-01

Eigenvalues of covariance matrix

Value 0.29 0.01

(%) 95.37 4.63

Trace 0.30

Matrix of correlations and variance ratios

1 0.7958

2 -0.1289 0.5721

Covariances & correlations with approximate sampling errors

1 COVS Z 1 1 0.285218 0.232601E-01 vrat 0.796 0.064

2 COVS Z 1 2 -0.817790E-02 0.410906E-02 corr -0.129 0.066

3 COVS Z 2 2 0.141039E-01 0.148463E-02 vrat 0.572 0.067

***** Estimates for RE 1 "animal" ***************************************

No. of levels = 2204

Covariance structure = NRM

Order of fit = 2

Covariance matrix

1 0.73171E-01

2 0.10786E-01 0.10547E-01

Eigenvalues of covariance matrix

Value 0.07 0.01

(%) 89.56 10.44

Trace 0.08

Matrix of correlations and variance ratios

1 0.2042

2 0.3882 0.4279

Covariances & correlations with approximate sampling errors

4 COVS A 1 1 0.731712E-01 0.242037E-01 vrat 0.204 0.064

5 COVS A 1 2 0.107858E-01 0.478930E-02 corr 0.388 0.170

6 COVS A 2 2 0.105473E-01 0.195608E-02 vrat 0.428 0.067

***** Estimates of phenotypic covariances ***********************************

Covariance matrix

1 0.35839

2 0.26079E-02 0.24651E-01

Eigenvalues of covariance matrix

Value 0.36 0.02

(%) 93.57 6.43

Trace 0.38

Correlation matrix

1 1.0000

2 0.0277 1.0000

Covariances & correlations with approximate sampling errors

7 COVS T 1 1 0.358389 0.179457E-01

8 COVS T 1 2 0.260789E-02 0.337561E-02 corr 0.028 0.036

9 COVS T 2 2 0.246513E-01 0.127253E-02

======== end of file ============================01-04-2014==========17:49====

======= Version 30-08-2013 ======================================= **KM** ====

Program WOMBAT: Summary of Pedigree Information

==============================================================================

Two traits analysis (WS x RFIsf)

Analysis type : "muv 2"

Data file : "DADOS.dat"

Pedigree file : "ReducedPedFile.dat"

Parameter file : "wombat.par"

No. of animal IDs in data file = = 8090

No. of animal IDs in total = = 8489

*****Pedigree Structure for random effect : 1 ****************************

Original no. of animals = 8489

No. of animals after pruning = 8432

... proportion (%) remaining = 99.3

No. of levels w/out records = 342

No. of levels with records = 8090 100.0%

... 1 record(s) = 7211 89.1%

... 2 record(s) = 879 10.9%

No. of animals w/out offspring = 6051 71.8%

No. of animals with offspring = 2381 28.2%

... and records = 2039 24.2%

No. of animals with unknown sire = 313

No. of animals with unknown dam = 384

No. of animals with both parents unknown = 312

No. of animals with records =

... and unknown sire = 1

... and unknown dam = 53

... and both parents unknown = 0

No. of sires = 325

... with progeny in the data = 320

... with records & progeny in data = 288

No. of dams = 2056

... with progeny in the data = 2056

... with records & progeny in data = 1751

No. of animals with known/unpruned grand-parents

... with paternal grandsire = 7357

... with paternal granddam = 7055

... with maternal grandsire = 6534

... with maternal granddam = 6467

Inbreeding coefficients for random effect 1 computed

No. of inbred animals = 5143

Average inbreeding coefficient = 1.5416 (in %)

... amongst inbred animals = 2.5274 (in %)

random effect no. = 1 NRM

no. of elements in NRM/GIN inverse 31835

log determinant = -5688.0517714250827

random effect no. = 2 IDE

no. of elements in NRM/GIN inverse 0

log determinant = 0.0000000000000000

======== end of file ============================06-02-2015==========13:52====

======= Version 30-08-2013 ======================================= **KM** ====

Program WOMBAT: Summary of information from Set-up step

==============================================================================

Analysis type : "muv 2"

Data file : "DADOS.dat"

Pedigree file : "ReducedPedFile.dat"

Parameter file : "wombat.par"

No. of traits = 2

nrec mean sdev min. max.

1 "WS" 8078 299.703 49.6462 160.030 489.760

2 "RFIsf" 891 -0.904938E-02 0.596324 -2.24260 4.91730

Numbers of individuals/records for pairs of traits

1 2

1 "WS" 8078 879

2 "RFIsf" 879 891

Covariables

1"WS" nrec mean sdev min. max.

1 "idv(2)" 8078 6.63568 3.07616 2.00000 18.0000

2 "idade(1)" 8078 473.414 105.028 293.000 645.000

2"RFIsf" nrec mean sdev min. max.

1 "idv(2)" 891 6.16611 3.04967 3.00000 17.0000

2 "idade(1)" 891 368.979 35.9742 267.000 511.000

Fixed effects

1 "WS" nlev

1 "gc" 201

2 "mn" 4

2 "RFIsf" nlev

1 "gc" 21

Random effects nlev

1 "animal" 8432 NRM

2 "peanim" 2107 IDE

======== end of file ============================06-02-2015==========13:52====

======= Version 30-08-2013 ======================================= **KM** ====

Program WOMBAT: Estimates of covariance components

==============================================================================

Analysis type : "muv 2"

Data file : "DADOS.dat"

Pedigree file : "ReducedPedFile.dat"

Parameter file : "wombat.par"

No. of traits = 2 WS RFIsf

No. of records = 8969 8078 891

No. of parameters = 7

Maximum log L = -30187.600

-1/2 AIC & AICC = -30194.600 -30194.606

-1/2 BIC = -30219.364 "Penalty factor" = 4.538

Parameter estimates with approx. sampling erors

1 CHOL Z 1 1 3.02584 0.211282E-01

2 CHOL Z 1 2 -0.164523E-02 0.307047E-01

3 CHOL Z 2 2 -0.624004 0.408895E-01

4 CHOL A 1 1 2.88223 0.479607E-01

5 CHOL A 1 2 0.591253E-01 0.397616E-01

6 CHOL A 2 2 -1.34522 0.183192

7 CHOL B 1 1 2.32970 0.507071E-01

Convergence criteria for last 3 iterates

Change in log likelihood = 0.016337 0.001318 0.000108

Change in parameter vector = 0.001718 0.000504 0.000141

Norm of gradient vector = 3.4413 0.8659 0.2529

Newton decrement = -0.0258 -0.0021 -0.0002

***** Estimates of residual covariances ************************************

Order of fit = 2

Covariance matrix

1 424.83

2 -0.33910E-01 0.28708

Eigenvalues of covariance matrix

Value 424.83 0.29

(%) 99.93 0.07

Trace 425.11

Matrix of correlations and variance ratios

1 0.5003

2 -0.0031 0.8009

Covariances & correlations with approximate sampling errors

1 COVS Z 1 1 424.826 17.9516 vrat 0.500 0.027

2 COVS Z 1 2 -0.339104E-01 0.632844 corr -0.003 0.057

3 COVS Z 2 2 0.287079 0.234779E-01 vrat 0.801 0.064

***** Estimates for RE 1 "animal" ***************************************

No. of levels = 8432

Covariance structure = NRM

Order of fit = 2

Covariance matrix

1 318.77

2 1.0556 0.71347E-01

Eigenvalues of covariance matrix

Value 318.77 0.07

(%) 99.98 0.02

Trace 318.84

Matrix of correlations and variance ratios

1 0.3754

2 0.2214 0.1991

Covariances & correlations with approximate sampling errors

4 COVS A 1 1 318.769 30.5768 vrat 0.375 0.030

5 COVS A 1 2 1.05563 0.711926 corr 0.221 0.154

6 COVS A 2 2 0.713467E-01 0.243318E-01 vrat 0.199 0.064

***** Estimates for RE 2 "peanim" ***************************************

No. of levels = 2107

Covariance structure = IDE

Order of fit = 1

Covariance matrix

1 105.57

Matrix of correlations and variance ratios

1 0.1243

Covariances & correlations with approximate sampling errors

7 COVS B 1 1 105.572 10.7065 vrat 0.124 0.012

***** Estimates of phenotypic covariances ***********************************

Covariance matrix

1 849.17

2 1.0217 0.35843

Eigenvalues of covariance matrix

Value 849.17 0.36

(%) 99.96 0.04

Trace 849.53

Correlation matrix

1 1.0000

2 0.0586 1.0000

Covariances & correlations with approximate sampling errors

8 COVS T 1 1 849.167 18.5615

9 COVS T 1 2 1.02172 0.581632 corr 0.059 0.033

10 COVS T 2 2 0.358425 0.179390E-01

======== end of file ============================06-02-2015==========13:52====

======= Version 30-08-2013 ======================================= **KM** ====

Program WOMBAT: Summary of Pedigree Information

==============================================================================

Two traits analysis (HH x RFIsf)

Analysis type : "muv 2"

Data file : "DADOS.dat"

Pedigree file : "ReducedPedFile.dat"

Parameter file : "wombat.par"

No. of animal IDs in data file = = 6559

No. of animal IDs in total = = 7099

*****Pedigree Structure for random effect : 1 ****************************

Original no. of animals = 7099

No. of animals after pruning = 7016

... proportion (%) remaining = 98.8

No. of levels w/out records = 457

No. of levels with records = 6559 100.0%

... 1 record(s) = 5679 86.6%

... 2 record(s) = 880 13.4%

No. of animals w/out offspring = 4828 68.8%

No. of animals with offspring = 2188 31.2%

... and records = 1731 24.7%

No. of animals with unknown sire = 148

No. of animals with unknown dam = 237

No. of animals with both parents unknown = 148

No. of animals with records =

... and unknown sire = 0

... and unknown dam = 18

... and both parents unknown = 0

No. of sires = 320

... with progeny in the data = 283

... with records & progeny in data = 240

No. of dams = 1868

... with progeny in the data = 1837

... with records & progeny in data = 1491

No. of animals with known/unpruned grand-parents

... with paternal grandsire = 6737

... with paternal granddam = 6483

... with maternal grandsire = 6338

... with maternal granddam = 6110

Inbreeding coefficients for random effect 1 computed

No. of inbred animals = 5148

Average inbreeding coefficient = 1.8503 (in %)

... amongst inbred animals = 2.5217 (in %)

random effect no. = 1 NRM

no. of elements in NRM/GIN inverse 26819

log determinant = -4814.0947710995106

======== end of file ============================06-02-2015==========13:53====

======= Version 30-08-2013 ======================================= **KM** ====

Program WOMBAT: Summary of information from Set-up step

==============================================================================

Analysis type : "muv 2"

Data file : "DADOS.dat"

Pedigree file : "ReducedPedFile.dat"

Parameter file : "wombat.par"

No. of traits = 2

nrec mean sdev min. max.

1 "HH" 6548 132.269 5.43028 100.000 149.000

2 "RFIsf" 891 -0.904938E-02 0.596324 -2.24260 4.91730

Numbers of individuals/records for pairs of traits

1 2

1 "HH" 6548 880

2 "RFIsf" 880 891

Covariables

1"HH" nrec mean sdev min. max.

1 "idv(2)" 6548 6.40043 2.93455 2.00000 17.0000

2 "idade(1)" 6548 472.701 107.333 293.000 645.000

2"RFIsf" nrec mean sdev min. max.

1 "idv(2)" 891 6.16611 3.04967 3.00000 17.0000

2 "idade(1)" 891 368.979 35.9742 267.000 511.000

Fixed effects

1 "HH" nlev

1 "gc" 169

2 "mn" 4

2 "RFIsf" nlev

1 "gc" 21

Random effects nlev

1 "animal" 7016 NRM

======== end of file ============================06-02-2015==========13:53====

======= Version 30-08-2013 ======================================= **KM** ====

Program WOMBAT: Estimates of covariance components

==============================================================================

Analysis type : "muv 2"

Data file : "DADOS.dat"

Pedigree file : "ReducedPedFile.dat"

Parameter file : "wombat.par"

No. of traits = 2 HH RFIsf

No. of records = 7439 6548 891

No. of parameters = 6

Maximum log L = -11439.268

-1/2 AIC & AICC = -11445.268 -11445.274

-1/2 BIC = -11465.930 "Penalty factor" = 4.444

Parameter estimates with approx. sampling erors

1 CHOL Z 1 1 0.897268 0.266277E-01

2 CHOL Z 1 2 0.146040E-01 0.382204E-01

3 CHOL Z 2 2 -0.634332 0.419179E-01

4 CHOL A 1 1 1.12000 0.316263E-01

5 CHOL A 1 2 0.235204E-01 0.400734E-01

6 CHOL A 2 2 -1.27169 0.160823

Convergence criteria for last 3 iterates

Change in log likelihood = 0.090861 0.001481 0.000032

Change in parameter vector = 0.007074 0.000987 0.000149

Norm of gradient vector = 18.0915 1.3791 0.1915

Newton decrement = -0.1643 -0.0026 -0.0001

***** Estimates of residual covariances ************************************

Order of fit = 2

Covariance matrix

1 6.0167

2 0.35822E-01 0.28142

Eigenvalues of covariance matrix

Value 6.02 0.28

(%) 95.54 4.46

Trace 6.30

Matrix of correlations and variance ratios

1 0.3904

2 0.0275 0.7805

Covariances & correlations with approximate sampling errors

1 COVS Z 1 1 6.01668 0.320421 vrat 0.390 0.026

2 COVS Z 1 2 0.358221E-01 0.937603E-01 corr 0.028 0.072

3 COVS Z 2 2 0.281420 0.235259E-01 vrat 0.780 0.065

***** Estimates for RE 1 "animal" ***************************************

No. of levels = 7016

Covariance structure = NRM

Order of fit = 2

Covariance matrix

1 9.3934

2 0.72087E-01 0.79154E-01

Eigenvalues of covariance matrix

Value 9.39 0.08

(%) 99.17 0.83

Trace 9.47

Matrix of correlations and variance ratios

1 0.6096

2 0.0836 0.2195

Covariances & correlations with approximate sampling errors

4 COVS A 1 1 9.39336 0.594154 vrat 0.610 0.026

5 COVS A 1 2 0.720868E-01 0.122871 corr 0.084 0.144

6 COVS A 2 2 0.791537E-01 0.250953E-01 vrat 0.220 0.065

***** Estimates of phenotypic covariances ***********************************

Covariance matrix

1 15.410

2 0.10791 0.36057

Eigenvalues of covariance matrix

Value 15.41 0.36

(%) 97.72 2.28

Trace 15.77

Correlation matrix

1 1.0000

2 0.0458 1.0000

Covariances & correlations with approximate sampling errors

7 COVS T 1 1 15.4100 0.382696

8 COVS T 1 2 0.107909 0.889641E-01 corr 0.046 0.038

9 COVS T 2 2 0.360574 0.181860E-01

======== end of file ============================06-02-2015==========13:54====

======= Version 30-08-2013 ======================================= **KM** ====

Program WOMBAT: Summary of Pedigree Information

==============================================================================

Two traits analysis (CC x RFIsf)

Analysis type : "muv 2"

Data file : "DADOS.dat"

Pedigree file : "ReducedPedFile.dat"

Parameter file : "wombat.par"

No. of animal IDs in data file = = 3941

No. of animal IDs in total = = 5416

*****Pedigree Structure for random effect : 1 ****************************

Original no. of animals = 5416

No. of animals after pruning = 5328

... proportion (%) remaining = 98.4

No. of levels w/out records = 1387

No. of levels with records = 3941 100.0%

... 1 record(s) = 3115 79.0%

... 2 record(s) = 826 21.0%

No. of animals w/out offspring = 3309 62.1%

No. of animals with offspring = 2019 37.9%

... and records = 632 11.9%

No. of animals with unknown sire = 94

No. of animals with unknown dam = 188

No. of animals with both parents unknown = 94

No. of animals with records =

... and unknown sire = 0

... and unknown dam = 0

... and both parents unknown = 0

No. of sires = 318

... with progeny in the data = 255

... with records & progeny in data = 217

No. of dams = 1701

... with progeny in the data = 1508

... with records & progeny in data = 412

No. of animals with known/unpruned grand-parents

... with paternal grandsire = 5140

... with paternal granddam = 4952

... with maternal grandsire = 4951

... with maternal granddam = 4736

Inbreeding coefficients for random effect 1 computed

No. of inbred animals = 4322

Average inbreeding coefficient = 2.1289 (in %)

... amongst inbred animals = 2.6244 (in %)

random effect no. = 1 NRM

no. of elements in NRM/GIN inverse 20457

log determinant = -3670.4017279806212

======== end of file ============================06-02-2015==========13:55====

======= Version 30-08-2013 ======================================= **KM** ====

Program WOMBAT: Summary of information from Set-up step

==============================================================================

Analysis type : "muv 2"

Data file : "DADOS.dat"

Pedigree file : "ReducedPedFile.dat"

Parameter file : "wombat.par"

No. of traits = 2

nrec mean sdev min. max.

1 "CC" 3876 164.140 8.72028 128.000 192.000

2 "RFIsf" 891 -0.904938E-02 0.596324 -2.24260 4.91730

Numbers of individuals/records for pairs of traits

1 2

1 "CC" 3876 826

2 "RFIsf" 826 891

Covariables

1"CC" nrec mean sdev min. max.

1 "idv(2)" 3876 6.27632 2.87660 2.00000 17.0000

2 "idade(1)" 3876 431.403 109.187 293.000 725.000

2"RFIsf" nrec mean sdev min. max.

1 "idv(2)" 891 6.16611 3.04967 3.00000 17.0000

2 "idade(1)" 891 368.979 35.9742 267.000 511.000

Fixed effects

1 "CC" nlev

1 "gc" 100

2 "mn" 4

2 "RFIsf" nlev

1 "gc" 21

Random effects nlev

1 "animal" 5328 NRM

======== end of file ============================06-02-2015==========13:55====

======= Version 30-08-2013 ======================================= **KM** ====

Program WOMBAT: Estimates of covariance components

==============================================================================

Analysis type : "muv 2"

Data file : "DADOS.dat"

Pedigree file : "ReducedPedFile.dat"

Parameter file : "wombat.par"

No. of traits = 2 CC RFIsf

No. of records = 4767 3876 891

No. of parameters = 6

Maximum log L = -8731.863

-1/2 AIC & AICC = -8737.863 -8737.872

-1/2 BIC = -8757.189 "Penalty factor" = 4.221

Parameter estimates with approx. sampling erors

1 CHOL Z 1 1 1.61747 0.233880E-01

2 CHOL Z 1 2 0.532776E-02 0.307087E-01

3 CHOL Z 2 2 -0.632455 0.415085E-01

4 CHOL A 1 1 1.18499 0.728401E-01

5 CHOL A 1 2 0.402513E-01 0.509168E-01

6 CHOL A 2 2 -1.28582 0.165185

Convergence criteria for last 3 iterates

Change in log likelihood = 1.722457 0.019368 0.000495

Change in parameter vector = 0.036228 0.005125 0.000880

Norm of gradient vector = 94.0882 5.9466 0.3925

Newton decrement = -3.3279 -0.0342 -0.0008

***** Estimates of residual covariances ************************************

Order of fit = 2

Covariance matrix

1 25.405

2 0.26854E-01 0.28229

Eigenvalues of covariance matrix

Value 25.40 0.28

(%) 98.90 1.10

Trace 25.69

Matrix of correlations and variance ratios

1 0.7037

2 0.0100 0.7834

Covariances & correlations with approximate sampling errors

1 COVS Z 1 1 25.4046 1.18833 vrat 0.704 0.038

2 COVS Z 1 2 0.268535E-01 0.154796 corr 0.010 0.058

3 COVS Z 2 2 0.282293 0.234307E-01 vrat 0.783 0.065

***** Estimates for RE 1 "animal" ***************************************

No. of levels = 5328

Covariance structure = NRM

Order of fit = 2

Covariance matrix

1 10.697

2 0.13165 0.78031E-01

Eigenvalues of covariance matrix

Value 10.70 0.08

(%) 99.29 0.71

Trace 10.78

Matrix of correlations and variance ratios

1 0.2963

2 0.1441 0.2166

Covariances & correlations with approximate sampling errors

4 COVS A 1 1 10.6972 1.55837 vrat 0.296 0.038

5 COVS A 1 2 0.131648 0.166851 corr 0.144 0.184

6 COVS A 2 2 0.780306E-01 0.248288E-01 vrat 0.217 0.065

***** Estimates of phenotypic covariances ***********************************

Covariance matrix

1 36.102

2 0.15850 0.36032

Eigenvalues of covariance matrix

Value 36.10 0.36

(%) 99.01 0.99

Trace 36.46

Correlation matrix

1 1.0000

2 0.0439 1.0000

Covariances & correlations with approximate sampling errors

7 COVS T 1 1 36.1018 0.972591

8 COVS T 1 2 0.158501 0.133247 corr 0.044 0.037

9 COVS T 2 2 0.360324 0.181396E-01

======== end of file ============================06-02-2015==========13:55====

======= Version 30-08-2013 ======================================= **KM** ====

Program WOMBAT: Summary of Pedigree Information

==============================================================================

Two traits analysis (LEA x RFIsf)

Analysis type : "muv 2"

Data file : "DADOS.dat"

Pedigree file : "ReducedPedFile.dat"

Parameter file : "wombat.par"

No. of animal IDs in data file = = 2350

No. of animal IDs in total = = 3729

*****Pedigree Structure for random effect : 1 ****************************

Original no. of animals = 3729

No. of animals after pruning = 3638

... proportion (%) remaining = 97.6

No. of levels w/out records = 1288

No. of levels with records = 2350 100.0%

... 1 record(s) = 1526 64.9%

... 2 record(s) = 824 35.1%

No. of animals w/out offspring = 1889 51.9%

No. of animals with offspring = 1749 48.1%

... and records = 461 12.7%

No. of animals with unknown sire = 64

No. of animals with unknown dam = 161

No. of animals with both parents unknown = 64

No. of animals with records =

... and unknown sire = 0

... and unknown dam = 0

... and both parents unknown = 0

No. of sires = 313

... with progeny in the data = 180

... with records & progeny in data = 84

No. of dams = 1436

... with progeny in the data = 1036

... with records & progeny in data = 371

No. of animals with known/unpruned grand-parents

... with paternal grandsire = 3493

... with paternal granddam = 3355

... with maternal grandsire = 3364

... with maternal granddam = 3240

Inbreeding coefficients for random effect 1 computed

No. of inbred animals = 3107

Average inbreeding coefficient = 2.3747 (in %)

... amongst inbred animals = 2.7805 (in %)

random effect no. = 1 NRM

no. of elements in NRM/GIN inverse 13937

log determinant = -2503.1878537727198

======== end of file ============================06-02-2015==========13:58====

======= Version 30-08-2013 ======================================= **KM** ====

Program WOMBAT: Summary of information from Set-up step

==============================================================================

Analysis type : "muv 2"

Data file : "DADOS.dat"

Pedigree file : "ReducedPedFile.dat"

Parameter file : "wombat.par"

No. of traits = 2

nrec mean sdev min. max.

1 "LEA" 2283 51.4305 8.89390 21.4000 83.4000

2 "RFIsf" 891 -0.904938E-02 0.596324 -2.24260 4.91730

Numbers of individuals/records for pairs of traits

1 2

1 "LEA" 2283 824

2 "RFIsf" 824 891

Covariables

1"LEA" nrec mean sdev min. max.

1 "idv(2)" 2283 6.11082 2.92852 2.00000 17.0000

2 "idade(1)" 2283 448.951 98.8881 306.000 609.000

2"RFIsf" nrec mean sdev min. max.

1 "idv(2)" 891 6.16611 3.04967 3.00000 17.0000

2 "idade(1)" 891 368.979 35.9742 267.000 511.000

Fixed effects

1 "LEA" nlev

1 "gc" 65

2 "mn" 4

2 "RFIsf" nlev

1 "gc" 21

Random effects nlev

1 "animal" 3638 NRM

======== end of file ============================06-02-2015==========13:58====

======= Version 30-08-2013 ======================================= **KM** ====

Program WOMBAT: Estimates of covariance components

==============================================================================

Analysis type : "muv 2"

Data file : "DADOS.dat"

Pedigree file : "ReducedPedFile.dat"

Parameter file : "wombat.par"

No. of traits = 2 LEA RFIsf

No. of records = 3174 2283 891

No. of parameters = 6

Maximum log L = -5104.694

-1/2 AIC & AICC = -5110.694 -5110.707

-1/2 BIC = -5128.791 "Penalty factor" = 4.016

Parameter estimates with approx. sampling erors

1 CHOL Z 1 1 1.49170 0.394436E-01

2 CHOL Z 1 2 0.283612E-01 0.366932E-01

3 CHOL Z 2 2 -0.639084 0.420111E-01

4 CHOL A 1 1 1.41513 0.679102E-01

5 CHOL A 1 2 -0.136101E-01 0.492385E-01

6 CHOL A 2 2 -1.25272 0.153959

Convergence criteria for last 3 iterates

Change in log likelihood = 30.480911 0.559032 0.000435

Change in parameter vector = 0.091488 0.014327 0.000546

Norm of gradient vector = 314.9049 37.5388 0.9584

Newton decrement = -56.3141 -1.0983 -0.0009

***** Estimates of residual covariances ************************************

Order of fit = 2

Covariance matrix

1 19.755

2 0.12606 0.27935

Eigenvalues of covariance matrix

Value 19.76 0.28

(%) 98.61 1.39

Trace 20.03

Matrix of correlations and variance ratios

1 0.5382

2 0.0537 0.7734

Covariances & correlations with approximate sampling errors

1 COVS Z 1 1 19.7548 1.55840 vrat 0.538 0.051

2 COVS Z 1 2 0.126055 0.163116 corr 0.054 0.069

3 COVS Z 2 2 0.279351 0.233779E-01 vrat 0.773 0.065

***** Estimates for RE 1 "animal" ***************************************

No. of levels = 3638

Covariance structure = NRM

Order of fit = 2

Covariance matrix

1 16.950

2 -0.56033E-01 0.81825E-01

Eigenvalues of covariance matrix

Value 16.95 0.08

(%) 99.52 0.48

Trace 17.03

Matrix of correlations and variance ratios

1 0.4618

2 -0.0476 0.2266

Covariances & correlations with approximate sampling errors

4 COVS A 1 1 16.9500 2.30215 vrat 0.462 0.051

5 COVS A 1 2 -0.560332E-01 0.202658 corr -0.048 0.172

6 COVS A 2 2 0.818254E-01 0.251221E-01 vrat 0.227 0.065

***** Estimates of phenotypic covariances ***********************************

Covariance matrix

1 36.705

2 0.70022E-01 0.36118

Eigenvalues of covariance matrix

Value 36.70 0.36

(%) 99.03 0.97

Trace 37.07

Correlation matrix

1 1.0000

2 0.0192 1.0000

Covariances & correlations with approximate sampling errors

7 COVS T 1 1 36.7048 1.36563

8 COVS T 1 2 0.700221E-01 0.141252 corr 0.019 0.039

9 COVS T 2 2 0.361177 0.182500E-01

======== end of file ============================06-02-2015==========13:58====

======= Version 30-08-2013 ======================================= **KM** ====

Program WOMBAT: Summary of Pedigree Information

==============================================================================

Two traits analysis (BF x RFIsf)

Analysis type : "muv 2"

Data file : "DADOS.dat"

Pedigree file : "ReducedPedFile.dat"

Parameter file : "wombat.par"

No. of animal IDs in data file = = 2350

No. of animal IDs in total = = 3729

*****Pedigree Structure for random effect : 1 ****************************

Original no. of animals = 3729

No. of animals after pruning = 3638

... proportion (%) remaining = 97.6

No. of levels w/out records = 1288

No. of levels with records = 2350 100.0%

... 1 record(s) = 1524 64.9%

... 2 record(s) = 826 35.1%

No. of animals w/out offspring = 1889 51.9%

No. of animals with offspring = 1749 48.1%

... and records = 461 12.7%

No. of animals with unknown sire = 64

No. of animals with unknown dam = 161

No. of animals with both parents unknown = 64

No. of animals with records =

... and unknown sire = 0

... and unknown dam = 0

... and both parents unknown = 0

No. of sires = 313

... with progeny in the data = 180

... with records & progeny in data = 84

No. of dams = 1436

... with progeny in the data = 1036

... with records & progeny in data = 371

No. of animals with known/unpruned grand-parents

... with paternal grandsire = 3493

... with paternal granddam = 3355

... with maternal grandsire = 3364

... with maternal granddam = 3240

Inbreeding coefficients for random effect 1 computed

No. of inbred animals = 3107

Average inbreeding coefficient = 2.3747 (in %)

... amongst inbred animals = 2.7805 (in %)

random effect no. = 1 NRM

no. of elements in NRM/GIN inverse 13937

log determinant = -2503.1878537727198

======== end of file ============================06-02-2015==========14:04====

======= Version 30-08-2013 ======================================= **KM** ====

Program WOMBAT: Summary of information from Set-up step

==============================================================================

Analysis type : "muv 2"

Data file : "DADOS.dat"

Pedigree file : "ReducedPedFile.dat"

Parameter file : "wombat.par"

No. of traits = 2

nrec mean sdev min. max.

1 "BF" 2285 1.75922 1.42798 0.00000 10.5000

2 "RFIsf" 891 -0.904938E-02 0.596324 -2.24260 4.91730

Numbers of individuals/records for pairs of traits

1 2

1 "BF" 2285 826

2 "RFIsf" 826 891

Covariables

1"BF" nrec mean sdev min. max.

1 "idv(2)" 2285 6.10810 2.92868 2.00000 17.0000

2 "idade(1)" 2285 449.081 98.9421 306.000 609.000

2"RFIsf" nrec mean sdev min. max.

1 "idv(2)" 891 6.16611 3.04967 3.00000 17.0000

2 "idade(1)" 891 368.979 35.9742 267.000 511.000

Fixed effects

1 "BF" nlev

1 "gc" 65

2 "mn" 4

2 "RFIsf" nlev

1 "gc" 21

Random effects nlev

1 "animal" 3638 NRM

======== end of file ============================06-02-2015==========14:04====

======= Version 30-08-2013 ======================================= **KM** ====

Program WOMBAT: Estimates of covariance components

==============================================================================

Analysis type : "muv 2"

Data file : "DADOS.dat"

Pedigree file : "ReducedPedFile.dat"

Parameter file : "wombat.par"

No. of traits = 2 BF RFIsf

No. of records = 3176 2285 891

No. of parameters = 6

Maximum log L = -1095.738

-1/2 AIC & AICC = -1101.738 -1101.751

-1/2 BIC = -1119.837 "Penalty factor" = 4.017

Parameter estimates with approx. sampling erors

1 CHOL Z 1 1 -0.220863 0.291809E-01

2 CHOL Z 1 2 -0.414701E-01 0.310746E-01

3 CHOL Z 2 2 -0.631356 0.409543E-01

4 CHOL A 1 1 -0.641673 0.853527E-01

5 CHOL A 1 2 0.902569E-01 0.500744E-01

6 CHOL A 2 2 -1.35376 0.187674

Convergence criteria for last 3 iterates

Change in log likelihood = 12.035718 0.028474 0.000033

Change in parameter vector = 0.096580 0.006810 0.000733

Norm of gradient vector = 264.8992 11.6298 0.2327

Newton decrement = -23.0813 -0.0578 -0.0001

***** Estimates of residual covariances ************************************

Order of fit = 2

Covariance matrix

1 0.64293

2 -0.33252E-01 0.28461

Eigenvalues of covariance matrix

Value 0.65 0.28

(%) 69.65 30.35

Trace 0.93

Matrix of correlations and variance ratios

1 0.6988

2 -0.0777 0.7918

Covariances & correlations with approximate sampling errors

1 COVS Z 1 1 0.642926 0.375223E-01 vrat 0.699 0.046

2 COVS Z 1 2 -0.332518E-01 0.249046E-01 corr -0.078 0.058

3 COVS Z 2 2 0.284606 0.231132E-01 vrat 0.792 0.063

***** Estimates for RE 1 "animal" ***************************************

No. of levels = 3638

Covariance structure = NRM

Order of fit = 2

Covariance matrix

1 0.27711

2 0.47512E-01 0.74849E-01

Eigenvalues of covariance matrix

Value 0.29 0.06

(%) 81.75 18.25

Trace 0.35

Matrix of correlations and variance ratios

1 0.3012

2 0.3299 0.2082

Covariances & correlations with approximate sampling errors

4 COVS A 1 1 0.277108 0.473039E-01 vrat 0.301 0.046

5 COVS A 1 2 0.475122E-01 0.264405E-01 corr 0.330 0.187

6 COVS A 2 2 0.748490E-01 0.240329E-01 vrat 0.208 0.063

***** Estimates of phenotypic covariances ***********************************

Covariance matrix

1 0.92003

2 0.14260E-01 0.35945

Eigenvalues of covariance matrix

Value 0.92 0.36

(%) 71.93 28.07

Trace 1.28

Correlation matrix

1 1.0000

2 0.0248 1.0000

Covariances & correlations with approximate sampling errors

7 COVS T 1 1 0.920035 0.311777E-01

8 COVS T 1 2 0.142604E-01 0.208266E-01 corr 0.025 0.036

9 COVS T 2 2 0.359455 0.180036E-01

======== end of file ============================06-02-2015==========14:04====

======= Version 30-08-2013 ======================================= **KM** ====

Program WOMBAT: Summary of Pedigree Information

==============================================================================

Two traits analysis (RF x RFIsf)

Analysis type : "muv 2"

Data file : "DADOS.dat"

Pedigree file : "ReducedPedFile.dat"

Parameter file : "wombat.par"

No. of animal IDs in data file = = 1882

No. of animal IDs in total = = 3143

*****Pedigree Structure for random effect : 1 ****************************

Original no. of animals = 3143

No. of animals after pruning = 3058

... proportion (%) remaining = 97.3

No. of levels w/out records = 1176

No. of levels with records = 1882 100.0%

... 1 record(s) = 1056 56.1%

... 2 record(s) = 826 43.9%

No. of animals w/out offspring = 1471 48.1%

No. of animals with offspring = 1587 51.9%

... and records = 411 13.4%

No. of animals with unknown sire = 60

No. of animals with unknown dam = 147

No. of animals with both parents unknown = 59

No. of animals with records =

... and unknown sire = 0

... and unknown dam = 0

... and both parents unknown = 0

No. of sires = 307

... with progeny in the data = 114

... with records & progeny in data = 43

No. of dams = 1280

... with progeny in the data = 782

... with records & progeny in data = 368

No. of animals with known/unpruned grand-parents

... with paternal grandsire = 2934

... with paternal granddam = 2808

... with maternal grandsire = 2809

... with maternal granddam = 2720

Inbreeding coefficients for random effect 1 computed

No. of inbred animals = 2612

Average inbreeding coefficient = 2.5603 (in %)

... amongst inbred animals = 2.9975 (in %)

random effect no. = 1 NRM

no. of elements in NRM/GIN inverse 11684

log determinant = -2102.1598822553833

======== end of file ============================06-02-2015==========14:09====

======= Version 30-08-2013 ======================================= **KM** ====

Program WOMBAT: Summary of information from Set-up step

==============================================================================

Analysis type : "muv 2"

Data file : "DADOS.dat"

Pedigree file : "ReducedPedFile.dat"

Parameter file : "wombat.par"

No. of traits = 2

nrec mean sdev min. max.

1 "RF" 1817 5.08663 2.54341 0.00000 19.2000

2 "RFIsf" 891 -0.904938E-02 0.596324 -2.24260 4.91730

Numbers of individuals/records for pairs of traits

1 2

1 "RF" 1817 826

2 "RFIsf" 826 891

Covariables

1"RF" nrec mean sdev min. max.

1 "idv(2)" 1817 6.09191 3.03138 2.00000 17.0000

2 "idade(1)" 1817 469.100 100.703 306.000 609.000

2"RFIsf" nrec mean sdev min. max.

1 "idv(2)" 891 6.16611 3.04967 3.00000 17.0000

2 "idade(1)" 891 368.979 35.9742 267.000 511.000

Fixed effects

1 "RF" nlev

1 "gc" 51

2 "mn" 3

2 "RFIsf" nlev

1 "gc" 21

Random effects nlev

1 "animal" 3058 NRM

======== end of file ============================06-02-2015==========14:09====

======= Version 30-08-2013 ======================================= **KM** ====

Program WOMBAT: Estimates of covariance components

==============================================================================

Analysis type : "muv 2"

Data file : "DADOS.dat"

Pedigree file : "ReducedPedFile.dat"

Parameter file : "wombat.par"

No. of traits = 2 RF RFIsf

No. of records = 2708 1817 891

No. of parameters = 6

Maximum log L = -1767.686

-1/2 AIC & AICC = -1773.686 -1773.701

-1/2 BIC = -1791.308 "Penalty factor" = 3.937

Parameter estimates with approx. sampling erors

1 CHOL Z 1 1 0.235696 0.364971E-01

2 CHOL Z 1 2 -0.464981E-01 0.334535E-01

3 CHOL Z 2 2 -0.634470 0.417827E-01

4 CHOL A 1 1 0.260862E-03 0.805415E-01

5 CHOL A 1 2 0.897709E-01 0.476451E-01

6 CHOL A 2 2 -1.34095 0.183130

Convergence criteria for last 3 iterates

Change in log likelihood = 7.265876 0.021815 0.000018

Change in parameter vector = 0.072388 0.006880 0.000641

Norm of gradient vector = 175.3950 9.0609 0.1296

Newton decrement = -14.0403 -0.0439 -0.0000

***** Estimates of residual covariances ************************************

Order of fit = 2

Covariance matrix

1 1.6022

2 -0.58857E-01 0.28329

Eigenvalues of covariance matrix

Value 1.60 0.28

(%) 85.11 14.89

Trace 1.89

Matrix of correlations and variance ratios

1 0.6156

2 -0.0874 0.7874

Covariances & correlations with approximate sampling errors

1 COVS Z 1 1 1.60222 0.116953 vrat 0.616 0.053

2 COVS Z 1 2 -0.588569E-01 0.422342E-01 corr -0.087 0.063

3 COVS Z 2 2 0.283291 0.232876E-01 vrat 0.787 0.064

***** Estimates for RE 1 "animal" ***************************************

No. of levels = 3058

Covariance structure = NRM

Order of fit = 2

Covariance matrix

1 1.0005

2 0.89794E-01 0.76492E-01

Eigenvalues of covariance matrix

Value 1.01 0.07

(%) 93.70 6.30

Trace 1.08

Matrix of correlations and variance ratios

1 0.3844

2 0.3246 0.2126

Covariances & correlations with approximate sampling errors

4 COVS A 1 1 1.00052 0.161167 vrat 0.384 0.053

5 COVS A 1 2 0.897944E-01 0.481548E-01 corr 0.325 0.175

6 COVS A 2 2 0.764922E-01 0.244758E-01 vrat 0.213 0.064

***** Estimates of phenotypic covariances ***********************************

Covariance matrix

1 2.6027

2 0.30937E-01 0.35978

Eigenvalues of covariance matrix

Value 2.60 0.36

(%) 87.87 12.13

Trace 2.96

Correlation matrix

1 1.0000

2 0.0320 1.0000

Covariances & correlations with approximate sampling errors

7 COVS T 1 1 2.60275 0.102928

8 COVS T 1 2 0.309375E-01 0.360536E-01 corr 0.032 0.037

9 COVS T 2 2 0.359784 0.180686E-01

======== end of file ============================06-02-2015==========14:09====

======= Version 30-08-2013 ======================================= **KM** ====

Program WOMBAT: Summary of Pedigree Information

==============================================================================

Two traits analysis (RG x DMI)

Analysis type : "muv 2"

Data file : "DADOS.dat"

Pedigree file : "ReducedPedFile.dat"

Parameter file : "wombat.par"

No. of animal IDs in data file = = 955

No. of animal IDs in total = = 2288

*****Pedigree Structure for random effect : 1 ****************************

Original no. of animals = 2288

No. of animals after pruning = 2204

... proportion (%) remaining = 96.3

No. of levels w/out records = 1249

No. of levels with records = 955 100.0%

... 2 record(s) = 955 100.0%

No. of animals w/out offspring = 828 37.6%

No. of animals with offspring = 1376 62.4%

... and records = 127 5.8%

No. of animals with unknown sire = 59

No. of animals with unknown dam = 145

No. of animals with both parents unknown = 58

No. of animals with records =

... and unknown sire = 0

... and unknown dam = 0

... and both parents unknown = 0

No. of sires = 296

... with progeny in the data = 78

... with records & progeny in data = 18

No. of dams = 1080

... with progeny in the data = 542

... with records & progeny in data = 98

No. of animals with known/unpruned grand-parents

... with paternal grandsire = 2083

... with paternal granddam = 1957

... with maternal grandsire = 1959

... with maternal granddam = 1872

random effect no. = 1 NRM

no. of elements in NRM/GIN inverse 8375

log determinant = -1487.8684515369519

======== end of file ============================25-02-2015==========17:59====

======= Version 30-08-2013 ======================================= **KM** ====

Program WOMBAT: Summary of information from Set-up step

==============================================================================

Analysis type : "muv 2"

Data file : "DADOS.dat"

Pedigree file : "ReducedPedFile.dat"

Parameter file : "wombat.par"

No. of traits = 2

nrec mean sdev min. max.

1 "RG" 955 0.236136E-02 0.122928 -0.707100 0.332300

2 "DMI" 955 6.83104 1.33500 2.15500 12.6400

Numbers of individuals/records for pairs of traits

1 2

1 "RG" 955 955

2 "DMI" 955 955

Covariables

1"RG" nrec mean sdev min. max.

1 "idv(2)" 955 6.15079 3.05304 3.00000 17.0000

2 "idade(1)" 955 369.980 37.3543 267.000 511.000

2"DMI" nrec mean sdev min. max.

1 "idv(2)" 955 6.15079 3.05304 3.00000 17.0000

2 "idade(1)" 955 369.980 37.3543 267.000 511.000

Fixed effects

1 "RG" nlev

1 "gc" 21

2 "DMI" nlev

1 "gc" 21

Random effects nlev

1 "animal" 2204 NRM

======== end of file ============================25-02-2015==========17:59====

======= Version 30-08-2013 ======================================= **KM** ====

Program WOMBAT: Estimates of covariance components

==============================================================================

Analysis type : "muv 2"

Data file : "DADOS.dat"

Pedigree file : "ReducedPedFile.dat"

Parameter file : "wombat.par"

No. of traits = 2 RG DMI

No. of records = 1910 955 955

No. of parameters = 6

Maximum log L = 1117.814

-1/2 AIC & AICC = 1111.814 1111.792

-1/2 BIC = 1095.226 "Penalty factor" = 3.765

Parameter estimates with approx. sampling erors

1 CHOL Z 1 1 -0.437397 0.567004E-01

2 CHOL Z 1 2 -0.285221E-01 0.712056E-02

3 CHOL Z 2 2 -2.25318 0.430944E-01

4 CHOL A 1 1 -0.505409 0.883630E-01

5 CHOL A 1 2 0.114820E-01 0.902898E-02

6 CHOL A 2 2 -2.97332 0.193994

Convergence criteria for last 3 iterates

Change in log likelihood = 0.040676 0.002578 0.000183

Change in parameter vector = 0.004194 0.001158 0.000307

Norm of gradient vector = 36.1749 7.8404 2.0665

Newton decrement = -0.1065 -0.0070 -0.0005

***** Estimates of residual covariances ************************************

Order of fit = 2

Covariance matrix

1 0.11852E-01

2 -0.18417E-01 0.41695

Eigenvalues of covariance matrix

Value 0.42 0.01

(%) 97.43 2.57

Trace 0.43

Matrix of correlations and variance ratios

1 0.8119

2 -0.2620 0.5340

Covariances & correlations with approximate sampling errors

1 COVS Z 1 1 0.118520E-01 0.939570E-03 vrat 0.812 0.064

2 COVS Z 1 2 -0.184171E-01 0.463629E-02 corr -0.262 0.064

3 COVS Z 2 2 0.416948 0.472822E-01 vrat 0.534 0.068

***** Estimates for RE 1 "animal" ***************************************

No. of levels = 2204

Covariance structure = NRM

Order of fit = 2

Covariance matrix

1 0.27464E-02

2 0.69266E-02 0.36392

Eigenvalues of covariance matrix

Value 0.36 0.00

(%) 99.29 0.71

Trace 0.37

Matrix of correlations and variance ratios

1 0.1881

2 0.2191 0.4660

Covariances & correlations with approximate sampling errors

4 COVS A 1 1 0.274645E-02 0.980296E-03 vrat 0.188 0.064

5 COVS A 1 2 0.692659E-02 0.541152E-02 corr 0.219 0.179

6 COVS A 2 2 0.363921 0.643143E-01 vrat 0.466 0.068

***** Estimates of phenotypic covariances ***********************************

Covariance matrix

1 0.14598E-01

2 -0.11491E-01 0.78087

Eigenvalues of covariance matrix

Value 0.78 0.01

(%) 98.19 1.81

Trace 0.80

Correlation matrix

1 1.0000

2 -0.1076 1.0000

Covariances & correlations with approximate sampling errors

7 COVS T 1 1 0.145985E-01 0.707917E-03

8 COVS T 1 2 -0.114906E-01 0.374917E-02 corr -0.108 0.035

9 COVS T 2 2 0.780869 0.408278E-01

======== end of file ============================25-02-2015==========17:59====

======= Version 30-08-2013 ======================================= **KM** ====

Program WOMBAT: Summary of Pedigree Information

==============================================================================

Two traits analysis (RG x ADG)

Analysis type : "muv 2"

Data file : "DADOS.dat"

Pedigree file : "ReducedPedFile.dat"

Parameter file : "wombat.par"

No. of animal IDs in data file = = 955

No. of animal IDs in total = = 2288

*****Pedigree Structure for random effect : 1 ****************************

Original no. of animals = 2288

No. of animals after pruning = 2204

... proportion (%) remaining = 96.3

No. of levels w/out records = 1249

No. of levels with records = 955 100.0%

... 2 record(s) = 955 100.0%

No. of animals w/out offspring = 828 37.6%

No. of animals with offspring = 1376 62.4%

... and records = 127 5.8%

No. of animals with unknown sire = 59

No. of animals with unknown dam = 145

No. of animals with both parents unknown = 58

No. of animals with records =

... and unknown sire = 0

... and unknown dam = 0

... and both parents unknown = 0

No. of sires = 296

... with progeny in the data = 78

... with records & progeny in data = 18

No. of dams = 1080

... with progeny in the data = 542

... with records & progeny in data = 98

No. of animals with known/unpruned grand-parents

... with paternal grandsire = 2083

... with paternal granddam = 1957

... with maternal grandsire = 1959

... with maternal granddam = 1872

random effect no. = 1 NRM

no. of elements in NRM/GIN inverse 8375

log determinant = -1487.8684515369519

======== end of file ============================25-02-2015==========18:01====

======= Version 30-08-2013 ======================================= **KM** ====

Program WOMBAT: Summary of information from Set-up step

==============================================================================

Analysis type : "muv 2"

Data file : "DADOS.dat"

Pedigree file : "ReducedPedFile.dat"

Parameter file : "wombat.par"

No. of traits = 2

nrec mean sdev min. max.

1 "RG" 955 0.236136E-02 0.122928 -0.707100 0.332300

2 "ADG" 955 1.00846 0.260202 0.176000 1.71800

Numbers of individuals/records for pairs of traits

1 2

1 "RG" 955 955

2 "ADG" 955 955

Covariables

1"RG" nrec mean sdev min. max.

1 "idv(2)" 955 6.15079 3.05304 3.00000 17.0000

2 "idade(1)" 955 369.980 37.3543 267.000 511.000

2"ADG" nrec mean sdev min. max.

1 "idv(2)" 955 6.15079 3.05304 3.00000 17.0000

2 "idade(1)" 955 369.980 37.3543 267.000 511.000

Fixed effects

1 "RG" nlev

1 "gc" 21

2 "ADG" nlev

1 "gc" 21

Random effects nlev

1 "animal" 2204 NRM

======== end of file ============================25-02-2015==========18:01====

======= Version 30-08-2013 ======================================= **KM** ====

Program WOMBAT: Estimates of covariance components

==============================================================================

Analysis type : "muv 2"

Data file : "DADOS.dat"

Pedigree file : "ReducedPedFile.dat"

Parameter file : "wombat.par"

No. of traits = 2 RG ADG

No. of records = 1910 955 955

No. of parameters = 6

Maximum log L = 3017.700

-1/2 AIC & AICC = 3011.700 3011.678

-1/2 BIC = 2995.112 "Penalty factor" = 3.765

Parameter estimates with approx. sampling erors

1 CHOL Z 1 1 -2.21566 0.395521E-01

2 CHOL Z 1 2 0.875217E-01 0.669658E-02

3 CHOL Z 2 2 -2.49920 0.588629E-01

4 CHOL A 1 1 -2.95873 0.181580

5 CHOL A 1 2 0.666340E-01 0.143645E-01

6 CHOL A 2 2 -2.58529 0.102573

Convergence criteria for last 3 iterates

Change in log likelihood = 0.047996 0.002832 0.000212

Change in parameter vector = 0.004312 0.000985 0.000307

Norm of gradient vector = 27.4791 6.7227 1.5183

Newton decrement = -0.1254 -0.0077 -0.0006

***** Estimates of residual covariances ************************************

Order of fit = 2

Covariance matrix

1 0.11899E-01

2 0.95470E-02 0.14409E-01

Eigenvalues of covariance matrix

Value 0.02 0.00

(%) 86.60 13.40

Trace 0.03

Matrix of correlations and variance ratios

1 0.8155

2 0.7291 0.5874

Covariances & correlations with approximate sampling errors

1 COVS Z 1 1 0.118988E-01 0.941245E-03 vrat 0.816 0.064

2 COVS Z 1 2 0.954702E-02 0.100091E-02 corr 0.729 0.031

3 COVS Z 2 2 0.144088E-01 0.148291E-02 vrat 0.587 0.067

***** Estimates for RE 1 "animal" ***************************************

No. of levels = 2204

Covariance structure = NRM

Order of fit = 2

Covariance matrix

1 0.26920E-02

2 0.34573E-02 0.10121E-01

Eigenvalues of covariance matrix

Value 0.01 0.00

(%) 89.60 10.40

Trace 0.01

Matrix of correlations and variance ratios

1 0.1845

2 0.6623 0.4126

Covariances & correlations with approximate sampling errors

4 COVS A 1 1 0.269200E-02 0.977626E-03 vrat 0.184 0.064

5 COVS A 1 2 0.345727E-02 0.113560E-02 corr 0.662 0.099

6 COVS A 2 2 0.101214E-01 0.191952E-02 vrat 0.413 0.067

***** Estimates of phenotypic covariances ***********************************

Covariance matrix

1 0.14591E-01

2 0.13004E-01 0.24530E-01

Eigenvalues of covariance matrix

Value 0.03 0.01

(%) 85.59 14.41

Trace 0.04

Correlation matrix

1 1.0000

2 0.6874 1.0000

Covariances & correlations with approximate sampling errors

7 COVS T 1 1 0.145908E-01 0.706813E-03

8 COVS T 1 2 0.130043E-01 0.796194E-03 corr 0.687 0.018

9 COVS T 2 2 0.245302E-01 0.125713E-02

======== end of file ============================25-02-2015==========18:01====

======= Version 30-08-2013 ======================================= **KM** ====

Program WOMBAT: Summary of Pedigree Information

==============================================================================

Two traits analysis (WS x RG)

Analysis type : "muv 2"

Data file : "DADOS.dat"

Pedigree file : "ReducedPedFile.dat"

Parameter file : "wombat.par"

No. of animal IDs in data file = = 8091

No. of animal IDs in total = = 8490

*****Pedigree Structure for random effect : 1 ****************************

Original no. of animals = 8490

No. of animals after pruning = 8433

... proportion (%) remaining = 99.3

No. of levels w/out records = 342

No. of levels with records = 8091 100.0%

... 1 record(s) = 7149 88.4%

... 2 record(s) = 942 11.6%

No. of animals w/out offspring = 6052 71.8%

No. of animals with offspring = 2381 28.2%

... and records = 2039 24.2%

No. of animals with unknown sire = 313

No. of animals with unknown dam = 384

No. of animals with both parents unknown = 312

No. of animals with records =

... and unknown sire = 1

... and unknown dam = 53

... and both parents unknown = 0

No. of sires = 325

... with progeny in the data = 320

... with records & progeny in data = 288

No. of dams = 2056

... with progeny in the data = 2056

... with records & progeny in data = 1751

No. of animals with known/unpruned grand-parents

... with paternal grandsire = 7358

... with paternal granddam = 7056

... with maternal grandsire = 6535

... with maternal granddam = 6468

random effect no. = 1 NRM

no. of elements in NRM/GIN inverse 31838

log determinant = -5688.7609053187680

random effect no. = 2 IDE

no. of elements in NRM/GIN inverse 0

log determinant = 0.0000000000000000

======== end of file ============================06-02-2015==========14:22====

======= Version 30-08-2013 ======================================= **KM** ====

Program WOMBAT: Summary of information from Set-up step

==============================================================================

Analysis type : "muv 2"

Data file : "DADOS.dat"

Pedigree file : "ReducedPedFile.dat"

Parameter file : "wombat.par"

No. of traits = 2

nrec mean sdev min. max.

1 "WS" 8078 299.703 49.6462 160.030 489.760

2 "RG" 955 0.236136E-02 0.122928 -0.707100 0.332300

Numbers of individuals/records for pairs of traits

1 2

1 "WS" 8078 942

2 "RG" 942 955

Covariables

1"WS" nrec mean sdev min. max.

1 "idv(2)" 8078 6.63568 3.07616 2.00000 18.0000

2 "idade(1)" 8078 473.414 105.028 293.000 645.000

2"RG" nrec mean sdev min. max.

1 "idv(2)" 955 6.15079 3.05304 3.00000 17.0000

2 "idade(1)" 955 369.980 37.3543 267.000 511.000

Fixed effects

1 "WS" nlev

1 "gc" 201

2 "mn" 4

2 "RG" nlev

1 "gc" 21

Random effects nlev

1 "animal" 8433 NRM

2 "peanim" 2107 IDE

======== end of file ============================06-02-2015==========14:22====

======= Version 30-08-2013 ======================================= **KM** ====

Program WOMBAT: Estimates of covariance components

==============================================================================

Analysis type : "muv 2"

Data file : "DADOS.dat"

Pedigree file : "ReducedPedFile.dat"

Parameter file : "wombat.par"

No. of traits = 2 WS RG

No. of records = 9033 8078 955

No. of parameters = 7

Maximum log L = -28683.508

-1/2 AIC & AICC = -28690.508 -28690.515

-1/2 BIC = -28715.298 "Penalty factor" = 4.541

Parameter estimates with approx. sampling erors

1 CHOL Z 1 1 3.02624 0.210827E-01

2 CHOL Z 1 2 0.141000E-01 0.585269E-02

3 CHOL Z 2 2 -2.22479 0.388026E-01

4 CHOL A 1 1 2.87554 0.484870E-01

5 CHOL A 1 2 0.177541E-01 0.750945E-02

6 CHOL A 2 2 -3.04152 0.202952

7 CHOL B 1 1 2.34903 0.493830E-01

Convergence criteria for last 3 iterates

Change in log likelihood = 0.063991 0.000819 0.000021

Change in parameter vector = 0.002169 0.000254 0.000030

Norm of gradient vector = 44.8489 4.5711 0.9522

Newton decrement = -0.1236 -0.0017 -0.0000

***** Estimates of residual covariances ************************************

Order of fit = 2

Covariance matrix

1 425.17

2 0.29074 0.11882E-01

Eigenvalues of covariance matrix

Value 425.17 0.01

(%) 100.00 0.00

Trace 425.18

Matrix of correlations and variance ratios

1 0.5005

2 0.1294 0.8207

Covariances & correlations with approximate sampling errors

1 COVS Z 1 1 425.165 17.9273 vrat 0.501 0.027

2 COVS Z 1 2 0.290735 0.121176 corr 0.129 0.053

3 COVS Z 2 2 0.118822E-01 0.923841E-03 vrat 0.821 0.063

***** Estimates for RE 1 "animal" ***************************************

No. of levels = 8433

Covariance structure = NRM

Order of fit = 2

Covariance matrix

1 314.53

2 0.31487 0.25964E-02

Eigenvalues of covariance matrix

Value 314.53 0.00

(%) 100.00 0.00

Trace 314.53

Matrix of correlations and variance ratios

1 0.3703

2 0.3484 0.1793

Covariances & correlations with approximate sampling errors

4 COVS A 1 1 314.528 30.5011 vrat 0.370 0.030

5 COVS A 1 2 0.314867 0.134746 corr 0.348 0.147

6 COVS A 2 2 0.259642E-02 0.948064E-03 vrat 0.179 0.063

***** Estimates for RE 2 "peanim" ***************************************

No. of levels = 2107

Covariance structure = IDE

Order of fit = 1

Covariance matrix

1 109.73

Matrix of correlations and variance ratios

1 0.1292

Covariances & correlations with approximate sampling errors

7 COVS B 1 1 109.733 10.8379 vrat 0.129 0.012

***** Estimates of phenotypic covariances ***********************************

Covariance matrix

1 849.43

2 0.60560 0.14479E-01

Eigenvalues of covariance matrix

Value 849.43 0.01

(%) 100.00 0.00

Trace 849.44

Correlation matrix

1 1.0000

2 0.1727 1.0000

Covariances & correlations with approximate sampling errors

8 COVS T 1 1 849.427 18.5506

9 COVS T 1 2 0.605602 0.112128 corr 0.173 0.031

10 COVS T 2 2 0.144787E-01 0.697077E-03

======== end of file ============================06-02-2015==========14:22====

======= Version 30-08-2013 ======================================= **KM** ====

Program WOMBAT: Summary of Pedigree Information

==============================================================================

Two traits analysis (HH x RG)

Analysis type : "muv 2"

Data file : "DADOS.dat"

Pedigree file : "ReducedPedFile.dat"

Parameter file : "wombat.par"

No. of animal IDs in data file = = 6560

No. of animal IDs in total = = 7100

*****Pedigree Structure for random effect : 1 ****************************

Original no. of animals = 7100

No. of animals after pruning = 7017

... proportion (%) remaining = 98.8

No. of levels w/out records = 457

No. of levels with records = 6560 100.0%

... 1 record(s) = 5617 85.6%

... 2 record(s) = 943 14.4%

No. of animals w/out offspring = 4829 68.8%

No. of animals with offspring = 2188 31.2%

... and records = 1731 24.7%

No. of animals with unknown sire = 148

No. of animals with unknown dam = 237

No. of animals with both parents unknown = 148

No. of animals with records =

... and unknown sire = 0

... and unknown dam = 18

... and both parents unknown = 0

No. of sires = 320

... with progeny in the data = 283

... with records & progeny in data = 240

No. of dams = 1868

... with progeny in the data = 1837

... with records & progeny in data = 1491

No. of animals with known/unpruned grand-parents

... with paternal grandsire = 6738

... with paternal granddam = 6484

... with maternal grandsire = 6339

... with maternal granddam = 6111

Inbreeding coefficients for random effect 1 computed

No. of inbred animals = 5149

Average inbreeding coefficient = 1.8504 (in %)

... amongst inbred animals = 2.5218 (in %)

random effect no. = 1 NRM

no. of elements in NRM/GIN inverse 26822

log determinant = -4814.8039049931958

======== end of file ============================06-02-2015==========14:25====

======= Version 30-08-2013 ======================================= **KM** ====

Program WOMBAT: Summary of information from Set-up step

==============================================================================

Analysis type : "muv 2"

Data file : "DADOS.dat"

Pedigree file : "ReducedPedFile.dat"

Parameter file : "wombat.par"

No. of traits = 2

nrec mean sdev min. max.

1 "HH" 6548 132.269 5.43028 100.000 149.000

2 "RG" 955 0.236136E-02 0.122928 -0.707100 0.332300

Numbers of individuals/records for pairs of traits

1 2

1 "HH" 6548 943

2 "RG" 943 955

Covariables

1"HH" nrec mean sdev min. max.

1 "idv(2)" 6548 6.40043 2.93455 2.00000 17.0000

2 "idade(1)" 6548 472.701 107.333 293.000 645.000

2"RG" nrec mean sdev min. max.

1 "idv(2)" 955 6.15079 3.05304 3.00000 17.0000

2 "idade(1)" 955 369.980 37.3543 267.000 511.000

Fixed effects

1 "HH" nlev

1 "gc" 169

2 "mn" 4

2 "RG" nlev

1 "gc" 21

Random effects nlev

1 "animal" 7017 NRM

======== end of file ============================06-02-2015==========14:25====

======= Version 30-08-2013 ======================================= **KM** ====

Program WOMBAT: Estimates of covariance components

==============================================================================

Analysis type : "muv 2"

Data file : "DADOS.dat"

Pedigree file : "ReducedPedFile.dat"

Parameter file : "wombat.par"

No. of traits = 2 HH RG

No. of records = 7503 6548 955

No. of parameters = 6

Maximum log L = -9947.439

-1/2 AIC & AICC = -9953.439 -9953.445

-1/2 BIC = -9974.128 "Penalty factor" = 4.448

Parameter estimates with approx. sampling erors

1 CHOL Z 1 1 0.895004 0.267575E-01

2 CHOL Z 1 2 -0.176533E-01 0.741928E-02

3 CHOL Z 2 2 -2.23501 0.425396E-01

4 CHOL A 1 1 1.12277 0.315417E-01

5 CHOL A 1 2 0.134642E-01 0.763932E-02

6 CHOL A 2 2 -2.95503 0.184386

Convergence criteria for last 3 iterates

Change in log likelihood = 0.089101 0.001535 0.000038

Change in parameter vector = 0.004250 0.000512 0.000080

Norm of gradient vector = 21.9806 1.5575 0.2428

Newton decrement = -0.1613 -0.0027 -0.0001

***** Estimates of residual covariances ************************************

Order of fit = 2

Covariance matrix

1 5.9895

2 -0.43204E-01 0.11759E-01

Eigenvalues of covariance matrix

Value 5.99 0.01

(%) 99.81 0.19

Trace 6.00

Matrix of correlations and variance ratios

1 0.3880

2 -0.1628 0.8025

Covariances & correlations with approximate sampling errors

1 COVS Z 1 1 5.98950 0.320528 vrat 0.388 0.026

2 COVS Z 1 2 -0.432036E-01 0.180961E-01 corr -0.163 0.069

3 COVS Z 2 2 0.117588E-01 0.945390E-03 vrat 0.803 0.065

***** Estimates for RE 1 "animal" ***************************************

No. of levels = 7017

Covariance structure = NRM

Order of fit = 2

Covariance matrix

1 9.4455

2 0.41380E-01 0.28933E-02

Eigenvalues of covariance matrix

Value 9.45 0.00

(%) 99.97 0.03

Trace 9.45

Matrix of correlations and variance ratios

1 0.6120

2 0.2503 0.1975

Covariances & correlations with approximate sampling errors

4 COVS A 1 1 9.44555 0.595857 vrat 0.612 0.026

5 COVS A 1 2 0.413802E-01 0.235444E-01 corr 0.250 0.144

6 COVS A 2 2 0.289333E-02 0.100149E-02 vrat 0.197 0.065

***** Estimates of phenotypic covariances ***********************************

Covariance matrix

1 15.435

2 -0.18234E-02 0.14652E-01

Eigenvalues of covariance matrix

Value 15.44 0.01

(%) 99.91 0.09

Trace 15.45

Correlation matrix

1 1.0000

2 -0.0038 1.0000

Covariances & correlations with approximate sampling errors

7 COVS T 1 1 15.4350 0.383872

8 COVS T 1 2 -0.182342E-02 0.171395E-01 corr -0.004 0.036

9 COVS T 2 2 0.146521E-01 0.713844E-03

======== end of file ============================06-02-2015==========14:25====

======= Version 30-08-2013 ======================================= **KM** ====

Program WOMBAT: Summary of Pedigree Information

==============================================================================

Two traits analysis (CC x RG)

Analysis type : "muv 2"

Data file : "DADOS.dat"

Pedigree file : "ReducedPedFile.dat"

Parameter file : "wombat.par"

No. of animal IDs in data file = = 3943

No. of animal IDs in total = = 5418

*****Pedigree Structure for random effect : 1 ****************************

Original no. of animals = 5418

No. of animals after pruning = 5330

... proportion (%) remaining = 98.4

No. of levels w/out records = 1387

No. of levels with records = 3943 100.0%

... 1 record(s) = 3055 77.5%

... 2 record(s) = 888 22.5%

No. of animals w/out offspring = 3311 62.1%

No. of animals with offspring = 2019 37.9%

... and records = 632 11.9%

No. of animals with unknown sire = 94

No. of animals with unknown dam = 188

No. of animals with both parents unknown = 94

No. of animals with records =

... and unknown sire = 0

... and unknown dam = 0

... and both parents unknown = 0

No. of sires = 318

... with progeny in the data = 255

... with records & progeny in data = 217

No. of dams = 1701

... with progeny in the data = 1508

... with records & progeny in data = 412

No. of animals with known/unpruned grand-parents

... with paternal grandsire = 5142

... with paternal granddam = 4954

... with maternal grandsire = 4953

... with maternal granddam = 4738

Inbreeding coefficients for random effect 1 computed

No. of inbred animals = 4324

Average inbreeding coefficient = 2.1293 (in %)

... amongst inbred animals = 2.6247 (in %)

random effect no. = 1 NRM

no. of elements in NRM/GIN inverse 20464

log determinant = -3671.8274443710275

======== end of file ============================06-02-2015==========14:26====

======= Version 30-08-2013 ======================================= **KM** ====

Program WOMBAT: Summary of information from Set-up step

==============================================================================

Analysis type : "muv 2"

Data file : "DADOS.dat"

Pedigree file : "ReducedPedFile.dat"

Parameter file : "wombat.par"

No. of traits = 2

nrec mean sdev min. max.

1 "CC" 3876 164.140 8.72028 128.000 192.000

2 "RG" 955 0.236136E-02 0.122928 -0.707100 0.332300

Numbers of individuals/records for pairs of traits

1 2

1 "CC" 3876 888

2 "RG" 888 955

Covariables

1"CC" nrec mean sdev min. max.

1 "idv(2)" 3876 6.27632 2.87660 2.00000 17.0000

2 "idade(1)" 3876 431.403 109.187 293.000 725.000

2"RG" nrec mean sdev min. max.

1 "idv(2)" 955 6.15079 3.05304 3.00000 17.0000

2 "idade(1)" 955 369.980 37.3543 267.000 511.000

Fixed effects

1 "CC" nlev

1 "gc" 100

2 "mn" 4

2 "RG" nlev

1 "gc" 21

Random effects nlev

1 "animal" 5330 NRM

======== end of file ============================06-02-2015==========14:26====

======= Version 30-08-2013 ======================================= **KM** ====

Program WOMBAT: Estimates of covariance components

==============================================================================

Analysis type : "muv 2"

Data file : "DADOS.dat"

Pedigree file : "ReducedPedFile.dat"

Parameter file : "wombat.par"

No. of traits = 2 CC RG

No. of records = 4831 3876 955

No. of parameters = 6

Maximum log L = -7239.207

-1/2 AIC & AICC = -7245.207 -7245.216

-1/2 BIC = -7264.574 "Penalty factor" = 4.228

Parameter estimates with approx. sampling erors

1 CHOL Z 1 1 1.61725 0.234275E-01

2 CHOL Z 1 2 0.142107E-02 0.596809E-02

3 CHOL Z 2 2 -2.21720 0.393412E-01

4 CHOL A 1 1 1.18579 0.728738E-01

5 CHOL A 1 2 0.183640E-01 0.965118E-02

6 CHOL A 2 2 -3.01102 0.201830

Convergence criteria for last 3 iterates

Change in log likelihood = 0.021920 0.000610 0.000022

Change in parameter vector = 0.003189 0.000579 0.000110

Norm of gradient vector = 6.7318 0.6311 0.1295

Newton decrement = -0.0385 -0.0010 -0.0000

***** Estimates of residual covariances ************************************

Order of fit = 2

Covariance matrix

1 25.394

2 0.71611E-02 0.11864E-01

Eigenvalues of covariance matrix

Value 25.39 0.01

(%) 99.95 0.05

Trace 25.41

Matrix of correlations and variance ratios

1 0.7033

2 0.0130 0.8112

Covariances & correlations with approximate sampling errors

1 COVS Z 1 1 25.3938 1.18983 vrat 0.703 0.039

2 COVS Z 1 2 0.716108E-02 0.300872E-01 corr 0.013 0.055

3 COVS Z 2 2 0.118641E-01 0.934629E-03 vrat 0.811 0.063

***** Estimates for RE 1 "animal" ***************************************

No. of levels = 5330

Covariance structure = NRM

Order of fit = 2

Covariance matrix

1 10.714

2 0.60110E-01 0.27619E-02

Eigenvalues of covariance matrix

Value 10.71 0.00

(%) 99.98 0.02

Trace 10.72

Matrix of correlations and variance ratios

1 0.2967

2 0.3494 0.1888

Covariances & correlations with approximate sampling errors

4 COVS A 1 1 10.7143 1.56158 vrat 0.297 0.039

5 COVS A 1 2 0.601103E-01 0.320123E-01 corr 0.349 0.184

6 COVS A 2 2 0.276194E-02 0.969860E-03 vrat 0.189 0.063

***** Estimates of phenotypic covariances ***********************************

Covariance matrix

1 36.108

2 0.67271E-01 0.14626E-01

Eigenvalues of covariance matrix

Value 36.11 0.01

(%) 99.96 0.04

Trace 36.12

Correlation matrix

1 1.0000

2 0.0926 1.0000

Covariances & correlations with approximate sampling errors

7 COVS T 1 1 36.1080 0.973409

8 COVS T 1 2 0.672714E-01 0.258411E-01 corr 0.093 0.035

9 COVS T 2 2 0.146261E-01 0.708440E-03

======== end of file ============================06-02-2015==========14:26====

======= Version 30-08-2013 ======================================= **KM** ====

Program WOMBAT: Summary of Pedigree Information

==============================================================================

Two traits analysis (LEA x RG)

Analysis type : "muv 2"

Data file : "DADOS.dat"

Pedigree file : "ReducedPedFile.dat"

Parameter file : "wombat.par"

No. of animal IDs in data file = = 2396

No. of animal IDs in total = = 3756

*****Pedigree Structure for random effect : 1 ****************************

Original no. of animals = 3756

No. of animals after pruning = 3665

... proportion (%) remaining = 97.6

No. of levels w/out records = 1269

No. of levels with records = 2396 100.0%

... 1 record(s) = 1554 64.9%

... 2 record(s) = 842 35.1%

No. of animals w/out offspring = 1913 52.2%

No. of animals with offspring = 1752 47.8%

... and records = 483 13.2%

No. of animals with unknown sire = 64

No. of animals with unknown dam = 161

No. of animals with both parents unknown = 64

No. of animals with records =

... and unknown sire = 0

... and unknown dam = 0

... and both parents unknown = 0

No. of sires = 313

... with progeny in the data = 180

... with records & progeny in data = 85

No. of dams = 1439

... with progeny in the data = 1042

... with records & progeny in data = 392

No. of animals with known/unpruned grand-parents

... with paternal grandsire = 3520

... with paternal granddam = 3382

... with maternal grandsire = 3391

... with maternal granddam = 3267

Inbreeding coefficients for random effect 1 computed

No. of inbred animals = 3134

Average inbreeding coefficient = 2.3793 (in %)

... amongst inbred animals = 2.7824 (in %)

random effect no. = 1 NRM

no. of elements in NRM/GIN inverse 14038

log determinant = -2522.5521618264124

======== end of file ============================06-02-2015==========14:30====

======= Version 30-08-2013 ======================================= **KM** ====

Program WOMBAT: Summary of information from Set-up step

==============================================================================

Analysis type : "muv 2"

Data file : "DADOS.dat"

Pedigree file : "ReducedPedFile.dat"

Parameter file : "wombat.par"

No. of traits = 2

nrec mean sdev min. max.

1 "aol" 2283 51.4305 8.89390 21.4000 83.4000

2 "GANR" 955 0.236136E-02 0.122928 -0.707100 0.332300

Numbers of individuals/records for pairs of traits

1 2

1 "aol" 2283 842

2 "GANR" 842 955

Covariables

1"aol" nrec mean sdev min. max.

1 "idv(2)" 2283 6.11082 2.92852 2.00000 17.0000

2 "idade(1)" 2283 448.951 98.8881 306.000 609.000

2"GANR" nrec mean sdev min. max.

1 "idv(2)" 955 6.15079 3.05304 3.00000 17.0000

2 "idade(1)" 955 369.980 37.3543 267.000 511.000

Fixed effects

1 "aol" nlev

1 "gc" 65

2 "mn" 4

2 "GANR" nlev

1 "gc" 21

Random effects nlev

1 "animal" 3665 NRM

======== end of file ============================06-02-2015==========14:30====

======= Version 30-08-2013 ======================================= **KM** ====

Program WOMBAT: Estimates of covariance components

==============================================================================

Analysis type : "muv 2"

Data file : "DADOS.dat"

Pedigree file : "ReducedPedFile.dat"

Parameter file : "wombat.par"

No. of traits = 2 aol GANR

No. of records = 3238 2283 955

No. of parameters = 6

Maximum log L = -3614.754

-1/2 AIC & AICC = -3620.754 -3620.767

-1/2 BIC = -3638.913 "Penalty factor" = 4.026

Parameter estimates with approx. sampling erors

1 CHOL Z 1 1 1.49130 0.394997E-01

2 CHOL Z 1 2 0.148052E-02 0.735214E-02

3 CHOL Z 2 2 -2.22462 0.404153E-01

4 CHOL A 1 1 1.41587 0.678915E-01

5 CHOL A 1 2 0.514131E-02 0.967268E-02

6 CHOL A 2 2 -2.91197 0.171608

Convergence criteria for last 3 iterates

Change in log likelihood = 0.107030 0.001871 0.000039

Change in parameter vector = 0.016482 0.001050 0.000323

Norm of gradient vector = 12.8075 4.7965 0.2330

Newton decrement = -0.2180 -0.0037 -0.0001

***** Estimates of residual covariances ************************************

Order of fit = 2

Covariance matrix

1 19.739

2 0.65778E-02 0.11690E-01

Eigenvalues of covariance matrix

Value 19.74 0.01

(%) 99.94 0.06

Trace 19.75

Matrix of correlations and variance ratios

1 0.5376

2 0.0137 0.7967

Covariances & correlations with approximate sampling errors

1 COVS Z 1 1 19.7392 1.55938 vrat 0.538 0.051

2 COVS Z 1 2 0.657778E-02 0.326649E-01 corr 0.014 0.068

3 COVS Z 2 2 0.116896E-01 0.943987E-03 vrat 0.797 0.065

***** Estimates for RE 1 "animal" ***************************************

No. of levels = 3665

Covariance structure = NRM

Order of fit = 2

Covariance matrix

1 16.975

2 0.21183E-01 0.29824E-02

Eigenvalues of covariance matrix

Value 16.98 0.00

(%) 99.98 0.02

Trace 16.98

Matrix of correlations and variance ratios

1 0.4624

2 0.0941 0.2033

Covariances & correlations with approximate sampling errors

4 COVS A 1 1 16.9751 2.30493 vrat 0.462 0.051

5 COVS A 1 2 0.211827E-01 0.398654E-01 corr 0.094 0.178

6 COVS A 2 2 0.298238E-02 0.100573E-02 vrat 0.203 0.065

***** Estimates of phenotypic covariances ***********************************

Covariance matrix

1 36.714

2 0.27760E-01 0.14672E-01

Eigenvalues of covariance matrix

Value 36.71 0.01

(%) 99.96 0.04

Trace 36.73

Correlation matrix

1 1.0000

2 0.0378 1.0000

Covariances & correlations with approximate sampling errors

7 COVS T 1 1 36.7143 1.36671

8 COVS T 1 2 0.277604E-01 0.279128E-01 corr 0.038 0.038

9 COVS T 2 2 0.146720E-01 0.715743E-03

======== end of file ============================06-02-2015==========14:30====

======= Version 30-08-2013 ======================================= **KM** ====

Program WOMBAT: Summary of Pedigree Information

==============================================================================

Two traits analysis (BF x RG)

Analysis type : "muv 2"

Data file : "DADOS.dat"

Pedigree file : "ReducedPedFile.dat"

Parameter file : "wombat.par"

No. of animal IDs in data file = = 2396

No. of animal IDs in total = = 3756

*****Pedigree Structure for random effect : 1 ****************************

Original no. of animals = 3756

No. of animals after pruning = 3665

... proportion (%) remaining = 97.6

No. of levels w/out records = 1269

No. of levels with records = 2396 100.0%

... 1 record(s) = 1552 64.8%

... 2 record(s) = 844 35.2%

No. of animals w/out offspring = 1913 52.2%

No. of animals with offspring = 1752 47.8%

... and records = 483 13.2%

No. of animals with unknown sire = 64

No. of animals with unknown dam = 161

No. of animals with both parents unknown = 64

No. of animals with records =

... and unknown sire = 0

... and unknown dam = 0

... and both parents unknown = 0

No. of sires = 313

... with progeny in the data = 180

... with records & progeny in data = 85

No. of dams = 1439

... with progeny in the data = 1042

... with records & progeny in data = 392

No. of animals with known/unpruned grand-parents

... with paternal grandsire = 3520

... with paternal granddam = 3382

... with maternal grandsire = 3391

... with maternal granddam = 3267

random effect no. = 1 NRM

no. of elements in NRM/GIN inverse 14038

log determinant = -2522.5521618264124

======== end of file ============================06-02-2015==========14:36====

======= Version 30-08-2013 ======================================= **KM** ====

Program WOMBAT: Summary of information from Set-up step

==============================================================================

Analysis type : "muv 2"

Data file : "DADOS.dat"

Pedigree file : "ReducedPedFile.dat"

Parameter file : "wombat.par"

No. of traits = 2

nrec mean sdev min. max.

1 "BF" 2285 1.75922 1.42798 0.00000 10.5000

2 "RG" 955 0.236136E-02 0.122928 -0.707100 0.332300

Numbers of individuals/records for pairs of traits

1 2

1 "BF" 2285 844

2 "RG" 844 955

Covariables

1"BF" nrec mean sdev min. max.

1 "idv(2)" 2285 6.10810 2.92868 2.00000 17.0000

2 "idade(1)" 2285 449.081 98.9421 306.000 609.000

2"RG" nrec mean sdev min. max.

1 "idv(2)" 955 6.15079 3.05304 3.00000 17.0000

2 "idade(1)" 955 369.980 37.3543 267.000 511.000

Fixed effects

1 "BF" nlev

1 "gc" 65

2 "mn" 4

2 "RG" nlev

1 "gc" 21

Random effects nlev

1 "animal" 3665 NRM

======== end of file ============================06-02-2015==========14:36====

======= Version 30-08-2013 ======================================= **KM** ====

Program WOMBAT: Estimates of covariance components

==============================================================================

Analysis type : "muv 2"

Data file : "DADOS.dat"

Pedigree file : "ReducedPedFile.dat"

Parameter file : "wombat.par"

No. of traits = 2 BF RG

No. of records = 3240 2285 955

No. of parameters = 6

Maximum log L = 393.208

-1/2 AIC & AICC = 387.208 387.195

-1/2 BIC = 369.047 "Penalty factor" = 4.027

Parameter estimates with approx. sampling erors

1 CHOL Z 1 1 -0.225386 0.295212E-01

2 CHOL Z 1 2 0.887783E-03 0.619004E-02

3 CHOL Z 2 2 -2.22648 0.406055E-01

4 CHOL A 1 1 -0.626772 0.839737E-01

5 CHOL A 1 2 -0.939037E-02 0.983036E-02

6 CHOL A 2 2 -2.91367 0.172193

Convergence criteria for last 3 iterates

Change in log likelihood = 0.940424 0.007681 0.000033

Change in parameter vector = 0.023655 0.000774 0.000098

Norm of gradient vector = 187.2090 21.3285 1.6154

Newton decrement = -1.9434 -0.0162 -0.0001

***** Estimates of residual covariances ************************************

Order of fit = 2

Covariance matrix

1 0.63714

2 0.70864E-03 0.11645E-01

Eigenvalues of covariance matrix

Value 0.64 0.01

(%) 98.21 1.79

Trace 0.65

Matrix of correlations and variance ratios

1 0.6906

2 0.0082 0.7933

Covariances & correlations with approximate sampling errors

1 COVS Z 1 1 0.637136 0.376181E-01 vrat 0.691 0.046

2 COVS Z 1 2 0.708636E-03 0.494029E-02 corr 0.008 0.057

3 COVS Z 2 2 0.116448E-01 0.945189E-03 vrat 0.793 0.065

***** Estimates for RE 1 "animal" ***************************************

No. of levels = 3665

Covariance structure = NRM

Order of fit = 2

Covariance matrix

1 0.28549

2 -0.50174E-02 0.30341E-02

Eigenvalues of covariance matrix

Value 0.29 0.00

(%) 98.98 1.02

Trace 0.29

Matrix of correlations and variance ratios

1 0.3094

2 -0.1705 0.2067

Covariances & correlations with approximate sampling errors

4 COVS A 1 1 0.285491 0.479475E-01 vrat 0.309 0.046

5 COVS A 1 2 -0.501740E-02 0.526707E-02 corr -0.170 0.178

6 COVS A 2 2 0.303410E-02 0.101306E-02 vrat 0.207 0.065

***** Estimates of phenotypic covariances ***********************************

Covariance matrix

1 0.92263

2 -0.43088E-02 0.14679E-01

Eigenvalues of covariance matrix

Value 0.92 0.01

(%) 98.44 1.56

Trace 0.94

Correlation matrix

1 1.0000

2 -0.0370 1.0000

Covariances & correlations with approximate sampling errors

7 COVS T 1 1 0.922627 0.314300E-01

8 COVS T 1 2 -0.430876E-02 0.411730E-02 corr -0.037 0.035

9 COVS T 2 2 0.146789E-01 0.717229E-03

======== end of file ============================06-02-2015==========14:36====

======= Version 30-08-2013 ======================================= **KM** ====

Program WOMBAT: Summary of Pedigree Information

==============================================================================

Two traits analysis (RF x RG)

Analysis type : "muv 2"

Data file : "DADOS.dat"

Pedigree file : "ReducedPedFile.dat"

Parameter file : "wombat.par"

No. of animal IDs in data file = = 1930

No. of animal IDs in total = = 3174

*****Pedigree Structure for random effect : 1 ****************************

Original no. of animals = 3174

No. of animals after pruning = 3089

... proportion (%) remaining = 97.3

No. of levels w/out records = 1159

No. of levels with records = 1930 100.0%

... 1 record(s) = 1088 56.4%

... 2 record(s) = 842 43.6%

No. of animals w/out offspring = 1497 48.5%

No. of animals with offspring = 1592 51.5%

... and records = 433 14.0%

No. of animals with unknown sire = 60

No. of animals with unknown dam = 147

No. of animals with both parents unknown = 59

No. of animals with records =

... and unknown sire = 0

... and unknown dam = 0

... and both parents unknown = 0

No. of sires = 307

... with progeny in the data = 114

... with records & progeny in data = 44

No. of dams = 1285

... with progeny in the data = 792

... with records & progeny in data = 389

No. of animals with known/unpruned grand-parents

... with paternal grandsire = 2965

... with paternal granddam = 2839

... with maternal grandsire = 2840

... with maternal granddam = 2751

Inbreeding coefficients for random effect 1 computed

No. of inbred animals = 2643

Average inbreeding coefficient = 2.5643 (in %)

... amongst inbred animals = 2.9971 (in %)

random effect no. = 1 NRM

no. of elements in NRM/GIN inverse 11801

log determinant = -2124.3928089367182

======== end of file ============================06-02-2015==========14:39====

======= Version 30-08-2013 ======================================= **KM** ====

Program WOMBAT: Summary of information from Set-up step

==============================================================================

Analysis type : "muv 2"

Data file : "DADOS.dat"

Pedigree file : "ReducedPedFile.dat"

Parameter file : "wombat.par"

No. of traits = 2

nrec mean sdev min. max.

1 "RF" 1817 5.08663 2.54341 0.00000 19.2000

2 "RG" 955 0.236136E-02 0.122928 -0.707100 0.332300

Numbers of individuals/records for pairs of traits

1 2

1 "RF" 1817 842

2 "RG" 842 955

Covariables

1"RF" nrec mean sdev min. max.

1 "idv(2)" 1817 6.09191 3.03138 2.00000 17.0000

2 "idade(1)" 1817 469.100 100.703 306.000 609.000

2"RG" nrec mean sdev min. max.

1 "idv(2)" 955 6.15079 3.05304 3.00000 17.0000

2 "idade(1)" 955 369.980 37.3543 267.000 511.000

Fixed effects

1 "RF" nlev

1 "gc" 51

2 "mn" 3

2 "RG" nlev

1 "gc" 21

Random effects nlev

1 "animal" 3089 NRM

======== end of file ============================06-02-2015==========14:39====

======= Version 30-08-2013 ======================================= **KM** ====

Program WOMBAT: Estimates of covariance components

==============================================================================

Analysis type : "muv 2"

Data file : "DADOS.dat"

Pedigree file : "ReducedPedFile.dat"

Parameter file : "wombat.par"

No. of traits = 2 RF RG

No. of records = 2772 1817 955

No. of parameters = 6

Maximum log L = -278.340

-1/2 AIC & AICC = -284.340 -284.355

-1/2 BIC = -302.034 "Penalty factor" = 3.949

Parameter estimates with approx. sampling erors

1 CHOL Z 1 1 0.234626 0.365613E-01

2 CHOL Z 1 2 0.106545E-01 0.667752E-02

3 CHOL Z 2 2 -2.23108 0.413626E-01

4 CHOL A 1 1 0.284968E-02 0.802454E-01

5 CHOL A 1 2 -0.101371E-01 0.956869E-02

6 CHOL A 2 2 -2.91740 0.174946

Convergence criteria for last 3 iterates

Change in log likelihood = 10.165819 0.101694 0.000100

Change in parameter vector = 0.039856 0.009166 0.000257

Norm of gradient vector = 480.6216 15.9564 1.4481

Newton decrement = -19.5880 -0.2066 -0.0002

***** Estimates of residual covariances ************************************

Order of fit = 2

Covariance matrix

1 1.5988

2 0.13472E-01 0.11651E-01

Eigenvalues of covariance matrix

Value 1.60 0.01

(%) 99.28 0.72

Trace 1.61

Matrix of correlations and variance ratios

1 0.6139

2 0.0987 0.7938

Covariances & correlations with approximate sampling errors

1 COVS Z 1 1 1.59880 0.116908 vrat 0.614 0.053

2 COVS Z 1 2 0.134720E-01 0.843471E-02 corr 0.099 0.062

3 COVS Z 2 2 0.116510E-01 0.947458E-03 vrat 0.794 0.065

***** Estimates for RE 1 "animal" ***************************************

No. of levels = 3089

Covariance structure = NRM

Order of fit = 2

Covariance matrix

1 1.0057

2 -0.10166E-01 0.30268E-02

Eigenvalues of covariance matrix

Value 1.01 0.00

(%) 99.71 0.29

Trace 1.01

Matrix of correlations and variance ratios

1 0.3861

2 -0.1843 0.2062

Covariances & correlations with approximate sampling errors

4 COVS A 1 1 1.00572 0.161408 vrat 0.386 0.053

5 COVS A 1 2 -0.101660E-01 0.960539E-02 corr -0.184 0.175

6 COVS A 2 2 0.302678E-02 0.101587E-02 vrat 0.206 0.065

***** Estimates of phenotypic covariances ***********************************

Covariance matrix

1 2.6045

2 0.33059E-02 0.14678E-01

Eigenvalues of covariance matrix

Value 2.60 0.01

(%) 99.44 0.56

Trace 2.62

Correlation matrix

1 1.0000

2 0.0169 1.0000

Covariances & correlations with approximate sampling errors

7 COVS T 1 1 2.60451 0.103083

8 COVS T 1 2 0.330594E-02 0.714024E-02 corr 0.017 0.037

9 COVS T 2 2 0.146777E-01 0.717288E-03

======== end of file ============================06-02-2015==========14:39====

======= Version 30-08-2013 ======================================= **KM** ====

Program WOMBAT: Summary of Pedigree Information

==============================================================================

Two traits analysis (RIG x DMI)

Analysis type : "muv 2"

Data file : "DADOS.dat"

Pedigree file : "ReducedPedFile.dat"

Parameter file : "wombat.par"

No. of animal IDs in data file = = 955

No. of animal IDs in total = = 2288

*****Pedigree Structure for random effect : 1 ****************************

Original no. of animals = 2288

No. of animals after pruning = 2204

... proportion (%) remaining = 96.3

No. of levels w/out records = 1249

No. of levels with records = 955 100.0%

... 2 record(s) = 955 100.0%

No. of animals w/out offspring = 828 37.6%

No. of animals with offspring = 1376 62.4%

... and records = 127 5.8%

No. of animals with unknown sire = 59

No. of animals with unknown dam = 145

No. of animals with both parents unknown = 58

No. of animals with records =

... and unknown sire = 0

... and unknown dam = 0

... and both parents unknown = 0

No. of sires = 296

... with progeny in the data = 78

... with records & progeny in data = 18

No. of dams = 1080

... with progeny in the data = 542

... with records & progeny in data = 98

No. of animals with known/unpruned grand-parents

... with paternal grandsire = 2083

... with paternal granddam = 1957

... with maternal grandsire = 1959

... with maternal granddam = 1872

random effect no. = 1 NRM

no. of elements in NRM/GIN inverse 8375

log determinant = -1487.8684515369519

======== end of file ============================25-02-2015==========18:10====

======= Version 30-08-2013 ======================================= **KM** ====

Program WOMBAT: Summary of information from Set-up step

==============================================================================

Analysis type : "muv 2"

Data file : "DADOS.dat"

Pedigree file : "ReducedPedFile.dat"

Parameter file : "wombat.par"

No. of traits = 2

nrec mean sdev min. max.

1 "RIG" 955 0.443529E-01 1.71606 -5.93190 4.92270

2 "DMI" 955 6.83104 1.33500 2.15500 12.6400

Numbers of individuals/records for pairs of traits

1 2

1 "RIG" 955 955

2 "DMI" 955 955

Covariables

1"RIG" nrec mean sdev min. max.

1 "idv(2)" 955 6.15079 3.05304 3.00000 17.0000

2 "idade(1)" 955 369.980 37.3543 267.000 511.000

2"DMI" nrec mean sdev min. max.

1 "idv(2)" 955 6.15079 3.05304 3.00000 17.0000

2 "idade(1)" 955 369.980 37.3543 267.000 511.000

Fixed effects

1 "RIG" nlev

1 "gc" 21

2 "DMI" nlev

1 "gc" 21

Random effects nlev

1 "animal" 2204 NRM

======== end of file ============================25-02-2015==========18:10====

======= Version 30-08-2013 ======================================= **KM** ====

Program WOMBAT: Estimates of covariance components

==============================================================================

Analysis type : "muv 2"

Data file : "DADOS.dat"

Pedigree file : "ReducedPedFile.dat"

Parameter file : "wombat.par"

No. of traits = 2 RIG DMI

No. of records = 1910 955 955

No. of parameters = 6

Maximum log L = -1242.102

-1/2 AIC & AICC = -1248.102 -1248.125

-1/2 BIC = -1264.691 "Penalty factor" = 3.765

Parameter estimates with approx. sampling erors

1 CHOL Z 1 1 1.56069 0.606004E-01

2 CHOL Z 1 2 -0.358911 0.390261E-01

3 CHOL Z 2 2 0.536303 0.356598E-01

4 CHOL A 1 1 -0.387394 0.208539

5 CHOL A 1 2 -0.165269 0.104439

6 CHOL A 2 2 -0.543094 0.821563E-01

Convergence criteria for last 3 iterates

Change in log likelihood = 0.810162 0.001257 0.000031

Change in parameter vector = 0.042630 0.003642 0.000639

Norm of gradient vector = 40.5183 0.8225 0.0882

Newton decrement = -1.6054 -0.0028 -0.0001

***** Estimates of residual covariances ************************************

Order of fit = 2

Covariance matrix

1 2.4358

2 -0.56015 0.41644

Eigenvalues of covariance matrix

Value 2.58 0.27

(%) 90.48 9.52

Trace 2.85

Matrix of correlations and variance ratios

1 0.8409

2 -0.5562 0.5330

Covariances & correlations with approximate sampling errors

1 COVS Z 1 1 2.43576 0.189157 vrat 0.841 0.064

2 COVS Z 1 2 -0.560150 0.708340E-01 corr -0.556 0.049

3 COVS Z 2 2 0.416438 0.470241E-01 vrat 0.533 0.068

***** Estimates for RE 1 "animal" ***************************************

No. of levels = 2204

Covariance structure = NRM

Order of fit = 2

Covariance matrix

1 0.46080

2 -0.11219 0.36481

Eigenvalues of covariance matrix

Value 0.53 0.29

(%) 64.78 35.22

Trace 0.83

Matrix of correlations and variance ratios

1 0.1591

2 -0.2736 0.4670

Covariances & correlations with approximate sampling errors

4 COVS A 1 1 0.460802 0.192191 vrat 0.159 0.064

5 COVS A 1 2 -0.112189 0.795567E-01 corr -0.274 0.162

6 COVS A 2 2 0.364814 0.639632E-01 vrat 0.467 0.068

***** Estimates of phenotypic covariances ***********************************

Covariance matrix

1 2.8966

2 -0.67234 0.78125

Eigenvalues of covariance matrix

Value 3.09 0.59

(%) 84.08 15.92

Trace 3.68

Correlation matrix

1 1.0000

2 -0.4469 1.0000

Covariances & correlations with approximate sampling errors

7 COVS T 1 1 2.89657 0.139866

8 COVS T 1 2 -0.672338 0.567435E-01 corr -0.447 0.028

9 COVS T 2 2 0.781253 0.407972E-01

======== end of file ============================25-02-2015==========18:10====

======= Version 30-08-2013 ======================================= **KM** ====

Program WOMBAT: Summary of Pedigree Information

==============================================================================

Two traits analysis (RIG x ADG)

Analysis type : "muv 2"

Data file : "DADOS.dat"

Pedigree file : "ReducedPedFile.dat"

Parameter file : "wombat.par"

No. of animal IDs in data file = = 955

No. of animal IDs in total = = 2288

*****Pedigree Structure for random effect : 1 ****************************

Original no. of animals = 2288

No. of animals after pruning = 2204

... proportion (%) remaining = 96.3

No. of levels w/out records = 1249

No. of levels with records = 955 100.0%

... 2 record(s) = 955 100.0%

No. of animals w/out offspring = 828 37.6%

No. of animals with offspring = 1376 62.4%

... and records = 127 5.8%

No. of animals with unknown sire = 59

No. of animals with unknown dam = 145

No. of animals with both parents unknown = 58

No. of animals with records =

... and unknown sire = 0

... and unknown dam = 0

... and both parents unknown = 0

No. of sires = 296

... with progeny in the data = 78

... with records & progeny in data = 18

No. of dams = 1080

... with progeny in the data = 542

... with records & progeny in data = 98

No. of animals with known/unpruned grand-parents

... with paternal grandsire = 2083

... with paternal granddam = 1957

... with maternal grandsire = 1959

... with maternal granddam = 1872

random effect no. = 1 NRM

no. of elements in NRM/GIN inverse 8375

log determinant = -1487.8684515369519

======== end of file ============================25-02-2015==========18:12====

======= Version 30-08-2013 ======================================= **KM** ====

Program WOMBAT: Summary of information from Set-up step

==============================================================================

Analysis type : "muv 2"

Data file : "DADOS.dat"

Pedigree file : "ReducedPedFile.dat"

Parameter file : "wombat.par"

No. of traits = 2

nrec mean sdev min. max.

1 "RIG" 955 0.443529E-01 1.71606 -5.93190 4.92270

2 "ADG" 955 1.00846 0.260202 0.176000 1.71800

Numbers of individuals/records for pairs of traits

1 2

1 "RIG" 955 955

2 "ADG" 955 955

Covariables

1"RIG" nrec mean sdev min. max.

1 "idv(2)" 955 6.15079 3.05304 3.00000 17.0000

2 "idade(1)" 955 369.980 37.3543 267.000 511.000

2"ADG" nrec mean sdev min. max.

1 "idv(2)" 955 6.15079 3.05304 3.00000 17.0000

2 "idade(1)" 955 369.980 37.3543 267.000 511.000

Fixed effects

1 "RIG" nlev

1 "gc" 21

2 "ADG" nlev

1 "gc" 21

Random effects nlev

1 "animal" 2204 NRM

======== end of file ============================25-02-2015==========18:12====

======= Version 30-08-2013 ======================================= **KM** ====

Program WOMBAT: Estimates of covariance components

==============================================================================

Analysis type : "muv 2"

Data file : "DADOS.dat"

Pedigree file : "ReducedPedFile.dat"

Parameter file : "wombat.par"

No. of traits = 2 RIG ADG

No. of records = 1910 955 955

No. of parameters = 6

Maximum log L = 308.489

-1/2 AIC & AICC = 302.489 302.467

-1/2 BIC = 285.901 "Penalty factor" = 3.765

Parameter estimates with approx. sampling erors

1 CHOL Z 1 1 0.445142 0.387835E-01

2 CHOL Z 1 2 0.503925E-01 0.714289E-02

3 CHOL Z 2 2 -2.22085 0.553478E-01

4 CHOL A 1 1 -0.387501 0.208207

5 CHOL A 1 2 0.193171E-01 0.187907E-01

6 CHOL A 2 2 -2.30834 0.893534E-01

Convergence criteria for last 3 iterates

Change in log likelihood = 0.042699 0.002897 0.000219

Change in parameter vector = 0.002201 0.001150 0.000221

Norm of gradient vector = 20.1938 4.5639 1.3279

Newton decrement = -0.1099 -0.0079 -0.0006

***** Estimates of residual covariances ************************************

Order of fit = 2

Covariance matrix

1 2.4358

2 0.78648E-01 0.14315E-01

Eigenvalues of covariance matrix

Value 2.44 0.01

(%) 99.52 0.48

Trace 2.45

Matrix of correlations and variance ratios

1 0.8409

2 0.4212 0.5825

Covariances & correlations with approximate sampling errors

1 COVS Z 1 1 2.43582 0.188939 vrat 0.841 0.064

2 COVS Z 1 2 0.786482E-01 0.123840E-01 corr 0.421 0.053

3 COVS Z 2 2 0.143153E-01 0.148915E-02 vrat 0.583 0.067

***** Estimates for RE 1 "animal" ***************************************

No. of levels = 2204

Covariance structure = NRM

Order of fit = 2

Covariance matrix

1 0.46070

2 0.13112E-01 0.10259E-01

Eigenvalues of covariance matrix

Value 0.46 0.01

(%) 97.90 2.10

Trace 0.47

Matrix of correlations and variance ratios

1 0.1591

2 0.1907 0.4175

Covariances & correlations with approximate sampling errors

4 COVS A 1 1 0.460703 0.191843 vrat 0.159 0.064

5 COVS A 1 2 0.131115E-01 0.138132E-01 corr 0.191 0.179

6 COVS A 2 2 0.102587E-01 0.193996E-02 vrat 0.417 0.067

***** Estimates of phenotypic covariances ***********************************

Covariance matrix

1 2.8965

2 0.91760E-01 0.24574E-01

Eigenvalues of covariance matrix

Value 2.90 0.02

(%) 99.26 0.74

Trace 2.92

Correlation matrix

1 1.0000

2 0.3439 1.0000

Covariances & correlations with approximate sampling errors

7 COVS T 1 1 2.89652 0.139843

8 COVS T 1 2 0.917597E-01 0.973035E-02 corr 0.344 0.031

9 COVS T 2 2 0.245740E-01 0.126360E-02

======== end of file ============================25-02-2015==========18:12====

======= Version 30-08-2013 ======================================= **KM** ====

Program WOMBAT: Summary of Pedigree Information

==============================================================================

Two traits analysis (WS x RIG)

Analysis type : "muv 2"

Data file : "DADOS.dat"

Pedigree file : "ReducedPedFile.dat"

Parameter file : "wombat.par"

No. of animal IDs in data file = = 8091

No. of animal IDs in total = = 8490

*****Pedigree Structure for random effect : 1 ****************************

Original no. of animals = 8490

No. of animals after pruning = 8433

... proportion (%) remaining = 99.3

No. of levels w/out records = 342

No. of levels with records = 8091 100.0%

... 1 record(s) = 7149 88.4%

... 2 record(s) = 942 11.6%

No. of animals w/out offspring = 6052 71.8%

No. of animals with offspring = 2381 28.2%

... and records = 2039 24.2%

No. of animals with unknown sire = 313

No. of animals with unknown dam = 384

No. of animals with both parents unknown = 312

No. of animals with records =

... and unknown sire = 1

... and unknown dam = 53

... and both parents unknown = 0

No. of sires = 325

... with progeny in the data = 320

... with records & progeny in data = 288

No. of dams = 2056

... with progeny in the data = 2056

... with records & progeny in data = 1751

No. of animals with known/unpruned grand-parents

... with paternal grandsire = 7358

... with paternal granddam = 7056

... with maternal grandsire = 6535

... with maternal granddam = 6468

random effect no. = 1 NRM

no. of elements in NRM/GIN inverse 31838

log determinant = -5688.7609053187680

random effect no. = 2 IDE

no. of elements in NRM/GIN inverse 0

log determinant = 0.0000000000000000

======== end of file ============================06-02-2015==========14:50====

======= Version 30-08-2013 ======================================= **KM** ====

Program WOMBAT: Summary of information from Set-up step

==============================================================================

Analysis type : "muv 2"

Data file : "DADOS.dat"

Pedigree file : "ReducedPedFile.dat"

Parameter file : "wombat.par"

No. of traits = 2

nrec mean sdev min. max.

1 "WS" 8078 299.703 49.6462 160.030 489.760

2 "RIG" 955 0.443529E-01 1.71606 -5.93190 4.92270

Numbers of individuals/records for pairs of traits

1 2

1 "WS" 8078 942

2 "RIG" 942 955

Covariables

1"WS" nrec mean sdev min. max.

1 "idv(2)" 8078 6.63568 3.07616 2.00000 18.0000

2 "idade(1)" 8078 473.414 105.028 293.000 645.000

2"RIG" nrec mean sdev min. max.

1 "idv(2)" 955 6.15079 3.05304 3.00000 17.0000

2 "idade(1)" 955 369.980 37.3543 267.000 511.000

Fixed effects

1 "WS" nlev

1 "gc" 201

2 "mn" 4

2 "RIG" nlev

1 "gc" 21

Random effects nlev

1 "animal" 8433 NRM

2 "peanim" 2107 IDE

======== end of file ============================06-02-2015==========14:50====

======= Version 30-08-2013 ======================================= **KM** ====

Program WOMBAT: Estimates of covariance components

==============================================================================

Analysis type : "muv 2"

Data file : "DADOS.dat"

Pedigree file : "ReducedPedFile.dat"

Parameter file : "wombat.par"

No. of traits = 2 WS RIG

No. of records = 9033 8078 955

No. of parameters = 7

Maximum log L = -31163.698

-1/2 AIC & AICC = -31170.698 -31170.704

-1/2 BIC = -31195.488 "Penalty factor" = 4.541

Parameter estimates with approx. sampling erors

1 CHOL Z 1 1 20.6236 0.435196

2 CHOL Z 1 2 0.389735E-01 0.834923E-01

3 CHOL Z 2 2 1.55416 0.605499E-01

4 CHOL A 1 1 2.87973 0.481662E-01

5 CHOL A 1 2 0.707613E-01 0.107088

6 CHOL A 2 2 -0.369328 0.200597

7 CHOL B 1 1 2.33330 0.504906E-01

Convergence criteria for last 3 iterates

Change in log likelihood = 0.103811 0.001312 0.000042

Change in parameter vector = 0.001310 0.000185 0.000065

Norm of gradient vector = 10.7022 0.9000 0.1376

Newton decrement = -0.1975 -0.0026 -0.0001

***** Estimates of residual covariances ************************************

Order of fit = 2

Covariance matrix

1 425.33

2 0.80378 2.4169

Eigenvalues of covariance matrix

Value 425.33 2.42

(%) 99.44 0.56

Trace 427.75

Matrix of correlations and variance ratios

1 0.5011

2 0.0251 0.8335

Covariances & correlations with approximate sampling errors

1 COVS Z 1 1 425.333 17.9506 vrat 0.501 0.027

2 COVS Z 1 2 0.803775 1.72249 corr 0.025 0.054

3 COVS Z 2 2 2.41695 0.188838 vrat 0.834 0.064

***** Estimates for RE 1 "animal" ***************************************

No. of levels = 8433

Covariance structure = NRM

Order of fit = 2

Covariance matrix

1 317.18

2 1.2602 0.48276

Eigenvalues of covariance matrix

Value 317.18 0.48

(%) 99.85 0.15

Trace 317.66

Matrix of correlations and variance ratios

1 0.3737

2 0.1018 0.1665

Covariances & correlations with approximate sampling errors

4 COVS A 1 1 317.177 30.5544 vrat 0.374 0.030

5 COVS A 1 2 1.26022 1.90931 corr 0.102 0.152

6 COVS A 2 2 0.482763 0.193868 vrat 0.166 0.064

***** Estimates for RE 2 "peanim" ***************************************

No. of levels = 2107

Covariance structure = IDE

Order of fit = 1

Covariance matrix

1 106.34

Matrix of correlations and variance ratios

1 0.1253

Covariances & correlations with approximate sampling errors

7 COVS B 1 1 106.335 10.7379 vrat 0.125 0.012

***** Estimates of phenotypic covariances ***********************************

Covariance matrix

1 848.85

2 2.0640 2.8997

Eigenvalues of covariance matrix

Value 848.85 2.89

(%) 99.66 0.34

Trace 851.75

Correlation matrix

1 1.0000

2 0.0416 1.0000

Covariances & correlations with approximate sampling errors

8 COVS T 1 1 848.846 18.5460

9 COVS T 1 2 2.06400 1.59351 corr 0.042 0.032

10 COVS T 2 2 2.89971 0.140350

======== end of file ============================06-02-2015==========14:51====

======= Version 30-08-2013 ======================================= **KM** ====

Program WOMBAT: Summary of Pedigree Information

==============================================================================

Two traits analysis (HH x RIG)

Analysis type : "muv 2"

Data file : "DADOS.dat"

Pedigree file : "ReducedPedFile.dat"

Parameter file : "wombat.par"

No. of animal IDs in data file = = 6560

No. of animal IDs in total = = 7100

*****Pedigree Structure for random effect : 1 ****************************

Original no. of animals = 7100

No. of animals after pruning = 7017

... proportion (%) remaining = 98.8

No. of levels w/out records = 457

No. of levels with records = 6560 100.0%

... 1 record(s) = 5617 85.6%

... 2 record(s) = 943 14.4%

No. of animals w/out offspring = 4829 68.8%

No. of animals with offspring = 2188 31.2%

... and records = 1731 24.7%

No. of animals with unknown sire = 148

No. of animals with unknown dam = 237

No. of animals with both parents unknown = 148

No. of animals with records =

... and unknown sire = 0

... and unknown dam = 18

... and both parents unknown = 0

No. of sires = 320

... with progeny in the data = 283

... with records & progeny in data = 240

No. of dams = 1868

... with progeny in the data = 1837

... with records & progeny in data = 1491

No. of animals with known/unpruned grand-parents

... with paternal grandsire = 6738

... with paternal granddam = 6484

... with maternal grandsire = 6339

... with maternal granddam = 6111

random effect no. = 1 NRM

no. of elements in NRM/GIN inverse 26822

log determinant = -4814.8039049931958

======== end of file ============================06-02-2015==========14:53====

======= Version 30-08-2013 ======================================= **KM** ====

Program WOMBAT: Summary of information from Set-up step

==============================================================================

Analysis type : "muv 2"

Data file : "DADOS.dat"

Pedigree file : "ReducedPedFile.dat"

Parameter file : "wombat.par"

No. of traits = 2

nrec mean sdev min. max.

1 "HH" 6548 132.269 5.43028 100.000 149.000

2 "RIG" 955 0.443529E-01 1.71606 -5.93190 4.92270

Numbers of individuals/records for pairs of traits

1 2

1 "HH" 6548 943

2 "RIG" 943 955

Covariables

1"HH" nrec mean sdev min. max.

1 "idv(2)" 6548 6.40043 2.93455 2.00000 17.0000

2 "idade(1)" 6548 472.701 107.333 293.000 645.000

2"RIG" nrec mean sdev min. max.

1 "idv(2)" 955 6.15079 3.05304 3.00000 17.0000

2 "idade(1)" 955 369.980 37.3543 267.000 511.000

Fixed effects

1 "HH" nlev

1 "gc" 169

2 "mn" 4

2 "RIG" nlev

1 "gc" 21

Random effects nlev

1 "animal" 7017 NRM

======== end of file ============================06-02-2015==========14:53====

======= Version 30-08-2013 ======================================= **KM** ====

Program WOMBAT: Estimates of covariance components

==============================================================================

Analysis type : "muv 2"

Data file : "DADOS.dat"

Pedigree file : "ReducedPedFile.dat"

Parameter file : "wombat.par"

No. of traits = 2 HH RIG

No. of records = 7503 6548 955

No. of parameters = 6

Maximum log L = -12412.671

-1/2 AIC & AICC = -12418.671 -12418.677

-1/2 BIC = -12439.360 "Penalty factor" = 4.448

Parameter estimates with approx. sampling erors

1 CHOL Z 1 1 2.44971 0.653974E-01

2 CHOL Z 1 2 -0.219782 0.102803

3 CHOL Z 2 2 1.53466 0.634405E-01

4 CHOL A 1 1 1.12156 0.315711E-01

5 CHOL A 1 2 0.977442E-01 0.105306

6 CHOL A 2 2 -0.353858 0.196006

Convergence criteria for last 3 iterates

Change in log likelihood = 0.175543 0.004218 0.000097

Change in parameter vector = 0.012712 0.001840 0.000298

Norm of gradient vector = 8.5941 2.2549 0.2637

Newton decrement = -0.3059 -0.0073 -0.0002

***** Estimates of residual covariances ************************************

Order of fit = 2

Covariance matrix

1 6.0011

2 -0.53840 2.4035

Eigenvalues of covariance matrix

Value 6.08 2.32

(%) 72.34 27.66

Trace 8.40

Matrix of correlations and variance ratios

1 0.3891

2 -0.1418 0.8271

Covariances & correlations with approximate sampling errors

1 COVS Z 1 1 6.00110 0.320410 vrat 0.389 0.026

2 COVS Z 1 2 -0.538402 0.251429 corr -0.142 0.067

3 COVS Z 2 2 2.40347 0.188597 vrat 0.827 0.064

***** Estimates for RE 1 "animal" ***************************************

No. of levels = 7017

Covariance structure = NRM

Order of fit = 2

Covariance matrix

1 9.4227

2 0.30004 0.50232

Eigenvalues of covariance matrix

Value 9.43 0.49

(%) 95.04 4.96

Trace 9.93

Matrix of correlations and variance ratios

1 0.6109

2 0.1379 0.1729

Covariances & correlations with approximate sampling errors

4 COVS A 1 1 9.42269 0.594970 vrat 0.611 0.026

5 COVS A 1 2 0.300039 0.323403 corr 0.138 0.147

6 COVS A 2 2 0.502323 0.195466 vrat 0.173 0.064

***** Estimates of phenotypic covariances ***********************************

Covariance matrix

1 15.424

2 -0.23836 2.9058

Eigenvalues of covariance matrix

Value 15.43 2.90

(%) 84.17 15.83

Trace 18.33

Correlation matrix

1 1.0000

2 -0.0356 1.0000

Covariances & correlations with approximate sampling errors

7 COVS T 1 1 15.4238 0.383299

8 COVS T 1 2 -0.238363 0.239808 corr -0.036 0.036

9 COVS T 2 2 2.90579 0.141055

======== end of file ============================06-02-2015==========14:53====

======= Version 30-08-2013 ======================================= **KM** ====

Program WOMBAT: Summary of Pedigree Information

==============================================================================

Two traits analysis (CC x RIG)

Analysis type : "muv 2"

Data file : "DADOS.dat"

Pedigree file : "ReducedPedFile.dat"

Parameter file : "wombat.par"

No. of animal IDs in data file = = 3943

No. of animal IDs in total = = 5418

*****Pedigree Structure for random effect : 1 ****************************

Original no. of animals = 5418

No. of animals after pruning = 5330

... proportion (%) remaining = 98.4

No. of levels w/out records = 1387

No. of levels with records = 3943 100.0%

... 1 record(s) = 3055 77.5%

... 2 record(s) = 888 22.5%

No. of animals w/out offspring = 3311 62.1%

No. of animals with offspring = 2019 37.9%

... and records = 632 11.9%

No. of animals with unknown sire = 94

No. of animals with unknown dam = 188

No. of animals with both parents unknown = 94

No. of animals with records =

... and unknown sire = 0

... and unknown dam = 0

... and both parents unknown = 0

No. of sires = 318

... with progeny in the data = 255

... with records & progeny in data = 217

No. of dams = 1701

... with progeny in the data = 1508

... with records & progeny in data = 412

No. of animals with known/unpruned grand-parents

... with paternal grandsire = 5142

... with paternal granddam = 4954

... with maternal grandsire = 4953

... with maternal granddam = 4738

random effect no. = 1 NRM

no. of elements in NRM/GIN inverse 20464

log determinant = -3671.8274443710275

======== end of file ============================06-02-2015==========14:55====

======= Version 30-08-2013 ======================================= **KM** ====

Program WOMBAT: Summary of information from Set-up step

==============================================================================

Analysis type : "muv 2"

Data file : "DADOS.dat"

Pedigree file : "ReducedPedFile.dat"

Parameter file : "wombat.par"

No. of traits = 2

nrec mean sdev min. max.

1 "CC" 3876 164.140 8.72028 128.000 192.000

2 "RIG" 955 0.443529E-01 1.71606 -5.93190 4.92270

Numbers of individuals/records for pairs of traits

1 2

1 "CC" 3876 888

2 "RIG" 888 955

Covariables

1"CC" nrec mean sdev min. max.

1 "idv(2)" 3876 6.27632 2.87660 2.00000 17.0000

2 "idade(1)" 3876 431.403 109.187 293.000 725.000

2"RIG" nrec mean sdev min. max.

1 "idv(2)" 955 6.15079 3.05304 3.00000 17.0000

2 "idade(1)" 955 369.980 37.3543 267.000 511.000

Fixed effects

1 "CC" nlev

1 "gc" 100

2 "mn" 4

2 "RIG" nlev

1 "gc" 21

Random effects nlev

1 "animal" 5330 NRM

======== end of file ============================06-02-2015==========14:55====

======= Version 30-08-2013 ======================================= **KM** ====

Program WOMBAT: Estimates of covariance components

==============================================================================

Analysis type : "muv 2"

Data file : "DADOS.dat"

Pedigree file : "ReducedPedFile.dat"

Parameter file : "wombat.par"

No. of traits = 2 CC RIG

No. of records = 4831 3876 955

No. of parameters = 6

Maximum log L = -9707.075

-1/2 AIC & AICC = -9713.075 -9713.084

-1/2 BIC = -9732.442 "Penalty factor" = 4.228

Parameter estimates with approx. sampling erors

1 CHOL Z 1 1 5.04138 0.117938

2 CHOL Z 1 2 -0.936593E-01 0.839749E-01

3 CHOL Z 2 2 1.55323 0.608417E-01

4 CHOL A 1 1 1.18422 0.729829E-01

5 CHOL A 1 2 0.121478 0.134147

6 CHOL A 2 2 -0.383393 0.204904

Convergence criteria for last 3 iterates

Change in log likelihood = 0.203195 0.003614 0.000120

Change in parameter vector = 0.007709 0.001652 0.000325

Norm of gradient vector = 14.2884 1.1641 0.1656

Newton decrement = -0.3754 -0.0062 -0.0002

***** Estimates of residual covariances ************************************

Order of fit = 2

Covariance matrix

1 25.415

2 -0.47217 2.4213

Eigenvalues of covariance matrix

Value 25.43 2.41

(%) 91.34 8.66

Trace 27.84

Matrix of correlations and variance ratios

1 0.7041

2 -0.0602 0.8348

Covariances & correlations with approximate sampling errors

1 COVS Z 1 1 25.4155 1.18914 vrat 0.704 0.039

2 COVS Z 1 2 -0.472172 0.423109 corr -0.060 0.054

3 COVS Z 2 2 2.42129 0.187909 vrat 0.835 0.063

***** Estimates for RE 1 "animal" ***************************************

No. of levels = 5330

Covariance structure = NRM

Order of fit = 2

Covariance matrix

1 10.681

2 0.39701 0.47926

Eigenvalues of covariance matrix

Value 10.70 0.46

(%) 95.84 4.16

Trace 11.16

Matrix of correlations and variance ratios

1 0.2959

2 0.1755 0.1652

Covariances & correlations with approximate sampling errors

4 COVS A 1 1 10.6806 1.55900 vrat 0.296 0.039

5 COVS A 1 2 0.397006 0.439106 corr 0.175 0.192

6 COVS A 2 2 0.479261 0.192073 vrat 0.165 0.063

***** Estimates of phenotypic covariances ***********************************

Covariance matrix

1 36.096

2 -0.75166E-01 2.9006

Eigenvalues of covariance matrix

Value 36.10 2.90

(%) 92.56 7.44

Trace 39.00

Correlation matrix

1 1.0000

2 -0.0073 1.0000

Covariances & correlations with approximate sampling errors

7 COVS T 1 1 36.0961 0.972429

8 COVS T 1 2 -0.751660E-01 0.364758 corr -0.007 0.036

9 COVS T 2 2 2.90056 0.140285

======== end of file ============================06-02-2015==========14:55====

======= Version 30-08-2013 ======================================= **KM** ====

Program WOMBAT: Summary of Pedigree Information

==============================================================================

Two traits analysis (LEA x RIG)

Analysis type : "muv 2"

Data file : "DADOS.dat"

Pedigree file : "ReducedPedFile.dat"

Parameter file : "wombat.par"

No. of animal IDs in data file = = 2396

No. of animal IDs in total = = 3756

*****Pedigree Structure for random effect : 1 ****************************

Original no. of animals = 3756

No. of animals after pruning = 3665

... proportion (%) remaining = 97.6

No. of levels w/out records = 1269

No. of levels with records = 2396 100.0%

... 1 record(s) = 1554 64.9%

... 2 record(s) = 842 35.1%

No. of animals w/out offspring = 1913 52.2%

No. of animals with offspring = 1752 47.8%

... and records = 483 13.2%

No. of animals with unknown sire = 64

No. of animals with unknown dam = 161

No. of animals with both parents unknown = 64

No. of animals with records =

... and unknown sire = 0

... and unknown dam = 0

... and both parents unknown = 0

No. of sires = 313

... with progeny in the data = 180

... with records & progeny in data = 85

No. of dams = 1439

... with progeny in the data = 1042

... with records & progeny in data = 392

No. of animals with known/unpruned grand-parents

... with paternal grandsire = 3520

... with paternal granddam = 3382

... with maternal grandsire = 3391

... with maternal granddam = 3267

random effect no. = 1 NRM

no. of elements in NRM/GIN inverse 14038

log determinant = -2522.5521618264124

======== end of file ============================06-02-2015==========15:26====

======= Version 30-08-2013 ======================================= **KM** ====

Program WOMBAT: Summary of information from Set-up step

==============================================================================

Analysis type : "muv 2"

Data file : "DADOS.dat"

Pedigree file : "ReducedPedFile.dat"

Parameter file : "wombat.par"

No. of traits = 2

nrec mean sdev min. max.

1 "LEA" 2283 51.4305 8.89390 21.4000 83.4000

2 "RIG" 955 0.443529E-01 1.71606 -5.93190 4.92270

Numbers of individuals/records for pairs of traits

1 2

1 "LEA" 2283 842

2 "RIG" 842 955

Covariables

1"LEA" nrec mean sdev min. max.

1 "idv(2)" 2283 6.11082 2.92852 2.00000 17.0000

2 "idade(1)" 2283 448.951 98.8881 306.000 609.000

2"RIG" nrec mean sdev min. max.

1 "idv(2)" 955 6.15079 3.05304 3.00000 17.0000

2 "idade(1)" 955 369.980 37.3543 267.000 511.000

Fixed effects

1 "LEA" nlev

1 "gc" 65

2 "mn" 4

2 "RIG" nlev

1 "gc" 21

Random effects nlev

1 "animal" 3665 NRM

======== end of file ============================06-02-2015==========15:26====

======= Version 30-08-2013 ======================================= **KM** ====

Program WOMBAT: Estimates of covariance components

==============================================================================

Analysis type : "muv 2"

Data file : "DADOS.dat"

Pedigree file : "ReducedPedFile.dat"

Parameter file : "wombat.par"

No. of traits = 2 LEA RIG

No. of records = 3238 2283 955

No. of parameters = 6

Maximum log L = -6079.310

-1/2 AIC & AICC = -6085.310 -6085.323

-1/2 BIC = -6103.469 "Penalty factor" = 4.026

Parameter estimates with approx. sampling erors

1 CHOL Z 1 1 4.45253 0.174832

2 CHOL Z 1 2 -0.140360 0.102000

3 CHOL Z 2 2 1.56011 0.602648E-01

4 CHOL A 1 1 1.41192 0.681200E-01

5 CHOL A 1 2 0.107965 0.129480

6 CHOL A 2 2 -0.425008 0.221474

Convergence criteria for last 3 iterates

Change in log likelihood = 0.244180 0.000536 0.000007

Change in parameter vector = 0.012180 0.001175 0.000131

Norm of gradient vector = 14.5819 0.2526 0.0201

Newton decrement = -0.4788 -0.0010 -0.0000

***** Estimates of residual covariances ************************************

Order of fit = 2

Covariance matrix

1 19.825

2 -0.62496 2.4536

Eigenvalues of covariance matrix

Value 19.85 2.43

(%) 89.09 10.91

Trace 22.28

Matrix of correlations and variance ratios

1 0.5407

2 -0.0896 0.8482

Covariances & correlations with approximate sampling errors

1 COVS Z 1 1 19.8250 1.55689 vrat 0.541 0.051

2 COVS Z 1 2 -0.624957 0.454038 corr -0.090 0.065

3 COVS Z 2 2 2.45365 0.187491 vrat 0.848 0.062

***** Estimates for RE 1 "animal" ***************************************

No. of levels = 3665

Covariance structure = NRM

Order of fit = 2

Covariance matrix

1 16.841

2 0.44307 0.43906

Eigenvalues of covariance matrix

Value 16.85 0.43

(%) 97.53 2.47

Trace 17.28

Matrix of correlations and variance ratios

1 0.4593

2 0.1629 0.1518

Covariances & correlations with approximate sampling errors

4 COVS A 1 1 16.8414 2.29448 vrat 0.459 0.051

5 COVS A 1 2 0.443068 0.531383 corr 0.163 0.198

6 COVS A 2 2 0.439064 0.187326 vrat 0.152 0.062

***** Estimates of phenotypic covariances ***********************************

Covariance matrix

1 36.666

2 -0.18189 2.8927

Eigenvalues of covariance matrix

Value 36.67 2.89

(%) 92.69 7.31

Trace 39.56

Correlation matrix

1 1.0000

2 -0.0177 1.0000

Covariances & correlations with approximate sampling errors

7 COVS T 1 1 36.6665 1.36199

8 COVS T 1 2 -0.181888 0.388599 corr -0.018 0.038

9 COVS T 2 2 2.89271 0.139142

======== end of file ============================06-02-2015==========15:26====

======= Version 30-08-2013 ======================================= **KM** ====

Program WOMBAT: Summary of Pedigree Information

==============================================================================

Two traits analysis (BF x RIG)

Analysis type : "muv 2"

Data file : "DADOS.dat"

Pedigree file : "ReducedPedFile.dat"

Parameter file : "wombat.par"

No. of animal IDs in data file = = 2396

No. of animal IDs in total = = 3756

*****Pedigree Structure for random effect : 1 ****************************

Original no. of animals = 3756

No. of animals after pruning = 3665

... proportion (%) remaining = 97.6

No. of levels w/out records = 1269

No. of levels with records = 2396 100.0%

... 1 record(s) = 1552 64.8%

... 2 record(s) = 844 35.2%

No. of animals w/out offspring = 1913 52.2%

No. of animals with offspring = 1752 47.8%

... and records = 483 13.2%

No. of animals with unknown sire = 64

No. of animals with unknown dam = 161

No. of animals with both parents unknown = 64

No. of animals with records =

... and unknown sire = 0

... and unknown dam = 0

... and both parents unknown = 0

No. of sires = 313

... with progeny in the data = 180

... with records & progeny in data = 85

No. of dams = 1439

... with progeny in the data = 1042

... with records & progeny in data = 392

No. of animals with known/unpruned grand-parents

... with paternal grandsire = 3520

... with paternal granddam = 3382

... with maternal grandsire = 3391

... with maternal granddam = 3267

random effect no. = 1 NRM

no. of elements in NRM/GIN inverse 14038

log determinant = -2522.5521618264124

======== end of file ============================06-02-2015==========15:31====

======= Version 30-08-2013 ======================================= **KM** ====

Program WOMBAT: Summary of information from Set-up step

==============================================================================

Analysis type : "muv 2"

Data file : "DADOS.dat"

Pedigree file : "ReducedPedFile.dat"

Parameter file : "wombat.par"

No. of traits = 2

nrec mean sdev min. max.

1 "BF" 2285 1.75922 1.42798 0.00000 10.5000

2 "RIG" 955 0.443529E-01 1.71606 -5.93190 4.92270

Numbers of individuals/records for pairs of traits

1 2

1 "RF" 2285 844

2 "RIG" 844 955

Covariables

1"BF" nrec mean sdev min. max.

1 "idv(2)" 2285 6.10810 2.92868 2.00000 17.0000

2 "idade(1)" 2285 449.081 98.9421 306.000 609.000

2"RIG" nrec mean sdev min. max.

1 "idv(2)" 955 6.15079 3.05304 3.00000 17.0000

2 "idade(1)" 955 369.980 37.3543 267.000 511.000

Fixed effects

1 "BF" nlev

1 "gc" 65

2 "mn" 4

2 "RIG" nlev

1 "gc" 21

Random effects nlev

1 "animal" 3665 NRM

======== end of file ============================06-02-2015==========15:31====

======= Version 30-08-2013 ======================================= **KM** ====

Program WOMBAT: Estimates of covariance components

==============================================================================

Analysis type : "muv 2"

Data file : "DADOS.dat"

Pedigree file : "ReducedPedFile.dat"

Parameter file : "wombat.par"

No. of traits = 2 BF RIG

No. of records = 3240 2285 955

No. of parameters = 6

Maximum log L = -2068.767

-1/2 AIC & AICC = -2074.767 -2074.780

-1/2 BIC = -2092.928 "Penalty factor" = 4.027

Parameter estimates with approx. sampling erors

1 CHOL Z 1 1 1.57338 0.592210E-01

2 CHOL Z 1 2 -0.977546E-02 0.439056E-01

3 CHOL Z 2 2 0.800254 0.234499E-01

4 CHOL A 1 1 -0.449522 0.224387

5 CHOL A 1 2 -0.201791 0.111546

6 CHOL A 2 2 -0.713680 0.126804

Convergence criteria for last 3 iterates

Change in log likelihood = 15.613466 0.037738 0.000177

Change in parameter vector = 0.083438 0.010860 0.001306

Norm of gradient vector = 386.4364 13.6327 0.4035

Newton decrement = -29.7770 -0.0784 -0.0004

***** Estimates of residual covariances ************************************

Order of fit = 2

Covariance matrix

1 0.64050

2 -0.15380E-01 2.4755

Eigenvalues of covariance matrix

Value 2.48 0.64

(%) 79.45 20.55

Trace 3.12

Matrix of correlations and variance ratios

1 0.6953

2 -0.0122 0.8588

Covariances & correlations with approximate sampling errors

1 COVS Z 1 1 0.640502 0.375741E-01 vrat 0.695 0.046

2 COVS Z 1 2 -0.153805E-01 0.691148E-01 corr -0.012 0.055

3 COVS Z 2 2 2.47552 0.186354 vrat 0.859 0.061

***** Estimates for RE 1 "animal" ***************************************

No. of levels = 3665

Covariance structure = NRM

Order of fit = 2

Covariance matrix

1 0.28066

2 -0.12873 0.40696

Eigenvalues of covariance matrix

Value 0.49 0.20

(%) 70.85 29.15

Trace 0.69

Matrix of correlations and variance ratios

1 0.3047

2 -0.3809 0.1412

Covariances & correlations with approximate sampling errors

4 COVS A 1 1 0.280661 0.475927E-01 vrat 0.305 0.046

5 COVS A 1 2 -0.128729 0.695295E-01 corr -0.381 0.209

6 COVS A 2 2 0.406958 0.182632 vrat 0.141 0.061

***** Estimates of phenotypic covariances ***********************************

Covariance matrix

1 0.92116

2 -0.14411 2.8825

Eigenvalues of covariance matrix

Value 2.89 0.91

(%) 76.06 23.94

Trace 3.80

Correlation matrix

1 1.0000

2 -0.0884 1.0000

Covariances & correlations with approximate sampling errors

7 COVS T 1 1 0.921163 0.312874E-01

8 COVS T 1 2 -0.144110 0.574420E-01 corr -0.088 0.035

9 COVS T 2 2 2.88248 0.137968

======== end of file ============================06-02-2015==========15:31====

======= Version 30-08-2013 ======================================= **KM** ====

Program WOMBAT: Summary of Pedigree Information

==============================================================================

Two traits analysis (RF x RIG)

Analysis type : "muv 2"

Data file : "DADOS.dat"

Pedigree file : "ReducedPedFile.dat"

Parameter file : "wombat.par"

No. of animal IDs in data file = = 1930

No. of animal IDs in total = = 3174

*****Pedigree Structure for random effect : 1 ****************************

Original no. of animals = 3174

No. of animals after pruning = 3089

... proportion (%) remaining = 97.3

No. of levels w/out records = 1159

No. of levels with records = 1930 100.0%

... 1 record(s) = 1088 56.4%

... 2 record(s) = 842 43.6%

No. of animals w/out offspring = 1497 48.5%

No. of animals with offspring = 1592 51.5%

... and records = 433 14.0%

No. of animals with unknown sire = 60

No. of animals with unknown dam = 147

No. of animals with both parents unknown = 59

No. of animals with records =

... and unknown sire = 0

... and unknown dam = 0

... and both parents unknown = 0

No. of sires = 307

... with progeny in the data = 114

... with records & progeny in data = 44

No. of dams = 1285

... with progeny in the data = 792

... with records & progeny in data = 389

No. of animals with known/unpruned grand-parents

... with paternal grandsire = 2965

... with paternal granddam = 2839

... with maternal grandsire = 2840

... with maternal granddam = 2751

random effect no. = 1 NRM

no. of elements in NRM/GIN inverse 11801

log determinant = -2124.3928089367182

======== end of file ============================06-02-2015==========15:35====

======= Version 30-08-2013 ======================================= **KM** ====

Program WOMBAT: Summary of information from Set-up step

==============================================================================

Analysis type : "muv 2"

Data file : "DADOS.dat"

Pedigree file : "ReducedPedFile.dat"

Parameter file : "wombat.par"

No. of traits = 2

nrec mean sdev min. max.

1 "RF" 1817 5.08663 2.54341 0.00000 19.2000

2 "RIG" 955 0.443529E-01 1.71606 -5.93190 4.92270

Numbers of individuals/records for pairs of traits

1 2

1 "RF" 1817 842

2 "RIG" 842 955

Covariables

1"RF" nrec mean sdev min. max.

1 "idv(2)" 1817 6.09191 3.03138 2.00000 17.0000

2 "idade(1)" 1817 469.100 100.703 306.000 609.000

2"RIG" nrec mean sdev min. max.

1 "idv(2)" 955 6.15079 3.05304 3.00000 17.0000

2 "idade(1)" 955 369.980 37.3543 267.000 511.000

Fixed effects

1 "RF" nlev

1 "gc" 51

2 "mn" 3

2 "RIG" nlev

1 "gc" 21

Random effects nlev

1 "animal" 3089 NRM

======== end of file ============================06-02-2015==========15:35====

======= Version 30-08-2013 ======================================= **KM** ====

Program WOMBAT: Estimates of covariance components

==============================================================================

Analysis type : "muv 2"

Data file : "DADOS.dat"

Pedigree file : "ReducedPedFile.dat"

Parameter file : "wombat.par"

No. of traits = 2 RF RIG

No. of records = 2772 1817 955

No. of parameters = 6

Maximum log L = -2742.985

-1/2 AIC & AICC = -2748.985 -2749.001

-1/2 BIC = -2766.679 "Penalty factor" = 3.949

Parameter estimates with approx. sampling erors

1 CHOL Z 1 1 1.57364 0.597827E-01

2 CHOL Z 1 2 0.339249E-01 0.752011E-01

3 CHOL Z 2 2 1.26569 0.463799E-01

4 CHOL A 1 1 -0.306090E-03 0.806570E-01

5 CHOL A 1 2 -0.204016 0.126463

6 CHOL A 2 2 -0.501069 0.255080

Convergence criteria for last 3 iterates

Change in log likelihood = 9.969523 0.065154 0.000028

Change in parameter vector = 0.067683 0.008320 0.000800

Norm of gradient vector = 180.7813 13.3241 0.1010

Newton decrement = -19.1561 -0.1296 -0.0001

***** Estimates of residual covariances ************************************

Order of fit = 2

Covariance matrix

1 1.6031

2 0.53386E-01 2.4763

Eigenvalues of covariance matrix

Value 2.48 1.60

(%) 60.78 39.22

Trace 4.08

Matrix of correlations and variance ratios

1 0.6160

2 0.0268 0.8583

Covariances & correlations with approximate sampling errors

1 COVS Z 1 1 1.60312 0.117028 vrat 0.616 0.053

2 COVS Z 1 2 0.533857E-01 0.118259 corr 0.027 0.059

3 COVS Z 2 2 2.47635 0.188153 vrat 0.858 0.062

***** Estimates for RE 1 "animal" ***************************************

No. of levels = 3089

Covariance structure = NRM

Order of fit = 2

Covariance matrix

1 0.99939

2 -0.20395 0.40872

Eigenvalues of covariance matrix

Value 1.06 0.35

(%) 75.49 24.51

Trace 1.41

Matrix of correlations and variance ratios

1 0.3840

2 -0.3191 0.1417

Covariances & correlations with approximate sampling errors

4 COVS A 1 1 0.999388 0.161215 vrat 0.384 0.053

5 COVS A 1 2 -0.203954 0.127616 corr -0.319 0.203

6 COVS A 2 2 0.408716 0.185517 vrat 0.142 0.062

***** Estimates of phenotypic covariances ***********************************

Covariance matrix

1 2.6025

2 -0.15057 2.8851

Eigenvalues of covariance matrix

Value 2.95 2.54

(%) 53.76 46.24

Trace 5.49

Correlation matrix

1 1.0000

2 -0.0549 1.0000

Covariances & correlations with approximate sampling errors

7 COVS T 1 1 2.60251 0.102918

8 COVS T 1 2 -0.150568 0.994552E-01 corr -0.055 0.036

9 COVS T 2 2 2.88506 0.138305

======== end of file ============================06-02-2015==========15:35====

======= Version 30-08-2013 ======================================= **KM** ====

Program WOMBAT: Summary of Pedigree Information

==============================================================================

Two traits analysis (RIGb x DMI)

Analysis type : "muv 2"

Data file : "DADOS.dat"

Pedigree file : "ReducedPedFile.dat"

Parameter file : "wombat.par"

No. of animal IDs in data file = = 955

No. of animal IDs in total = = 2288

*****Pedigree Structure for random effect : 1 ****************************

Original no. of animals = 2288

No. of animals after pruning = 2204

... proportion (%) remaining = 96.3

No. of levels w/out records = 1249

No. of levels with records = 955 100.0%

... 1 record(s) = 61 6.4%

... 2 record(s) = 894 93.6%

No. of animals w/out offspring = 828 37.6%

No. of animals with offspring = 1376 62.4%

... and records = 127 5.8%

No. of animals with unknown sire = 59

No. of animals with unknown dam = 145

No. of animals with both parents unknown = 58

No. of animals with records =

... and unknown sire = 0

... and unknown dam = 0

... and both parents unknown = 0

No. of sires = 296

... with progeny in the data = 78

... with records & progeny in data = 18

No. of dams = 1080

... with progeny in the data = 542

... with records & progeny in data = 98

No. of animals with known/unpruned grand-parents

... with paternal grandsire = 2083

... with paternal granddam = 1957

... with maternal grandsire = 1959

... with maternal granddam = 1872

random effect no. = 1 NRM

no. of elements in NRM/GIN inverse 8375

log determinant = -1487.8684515369519

======== end of file ============================25-02-2015==========18:14====

======= Version 30-08-2013 ======================================= **KM** ====

Program WOMBAT: Summary of information from Set-up step

==============================================================================

Analysis type : "muv 2"

Data file : "DADOS.dat"

Pedigree file : "ReducedPedFile.dat"

Parameter file : "wombat.par"

No. of traits = 2

nrec mean sdev min. max.

1 "RIGb" 894 0.424196E-01 1.70140 -6.35080 4.85930

2 "DMI" 955 6.83104 1.33500 2.15500 12.6400

Numbers of individuals/records for pairs of traits

1 2

1 "RIGb" 894 894

2 "DMI" 894 955

Covariables

1"RIGb" nrec mean sdev min. max.

1 "idv(2)" 894 6.16443 3.04994 3.00000 17.0000

2 "idade(1)" 894 368.964 35.9309 267.000 511.000

2"DMI" nrec mean sdev min. max.

1 "idv(2)" 955 6.15079 3.05304 3.00000 17.0000

2 "idade(1)" 955 369.980 37.3543 267.000 511.000

Fixed effects

1 "RIGb" nlev

1 "gc" 21

2 "DMI" nlev

1 "gc" 21

Random effects nlev

1 "animal" 2204 NRM

======== end of file ============================25-02-2015==========18:14====

======= Version 30-08-2013 ======================================= **KM** ====

Program WOMBAT: Estimates of covariance components

==============================================================================

Analysis type : "muv 2"

Data file : "DADOS.dat"

Pedigree file : "ReducedPedFile.dat"

Parameter file : "wombat.par"

No. of traits = 2 RIGb DMI

No. of records = 1849 894 955

No. of parameters = 6

Maximum log L = -1200.817

-1/2 AIC & AICC = -1206.817 -1206.840

-1/2 BIC = -1223.305 "Penalty factor" = 3.748

Parameter estimates with approx. sampling erors

1 CHOL Z 1 1 1.60216 0.588801E-01

2 CHOL Z 1 2 -0.334476 0.378941E-01

3 CHOL Z 2 2 0.553592 0.357428E-01

4 CHOL A 1 1 -0.621042 0.297897

5 CHOL A 1 2 -0.166162 0.120124

6 CHOL A 2 2 -0.547556 0.859458E-01

Convergence criteria for last 3 iterates

Change in log likelihood = 0.337745 0.000705 0.000033

Change in parameter vector = 0.029742 0.003452 0.000767

Norm of gradient vector = 21.8124 0.5165 0.0823

Newton decrement = -0.6688 -0.0017 -0.0001

***** Estimates of residual covariances ************************************

Order of fit = 2

Covariance matrix

1 2.5669

2 -0.53588 0.41834

Eigenvalues of covariance matrix

Value 2.69 0.29

(%) 90.22 9.78

Trace 2.99

Matrix of correlations and variance ratios

1 0.8989

2 -0.5171 0.5360

Covariances & correlations with approximate sampling errors

1 COVS Z 1 1 2.56691 0.188670 vrat 0.899 0.059

2 COVS Z 1 2 -0.535883 0.692749E-01 corr -0.517 0.050

3 COVS Z 2 2 0.418338 0.468204E-01 vrat 0.536 0.067

***** Estimates for RE 1 "animal" ***************************************

No. of levels = 2204

Covariance structure = NRM

Order of fit = 2

Covariance matrix

1 0.28878

2 -0.89293E-01 0.36211

Eigenvalues of covariance matrix

Value 0.42 0.23

(%) 64.83 35.17

Trace 0.65

Matrix of correlations and variance ratios

1 0.1011

2 -0.2761 0.4640

Covariances & correlations with approximate sampling errors

4 COVS A 1 1 0.288782 0.172055 vrat 0.101 0.059

5 COVS A 1 2 -0.892928E-01 0.726123E-01 corr -0.276 0.190

6 COVS A 2 2 0.362112 0.634501E-01 vrat 0.464 0.067

***** Estimates of phenotypic covariances ***********************************

Covariance matrix

1 2.8557

2 -0.62518 0.78045

Eigenvalues of covariance matrix

Value 3.03 0.61

(%) 83.32 16.68

Trace 3.64

Correlation matrix

1 1.0000

2 -0.4188 1.0000

Covariances & correlations with approximate sampling errors

7 COVS T 1 1 2.85569 0.139629

8 COVS T 1 2 -0.625176 0.556075E-01 corr -0.419 0.029

9 COVS T 2 2 0.780450 0.406572E-01

======== end of file ============================25-02-2015==========18:14====

======= Version 30-08-2013 ======================================= **KM** ====

Program WOMBAT: Summary of Pedigree Information

==============================================================================

Two traits analysis (RIGb x ADG)

Analysis type : "muv 2"

Data file : "DADOS.dat"

Pedigree file : "ReducedPedFile.dat"

Parameter file : "wombat.par"

No. of animal IDs in data file = = 955

No. of animal IDs in total = = 2288

*****Pedigree Structure for random effect : 1 ****************************

Original no. of animals = 2288

No. of animals after pruning = 2204

... proportion (%) remaining = 96.3

No. of levels w/out records = 1249

No. of levels with records = 955 100.0%

... 1 record(s) = 61 6.4%

... 2 record(s) = 894 93.6%

No. of animals w/out offspring = 828 37.6%

No. of animals with offspring = 1376 62.4%

... and records = 127 5.8%

No. of animals with unknown sire = 59

No. of animals with unknown dam = 145

No. of animals with both parents unknown = 58

No. of animals with records =

... and unknown sire = 0

... and unknown dam = 0

... and both parents unknown = 0

No. of sires = 296

... with progeny in the data = 78

... with records & progeny in data = 18

No. of dams = 1080

... with progeny in the data = 542

... with records & progeny in data = 98

No. of animals with known/unpruned grand-parents

... with paternal grandsire = 2083

... with paternal granddam = 1957

... with maternal grandsire = 1959

... with maternal granddam = 1872

random effect no. = 1 NRM

no. of elements in NRM/GIN inverse 8375

log determinant = -1487.8684515369519

======== end of file ============================25-02-2015==========18:16====

======= Version 30-08-2013 ======================================= **KM** ====

Program WOMBAT: Summary of information from Set-up step

==============================================================================

Analysis type : "muv 2"

Data file : "DADOS.dat"

Pedigree file : "ReducedPedFile.dat"

Parameter file : "wombat.par"

No. of traits = 2

nrec mean sdev min. max.

1 "RIGb" 894 0.424196E-01 1.70140 -6.35080 4.85930

2 "ADG" 955 1.00846 0.260202 0.176000 1.71800

Numbers of individuals/records for pairs of traits

1 2

1 "RIGb" 894 894

2 "ADG" 894 955

Covariables

1"RIGb" nrec mean sdev min. max.

1 "idv(2)" 894 6.16443 3.04994 3.00000 17.0000

2 "idade(1)" 894 368.964 35.9309 267.000 511.000

2"ADG" nrec mean sdev min. max.

1 "idv(2)" 955 6.15079 3.05304 3.00000 17.0000

2 "idade(1)" 955 369.980 37.3543 267.000 511.000

Fixed effects

1 "RIGb" nlev

1 "gc" 21

2 "ADG" nlev

1 "gc" 21

Random effects nlev

1 "animal" 2204 NRM

======== end of file ============================25-02-2015==========18:16====

======= Version 30-08-2013 ======================================= **KM** ====

Program WOMBAT: Estimates of covariance components

==============================================================================

Analysis type : "muv 2"

Data file : "DADOS.dat"

Pedigree file : "ReducedPedFile.dat"

Parameter file : "wombat.par"

No. of traits = 2 RIGb ADG

No. of records = 1849 894 955

No. of parameters = 6

Maximum log L = 376.320

-1/2 AIC & AICC = 370.320 370.297

-1/2 BIC = 353.831 "Penalty factor" = 3.748

Parameter estimates with approx. sampling erors

1 CHOL Z 1 1 0.462401 0.372651E-01

2 CHOL Z 1 2 0.541864E-01 0.693428E-02

3 CHOL Z 2 2 -2.23414 0.566445E-01

4 CHOL A 1 1 -0.552514 0.265732

5 CHOL A 1 2 0.140926E-01 0.210899E-01

6 CHOL A 2 2 -2.30608 0.891388E-01

Convergence criteria for last 3 iterates

Change in log likelihood = 0.006267 0.000609 0.000066

Change in parameter vector = 0.002331 0.000478 0.000193

Norm of gradient vector = 5.9624 2.0829 0.6701

Newton decrement = -0.0179 -0.0018 -0.0002

***** Estimates of residual covariances ************************************

Order of fit = 2

Covariance matrix

1 2.5214

2 0.86042E-01 0.14403E-01

Eigenvalues of covariance matrix

Value 2.52 0.01

(%) 99.55 0.45

Trace 2.54

Matrix of correlations and variance ratios

1 0.8839

2 0.4515 0.5871

Covariances & correlations with approximate sampling errors

1 COVS Z 1 1 2.52137 0.187918 vrat 0.884 0.060

2 COVS Z 1 2 0.860416E-01 0.123146E-01 corr 0.452 0.051

3 COVS Z 2 2 0.144031E-01 0.147795E-02 vrat 0.587 0.067

***** Estimates for RE 1 "animal" ***************************************

No. of levels = 2204

Covariance structure = NRM

Order of fit = 2

Covariance matrix

1 0.33120

2 0.81103E-02 0.10129E-01

Eigenvalues of covariance matrix

Value 0.33 0.01

(%) 97.09 2.91

Trace 0.34

Matrix of correlations and variance ratios

1 0.1161

2 0.1400 0.4129

Covariances & correlations with approximate sampling errors

4 COVS A 1 1 0.331202 0.176021 vrat 0.116 0.060

5 COVS A 1 2 0.811029E-02 0.129825E-01 corr 0.140 0.205

6 COVS A 2 2 0.101289E-01 0.191220E-02 vrat 0.413 0.067

***** Estimates of phenotypic covariances ***********************************

Covariance matrix

1 2.8526

2 0.94152E-01 0.24532E-01

Eigenvalues of covariance matrix

Value 2.86 0.02

(%) 99.26 0.74

Trace 2.88

Correlation matrix

1 1.0000

2 0.3559 1.0000

Covariances & correlations with approximate sampling errors

7 COVS T 1 1 2.85257 0.139949

8 COVS T 1 2 0.941519E-01 0.969787E-02 corr 0.356 0.031

9 COVS T 2 2 0.245320E-01 0.125630E-02

======== end of file ============================25-02-2015==========18:16====

======= Version 30-08-2013 ======================================= **KM** ====

Program WOMBAT: Summary of Pedigree Information

==============================================================================

Two traits analysis (WS x RIGb)

Analysis type : "muv 2"

Data file : "DADOS.dat"

Pedigree file : "ReducedPedFile.dat"

Parameter file : "wombat.par"

No. of animal IDs in data file = = 8090

No. of animal IDs in total = = 8489

*****Pedigree Structure for random effect : 1 ****************************

Original no. of animals = 8489

No. of animals after pruning = 8432

... proportion (%) remaining = 99.3

No. of levels w/out records = 342

No. of levels with records = 8090 100.0%

... 1 record(s) = 7208 89.1%

... 2 record(s) = 882 10.9%

No. of animals w/out offspring = 6051 71.8%

No. of animals with offspring = 2381 28.2%

... and records = 2039 24.2%

No. of animals with unknown sire = 313

No. of animals with unknown dam = 384

No. of animals with both parents unknown = 312

No. of animals with records =

... and unknown sire = 1

... and unknown dam = 53

... and both parents unknown = 0

No. of sires = 325

... with progeny in the data = 320

... with records & progeny in data = 288

No. of dams = 2056

... with progeny in the data = 2056

... with records & progeny in data = 1751

No. of animals with known/unpruned grand-parents

... with paternal grandsire = 7357

... with paternal granddam = 7055

... with maternal grandsire = 6534

... with maternal granddam = 6467

random effect no. = 1 NRM

no. of elements in NRM/GIN inverse 31835

log determinant = -5688.0517714250827

random effect no. = 2 IDE

no. of elements in NRM/GIN inverse 0

log determinant = 0.0000000000000000

======== end of file ============================06-02-2015==========15:57====

======= Version 30-08-2013 ======================================= **KM** ====

Program WOMBAT: Summary of information from Set-up step

==============================================================================

Analysis type : "muv 2"

Data file : "DADOS.dat"

Pedigree file : "ReducedPedFile.dat"

Parameter file : "wombat.par"

No. of traits = 2

nrec mean sdev min. max.

1 "WS" 8078 299.703 49.6462 160.030 489.760

2 "RIGb" 894 0.424196E-01 1.70140 -6.35080 4.85930

Numbers of individuals/records for pairs of traits

1 2

1 "WS" 8078 882

2 "RIGb" 882 894

Covariables

1"WS" nrec mean sdev min. max.

1 "idv(2)" 8078 6.63568 3.07616 2.00000 18.0000

2 "idade(1)" 8078 473.414 105.028 293.000 645.000

2"RIGb" nrec mean sdev min. max.

1 "idv(2)" 894 6.16443 3.04994 3.00000 17.0000

2 "idade(1)" 894 368.964 35.9309 267.000 511.000

Fixed effects

1 "WS" nlev

1 "gc" 201

2 "mn" 4

2 "RIGb" nlev

1 "gc" 21

Random effects nlev

1 "animal" 8432 NRM

2 "peanim" 2107 IDE

======== end of file ============================06-02-2015==========15:57====

======= Version 30-08-2013 ======================================= **KM** ====

Program WOMBAT: Estimates of covariance components

==============================================================================

Analysis type : "muv 2"

Data file : "DADOS.dat"

Pedigree file : "ReducedPedFile.dat"

Parameter file : "wombat.par"

No. of traits = 2 WS RIGb

No. of records = 8972 8078 894

No. of parameters = 7

Maximum log L = -31098.826

-1/2 AIC & AICC = -31105.826 -31105.832

-1/2 BIC = -31130.591 "Penalty factor" = 4.538

Parameter estimates with approx. sampling erors

1 CHOL Z 1 1 20.6363 0.434608

2 CHOL Z 1 2 0.872750E-01 0.845264E-01

3 CHOL Z 2 2 1.58625 0.591908E-01

4 CHOL A 1 1 2.87805 0.482525E-01

5 CHOL A 1 2 0.498844E-01 0.102884

6 CHOL A 2 2 -0.543474 0.261238

7 CHOL B 1 1 2.33403 0.504371E-01

Convergence criteria for last 3 iterates

Change in log likelihood = 0.103728 0.001201 0.000037

Change in parameter vector = 0.001240 0.000159 0.000062

Norm of gradient vector = 10.8506 0.8857 0.1359

Newton decrement = -0.1959 -0.0023 -0.0001

***** Estimates of residual covariances ************************************

Order of fit = 2

Covariance matrix

1 425.86

2 1.8010 2.5238

Eigenvalues of covariance matrix

Value 425.86 2.52

(%) 99.41 0.59

Trace 428.38

Matrix of correlations and variance ratios

1 0.5019

2 0.0549 0.8814

Covariances & correlations with approximate sampling errors

1 COVS Z 1 1 425.857 17.9374 vrat 0.502 0.027

2 COVS Z 1 2 1.80103 1.74533 corr 0.055 0.053

3 COVS Z 2 2 2.52380 0.189122 vrat 0.881 0.061

***** Estimates for RE 1 "animal" ***************************************

No. of levels = 8432

Covariance structure = NRM

Order of fit = 2

Covariance matrix

1 316.12

2 0.88693 0.33973

Eigenvalues of covariance matrix

Value 316.12 0.34

(%) 99.89 0.11

Trace 316.46

Matrix of correlations and variance ratios

1 0.3726

2 0.0856 0.1186

Covariances & correlations with approximate sampling errors

4 COVS A 1 1 316.115 30.5067 vrat 0.373 0.030

5 COVS A 1 2 0.886927 1.83042 corr 0.086 0.173

6 COVS A 2 2 0.339733 0.177968 vrat 0.119 0.061

***** Estimates for RE 2 "peanim" ***************************************

No. of levels = 2107

Covariance structure = IDE

Order of fit = 1

Covariance matrix

1 106.49

Matrix of correlations and variance ratios

1 0.1255

Covariances & correlations with approximate sampling errors

7 COVS B 1 1 106.492 10.7423 vrat 0.126 0.012

***** Estimates of phenotypic covariances ***********************************

Covariance matrix

1 848.46

2 2.6880 2.8635

Eigenvalues of covariance matrix

Value 848.47 2.85

(%) 99.66 0.34

Trace 851.33

Correlation matrix

1 1.0000

2 0.0545 1.0000

Covariances & correlations with approximate sampling errors

8 COVS T 1 1 848.464 18.5225

9 COVS T 1 2 2.68796 1.60558 corr 0.055 0.032

10 COVS T 2 2 2.86353 0.140892

======== end of file ============================06-02-2015==========15:57====

======= Version 30-08-2013 ======================================= **KM** ====

Program WOMBAT: Summary of Pedigree Information

==============================================================================

Two traits analysis (HH x RIGb)

Analysis type : "muv 2"

Data file : "DADOS.dat"

Pedigree file : "ReducedPedFile.dat"

Parameter file : "wombat.par"

No. of animal IDs in data file = = 6560

No. of animal IDs in total = = 7100

*****Pedigree Structure for random effect : 1 ****************************

Original no. of animals = 7100

No. of animals after pruning = 7017

... proportion (%) remaining = 98.8

No. of levels w/out records = 457

No. of levels with records = 6560 100.0%

... 1 record(s) = 5617 85.6%

... 2 record(s) = 943 14.4%

No. of animals w/out offspring = 4829 68.8%

No. of animals with offspring = 2188 31.2%

... and records = 1731 24.7%

No. of animals with unknown sire = 148

No. of animals with unknown dam = 237

No. of animals with both parents unknown = 148

No. of animals with records =

... and unknown sire = 0

... and unknown dam = 18

... and both parents unknown = 0

No. of sires = 320

... with progeny in the data = 283

... with records & progeny in data = 240

No. of dams = 1868

... with progeny in the data = 1837

... with records & progeny in data = 1491

No. of animals with known/unpruned grand-parents

... with paternal grandsire = 6738

... with paternal granddam = 6484

... with maternal grandsire = 6339

... with maternal granddam = 6111

random effect no. = 1 NRM

no. of elements in NRM/GIN inverse 26822

log determinant = -4814.8039049931958

======== end of file ============================06-02-2015==========14:53====

======= Version 30-08-2013 ======================================= **KM** ====

Program WOMBAT: Summary of information from Set-up step

==============================================================================

Analysis type : "muv 2"

Data file : "DADOS.dat"

Pedigree file : "ReducedPedFile.dat"

Parameter file : "wombat.par"

No. of traits = 2

nrec mean sdev min. max.

1 "HH" 6548 132.269 5.43028 100.000 149.000

2 "RIGb" 955 0.443529E-01 1.71606 -5.93190 4.92270

Numbers of individuals/records for pairs of traits

1 2

1 "HH" 6548 943

2 "RIGb" 943 955

Covariables

1"HH" nrec mean sdev min. max.

1 "idv(2)" 6548 6.40043 2.93455 2.00000 17.0000

2 "idade(1)" 6548 472.701 107.333 293.000 645.000

2"RIGb" nrec mean sdev min. max.

1 "idv(2)" 955 6.15079 3.05304 3.00000 17.0000

2 "idade(1)" 955 369.980 37.3543 267.000 511.000

Fixed effects

1 "HH" nlev

1 "gc" 169

2 "mn" 4

2 "RIGb" nlev

1 "gc" 21

Random effects nlev

1 "animal" 7017 NRM

======== end of file ============================06-02-2015==========14:53====

======= Version 30-08-2013 ======================================= **KM** ====

Program WOMBAT: Estimates of covariance components

==============================================================================

Analysis type : "muv 2"

Data file : "DADOS.dat"

Pedigree file : "ReducedPedFile.dat"

Parameter file : "wombat.par"

No. of traits = 2 HH RIGb

No. of records = 7503 6548 955

No. of parameters = 6

Maximum log L = -12412.671

-1/2 AIC & AICC = -12418.671 -12418.677

-1/2 BIC = -12439.360 "Penalty factor" = 4.448

Parameter estimates with approx. sampling erors

1 CHOL Z 1 1 2.44971 0.653974E-01

2 CHOL Z 1 2 -0.219782 0.102803

3 CHOL Z 2 2 1.53466 0.634405E-01

4 CHOL A 1 1 1.12156 0.315711E-01

5 CHOL A 1 2 0.977442E-01 0.105306

6 CHOL A 2 2 -0.353858 0.196006

Convergence criteria for last 3 iterates

Change in log likelihood = 0.175543 0.004218 0.000097

Change in parameter vector = 0.012712 0.001840 0.000298

Norm of gradient vector = 8.5941 2.2549 0.2637

Newton decrement = -0.3059 -0.0073 -0.0002

***** Estimates of residual covariances ************************************

Order of fit = 2

Covariance matrix

1 6.0011

2 -0.53840 2.4035

Eigenvalues of covariance matrix

Value 6.08 2.32

(%) 72.34 27.66

Trace 8.40

Matrix of correlations and variance ratios

1 0.3891

2 -0.1418 0.8271

Covariances & correlations with approximate sampling errors

1 COVS Z 1 1 6.00110 0.320410 vrat 0.389 0.026

2 COVS Z 1 2 -0.538402 0.251429 corr -0.142 0.067

3 COVS Z 2 2 2.40347 0.188597 vrat 0.827 0.064

***** Estimates for RE 1 "animal" ***************************************

No. of levels = 7017

Covariance structure = NRM

Order of fit = 2

Covariance matrix

1 9.4227

2 0.30004 0.50232

Eigenvalues of covariance matrix

Value 9.43 0.49

(%) 95.04 4.96

Trace 9.93

Matrix of correlations and variance ratios

1 0.6109

2 0.1379 0.1729

Covariances & correlations with approximate sampling errors

4 COVS A 1 1 9.42269 0.594970 vrat 0.611 0.026

5 COVS A 1 2 0.300039 0.323403 corr 0.138 0.147

6 COVS A 2 2 0.502323 0.195466 vrat 0.173 0.064

***** Estimates of phenotypic covariances ***********************************

Covariance matrix

1 15.424

2 -0.23836 2.9058

Eigenvalues of covariance matrix

Value 15.43 2.90

(%) 84.17 15.83

Trace 18.33

Correlation matrix

1 1.0000

2 -0.0356 1.0000

Covariances & correlations with approximate sampling errors

7 COVS T 1 1 15.4238 0.383299

8 COVS T 1 2 -0.238363 0.239808 corr -0.036 0.036

9 COVS T 2 2 2.90579 0.141055

======== end of file ============================06-02-2015==========14:53====

======= Version 30-08-2013 ======================================= **KM** ====

Program WOMBAT: Summary of Pedigree Information

==============================================================================

Two traits analysis (CC x RIGb)

Analysis type : "muv 2"

Data file : "DADOS.dat"

Pedigree file : "ReducedPedFile.dat"

Parameter file : "wombat.par"

No. of animal IDs in data file = = 3941

No. of animal IDs in total = = 5416

*****Pedigree Structure for random effect : 1 ****************************

Original no. of animals = 5416

No. of animals after pruning = 5328

... proportion (%) remaining = 98.4

No. of levels w/out records = 1387

No. of levels with records = 3941 100.0%

... 1 record(s) = 3112 79.0%

... 2 record(s) = 829 21.0%

No. of animals w/out offspring = 3309 62.1%

No. of animals with offspring = 2019 37.9%

... and records = 632 11.9%

No. of animals with unknown sire = 94

No. of animals with unknown dam = 188

No. of animals with both parents unknown = 94

No. of animals with records =

... and unknown sire = 0

... and unknown dam = 0

... and both parents unknown = 0

No. of sires = 318

... with progeny in the data = 255

... with records & progeny in data = 217

No. of dams = 1701

... with progeny in the data = 1508

... with records & progeny in data = 412

No. of animals with known/unpruned grand-parents

... with paternal grandsire = 5140

... with paternal granddam = 4952

... with maternal grandsire = 4951

... with maternal granddam = 4736

Inbreeding coefficients for random effect 1 computed

No. of inbred animals = 4322

Average inbreeding coefficient = 2.1289 (in %)

... amongst inbred animals = 2.6244 (in %)

random effect no. = 1 NRM

no. of elements in NRM/GIN inverse 20457

log determinant = -3670.4017279806212

======== end of file ============================06-02-2015==========15:58====

======= Version 30-08-2013 ======================================= **KM** ====

Program WOMBAT: Summary of information from Set-up step

==============================================================================

Analysis type : "muv 2"

Data file : "DADOS.dat"

Pedigree file : "ReducedPedFile.dat"

Parameter file : "wombat.par"

No. of traits = 2

nrec mean sdev min. max.

1 "CC" 3876 164.140 8.72028 128.000 192.000

2 "RIGb" 894 0.424196E-01 1.70140 -6.35080 4.85930

Numbers of individuals/records for pairs of traits

1 2

1 "CC" 3876 829

2 "RIGb" 829 894

Covariables

1"CC" nrec mean sdev min. max.

1 "idv(2)" 3876 6.27632 2.87660 2.00000 17.0000

2 "idade(1)" 3876 431.403 109.187 293.000 725.000

2"RIGb" nrec mean sdev min. max.

1 "idv(2)" 894 6.16443 3.04994 3.00000 17.0000

2 "idade(1)" 894 368.964 35.9309 267.000 511.000

Fixed effects

1 "CC" nlev

1 "gc" 100

2 "mn" 4

2 "RIGb" nlev

1 "gc" 21

Random effects nlev

1 "animal" 5328 NRM

======== end of file ============================06-02-2015==========15:58====

======= Version 30-08-2013 ======================================= **KM** ====

Program WOMBAT: Estimates of covariance components

==============================================================================

Analysis type : "muv 2"

Data file : "DADOS.dat"

Pedigree file : "ReducedPedFile.dat"

Parameter file : "wombat.par"

No. of traits = 2 CC RIGb

No. of records = 4770 3876 894

No. of parameters = 6

Maximum log L = -9643.046

-1/2 AIC & AICC = -9649.046 -9649.055

-1/2 BIC = -9668.374 "Penalty factor" = 4.221

Parameter estimates with approx. sampling erors

1 CHOL Z 1 1 5.04242 0.117876

2 CHOL Z 1 2 -0.555472E-01 0.859362E-01

3 CHOL Z 2 2 1.58616 0.595323E-01

4 CHOL A 1 1 1.18349 0.730280E-01

5 CHOL A 1 2 0.133373 0.132851

6 CHOL A 2 2 -0.553062 0.262125

Convergence criteria for last 3 iterates

Change in log likelihood = 0.203007 0.003602 0.000119

Change in parameter vector = 0.007663 0.001641 0.000323

Norm of gradient vector = 14.2685 1.1597 0.1651

Newton decrement = -0.3751 -0.0061 -0.0002

***** Estimates of residual covariances ************************************

Order of fit = 2

Covariance matrix

1 25.426

2 -0.28009 2.5190

Eigenvalues of covariance matrix

Value 25.43 2.52

(%) 91.00 9.00

Trace 27.94

Matrix of correlations and variance ratios

1 0.7045

2 -0.0350 0.8784

Covariances & correlations with approximate sampling errors

1 COVS Z 1 1 25.4259 1.18876 vrat 0.704 0.038

2 COVS Z 1 2 -0.280092 0.433111 corr -0.035 0.054

3 COVS Z 2 2 2.51897 0.188020 vrat 0.878 0.060

***** Estimates for RE 1 "animal" ***************************************

No. of levels = 5328

Covariance structure = NRM

Order of fit = 2

Covariance matrix

1 10.665

2 0.43556 0.34863

Eigenvalues of covariance matrix

Value 10.68 0.33

(%) 97.00 3.00

Trace 11.01

Matrix of correlations and variance ratios

1 0.2955

2 0.2259 0.1216

Covariances & correlations with approximate sampling errors

4 COVS A 1 1 10.6651 1.55770 vrat 0.296 0.038

5 COVS A 1 2 0.435562 0.435035 corr 0.226 0.221

6 COVS A 2 2 0.348627 0.177185 vrat 0.122 0.060

***** Estimates of phenotypic covariances ***********************************

Covariance matrix

1 36.091

2 0.15547 2.8676

Eigenvalues of covariance matrix

Value 36.09 2.87

(%) 92.64 7.36

Trace 38.96

Correlation matrix

1 1.0000

2 0.0153 1.0000

Covariances & correlations with approximate sampling errors

7 COVS T 1 1 36.0911 0.971976

8 COVS T 1 2 0.155470 0.372817 corr 0.015 0.037

9 COVS T 2 2 2.86760 0.141234

======== end of file ============================06-02-2015==========15:58====

======= Version 30-08-2013 ======================================= **KM** ====

Program WOMBAT: Summary of Pedigree Information

==============================================================================

Two traits analysis (LEA x RIGb)

Analysis type : "muv 2"

Data file : "DADOS.dat"

Pedigree file : "ReducedPedFile.dat"

Parameter file : "wombat.par"

No. of animal IDs in data file = = 2350

No. of animal IDs in total = = 3729

*****Pedigree Structure for random effect : 1 ****************************

Original no. of animals = 3729

No. of animals after pruning = 3638

... proportion (%) remaining = 97.6

No. of levels w/out records = 1288

No. of levels with records = 2350 100.0%

... 1 record(s) = 1523 64.8%

... 2 record(s) = 827 35.2%

No. of animals w/out offspring = 1889 51.9%

No. of animals with offspring = 1749 48.1%

... and records = 461 12.7%

No. of animals with unknown sire = 64

No. of animals with unknown dam = 161

No. of animals with both parents unknown = 64

No. of animals with records =

... and unknown sire = 0

... and unknown dam = 0

... and both parents unknown = 0

No. of sires = 313

... with progeny in the data = 180

... with records & progeny in data = 84

No. of dams = 1436

... with progeny in the data = 1036

... with records & progeny in data = 371

No. of animals with known/unpruned grand-parents

... with paternal grandsire = 3493

... with paternal granddam = 3355

... with maternal grandsire = 3364

... with maternal granddam = 3240

Inbreeding coefficients for random effect 1 computed

No. of inbred animals = 3107

Average inbreeding coefficient = 2.3747 (in %)

... amongst inbred animals = 2.7805 (in %)

random effect no. = 1 NRM

no. of elements in NRM/GIN inverse 13937

log determinant = -2503.1878537727198

======== end of file ============================06-02-2015==========16:02====

======= Version 30-08-2013 ======================================= **KM** ====

Program WOMBAT: Summary of information from Set-up step

==============================================================================

Analysis type : "muv 2"

Data file : "DADOS.dat"

Pedigree file : "ReducedPedFile.dat"

Parameter file : "wombat.par"

No. of traits = 2

nrec mean sdev min. max.

1 "LEA" 2283 51.4305 8.89390 21.4000 83.4000

2 "RIGb" 894 0.424196E-01 1.70140 -6.35080 4.85930

Numbers of individuals/records for pairs of traits

1 2

1 "LEA" 2283 827

2 "RIGb" 827 894

Covariables

1"LEA" nrec mean sdev min. max.

1 "idv(2)" 2283 6.11082 2.92852 2.00000 17.0000

2 "idade(1)" 2283 448.951 98.8881 306.000 609.000

2"RIGb" nrec mean sdev min. max.

1 "idv(2)" 894 6.16443 3.04994 3.00000 17.0000

2 "idade(1)" 894 368.964 35.9309 267.000 511.000

Fixed effects

1 "LEA" nlev

1 "gc" 65

2 "mn" 4

2 "RIGb" nlev

1 "gc" 21

Random effects nlev

1 "animal" 3638 NRM

======== end of file ============================06-02-2015==========16:02====

======= Version 30-08-2013 ======================================= **KM** ====

Program WOMBAT: Estimates of covariance components

==============================================================================

Analysis type : "muv 2"

Data file : "DADOS.dat"

Pedigree file : "ReducedPedFile.dat"

Parameter file : "wombat.par"

No. of traits = 2 LEA RIGb

No. of records = 3177 2283 894

No. of parameters = 6

Maximum log L = -6015.128

-1/2 AIC & AICC = -6021.128 -6021.141

-1/2 BIC = -6039.228 "Penalty factor" = 4.017

Parameter estimates with approx. sampling erors

1 CHOL Z 1 1 4.45284 0.174750

2 CHOL Z 1 2 -0.141029 0.102288

3 CHOL Z 2 2 1.59491 0.586415E-01

4 CHOL A 1 1 1.41177 0.681037E-01

5 CHOL A 1 2 0.154260 0.127037

6 CHOL A 2 2 -0.651077 0.309884

Convergence criteria for last 3 iterates

Change in log likelihood = 0.267586 0.001656 0.000020

Change in parameter vector = 0.017107 0.002702 0.000254

Norm of gradient vector = 14.1568 0.4162 0.0387

Newton decrement = -0.5148 -0.0030 -0.0000

***** Estimates of residual covariances ************************************

Order of fit = 2

Covariance matrix

1 19.828

2 -0.62798 2.5636

Eigenvalues of covariance matrix

Value 19.85 2.54

(%) 88.65 11.35

Trace 22.39

Matrix of correlations and variance ratios

1 0.5408

2 -0.0881 0.8966

Covariances & correlations with approximate sampling errors

1 COVS Z 1 1 19.8278 1.55627 vrat 0.541 0.051

2 COVS Z 1 2 -0.627982 0.454793 corr -0.088 0.064

3 COVS Z 2 2 2.56363 0.185572 vrat 0.897 0.057

***** Estimates for RE 1 "animal" ***************************************

No. of levels = 3638

Covariance structure = NRM

Order of fit = 2

Covariance matrix

1 16.837

2 0.63296 0.29574

Eigenvalues of covariance matrix

Value 16.86 0.27

(%) 98.41 1.59

Trace 17.13

Matrix of correlations and variance ratios

1 0.4592

2 0.2837 0.1034

Covariances & correlations with approximate sampling errors

4 COVS A 1 1 16.8365 2.29326 vrat 0.459 0.051

5 COVS A 1 2 0.632963 0.522452 corr 0.284 0.241

6 COVS A 2 2 0.295741 0.167251 vrat 0.103 0.057

***** Estimates of phenotypic covariances ***********************************

Covariance matrix

1 36.664

2 0.49809E-02 2.8594

Eigenvalues of covariance matrix

Value 36.66 2.86

(%) 92.77 7.23

Trace 39.52

Correlation matrix

1 1.0000

2 0.0005 1.0000

Covariances & correlations with approximate sampling errors

7 COVS T 1 1 36.6643 1.36163

8 COVS T 1 2 0.498092E-02 0.387047 corr 0.000 0.038

9 COVS T 2 2 2.85938 0.139882

======== end of file ============================06-02-2015==========16:02====

======= Version 30-08-2013 ======================================= **KM** ====

Program WOMBAT: Summary of Pedigree Information

==============================================================================

Two traits analysis (BF x RIGb)

Analysis type : "muv 2"

Data file : "DADOS.dat"

Pedigree file : "ReducedPedFile.dat"

Parameter file : "wombat.par"

No. of animal IDs in data file = = 2350

No. of animal IDs in total = = 3729

*****Pedigree Structure for random effect : 1 ****************************

Original no. of animals = 3729

No. of animals after pruning = 3638

... proportion (%) remaining = 97.6

No. of levels w/out records = 1288

No. of levels with records = 2350 100.0%

... 1 record(s) = 1521 64.7%

... 2 record(s) = 829 35.3%

No. of animals w/out offspring = 1889 51.9%

No. of animals with offspring = 1749 48.1%

... and records = 461 12.7%

No. of animals with unknown sire = 64

No. of animals with unknown dam = 161

No. of animals with both parents unknown = 64

No. of animals with records =

... and unknown sire = 0

... and unknown dam = 0

... and both parents unknown = 0

No. of sires = 313

... with progeny in the data = 180

... with records & progeny in data = 84

No. of dams = 1436

... with progeny in the data = 1036

... with records & progeny in data = 371

No. of animals with known/unpruned grand-parents

... with paternal grandsire = 3493

... with paternal granddam = 3355

... with maternal grandsire = 3364

... with maternal granddam = 3240

Inbreeding coefficients for random effect 1 computed

No. of inbred animals = 3107

Average inbreeding coefficient = 2.3747 (in %)

... amongst inbred animals = 2.7805 (in %)

random effect no. = 1 NRM

no. of elements in NRM/GIN inverse 13937

log determinant = -2503.1878537727198

======== end of file ============================06-02-2015==========16:04====

======= Version 30-08-2013 ======================================= **KM** ====

Program WOMBAT: Summary of information from Set-up step

==============================================================================

Analysis type : "muv 2"

Data file : "DADOS.dat"

Pedigree file : "ReducedPedFile.dat"

Parameter file : "wombat.par"

No. of traits = 2

nrec mean sdev min. max.

1 "BF" 2285 1.75922 1.42798 0.00000 10.5000

2 "RIGb" 894 0.424196E-01 1.70140 -6.35080 4.85930

Numbers of individuals/records for pairs of traits

1 2

1 "BF" 2285 829

2 "RIGb" 829 894

Covariables

1"BF" nrec mean sdev min. max.

1 "idv(2)" 2285 6.10810 2.92868 2.00000 17.0000

2 "idade(1)" 2285 449.081 98.9421 306.000 609.000

2"RIGb" nrec mean sdev min. max.

1 "idv(2)" 894 6.16443 3.04994 3.00000 17.0000

2 "idade(1)" 894 368.964 35.9309 267.000 511.000

Fixed effects

1 "BF" nlev

1 "gc" 65

2 "mn" 4

2 "RIGb" nlev

1 "gc" 21

Random effects nlev

1 "animal" 3638 NRM

======== end of file ============================06-02-2015==========16:04====

======= Version 30-08-2013 ======================================= **KM** ====

Program WOMBAT: Estimates of covariance components

==============================================================================

Analysis type : "muv 2"

Data file : "DADOS.dat"

Pedigree file : "ReducedPedFile.dat"

Parameter file : "wombat.par"

No. of traits = 2 BF RIGb

No. of records = 3179 2285 894

No. of parameters = 6

Maximum log L = -2005.826

-1/2 AIC & AICC = -2011.826 -2011.839

-1/2 BIC = -2029.928 "Penalty factor" = 4.017

Parameter estimates with approx. sampling erors

1 CHOL Z 1 1 1.60753 0.577889E-01

2 CHOL Z 1 2 0.139977E-01 0.433143E-01

3 CHOL Z 2 2 0.800025 0.235231E-01

4 CHOL A 1 1 -0.660544 0.308394

5 CHOL A 1 2 -0.242827 0.139995

6 CHOL A 2 2 -0.752425 0.175226

Convergence criteria for last 3 iterates

Change in log likelihood = 0.038878 0.000767 0.000006

Change in parameter vector = 0.020406 0.002725 0.000297

Norm of gradient vector = 5.6705 1.6341 0.0345

Newton decrement = -0.0836 -0.0016 -0.0000

***** Estimates of residual covariances ************************************

Order of fit = 2

Covariance matrix

1 0.64024

2 0.22502E-01 2.5842

Eigenvalues of covariance matrix

Value 2.58 0.64

(%) 80.15 19.85

Trace 3.22

Matrix of correlations and variance ratios

1 0.6950

2 0.0175 0.9064

Covariances & correlations with approximate sampling errors

1 COVS Z 1 1 0.640236 0.375630E-01 vrat 0.695 0.046

2 COVS Z 1 2 0.225017E-01 0.695908E-01 corr 0.017 0.054

3 COVS Z 2 2 2.58415 0.185795 vrat 0.906 0.057

***** Estimates for RE 1 "animal" ***************************************

No. of levels = 3638

Covariance structure = NRM

Order of fit = 2

Covariance matrix

1 0.28102

2 -0.12544 0.26684

Eigenvalues of covariance matrix

Value 0.40 0.15

(%) 72.93 27.07

Trace 0.55

Matrix of correlations and variance ratios

1 0.3050

2 -0.4581 0.0936

Covariances & correlations with approximate sampling errors

4 COVS A 1 1 0.281016 0.475965E-01 vrat 0.305 0.046

5 COVS A 1 2 -0.125437 0.673561E-01 corr -0.458 0.262

6 COVS A 2 2 0.266845 0.164587 vrat 0.094 0.057

***** Estimates of phenotypic covariances ***********************************

Covariance matrix

1 0.92125

2 -0.10294 2.8510

Eigenvalues of covariance matrix

Value 2.86 0.92

(%) 75.72 24.28

Trace 3.77

Correlation matrix

1 1.0000

2 -0.0635 1.0000

Covariances & correlations with approximate sampling errors

7 COVS T 1 1 0.921252 0.312941E-01

8 COVS T 1 2 -0.102936 0.579375E-01 corr -0.064 0.036

9 COVS T 2 2 2.85100 0.139003

======== end of file ============================06-02-2015==========16:04====

======= Version 30-08-2013 ======================================= **KM** ====

Program WOMBAT: Summary of Pedigree Information

==============================================================================

Two traits analysis (RF x RIGb)

Analysis type : "muv 2"

Data file : "DADOS.dat"

Pedigree file : "ReducedPedFile.dat"

Parameter file : "wombat.par"

No. of animal IDs in data file = = 1884

No. of animal IDs in total = = 3147

*****Pedigree Structure for random effect : 1 ****************************

Original no. of animals = 3147

No. of animals after pruning = 3062

... proportion (%) remaining = 97.3

No. of levels w/out records = 1178

No. of levels with records = 1884 100.0%

... 1 record(s) = 1057 56.1%

... 2 record(s) = 827 43.9%

No. of animals w/out offspring = 1473 48.1%

No. of animals with offspring = 1589 51.9%

... and records = 411 13.4%

No. of animals with unknown sire = 60

No. of animals with unknown dam = 147

No. of animals with both parents unknown = 59

No. of animals with records =

... and unknown sire = 0

... and unknown dam = 0

... and both parents unknown = 0

No. of sires = 307

... with progeny in the data = 114

... with records & progeny in data = 43

No. of dams = 1282

... with progeny in the data = 783

... with records & progeny in data = 368

No. of animals with known/unpruned grand-parents

... with paternal grandsire = 2938

... with paternal granddam = 2812

... with maternal grandsire = 2813

... with maternal granddam = 2724

Inbreeding coefficients for random effect 1 computed

No. of inbred animals = 2616

Average inbreeding coefficient = 2.5605 (in %)

... amongst inbred animals = 2.9970 (in %)

random effect no. = 1 NRM

no. of elements in NRM/GIN inverse 11700

log determinant = -2105.0285008830233

======== end of file ============================06-02-2015==========16:08====

======= Version 30-08-2013 ======================================= **KM** ====

Program WOMBAT: Summary of information from Set-up step

==============================================================================

Analysis type : "muv 2"

Data file : "DADOS.dat"

Pedigree file : "ReducedPedFile.dat"

Parameter file : "wombat.par"

No. of traits = 2

nrec mean sdev min. max.

1 "RF" 1817 5.08663 2.54341 0.00000 19.2000

2 "RIGb" 894 0.424196E-01 1.70140 -6.35080 4.85930

Numbers of individuals/records for pairs of traits

1 2

1 "RF" 1817 827

2 "RIGb" 827 894

Covariables

1"RF" nrec mean sdev min. max.

1 "idv(2)" 1817 6.09191 3.03138 2.00000 17.0000

2 "idade(1)" 1817 469.100 100.703 306.000 609.000

2"RIGb" nrec mean sdev min. max.

1 "idv(2)" 894 6.16443 3.04994 3.00000 17.0000

2 "idade(1)" 894 368.964 35.9309 267.000 511.000

Fixed effects

1 "RF" nlev

1 "gc" 51

2 "mn" 3

2 "RIGb" nlev

1 "gc" 21

Random effects nlev

1 "animal" 3062 NRM

======== end of file ============================06-02-2015==========16:08====

======= Version 30-08-2013 ======================================= **KM** ====

Program WOMBAT: Estimates of covariance components

==============================================================================

Analysis type : "muv 2"

Data file : "DADOS.dat"

Pedigree file : "ReducedPedFile.dat"

Parameter file : "wombat.par"

No. of traits = 2 RF RIGb

No. of records = 2711 1817 894

No. of parameters = 6

Maximum log L = -2678.742

-1/2 AIC & AICC = -2684.742 -2684.758

-1/2 BIC = -2702.368 "Penalty factor" = 3.938

Parameter estimates with approx. sampling erors

1 CHOL Z 1 1 1.60905 0.582350E-01

2 CHOL Z 1 2 0.552345E-01 0.736941E-01

3 CHOL Z 2 2 1.26430 0.465327E-01

4 CHOL A 1 1 0.897451E-03 0.804830E-01

5 CHOL A 1 2 -0.215022 0.121190

6 CHOL A 2 2 -0.766576 0.389625

Convergence criteria for last 3 iterates

Change in log likelihood = 9.841208 0.065637 0.000096

Change in parameter vector = 0.076656 0.016200 0.002095

Norm of gradient vector = 179.9934 13.0415 0.1131

Newton decrement = -18.8970 -0.1301 -0.0002

***** Estimates of residual covariances ************************************

Order of fit = 2

Covariance matrix

1 1.6015

2 0.88875E-01 2.5891

Eigenvalues of covariance matrix

Value 2.60 1.59

(%) 61.97 38.03

Trace 4.19

Matrix of correlations and variance ratios

1 0.6152

2 0.0436 0.9081

Covariances & correlations with approximate sampling errors

1 COVS Z 1 1 1.60152 0.116964 vrat 0.615 0.053

2 COVS Z 1 2 0.888752E-01 0.118457 corr 0.044 0.058

3 COVS Z 2 2 2.58906 0.187407 vrat 0.908 0.057

***** Estimates for RE 1 "animal" ***************************************

No. of levels = 3062

Covariance structure = NRM

Order of fit = 2

Covariance matrix

1 1.0018

2 -0.21521 0.26209

Eigenvalues of covariance matrix

Value 1.06 0.20

(%) 83.86 16.14

Trace 1.26

Matrix of correlations and variance ratios

1 0.3848

2 -0.4200 0.0919

Covariances & correlations with approximate sampling errors

4 COVS A 1 1 1.00180 0.161255 vrat 0.385 0.053

5 COVS A 1 2 -0.215215 0.122807 corr -0.420 0.257

6 COVS A 2 2 0.262089 0.166637 vrat 0.092 0.057

***** Estimates of phenotypic covariances ***********************************

Covariance matrix

1 2.6033

2 -0.12634 2.8511

Eigenvalues of covariance matrix

Value 2.90 2.55

(%) 53.24 46.76

Trace 5.45

Correlation matrix

1 1.0000

2 -0.0464 1.0000

Covariances & correlations with approximate sampling errors

7 COVS T 1 1 2.60331 0.102976

8 COVS T 1 2 -0.126340 0.994239E-01 corr -0.046 0.036

9 COVS T 2 2 2.85114 0.139054

======== end of file ============================06-02-2015==========16:09====

======= Version 30-08-2013 ======================================= **KM** ====

Program WOMBAT: Summary of Pedigree Information

==============================================================================

Two traits analysis (RIGsf x DMI)

Analysis type : "muv 2"

Data file : "DADOS.dat"

Pedigree file : "ReducedPedFile.dat"

Parameter file : "wombat.par"

No. of animal IDs in data file = = 955

No. of animal IDs in total = = 2288

*****Pedigree Structure for random effect : 1 ****************************

Original no. of animals = 2288

No. of animals after pruning = 2204

... proportion (%) remaining = 96.3

No. of levels w/out records = 1249

No. of levels with records = 955 100.0%

... 1 record(s) = 64 6.7%

... 2 record(s) = 891 93.3%

No. of animals w/out offspring = 828 37.6%

No. of animals with offspring = 1376 62.4%

... and records = 127 5.8%

No. of animals with unknown sire = 59

No. of animals with unknown dam = 145

No. of animals with both parents unknown = 58

No. of animals with records =

... and unknown sire = 0

... and unknown dam = 0

... and both parents unknown = 0

No. of sires = 296

... with progeny in the data = 78

... with records & progeny in data = 18

No. of dams = 1080

... with progeny in the data = 542

... with records & progeny in data = 98

No. of animals with known/unpruned grand-parents

... with paternal grandsire = 2083

... with paternal granddam = 1957

... with maternal grandsire = 1959

... with maternal granddam = 1872

random effect no. = 1 NRM

no. of elements in NRM/GIN inverse 8375

log determinant = -1487.8684515369519

======== end of file ============================25-02-2015==========18:20====

======= Version 30-08-2013 ======================================= **KM** ====

Program WOMBAT: Summary of information from Set-up step

==============================================================================

Analysis type : "muv 2"

Data file : "DADOS.dat"

Pedigree file : "ReducedPedFile.dat"

Parameter file : "wombat.par"

No. of traits = 2

nrec mean sdev min. max.

1 "RIGsf" 891 0.436001E-01 1.69595 -5.94410 4.80400

2 "DMI" 955 6.83104 1.33500 2.15500 12.6400

Numbers of individuals/records for pairs of traits

1 2

1 "RIGsf" 891 891

2 "DMI" 891 955

Covariables

1"RIGsf" nrec mean sdev min. max.

1 "idv(2)" 891 6.16611 3.04967 3.00000 17.0000

2 "idade(1)" 891 368.979 35.9742 267.000 511.000

2"DMI" nrec mean sdev min. max.

1 "idv(2)" 955 6.15079 3.05304 3.00000 17.0000

2 "idade(1)" 955 369.980 37.3543 267.000 511.000

Fixed effects

1 "RIGsf" nlev

1 "gc" 21

2 "DMI" nlev

1 "gc" 21

Random effects nlev

1 "animal" 2204 NRM

======== end of file ============================25-02-2015==========18:20====

======= Version 30-08-2013 ======================================= **KM** ====

Program WOMBAT: Estimates of covariance components

==============================================================================

Analysis type : "muv 2"

Data file : "DADOS.dat"

Pedigree file : "ReducedPedFile.dat"

Parameter file : "wombat.par"

No. of traits = 2 RIGsf DMI

No. of records = 1846 891 955

No. of parameters = 6

Maximum log L = -1196.365

-1/2 AIC & AICC = -1202.365 -1202.388

-1/2 BIC = -1218.848 "Penalty factor" = 3.747

Parameter estimates with approx. sampling erors

1 CHOL Z 1 1 1.59345 0.590842E-01

2 CHOL Z 1 2 -0.330136 0.381443E-01

3 CHOL Z 2 2 0.556297 0.356760E-01

4 CHOL A 1 1 -0.610335 0.293189

5 CHOL A 1 2 -0.170199 0.119102

6 CHOL A 2 2 -0.549743 0.865026E-01

Convergence criteria for last 3 iterates

Change in log likelihood = 0.312970 0.000645 0.000030

Change in parameter vector = 0.027735 0.003272 0.000724

Norm of gradient vector = 21.0216 0.5099 0.0784

Newton decrement = -0.6201 -0.0015 -0.0001

***** Estimates of residual covariances ************************************

Order of fit = 2

Covariance matrix

1 2.5391

2 -0.52606 0.41846

Eigenvalues of covariance matrix

Value 2.66 0.30

(%) 90.02 9.98

Trace 2.96

Matrix of correlations and variance ratios

1 0.8959

2 -0.5103 0.5362

Covariances & correlations with approximate sampling errors

1 COVS Z 1 1 2.53909 0.188296 vrat 0.896 0.060

2 COVS Z 1 2 -0.526055 0.692598E-01 corr -0.510 0.050

3 COVS Z 2 2 0.418456 0.468960E-01 vrat 0.536 0.067

***** Estimates for RE 1 "animal" ***************************************

No. of levels = 2204

Covariance structure = NRM

Order of fit = 2

Covariance matrix

1 0.29503

2 -0.92447E-01 0.36201

Eigenvalues of covariance matrix

Value 0.43 0.23

(%) 64.96 35.04

Trace 0.66

Matrix of correlations and variance ratios

1 0.1041

2 -0.2829 0.4638

Covariances & correlations with approximate sampling errors

4 COVS A 1 1 0.295032 0.173001 vrat 0.104 0.060

5 COVS A 1 2 -0.924469E-01 0.729843E-01 corr -0.283 0.188

6 COVS A 2 2 0.362010 0.635593E-01 vrat 0.464 0.067

***** Estimates of phenotypic covariances ***********************************

Covariance matrix

1 2.8341

2 -0.61850 0.78047

Eigenvalues of covariance matrix

Value 3.01 0.61

(%) 83.16 16.84

Trace 3.61

Correlation matrix

1 1.0000

2 -0.4159 1.0000

Covariances & correlations with approximate sampling errors

7 COVS T 1 1 2.83412 0.138899

8 COVS T 1 2 -0.618502 0.554581E-01 corr -0.416 0.029

9 COVS T 2 2 0.780466 0.406748E-01

======== end of file ============================25-02-2015==========18:20====

======= Version 30-08-2013 ======================================= **KM** ====

Program WOMBAT: Summary of Pedigree Information

==============================================================================

Two traits analysis (RIGsf x ADG)

Analysis type : "muv 2"

Data file : "DADOS.dat"

Pedigree file : "ReducedPedFile.dat"

Parameter file : "wombat.par"

No. of animal IDs in data file = = 955

No. of animal IDs in total = = 2288

*****Pedigree Structure for random effect : 1 ****************************

Original no. of animals = 2288

No. of animals after pruning = 2204

... proportion (%) remaining = 96.3

No. of levels w/out records = 1249

No. of levels with records = 955 100.0%

... 1 record(s) = 64 6.7%

... 2 record(s) = 891 93.3%

No. of animals w/out offspring = 828 37.6%

No. of animals with offspring = 1376 62.4%

... and records = 127 5.8%

No. of animals with unknown sire = 59

No. of animals with unknown dam = 145

No. of animals with both parents unknown = 58

No. of animals with records =

... and unknown sire = 0

... and unknown dam = 0

... and both parents unknown = 0

No. of sires = 296

... with progeny in the data = 78

... with records & progeny in data = 18

No. of dams = 1080

... with progeny in the data = 542

... with records & progeny in data = 98

No. of animals with known/unpruned grand-parents

... with paternal grandsire = 2083

... with paternal granddam = 1957

... with maternal grandsire = 1959

... with maternal granddam = 1872

random effect no. = 1 NRM

no. of elements in NRM/GIN inverse 8375

log determinant = -1487.8684515369519

======== end of file ============================25-02-2015==========18:22====

======= Version 30-08-2013 ======================================= **KM** ====

Program WOMBAT: Summary of information from Set-up step

==============================================================================

Analysis type : "muv 2"

Data file : "DADOS.dat"

Pedigree file : "ReducedPedFile.dat"

Parameter file : "wombat.par"

No. of traits = 2

nrec mean sdev min. max.

1 "RIGsf" 891 0.436001E-01 1.69595 -5.94410 4.80400

2 "ADG" 955 1.00846 0.260202 0.176000 1.71800

Numbers of individuals/records for pairs of traits

1 2

1 "RIGsf" 891 891

2 "ADG" 891 955

Covariables

1"RIGsf" nrec mean sdev min. max.

1 "idv(2)" 891 6.16611 3.04967 3.00000 17.0000

2 "idade(1)" 891 368.979 35.9742 267.000 511.000

2"ADG" nrec mean sdev min. max.

1 "idv(2)" 955 6.15079 3.05304 3.00000 17.0000

2 "idade(1)" 955 369.980 37.3543 267.000 511.000

Fixed effects

1 "RIGsf" nlev

1 "gc" 21

2 "ADG" nlev

1 "gc" 21

Random effects nlev

1 "animal" 2204 NRM

======== end of file ============================25-02-2015==========18:22====

======= Version 30-08-2013 ======================================= **KM** ====

Program WOMBAT: Estimates of covariance components

==============================================================================

Analysis type : "muv 2"

Data file : "DADOS.dat"

Pedigree file : "ReducedPedFile.dat"

Parameter file : "wombat.par"

No. of traits = 2 RIGsf ADG

No. of records = 1846 891 955

No. of parameters = 6

Maximum log L = 382.767

-1/2 AIC & AICC = 376.767 376.745

-1/2 BIC = 360.284 "Penalty factor" = 3.747

Parameter estimates with approx. sampling erors

1 CHOL Z 1 1 0.456561 0.376714E-01

2 CHOL Z 1 2 0.546086E-01 0.696731E-02

3 CHOL Z 2 2 -2.23657 0.570460E-01

4 CHOL A 1 1 -0.538857 0.260776

5 CHOL A 1 2 0.129166E-01 0.209847E-01

6 CHOL A 2 2 -2.30370 0.892136E-01

Convergence criteria for last 3 iterates

Change in log likelihood = 0.009621 0.000847 0.000085

Change in parameter vector = 0.002750 0.000500 0.000205

Norm of gradient vector = 7.1443 2.4624 0.7564

Newton decrement = -0.0267 -0.0025 -0.0003

***** Estimates of residual covariances ************************************

Order of fit = 2

Covariance matrix

1 2.4921

2 0.86207E-01 0.14394E-01

Eigenvalues of covariance matrix

Value 2.50 0.01

(%) 99.55 0.45

Trace 2.51

Matrix of correlations and variance ratios

1 0.8798

2 0.4552 0.5866

Covariances & correlations with approximate sampling errors

1 COVS Z 1 1 2.49209 0.187761 vrat 0.880 0.061

2 COVS Z 1 2 0.862071E-01 0.122960E-01 corr 0.455 0.051

3 COVS Z 2 2 0.143935E-01 0.147721E-02 vrat 0.587 0.067

***** Estimates for RE 1 "animal" ***************************************

No. of levels = 2204

Covariance structure = NRM

Order of fit = 2

Covariance matrix

1 0.34037

2 0.75358E-02 0.10145E-01

Eigenvalues of covariance matrix

Value 0.34 0.01

(%) 97.15 2.85

Trace 0.35

Matrix of correlations and variance ratios

1 0.1202

2 0.1282 0.4134

Covariances & correlations with approximate sampling errors

4 COVS A 1 1 0.340373 0.177522 vrat 0.120 0.061

5 COVS A 1 2 0.753575E-02 0.130010E-01 corr 0.128 0.204

6 COVS A 2 2 0.101447E-01 0.191222E-02 vrat 0.413 0.067

***** Estimates of phenotypic covariances ***********************************

Covariance matrix

1 2.8325

2 0.93743E-01 0.24538E-01

Eigenvalues of covariance matrix

Value 2.84 0.02

(%) 99.25 0.75

Trace 2.86

Correlation matrix

1 1.0000

2 0.3556 1.0000

Covariances & correlations with approximate sampling errors

7 COVS T 1 1 2.83246 0.139364

8 COVS T 1 2 0.937428E-01 0.968248E-02 corr 0.356 0.031

9 COVS T 2 2 0.245382E-01 0.125678E-02

======== end of file ============================25-02-2015==========18:22====

======= Version 30-08-2013 ======================================= **KM** ====

Program WOMBAT: Summary of Pedigree Information

==============================================================================

Two traits analysis (WS x RIGsf)

Analysis type : "muv 2"

Data file : "DADOS.dat"

Pedigree file : "ReducedPedFile.dat"

Parameter file : "wombat.par"

No. of animal IDs in data file = = 8090

No. of animal IDs in total = = 8489

*****Pedigree Structure for random effect : 1 ****************************

Original no. of animals = 8489

No. of animals after pruning = 8432

... proportion (%) remaining = 99.3

No. of levels w/out records = 342

No. of levels with records = 8090 100.0%

... 1 record(s) = 7211 89.1%

... 2 record(s) = 879 10.9%

No. of animals w/out offspring = 6051 71.8%

No. of animals with offspring = 2381 28.2%

... and records = 2039 24.2%

No. of animals with unknown sire = 313

No. of animals with unknown dam = 384

No. of animals with both parents unknown = 312

No. of animals with records =

... and unknown sire = 1

... and unknown dam = 53

... and both parents unknown = 0

No. of sires = 325

... with progeny in the data = 320

... with records & progeny in data = 288

No. of dams = 2056

... with progeny in the data = 2056

... with records & progeny in data = 1751

No. of animals with known/unpruned grand-parents

... with paternal grandsire = 7357

... with paternal granddam = 7055

... with maternal grandsire = 6534

... with maternal granddam = 6467

Inbreeding coefficients for random effect 1 computed

No. of inbred animals = 5143

Average inbreeding coefficient = 1.5416 (in %)

... amongst inbred animals = 2.5274 (in %)

random effect no. = 1 NRM

no. of elements in NRM/GIN inverse 31835

log determinant = -5688.0517714250827

random effect no. = 2 IDE

no. of elements in NRM/GIN inverse 0

log determinant = 0.0000000000000000

======== end of file ============================06-02-2015==========16:12====

======= Version 30-08-2013 ======================================= **KM** ====

Program WOMBAT: Summary of information from Set-up step

==============================================================================

Analysis type : "muv 2"

Data file : "DADOS.dat"

Pedigree file : "ReducedPedFile.dat"

Parameter file : "wombat.par"

No. of traits = 2

nrec mean sdev min. max.

1 "WS" 8078 299.703 49.6462 160.030 489.760

2 "RIGsf" 891 0.436001E-01 1.69595 -5.94410 4.80400

Numbers of individuals/records for pairs of traits

1 2

1 "WS" 8078 879

2 "RIGsf" 879 891

Covariables

1"WS" nrec mean sdev min. max.

1 "idv(2)" 8078 6.63568 3.07616 2.00000 18.0000

2 "idade(1)" 8078 473.414 105.028 293.000 645.000

2"RIGsf" nrec mean sdev min. max.

1 "idv(2)" 891 6.16611 3.04967 3.00000 17.0000

2 "idade(1)" 891 368.979 35.9742 267.000 511.000

Fixed effects

1 "WS" nlev

1 "gc" 201

2 "mn" 4

2 "RIGsf" nlev

1 "gc" 21

Random effects nlev

1 "animal" 8432 NRM

2 "peanim" 2107 IDE

======== end of file ============================06-02-2015==========16:12====

======= Version 30-08-2013 ======================================= **KM** ====

Program WOMBAT: Estimates of covariance components

==============================================================================

Analysis type : "muv 2"

Data file : "DADOS.dat"

Pedigree file : "ReducedPedFile.dat"

Parameter file : "wombat.par"

No. of traits = 2 WS RIGsf

No. of records = 8969 8078 891

No. of parameters = 7

Maximum log L = -31092.482

-1/2 AIC & AICC = -31099.482 -31099.488

-1/2 BIC = -31124.246 "Penalty factor" = 4.538

Parameter estimates with approx. sampling erors

1 CHOL Z 1 1 20.6356 0.434628

2 CHOL Z 1 2 0.924237E-01 0.844644E-01

3 CHOL Z 2 2 1.57794 0.594722E-01

4 CHOL A 1 1 2.87811 0.482489E-01

5 CHOL A 1 2 0.438482E-01 0.102994

6 CHOL A 2 2 -0.534480 0.258636

7 CHOL B 1 1 2.33413 0.504309E-01

Convergence criteria for last 3 iterates

Change in log likelihood = 0.106892 0.001334 0.000047

Change in parameter vector = 0.001224 0.000183 0.000073

Norm of gradient vector = 10.9572 0.9217 0.1490

Newton decrement = -0.2026 -0.0026 -0.0001

***** Estimates of residual covariances ************************************

Order of fit = 2

Covariance matrix

1 425.83

2 1.9072 2.4984

Eigenvalues of covariance matrix

Value 425.84 2.49

(%) 99.42 0.58

Trace 428.32

Matrix of correlations and variance ratios

1 0.5019

2 0.0585 0.8786

Covariances & correlations with approximate sampling errors

1 COVS Z 1 1 425.826 17.9376 vrat 0.502 0.027

2 COVS Z 1 2 1.90722 1.74395 corr 0.058 0.053

3 COVS Z 2 2 2.49843 0.188997 vrat 0.879 0.061

***** Estimates for RE 1 "animal" ***************************************

No. of levels = 8432

Covariance structure = NRM

Order of fit = 2

Covariance matrix

1 316.15

2 0.77965 0.34529

Eigenvalues of covariance matrix

Value 316.15 0.34

(%) 99.89 0.11

Trace 316.50

Matrix of correlations and variance ratios

1 0.3726

2 0.0746 0.1214

Covariances & correlations with approximate sampling errors

4 COVS A 1 1 316.152 30.5080 vrat 0.373 0.030

5 COVS A 1 2 0.779649 1.83224 corr 0.075 0.173

6 COVS A 2 2 0.345288 0.179061 vrat 0.121 0.061

***** Estimates for RE 2 "peanim" ***************************************

No. of levels = 2107

Covariance structure = IDE

Order of fit = 1

Covariance matrix

1 106.51

Matrix of correlations and variance ratios

1 0.1255

Covariances & correlations with approximate sampling errors

7 COVS B 1 1 106.512 10.7430 vrat 0.126 0.012

***** Estimates of phenotypic covariances ***********************************

Covariance matrix

1 848.49

2 2.6869 2.8437

Eigenvalues of covariance matrix

Value 848.50 2.84

(%) 99.67 0.33

Trace 851.33

Correlation matrix

1 1.0000

2 0.0547 1.0000

Covariances & correlations with approximate sampling errors

8 COVS T 1 1 848.490 18.5236

9 COVS T 1 2 2.68687 1.60333 corr 0.055 0.033

10 COVS T 2 2 2.84372 0.140286

======== end of file ============================06-02-2015==========16:12====

======= Version 30-08-2013 ======================================= **KM** ====

Program WOMBAT: Summary of Pedigree Information

==============================================================================

Two traits analysis (HH x RIGsf)

Analysis type : "muv 2"

Data file : "DADOS.dat"

Pedigree file : "ReducedPedFile.dat"

Parameter file : "wombat.par"

No. of animal IDs in data file = = 6559

No. of animal IDs in total = = 7099

*****Pedigree Structure for random effect : 1 ****************************

Original no. of animals = 7099

No. of animals after pruning = 7016

... proportion (%) remaining = 98.8

No. of levels w/out records = 457

No. of levels with records = 6559 100.0%

... 1 record(s) = 5679 86.6%

... 2 record(s) = 880 13.4%

No. of animals w/out offspring = 4828 68.8%

No. of animals with offspring = 2188 31.2%

... and records = 1731 24.7%

No. of animals with unknown sire = 148

No. of animals with unknown dam = 237

No. of animals with both parents unknown = 148

No. of animals with records =

... and unknown sire = 0

... and unknown dam = 18

... and both parents unknown = 0

No. of sires = 320

... with progeny in the data = 283

... with records & progeny in data = 240

No. of dams = 1868

... with progeny in the data = 1837

... with records & progeny in data = 1491

No. of animals with known/unpruned grand-parents

... with paternal grandsire = 6737

... with paternal granddam = 6483

... with maternal grandsire = 6338

... with maternal granddam = 6110

random effect no. = 1 NRM

no. of elements in NRM/GIN inverse 26819

log determinant = -4814.0947710995106

======== end of file ============================06-02-2015==========16:15====

======= Version 30-08-2013 ======================================= **KM** ====

Program WOMBAT: Summary of information from Set-up step

==============================================================================

Analysis type : "muv 2"

Data file : "DADOS.dat"

Pedigree file : "ReducedPedFile.dat"

Parameter file : "wombat.par"

No. of traits = 2

nrec mean sdev min. max.

1 "HH" 6548 132.269 5.43028 100.000 149.000

2 "RIGsf" 891 0.436001E-01 1.69595 -5.94410 4.80400

Numbers of individuals/records for pairs of traits

1 2

1 "alt" 6548 880

2 "RIGsf" 880 891

Covariables

1"HH" nrec mean sdev min. max.

1 "idv(2)" 6548 6.40043 2.93455 2.00000 17.0000

2 "idade(1)" 6548 472.701 107.333 293.000 645.000

2"RIGsf" nrec mean sdev min. max.

1 "idv(2)" 891 6.16611 3.04967 3.00000 17.0000

2 "idade(1)" 891 368.979 35.9742 267.000 511.000

Fixed effects

1 "HH" nlev

1 "gc" 169

2 "mn" 4

2 "RIGsf" nlev

1 "gc" 21

Random effects nlev

1 "animal" 7016 NRM

======== end of file ============================06-02-2015==========16:15====

======= Version 30-08-2013 ======================================= **KM** ====

Program WOMBAT: Estimates of covariance components

==============================================================================

Analysis type : "muv 2"

Data file : "DADOS.dat"

Pedigree file : "ReducedPedFile.dat"

Parameter file : "wombat.par"

No. of traits = 2 alt CGR

No. of records = 7439 6548 891

No. of parameters = 6

Maximum log L = -12341.888

-1/2 AIC & AICC = -12347.888 -12347.894

-1/2 BIC = -12368.550 "Penalty factor" = 4.444

Parameter estimates with approx. sampling erors

1 CHOL Z 1 1 2.44827 0.654513E-01

2 CHOL Z 1 2 -0.235946 0.105049

3 CHOL Z 2 2 1.55832 0.628934E-01

4 CHOL A 1 1 1.12228 0.315540E-01

5 CHOL A 1 2 0.917128E-01 0.103677

6 CHOL A 2 2 -0.513473 0.248845

Convergence criteria for last 3 iterates

Change in log likelihood = 0.174061 0.004316 0.000103

Change in parameter vector = 0.012631 0.001894 0.000317

Norm of gradient vector = 8.4767 2.2612 0.2688

Newton decrement = -0.3027 -0.0075 -0.0002

***** Estimates of residual covariances ************************************

Order of fit = 2

Covariance matrix

1 5.9940

2 -0.57766 2.4840

Eigenvalues of covariance matrix

Value 6.09 2.39

(%) 71.79 28.21

Trace 8.48

Matrix of correlations and variance ratios

1 0.3885

2 -0.1497 0.8714

Covariances & correlations with approximate sampling errors

1 COVS Z 1 1 5.99404 0.320485 vrat 0.388 0.026

2 COVS Z 1 2 -0.577659 0.256693 corr -0.150 0.067

3 COVS Z 2 2 2.48404 0.188771 vrat 0.871 0.062

***** Estimates for RE 1 "animal" ***************************************

No. of levels = 7016

Covariance structure = NRM

Order of fit = 2

Covariance matrix

1 9.4362

2 0.28173 0.36651

Eigenvalues of covariance matrix

Value 9.44 0.36

(%) 96.35 3.65

Trace 9.80

Matrix of correlations and variance ratios

1 0.6115

2 0.1515 0.1286

Covariances & correlations with approximate sampling errors

4 COVS A 1 1 9.43624 0.595502 vrat 0.612 0.026

5 COVS A 1 2 0.281727 0.318639 corr 0.151 0.168

6 COVS A 2 2 0.366510 0.181022 vrat 0.129 0.062

***** Estimates of phenotypic covariances ***********************************

Covariance matrix

1 15.430

2 -0.29593 2.8506

Eigenvalues of covariance matrix

Value 15.44 2.84

(%) 84.44 15.56

Trace 18.28

Correlation matrix

1 1.0000

2 -0.0446 1.0000

Covariances & correlations with approximate sampling errors

7 COVS T 1 1 15.4303 0.383634

8 COVS T 1 2 -0.295932 0.242626 corr -0.045 0.037

9 COVS T 2 2 2.85055 0.141050

======== end of file ============================06-02-2015==========16:15====

======= Version 30-08-2013 ======================================= **KM** ====

Program WOMBAT: Summary of Pedigree Information

==============================================================================

Two traits analysis (CC x RIGsf)

Analysis type : "muv 2"

Data file : "DADOS.dat"

Pedigree file : "ReducedPedFile.dat"

Parameter file : "wombat.par"

No. of animal IDs in data file = = 3941

No. of animal IDs in total = = 5416

*****Pedigree Structure for random effect : 1 ****************************

Original no. of animals = 5416

No. of animals after pruning = 5328

... proportion (%) remaining = 98.4

No. of levels w/out records = 1387

No. of levels with records = 3941 100.0%

... 1 record(s) = 3115 79.0%

... 2 record(s) = 826 21.0%

No. of animals w/out offspring = 3309 62.1%

No. of animals with offspring = 2019 37.9%

... and records = 632 11.9%

No. of animals with unknown sire = 94

No. of animals with unknown dam = 188

No. of animals with both parents unknown = 94

No. of animals with records =

... and unknown sire = 0

... and unknown dam = 0

... and both parents unknown = 0

No. of sires = 318

... with progeny in the data = 255

... with records & progeny in data = 217

No. of dams = 1701

... with progeny in the data = 1508

... with records & progeny in data = 412

No. of animals with known/unpruned grand-parents

... with paternal grandsire = 5140

... with paternal granddam = 4952

... with maternal grandsire = 4951

... with maternal granddam = 4736

Inbreeding coefficients for random effect 1 computed

No. of inbred animals = 4322

Average inbreeding coefficient = 2.1289 (in %)

... amongst inbred animals = 2.6244 (in %)

random effect no. = 1 NRM

no. of elements in NRM/GIN inverse 20457

log determinant = -3670.4017279806212

======== end of file ============================06-02-2015==========16:17====

======= Version 30-08-2013 ======================================= **KM** ====

Program WOMBAT: Summary of information from Set-up step

==============================================================================

Analysis type : "muv 2"

Data file : "DADOS.dat"

Pedigree file : "ReducedPedFile.dat"

Parameter file : "wombat.par"

No. of traits = 2

nrec mean sdev min. max.

1 "CC" 3876 164.140 8.72028 128.000 192.000

2 "RIGsf" 891 0.436001E-01 1.69595 -5.94410 4.80400

Numbers of individuals/records for pairs of traits

1 2

1 "CC" 3876 826

2 "RIGsf" 826 891

Covariables

1"CC" nrec mean sdev min. max.

1 "idv(2)" 3876 6.27632 2.87660 2.00000 17.0000

2 "idade(1)" 3876 431.403 109.187 293.000 725.000

2"RIGsf" nrec mean sdev min. max.

1 "idv(2)" 891 6.16611 3.04967 3.00000 17.0000

2 "idade(1)" 891 368.979 35.9742 267.000 511.000

Fixed effects

1 "CC" nlev

1 "gc" 100

2 "mn" 4

2 "RIGsf" nlev

1 "gc" 21

Random effects nlev

1 "animal" 5328 NRM

======== end of file ============================06-02-2015==========16:17====

======= Version 30-08-2013 ======================================= **KM** ====

Program WOMBAT: Estimates of covariance components

==============================================================================

Analysis type : "muv 2"

Data file : "DADOS.dat"

Pedigree file : "ReducedPedFile.dat"

Parameter file : "wombat.par"

No. of traits = 2 CC RIGsf

No. of records = 4767 3876 891

No. of parameters = 6

Maximum log L = -9636.813

-1/2 AIC & AICC = -9642.813 -9642.822

-1/2 BIC = -9662.138 "Penalty factor" = 4.221

Parameter estimates with approx. sampling erors

1 CHOL Z 1 1 5.04239 0.117891

2 CHOL Z 1 2 -0.459946E-01 0.859501E-01

3 CHOL Z 2 2 1.57835 0.597991E-01

4 CHOL A 1 1 1.18351 0.730371E-01

5 CHOL A 1 2 0.122623 0.133309

6 CHOL A 2 2 -0.540167 0.258289

Convergence criteria for last 3 iterates

Change in log likelihood = 0.203077 0.003597 0.000119

Change in parameter vector = 0.007666 0.001641 0.000323

Norm of gradient vector = 14.2752 1.1596 0.1649

Newton decrement = -0.3753 -0.0061 -0.0002

***** Estimates of residual covariances ************************************

Order of fit = 2

Covariance matrix

1 25.426

2 -0.23192 2.4933

Eigenvalues of covariance matrix

Value 25.43 2.49

(%) 91.08 8.92

Trace 27.92

Matrix of correlations and variance ratios

1 0.7045

2 -0.0291 0.8755

Covariances & correlations with approximate sampling errors

1 COVS Z 1 1 25.4257 1.18891 vrat 0.704 0.039

2 COVS Z 1 2 -0.231923 0.433230 corr -0.029 0.055

3 COVS Z 2 2 2.49330 0.188122 vrat 0.876 0.061

***** Estimates for RE 1 "animal" ***************************************

No. of levels = 5328

Covariance structure = NRM

Order of fit = 2

Covariance matrix

1 10.666

2 0.40046 0.35452

Eigenvalues of covariance matrix

Value 10.68 0.34

(%) 96.92 3.08

Trace 11.02

Matrix of correlations and variance ratios

1 0.2955

2 0.2059 0.1245

Covariances & correlations with approximate sampling errors

4 COVS A 1 1 10.6656 1.55797 vrat 0.296 0.039

5 COVS A 1 2 0.400465 0.436374 corr 0.206 0.220

6 COVS A 2 2 0.354518 0.178644 vrat 0.124 0.061

***** Estimates of phenotypic covariances ***********************************

Covariance matrix

1 36.091

2 0.16854 2.8478

Eigenvalues of covariance matrix

Value 36.09 2.85

(%) 92.69 7.31

Trace 38.94

Correlation matrix

1 1.0000

2 0.0166 1.0000

Covariances & correlations with approximate sampling errors

7 COVS T 1 1 36.0913 0.972030

8 COVS T 1 2 0.168542 0.372452 corr 0.017 0.037

9 COVS T 2 2 2.84782 0.140645

======== end of file ============================06-02-2015==========16:17====

======= Version 30-08-2013 ======================================= **KM** ====

Program WOMBAT: Summary of Pedigree Information

==============================================================================

Two traits analysis (LEA x RIGsf)

Analysis type : "muv 2"

Data file : "DADOS.dat"

Pedigree file : "ReducedPedFile.dat"

Parameter file : "wombat.par"

No. of animal IDs in data file = = 2350

No. of animal IDs in total = = 3729

*****Pedigree Structure for random effect : 1 ****************************

Original no. of animals = 3729

No. of animals after pruning = 3638

... proportion (%) remaining = 97.6

No. of levels w/out records = 1288

No. of levels with records = 2350 100.0%

... 1 record(s) = 1526 64.9%

... 2 record(s) = 824 35.1%

No. of animals w/out offspring = 1889 51.9%

No. of animals with offspring = 1749 48.1%

... and records = 461 12.7%

No. of animals with unknown sire = 64

No. of animals with unknown dam = 161

No. of animals with both parents unknown = 64

No. of animals with records =

... and unknown sire = 0

... and unknown dam = 0

... and both parents unknown = 0

No. of sires = 313

... with progeny in the data = 180

... with records & progeny in data = 84

No. of dams = 1436

... with progeny in the data = 1036

... with records & progeny in data = 371

No. of animals with known/unpruned grand-parents

... with paternal grandsire = 3493

... with paternal granddam = 3355

... with maternal grandsire = 3364

... with maternal granddam = 3240

Inbreeding coefficients for random effect 1 computed

No. of inbred animals = 3107

Average inbreeding coefficient = 2.3747 (in %)

... amongst inbred animals = 2.7805 (in %)

random effect no. = 1 NRM

no. of elements in NRM/GIN inverse 13937

log determinant = -2503.1878537727198

======== end of file ============================06-02-2015==========16:27====

======= Version 30-08-2013 ======================================= **KM** ====

Program WOMBAT: Summary of information from Set-up step

==============================================================================

Analysis type : "muv 2"

Data file : "DADOS.dat"

Pedigree file : "ReducedPedFile.dat"

Parameter file : "wombat.par"

No. of traits = 2

nrec mean sdev min. max.

1 "LEA" 2283 51.4305 8.89390 21.4000 83.4000

2 "RIGsf" 891 0.436001E-01 1.69595 -5.94410 4.80400

Numbers of individuals/records for pairs of traits

1 2

1 "LEA" 2283 824

2 "RIGsf" 824 891

Covariables

1"LEA" nrec mean sdev min. max.

1 "idv(2)" 2283 6.11082 2.92852 2.00000 17.0000

2 "idade(1)" 2283 448.951 98.8881 306.000 609.000

2"RIGsf" nrec mean sdev min. max.

1 "idv(2)" 891 6.16611 3.04967 3.00000 17.0000

2 "idade(1)" 891 368.979 35.9742 267.000 511.000

Fixed effects

1 "LEA" nlev

1 "gc" 65

2 "mn" 4

2 "RIGsf" nlev

1 "gc" 21

Random effects nlev

1 "animal" 3638 NRM

======== end of file ============================06-02-2015==========16:27====

======= Version 30-08-2013 ======================================= **KM** ====

Program WOMBAT: Estimates of covariance components

==============================================================================

Analysis type : "muv 2"

Data file : "DADOS.dat"

Pedigree file : "ReducedPedFile.dat"

Parameter file : "wombat.par"

No. of traits = 2 LEA RIGsf

No. of records = 3174 2283 891

No. of parameters = 6

Maximum log L = -6008.713

-1/2 AIC & AICC = -6014.713 -6014.726

-1/2 BIC = -6032.810 "Penalty factor" = 4.016

Parameter estimates with approx. sampling erors

1 CHOL Z 1 1 4.45232 0.174783

2 CHOL Z 1 2 -0.149796 0.102324

3 CHOL Z 2 2 1.58596 0.589395E-01

4 CHOL A 1 1 1.41200 0.680887E-01

5 CHOL A 1 2 0.160384 0.127195

6 CHOL A 2 2 -0.643167 0.307111

Convergence criteria for last 3 iterates

Change in log likelihood = 0.247996 0.001957 0.000027

Change in parameter vector = 0.017313 0.002906 0.000301

Norm of gradient vector = 12.9449 0.4902 0.0441

Newton decrement = -0.4745 -0.0036 -0.0000

***** Estimates of residual covariances ************************************

Order of fit = 2

Covariance matrix

1 19.823

2 -0.66694 2.5377

Eigenvalues of covariance matrix

Value 19.85 2.51

(%) 88.77 11.23

Trace 22.36

Matrix of correlations and variance ratios

1 0.5406

2 -0.0940 0.8936

Covariances & correlations with approximate sampling errors

1 COVS Z 1 1 19.8231 1.55637 vrat 0.541 0.051

2 COVS Z 1 2 -0.666939 0.454779 corr -0.094 0.064

3 COVS Z 2 2 2.53771 0.185285 vrat 0.894 0.058

***** Estimates for RE 1 "animal" ***************************************

No. of levels = 3638

Covariance structure = NRM

Order of fit = 2

Covariance matrix

1 16.844

2 0.65824 0.30200

Eigenvalues of covariance matrix

Value 16.87 0.28

(%) 98.39 1.61

Trace 17.15

Matrix of correlations and variance ratios

1 0.4594

2 0.2918 0.1064

Covariances & correlations with approximate sampling errors

4 COVS A 1 1 16.8441 2.29378 vrat 0.459 0.051

5 COVS A 1 2 0.658240 0.523391 corr 0.292 0.239

6 COVS A 2 2 0.302005 0.168192 vrat 0.106 0.058

***** Estimates of phenotypic covariances ***********************************

Covariance matrix

1 36.667

2 -0.86990E-02 2.8397

Eigenvalues of covariance matrix

Value 36.67 2.84

(%) 92.81 7.19

Trace 39.51

Correlation matrix

1 1.0000

2 -0.0009 1.0000

Covariances & correlations with approximate sampling errors

7 COVS T 1 1 36.6672 1.36189

8 COVS T 1 2 -0.869904E-02 0.386902 corr -0.001 0.038

9 COVS T 2 2 2.83971 0.139270

======== end of file ============================06-02-2015==========16:27====

======= Version 30-08-2013 ======================================= **KM** ====

Program WOMBAT: Summary of Pedigree Information

==============================================================================

Two traits analysis (BF x RIGsf)

Analysis type : "muv 2"

Data file : "DADOS.dat"

Pedigree file : "ReducedPedFile.dat"

Parameter file : "wombat.par"

No. of animal IDs in data file = = 2350

No. of animal IDs in total = = 3729

*****Pedigree Structure for random effect : 1 ****************************

Original no. of animals = 3729

No. of animals after pruning = 3638

... proportion (%) remaining = 97.6

No. of levels w/out records = 1288

No. of levels with records = 2350 100.0%

... 1 record(s) = 1524 64.9%

... 2 record(s) = 826 35.1%

No. of animals w/out offspring = 1889 51.9%

No. of animals with offspring = 1749 48.1%

... and records = 461 12.7%

No. of animals with unknown sire = 64

No. of animals with unknown dam = 161

No. of animals with both parents unknown = 64

No. of animals with records =

... and unknown sire = 0

... and unknown dam = 0

... and both parents unknown = 0

No. of sires = 313

... with progeny in the data = 180

... with records & progeny in data = 84

No. of dams = 1436

... with progeny in the data = 1036

... with records & progeny in data = 371

No. of animals with known/unpruned grand-parents

... with paternal grandsire = 3493

... with paternal granddam = 3355

... with maternal grandsire = 3364

... with maternal granddam = 3240

Inbreeding coefficients for random effect 1 computed

No. of inbred animals = 3107

Average inbreeding coefficient = 2.3747 (in %)

... amongst inbred animals = 2.7805 (in %)

random effect no. = 1 NRM

no. of elements in NRM/GIN inverse 13937

log determinant = -2503.1878537727198

======== end of file ============================06-02-2015==========16:33====

======= Version 30-08-2013 ======================================= **KM** ====

Program WOMBAT: Summary of information from Set-up step

==============================================================================

Analysis type : "muv 2"

Data file : "DADOS.dat"

Pedigree file : "ReducedPedFile.dat"

Parameter file : "wombat.par"

No. of traits = 2

nrec mean sdev min. max.

1 "BF" 2285 1.75922 1.42798 0.00000 10.5000

2 "RIGsf" 891 0.436001E-01 1.69595 -5.94410 4.80400

Numbers of individuals/records for pairs of traits

1 2

1 "BF" 2285 826

2 "RIGsf" 826 891

Covariables

1"BF" nrec mean sdev min. max.

1 "idv(2)" 2285 6.10810 2.92868 2.00000 17.0000

2 "idade(1)" 2285 449.081 98.9421 306.000 609.000

2"RIGsf" nrec mean sdev min. max.

1 "idv(2)" 891 6.16611 3.04967 3.00000 17.0000

2 "idade(1)" 891 368.979 35.9742 267.000 511.000

Fixed effects

1 "BF" nlev

1 "gc" 65

2 "mn" 4

2 "RIGsf" nlev

1 "gc" 21

Random effects nlev

1 "animal" 3638 NRM

======== end of file ============================06-02-2015==========16:33====

======= Version 30-08-2013 ======================================= **KM** ====

Program WOMBAT: Estimates of covariance components

==============================================================================

Analysis type : "muv 2"

Data file : "DADOS.dat"

Pedigree file : "ReducedPedFile.dat"

Parameter file : "wombat.par"

No. of traits = 2 BF RIGsf

No. of records = 3176 2285 891

No. of parameters = 6

Maximum log L = -1999.775

-1/2 AIC & AICC = -2005.775 -2005.788

-1/2 BIC = -2023.874 "Penalty factor" = 4.017

Parameter estimates with approx. sampling erors

1 CHOL Z 1 1 1.59967 0.580496E-01

2 CHOL Z 1 2 0.213940E-01 0.434955E-01

3 CHOL Z 2 2 0.800022 0.235422E-01

4 CHOL A 1 1 -0.650348 0.304397

5 CHOL A 1 2 -0.240227 0.138910

6 CHOL A 2 2 -0.750419 0.172707

Convergence criteria for last 3 iterates

Change in log likelihood = 0.035002 0.000723 0.000006

Change in parameter vector = 0.019634 0.002767 0.000294

Norm of gradient vector = 5.5809 1.5724 0.0325

Newton decrement = -0.0750 -0.0015 -0.0000

***** Estimates of residual covariances ************************************

Order of fit = 2

Covariance matrix

1 0.64049

2 0.34223E-01 2.5589

Eigenvalues of covariance matrix

Value 2.56 0.64

(%) 80.00 20.00

Trace 3.20

Matrix of correlations and variance ratios

1 0.6953

2 0.0267 0.9038

Covariances & correlations with approximate sampling errors

1 COVS Z 1 1 0.640493 0.375506E-01 vrat 0.695 0.046

2 COVS Z 1 2 0.342234E-01 0.695287E-01 corr 0.027 0.054

3 COVS Z 2 2 2.55895 0.185721 vrat 0.904 0.057

***** Estimates for RE 1 "animal" ***************************************

No. of levels = 3638

Covariance structure = NRM

Order of fit = 2

Covariance matrix

1 0.28065

2 -0.12537 0.27234

Eigenvalues of covariance matrix

Value 0.40 0.15

(%) 72.68 27.32

Trace 0.55

Matrix of correlations and variance ratios

1 0.3047

2 -0.4535 0.0962

Covariances & correlations with approximate sampling errors

4 COVS A 1 1 0.280652 0.475546E-01 vrat 0.305 0.046

5 COVS A 1 2 -0.125366 0.674691E-01 corr -0.453 0.260

6 COVS A 2 2 0.272342 0.165800 vrat 0.096 0.057

***** Estimates of phenotypic covariances ***********************************

Covariance matrix

1 0.92115

2 -0.91142E-01 2.8313

Eigenvalues of covariance matrix

Value 2.84 0.92

(%) 75.57 24.43

Trace 3.75

Correlation matrix

1 1.0000

2 -0.0564 1.0000

Covariances & correlations with approximate sampling errors

7 COVS T 1 1 0.921145 0.312812E-01

8 COVS T 1 2 -0.911423E-01 0.578297E-01 corr -0.056 0.036

9 COVS T 2 2 2.83129 0.138386

======== end of file ============================06-02-2015==========16:33====

======= Version 30-08-2013 ======================================= **KM** ====

Program WOMBAT: Summary of Pedigree Information

==============================================================================

Two traits analysis (RF x RIGsf)

Analysis type : "muv 2"

Data file : "DADOS.dat"

Pedigree file : "ReducedPedFile.dat"

Parameter file : "wombat.par"

No. of animal IDs in data file = = 1882

No. of animal IDs in total = = 3143

*****Pedigree Structure for random effect : 1 ****************************

Original no. of animals = 3143

No. of animals after pruning = 3058

... proportion (%) remaining = 97.3

No. of levels w/out records = 1176

No. of levels with records = 1882 100.0%

... 1 record(s) = 1056 56.1%

... 2 record(s) = 826 43.9%

No. of animals w/out offspring = 1471 48.1%

No. of animals with offspring = 1587 51.9%

... and records = 411 13.4%

No. of animals with unknown sire = 60

No. of animals with unknown dam = 147

No. of animals with both parents unknown = 59

No. of animals with records =

... and unknown sire = 0

... and unknown dam = 0

... and both parents unknown = 0

No. of sires = 307

... with progeny in the data = 114

... with records & progeny in data = 43

No. of dams = 1280

... with progeny in the data = 782

... with records & progeny in data = 368

No. of animals with known/unpruned grand-parents

... with paternal grandsire = 2934

... with paternal granddam = 2808

... with maternal grandsire = 2809

... with maternal granddam = 2720

Inbreeding coefficients for random effect 1 computed

No. of inbred animals = 2612

Average inbreeding coefficient = 2.5603 (in %)

... amongst inbred animals = 2.9975 (in %)

random effect no. = 1 NRM

no. of elements in NRM/GIN inverse 11684

log determinant = -2102.1598822553833

======== end of file ============================06-02-2015==========16:36====

======= Version 30-08-2013 ======================================= **KM** ====

Program WOMBAT: Summary of information from Set-up step

==============================================================================

Analysis type : "muv 2"

Data file : "DADOS.dat"

Pedigree file : "ReducedPedFile.dat"

Parameter file : "wombat.par"

No. of traits = 2

nrec mean sdev min. max.

1 "RF" 1817 5.08663 2.54341 0.00000 19.2000

2 "RIGsf" 891 0.436001E-01 1.69595 -5.94410 4.80400

Numbers of individuals/records for pairs of traits

1 2

1 "RF" 1817 826

2 "RIGsf" 826 891

Covariables

1"RF" nrec mean sdev min. max.

1 "idv(2)" 1817 6.09191 3.03138 2.00000 17.0000

2 "idade(1)" 1817 469.100 100.703 306.000 609.000

2"RIGsf" nrec mean sdev min. max.

1 "idv(2)" 891 6.16611 3.04967 3.00000 17.0000

2 "idade(1)" 891 368.979 35.9742 267.000 511.000

Fixed effects

1 "RF" nlev

1 "gc" 51

2 "mn" 3

2 "RIGsf" nlev

1 "gc" 21

Random effects nlev

1 "animal" 3058 NRM

======== end of file ============================06-02-2015==========16:36====

======= Version 30-08-2013 ======================================= **KM** ====

Program WOMBAT: Estimates of covariance components

==============================================================================

Analysis type : "muv 2"

Data file : "DADOS.dat"

Pedigree file : "ReducedPedFile.dat"

Parameter file : "wombat.par"

No. of traits = 2 RF RIGsf

No. of records = 2708 1817 891

No. of parameters = 6

Maximum log L = -2672.458

-1/2 AIC & AICC = -2678.458 -2678.474

-1/2 BIC = -2696.080 "Penalty factor" = 3.937

Parameter estimates with approx. sampling erors

1 CHOL Z 1 1 1.60153 0.584588E-01

2 CHOL Z 1 2 0.931036E-01 0.739994E-01

3 CHOL Z 2 2 1.26232 0.468373E-01

4 CHOL A 1 1 0.472894E-03 0.805052E-01

5 CHOL A 1 2 -0.224522 0.121445

6 CHOL A 2 2 -0.767084 0.393612

Convergence criteria for last 3 iterates

Change in log likelihood = 9.695868 0.063904 0.000158

Change in parameter vector = 0.078965 0.018338 0.002759

Norm of gradient vector = 178.6251 12.5947 0.1189

Newton decrement = -18.6155 -0.1263 -0.0003

***** Estimates of residual covariances ************************************

Order of fit = 2

Covariance matrix

1 1.6021

2 0.14911 2.5649

Eigenvalues of covariance matrix

Value 2.59 1.58

(%) 62.09 37.91

Trace 4.17

Matrix of correlations and variance ratios

1 0.6155

2 0.0736 0.9060

Covariances & correlations with approximate sampling errors

1 COVS Z 1 1 1.60212 0.116942 vrat 0.615 0.053

2 COVS Z 1 2 0.149108 0.118371 corr 0.074 0.059

3 COVS Z 2 2 2.56489 0.187247 vrat 0.906 0.058

***** Estimates for RE 1 "animal" ***************************************

No. of levels = 3058

Covariance structure = NRM

Order of fit = 2

Covariance matrix

1 1.0009

2 -0.22463 0.26605

Eigenvalues of covariance matrix

Value 1.06 0.20

(%) 83.99 16.01

Trace 1.27

Matrix of correlations and variance ratios

1 0.3845

2 -0.4353 0.0940

Covariances & correlations with approximate sampling errors

4 COVS A 1 1 1.00095 0.161163 vrat 0.385 0.053

5 COVS A 1 2 -0.224629 0.122941 corr -0.435 0.258

6 COVS A 2 2 0.266045 0.167547 vrat 0.094 0.058

***** Estimates of phenotypic covariances ***********************************

Covariance matrix

1 2.6031

2 -0.75521E-01 2.8309

Eigenvalues of covariance matrix

Value 2.85 2.58

(%) 52.52 47.48

Trace 5.43

Correlation matrix

1 1.0000

2 -0.0278 1.0000

Covariances & correlations with approximate sampling errors

7 COVS T 1 1 2.60307 0.102942

8 COVS T 1 2 -0.755206E-01 0.992288E-01 corr -0.028 0.036

9 COVS T 2 2 2.83094 0.138382

======== end of file ============================06-02-2015==========16:36====
