# Supplementary material for: Phenotypic and Genetic Correlations of Feed Efficiency Traits with Growth and Carcass Traits in Nellore Cattle Selected for Postweaning Weight
Source: PLoS One. 2016 Aug 18;11(8):e0161366. doi: 10.1371/journal.pone.0161366 (PMC4990259; doi:10.1371/journal.pone.0161366)
Supplement: S2 File — (DOCX) [file pone.0161366.s002.docx]

**S2 File. Pedigree information, model of analysis, parameter estimates, and the resulting covariance matrices for Table 3.**

======= Version 30-08-2013 ======================================= **KM** ====

Program WOMBAT: Summary of Pedigree Information

==============================================================================

Two traits analysis (ADG x DMI)

Analysis type : "muv 2"

Data file : "DADOS.dat"

Pedigree file : "ReducedPedFile.dat"

Parameter file : "wombat.par"

No. of animal IDs in data file = = 955

No. of animal IDs in total = = 2288

*****Pedigree Structure for random effect : 1 ****************************

Original no. of animals = 2288

No. of animals after pruning = 2204

... proportion (%) remaining = 96.3

No. of levels w/out records = 1249

No. of levels with records = 955 100.0%

... 2 record(s) = 955 100.0%

No. of animals w/out offspring = 828 37.6%

No. of animals with offspring = 1376 62.4%

... and records = 127 5.8%

No. of animals with unknown sire = 59

No. of animals with unknown dam = 145

No. of animals with both parents unknown = 58

No. of animals with records =

... and unknown sire = 0

... and unknown dam = 0

... and both parents unknown = 0

No. of sires = 296

... with progeny in the data = 78

... with records & progeny in data = 18

No. of dams = 1080

... with progeny in the data = 542

... with records & progeny in data = 98

No. of animals with known/unpruned grand-parents

... with paternal grandsire = 2083

... with paternal granddam = 1957

... with maternal grandsire = 1959

... with maternal granddam = 1872

Inbreeding coefficients for random effect 1 computed

No. of inbred animals = 1768

Average inbreeding coefficient = 2.1450 (in %)

... amongst inbred animals = 2.6740 (in %)

random effect no. = 1 NRM

no. of elements in NRM/GIN inverse 8375

log determinant = -1487.8684515369519

======== end of file ============================10-02-2015==========13:02====

======= Version 30-08-2013 ======================================= **KM** ====

Program WOMBAT: Summary of information from Set-up step

==============================================================================

Analysis type : "muv 2"

Data file : "DADOS.dat"

Pedigree file : "ReducedPedFile.dat"

Parameter file : "wombat.par"

No. of traits = 2

nrec mean sdev min. max.

1 "ADG" 955 1.00846 0.260202 0.176000 1.71800

2 "DMI" 955 6.83104 1.33500 2.15500 12.6400

Numbers of individuals/records for pairs of traits

1 2

1 "ADG" 955 955

2 "DMI" 955 955

Covariables

1"ADG" nrec mean sdev min. max.

1 "idv(2)" 955 6.15079 3.05304 3.00000 17.0000

2 "idade(1)" 955 369.980 37.3543 267.000 511.000

2"DMI" nrec mean sdev min. max.

1 "idv(2)" 955 6.15079 3.05304 3.00000 17.0000

2 "idade(1)" 955 369.980 37.3543 267.000 511.000

Fixed effects

1 "ADG" nlev

1 "gc" 21

2 "mn" 4

2 "DMI" nlev

1 "gc" 21

Random effects nlev

1 "animal" 2204 NRM

======== end of file ============================10-02-2015==========13:02====

======= Version 30-08-2013 ======================================= **KM** ====

Program WOMBAT: Estimates of covariance components

==============================================================================

Analysis type : "muv 2"

Data file : "DADOS.dat"

Pedigree file : "ReducedPedFile.dat"

Parameter file : "wombat.par"

No. of traits = 2 ADG DMI

No. of records = 1910 955 955

No. of parameters = 6

Maximum log L = 1043.842

-1/2 AIC & AICC = 1037.842 1037.820

-1/2 BIC = 1021.259 "Penalty factor" = 3.764

Parameter estimates with approx. sampling erors

1 CHOL Z 1 1 -0.435039 0.564281E-01

2 CHOL Z 1 2 0.431876E-01 0.901521E-02

3 CHOL Z 2 2 -2.18868 0.424649E-01

4 CHOL A 1 1 -0.510034 0.888563E-01

5 CHOL A 1 2 0.840201E-01 0.108622E-01

6 CHOL A 2 2 -2.89649 0.184939

Convergence criteria for last 3 iterates

Change in log likelihood = 0.009097 0.000703 0.000058

Change in parameter vector = 0.003574 0.000815 0.000218

Norm of gradient vector = 14.2530 3.7247 1.1220

Newton decrement = -0.0247 -0.0020 -0.0002

***** Estimates of residual covariances ************************************

Order of fit = 2

Covariance matrix

1 0.14424E-01

2 0.27953E-01 0.41892

Eigenvalues of covariance matrix

Value 0.42 0.01

(%) 97.12 2.88

Trace 0.43

Matrix of correlations and variance ratios

1 0.5880

2 0.3596 0.5374

Covariances & correlations with approximate sampling errors

1 COVS Z 1 1 0.144236E-01 0.147303E-02 vrat 0.588 0.066

2 COVS Z 1 2 0.279527E-01 0.681787E-02 corr 0.360 0.063

3 COVS Z 2 2 0.418919 0.472775E-01 vrat 0.537 0.068

***** Estimates for RE 1 "animal" ***************************************

No. of levels = 2204

Covariance structure = NRM

Order of fit = 2

Covariance matrix

1 0.10108E-01

2 0.50452E-01 0.36057

Eigenvalues of covariance matrix

Value 0.37 0.00

(%) 99.19 0.81

Trace 0.37

Matrix of correlations and variance ratios

1 0.4120

2 0.8357 0.4626

Covariances & correlations with approximate sampling errors

4 COVS A 1 1 0.101082E-01 0.189947E-02 vrat 0.412 0.066

5 COVS A 1 2 0.504520E-01 0.945927E-02 corr 0.836 0.063

6 COVS A 2 2 0.360571 0.640780E-01 vrat 0.463 0.068

***** Estimates of phenotypic covariances ***********************************

Covariance matrix

1 0.24532E-01

2 0.78405E-01 0.77949

Eigenvalues of covariance matrix

Value 0.79 0.02

(%) 97.95 2.05

Trace 0.80

Correlation matrix

1 1.0000

2 0.5670 1.0000

Covariances & correlations with approximate sampling errors

7 COVS T 1 1 0.245319E-01 0.125596E-02

8 COVS T 1 2 0.784046E-01 0.595477E-02 corr 0.567 0.024

9 COVS T 2 2 0.779489 0.406894E-01

======== end of file ============================10-02-2015==========13:02====

======= Version 30-08-2013 ======================================= **KM** ====

Program WOMBAT: Summary of Pedigree Information

==============================================================================

Two traits analysis (BW0.75 x DMI)

Analysis type : "muv 2"

Data file : "DADOS.dat"

Pedigree file : "ReducedPedFile.dat"

Parameter file : "wombat.par"

No. of animal IDs in data file = = 955

No. of animal IDs in total = = 2288

*****Pedigree Structure for random effect : 1 ****************************

Original no. of animals = 2288

No. of animals after pruning = 2204

... proportion (%) remaining = 96.3

No. of levels w/out records = 1249

No. of levels with records = 955 100.0%

... 2 record(s) = 955 100.0%

No. of animals w/out offspring = 828 37.6%

No. of animals with offspring = 1376 62.4%

... and records = 127 5.8%

No. of animals with unknown sire = 59

No. of animals with unknown dam = 145

No. of animals with both parents unknown = 58

No. of animals with records =

... and unknown sire = 0

... and unknown dam = 0

... and both parents unknown = 0

No. of sires = 296

... with progeny in the data = 78

... with records & progeny in data = 18

No. of dams = 1080

... with progeny in the data = 542

... with records & progeny in data = 98

No. of animals with known/unpruned grand-parents

... with paternal grandsire = 2083

... with paternal granddam = 1957

... with maternal grandsire = 1959

... with maternal granddam = 1872

Inbreeding coefficients for random effect 1 computed

No. of inbred animals = 1768

Average inbreeding coefficient = 2.1450 (in %)

... amongst inbred animals = 2.6740 (in %)

random effect no. = 1 NRM

no. of elements in NRM/GIN inverse 8375

log determinant = -1487.8684515369519

======== end of file ============================10-02-2015==========13:03====

======= Version 30-08-2013 ======================================= **KM** ====

Program WOMBAT: Summary of information from Set-up step

==============================================================================

Analysis type : "muv 2"

Data file : "DADOS.dat"

Pedigree file : "ReducedPedFile.dat"

Parameter file : "wombat.par"

No. of traits = 2

nrec mean sdev min. max.

1 "BW0.75" 955 68.1510 10.1292 37.4020 94.5940

2 "DMI" 955 6.83104 1.33500 2.15500 12.6400

Numbers of individuals/records for pairs of traits

1 2

1 " BW0.75" 955 955

2 "DMI" 955 955

Covariables

1" BW0.75" nrec mean sdev min. max.

1 "idv(2)" 955 6.15079 3.05304 3.00000 17.0000

2 "idade(1)" 955 369.980 37.3543 267.000 511.000

2"DMI" nrec mean sdev min. max.

1 "idv(2)" 955 6.15079 3.05304 3.00000 17.0000

2 "idade(1)" 955 369.980 37.3543 267.000 511.000

Fixed effects

1 " BW0.75" nlev

1 "gc" 21

2 "mn" 4

2 "DMI" nlev

1 "gc" 21

Random effects nlev

1 "animal" 2204 NRM

======== end of file ============================10-02-2015==========13:03====

======= Version 30-08-2013 ======================================= **KM** ====

Program WOMBAT: Estimates of covariance components

==============================================================================

Analysis type : "muv 2"

Data file : "DADOS.dat"

Pedigree file : "ReducedPedFile.dat"

Parameter file : "wombat.par"

No. of traits = 2 BW0.75 DMI

No. of records = 1910 955 955

No. of parameters = 6

Maximum log L = -2172.344

-1/2 AIC & AICC = -2178.344 -2178.366

-1/2 BIC = -2194.928 "Penalty factor" = 3.764

Parameter estimates with approx. sampling erors

1 CHOL Z 1 1 3.63701 0.288538

2 CHOL Z 1 2 0.298597 0.576658E-01

3 CHOL Z 2 2 0.557732 0.259291E-01

4 CHOL A 1 1 1.44896 0.859508E-01

5 CHOL A 1 2 0.510993 0.636012E-01

6 CHOL A 2 2 -1.02793 0.136701

Convergence criteria for last 3 iterates

Change in log likelihood = 1.517178 0.001936 0.000080

Change in parameter vector = 0.075079 0.002879 0.000401

Norm of gradient vector = 57.9220 1.6639 0.2319

Newton decrement = -3.1026 -0.0048 -0.0002

***** Estimates of residual covariances ************************************

Order of fit = 2

Covariance matrix

1 13.228

2 1.0860 0.40023

Eigenvalues of covariance matrix

Value 13.32 0.31

(%) 97.73 2.27

Trace 13.63

Matrix of correlations and variance ratios

1 0.4218

2 0.4720 0.5070

Covariances & correlations with approximate sampling errors

1 COVS Z 1 1 13.2279 2.09883 vrat 0.422 0.077

2 COVS Z 1 2 1.08600 0.267212 corr 0.472 0.071

3 COVS Z 2 2 0.400226 0.479450E-01 vrat 0.507 0.069

***** Estimates for RE 1 "animal" ***************************************

No. of levels = 2204

Covariance structure = NRM

Order of fit = 2

Covariance matrix

1 18.136

2 2.1762 0.38910

Eigenvalues of covariance matrix

Value 18.40 0.13

(%) 99.32 0.68

Trace 18.53

Matrix of correlations and variance ratios

1 0.5782

2 0.8192 0.4930

Covariances & correlations with approximate sampling errors

4 COVS A 1 1 18.1363 3.11766 vrat 0.578 0.077

5 COVS A 1 2 2.17615 0.397832 corr 0.819 0.055

6 COVS A 2 2 0.389097 0.671439E-01 vrat 0.493 0.069

***** Estimates of phenotypic covariances ***********************************

Covariance matrix

1 31.364

2 3.2622 0.78932

Eigenvalues of covariance matrix

Value 31.71 0.45

(%) 98.62 1.38

Trace 32.15

Correlation matrix

1 1.0000

2 0.6556 1.0000

Covariances & correlations with approximate sampling errors

7 COVS T 1 1 31.3642 1.76481

8 COVS T 1 2 3.26216 0.233545 corr 0.656 0.022

9 COVS T 2 2 0.789323 0.419268E-01

======== end of file ============================10-02-2015==========13:03====

======= Version 30-08-2013 ======================================= **KM** ====

Program WOMBAT: Summary of Pedigree Information

==============================================================================

Two traits analysis (WS x DMI)

Analysis type : "muv 2"

Data file : "DADOS.dat"

Pedigree file : "ReducedPedFile.dat"

Parameter file : "wombat.par"

No. of animal IDs in data file = = 8091

No. of animal IDs in total = = 8490

*****Pedigree Structure for random effect : 1 ****************************

Original no. of animals = 8490

No. of animals after pruning = 8433

... proportion (%) remaining = 99.3

No. of levels w/out records = 342

No. of levels with records = 8091 100.0%

... 1 record(s) = 7149 88.4%

... 2 record(s) = 942 11.6%

No. of animals w/out offspring = 6052 71.8%

No. of animals with offspring = 2381 28.2%

... and records = 2039 24.2%

No. of animals with unknown sire = 313

No. of animals with unknown dam = 384

No. of animals with both parents unknown = 312

No. of animals with records =

... and unknown sire = 1

... and unknown dam = 53

... and both parents unknown = 0

No. of sires = 325

... with progeny in the data = 320

... with records & progeny in data = 288

No. of dams = 2056

... with progeny in the data = 2056

... with records & progeny in data = 1751

No. of animals with known/unpruned grand-parents

... with paternal grandsire = 7358

... with paternal granddam = 7056

... with maternal grandsire = 6535

... with maternal granddam = 6468

random effect no. = 1 NRM

no. of elements in NRM/GIN inverse 31838

log determinant = -5688.7609053187680

random effect no. = 2 IDE

no. of elements in NRM/GIN inverse 0

log determinant = 0.0000000000000000

======== end of file ============================10-02-2015==========12:02====

======= Version 30-08-2013 ======================================= **KM** ====

Program WOMBAT: Summary of information from Set-up step

==============================================================================

Analysis type : "muv 2"

Data file : "DADOS.dat"

Pedigree file : "ReducedPedFile.dat"

Parameter file : "wombat.par"

No. of traits = 2

nrec mean sdev min. max.

1 "WS" 8078 299.703 49.6462 160.030 489.760

2 "DMI" 955 6.83104 1.33500 2.15500 12.6400

Numbers of individuals/records for pairs of traits

1 2

1 "WS" 8078 942

2 "DMI" 942 955

Covariables

1"WS" nrec mean sdev min. max.

1 "idv(2)" 8078 6.63568 3.07616 2.00000 18.0000

2 "idade(1)" 8078 473.414 105.028 293.000 645.000

2"DMI" nrec mean sdev min. max.

1 "idv(2)" 955 6.15079 3.05304 3.00000 17.0000

2 "idade(1)" 955 369.980 37.3543 267.000 511.000

Fixed effects

1 "WS" nlev

1 "gc" 201

2 "mn" 4

2 "DMI" nlev

1 "gc" 21

Random effects nlev

1 "animal" 8433 NRM

2 "peanim" 2107 IDE

======== end of file ============================10-02-2015==========12:02====

======= Version 30-08-2013 ======================================= **KM** ====

Program WOMBAT: Estimates of covariance components

==============================================================================

Analysis type : "muv 2"

Data file : "DADOS.dat"

Pedigree file : "ReducedPedFile.dat"

Parameter file : "wombat.par"

No. of traits = 2 WS DMI

No. of records = 9033 8078 955

No. of parameters = 7

Maximum log L = -30284.805

-1/2 AIC & AICC = -30291.805 -30291.811

-1/2 BIC = -30316.595 "Penalty factor" = 4.541

Parameter estimates with approx. sampling erors

1 CHOL Z 1 1 20.4088 0.438990

2 CHOL Z 1 2 0.334913 0.340432E-01

3 CHOL Z 2 2 0.559519 0.250676E-01

4 CHOL A 1 1 2.92945 0.439925E-01

5 CHOL A 1 2 0.422699 0.452764E-01

6 CHOL A 2 2 -1.18124 0.174165

7 CHOL B 1 1 2.24940 0.538989E-01

Convergence criteria for last 3 iterates

Change in log likelihood = 0.025045 0.001356 0.000076

Change in parameter vector = 0.001288 0.000620 0.000113

Norm of gradient vector = 7.7247 1.3881 0.3038

Newton decrement = -0.0558 -0.0029 -0.0002

***** Estimates of residual covariances ************************************

Order of fit = 2

Covariance matrix

1 416.52

2 6.8352 0.42523

Eigenvalues of covariance matrix

Value 416.63 0.31

(%) 99.92 0.08

Trace 416.94

Matrix of correlations and variance ratios

1 0.4862

2 0.5136 0.6091

Covariances & correlations with approximate sampling errors

1 COVS Z 1 1 416.520 17.9185 vrat 0.486 0.027

2 COVS Z 1 2 6.83518 0.738396 corr 0.514 0.042

3 COVS Z 2 2 0.425229 0.363701E-01 vrat 0.609 0.055

***** Estimates for RE 1 "animal" ***************************************

No. of levels = 8433

Covariance structure = NRM

Order of fit = 2

Covariance matrix

1 350.34

2 7.9118 0.27286

Eigenvalues of covariance matrix

Value 350.52 0.09

(%) 99.97 0.03

Trace 350.61

Matrix of correlations and variance ratios

1 0.4089

2 0.8092 0.3909

Covariances & correlations with approximate sampling errors

4 COVS A 1 1 350.340 30.8247 vrat 0.409 0.030

5 COVS A 1 2 7.91182 0.975184 corr 0.809 0.062

6 COVS A 2 2 0.272861 0.446160E-01 vrat 0.391 0.055

***** Estimates for RE 2 "peanim" ***************************************

No. of levels = 2107

Covariance structure = IDE

Order of fit = 1

Covariance matrix

1 89.910

Matrix of correlations and variance ratios

1 0.1049

Covariances & correlations with approximate sampling errors

7 COVS B 1 1 89.9097 9.69207 vrat 0.105 0.011

***** Estimates of phenotypic covariances ***********************************

Covariance matrix

1 856.77

2 14.747 0.69809

Eigenvalues of covariance matrix

Value 857.02 0.44

(%) 99.95 0.05

Trace 857.47

Correlation matrix

1 1.0000

2 0.6030 1.0000

Covariances & correlations with approximate sampling errors

8 COVS T 1 1 856.770 18.8198

9 COVS T 1 2 14.7470 0.712416 corr 0.603 0.019

10 COVS T 2 2 0.698090 0.318593E-01

======== end of file ============================10-02-2015==========12:02====

======= Version 30-08-2013 ======================================= **KM** ====

Program WOMBAT: Summary of Pedigree Information

==============================================================================

Two traits analysis (HH x DMI)

Analysis type : "muv 2"

Data file : "DADOS.dat"

Pedigree file : "ReducedPedFile.dat"

Parameter file : "wombat.par"

No. of animal IDs in data file = = 6560

No. of animal IDs in total = = 7100

*****Pedigree Structure for random effect : 1 ****************************

Original no. of animals = 7100

No. of animals after pruning = 7017

... proportion (%) remaining = 98.8

No. of levels w/out records = 457

No. of levels with records = 6560 100.0%

... 1 record(s) = 5617 85.6%

... 2 record(s) = 943 14.4%

No. of animals w/out offspring = 4829 68.8%

No. of animals with offspring = 2188 31.2%

... and records = 1731 24.7%

No. of animals with unknown sire = 148

No. of animals with unknown dam = 237

No. of animals with both parents unknown = 148

No. of animals with records =

... and unknown sire = 0

... and unknown dam = 18

... and both parents unknown = 0

No. of sires = 320

... with progeny in the data = 283

... with records & progeny in data = 240

No. of dams = 1868

... with progeny in the data = 1837

... with records & progeny in data = 1491

No. of animals with known/unpruned grand-parents

... with paternal grandsire = 6738

... with paternal granddam = 6484

... with maternal grandsire = 6339

... with maternal granddam = 6111

random effect no. = 1 NRM

no. of elements in NRM/GIN inverse 26822

log determinant = -4814.8039049931958

======== end of file ============================10-02-2015==========12:09====

======= Version 30-08-2013 ======================================= **KM** ====

Program WOMBAT: Summary of information from Set-up step

==============================================================================

Analysis type : "muv 2"

Data file : "DADOS.dat"

Pedigree file : "ReducedPedFile.dat"

Parameter file : "wombat.par"

No. of traits = 2

nrec mean sdev min. max.

1 "HH" 6548 132.269 5.43028 100.000 149.000

2 "DMI" 955 6.83104 1.33500 2.15500 12.6400

Numbers of individuals/records for pairs of traits

1 2

1 "HH" 6548 943

2 "DMI" 943 955

Covariables

1"HH" nrec mean sdev min. max.

1 "idv(2)" 6548 6.40043 2.93455 2.00000 17.0000

2 "idade(1)" 6548 472.701 107.333 293.000 645.000

2"DMI" nrec mean sdev min. max.

1 "idv(2)" 955 6.15079 3.05304 3.00000 17.0000

2 "idade(1)" 955 369.980 37.3543 267.000 511.000

Fixed effects

1 "HH" nlev

1 "gc" 169

2 "mn" 4

2 "DMI" nlev

1 "gc" 21

Random effects nlev

1 "animal" 7017 NRM

======== end of file ============================10-02-2015==========12:09====

======= Version 30-08-2013 ======================================= **KM** ====

Program WOMBAT: Estimates of covariance components

==============================================================================

Analysis type : "muv 2"

Data file : "DADOS.dat"

Pedigree file : "ReducedPedFile.dat"

Parameter file : "wombat.par"

No. of traits = 2 HH DMI

No. of records = 7503 6548 955

No. of parameters = 6

Maximum log L = -11672.635

-1/2 AIC & AICC = -11678.635 -11678.641

-1/2 BIC = -11699.324 "Penalty factor" = 4.448

Parameter estimates with approx. sampling erors

1 CHOL Z 1 1 2.43209 0.656328E-01

2 CHOL Z 1 2 0.196502 0.487622E-01

3 CHOL Z 2 2 0.628366 0.312813E-01

4 CHOL A 1 1 1.13086 0.311172E-01

5 CHOL A 1 2 0.360247 0.535191E-01

6 CHOL A 2 2 -0.770008 0.115606

Convergence criteria for last 3 iterates

Change in log likelihood = 0.135118 0.002001 0.000046

Change in parameter vector = 0.012029 0.001521 0.000269

Norm of gradient vector = 14.5438 1.3156 0.1837

Newton decrement = -0.2521 -0.0037 -0.0001

***** Estimates of residual covariances ************************************

Order of fit = 2

Covariance matrix

1 5.9151

2 0.47791 0.43346

Eigenvalues of covariance matrix

Value 5.96 0.39

(%) 93.82 6.18

Trace 6.35

Matrix of correlations and variance ratios

1 0.3813

2 0.2985 0.5574

Covariances & correlations with approximate sampling errors

1 COVS Z 1 1 5.91506 0.319250 vrat 0.381 0.026

2 COVS Z 1 2 0.477911 0.121310 corr 0.298 0.068

3 COVS Z 2 2 0.433457 0.447925E-01 vrat 0.557 0.064

***** Estimates for RE 1 "animal" ***************************************

No. of levels = 7017

Covariance structure = NRM

Order of fit = 2

Covariance matrix

1 9.5995

2 1.1162 0.34416

Eigenvalues of covariance matrix

Value 9.73 0.21

(%) 97.87 2.13

Trace 9.94

Matrix of correlations and variance ratios

1 0.6187

2 0.6141 0.4426

Covariances & correlations with approximate sampling errors

4 COVS A 1 1 9.59954 0.597422 vrat 0.619 0.026

5 COVS A 1 2 1.11616 0.175239 corr 0.614 0.076

6 COVS A 2 2 0.344156 0.589192E-01 vrat 0.443 0.064

***** Estimates of phenotypic covariances ***********************************

Covariance matrix

1 15.515

2 1.5941 0.77761

Eigenvalues of covariance matrix

Value 15.69 0.61

(%) 96.27 3.73

Trace 16.29

Correlation matrix

1 1.0000

2 0.4589 1.0000

Covariances & correlations with approximate sampling errors

7 COVS T 1 1 15.5146 0.386125

8 COVS T 1 2 1.59407 0.120983 corr 0.459 0.028

9 COVS T 2 2 0.777613 0.392417E-01

======== end of file ============================10-02-2015==========12:09====

======= Version 30-08-2013 ======================================= **KM** ====

Program WOMBAT: Summary of Pedigree Information

==============================================================================

Two traits analysis (CC x DMI)

Analysis type : "muv 2"

Data file : "DADOS.dat"

Pedigree file : "ReducedPedFile.dat"

Parameter file : "wombat.par"

No. of animal IDs in data file = = 3943

No. of animal IDs in total = = 5418

*****Pedigree Structure for random effect : 1 ****************************

Original no. of animals = 5418

No. of animals after pruning = 5330

... proportion (%) remaining = 98.4

No. of levels w/out records = 1387

No. of levels with records = 3943 100.0%

... 1 record(s) = 3055 77.5%

... 2 record(s) = 888 22.5%

No. of animals w/out offspring = 3311 62.1%

No. of animals with offspring = 2019 37.9%

... and records = 632 11.9%

No. of animals with unknown sire = 94

No. of animals with unknown dam = 188

No. of animals with both parents unknown = 94

No. of animals with records =

... and unknown sire = 0

... and unknown dam = 0

... and both parents unknown = 0

No. of sires = 318

... with progeny in the data = 255

... with records & progeny in data = 217

No. of dams = 1701

... with progeny in the data = 1508

... with records & progeny in data = 412

No. of animals with known/unpruned grand-parents

... with paternal grandsire = 5142

... with paternal granddam = 4954

... with maternal grandsire = 4953

... with maternal granddam = 4738

random effect no. = 1 NRM

no. of elements in NRM/GIN inverse 20464

log determinant = -3671.8274443710275

======== end of file ============================10-02-2015==========12:46====

======= Version 30-08-2013 ======================================= **KM** ====

Program WOMBAT: Summary of information from Set-up step

==============================================================================

Analysis type : "muv 2"

Data file : "DADOS.dat"

Pedigree file : "ReducedPedFile.dat"

Parameter file : "wombat.par"

No. of traits = 2

nrec mean sdev min. max.

1 "CC" 3876 164.140 8.72028 128.000 192.000

2 "DMI" 955 6.83104 1.33500 2.15500 12.6400

Numbers of individuals/records for pairs of traits

1 2

1 "CC" 3876 888

2 "DMI" 888 955

Covariables

1"CC" nrec mean sdev min. max.

1 "idv(2)" 3876 6.27632 2.87660 2.00000 17.0000

2 "idade(1)" 3876 431.403 109.187 293.000 725.000

2"DMI" nrec mean sdev min. max.

1 "idv(2)" 955 6.15079 3.05304 3.00000 17.0000

2 "idade(1)" 955 369.980 37.3543 267.000 511.000

Fixed effects

1 "CC" nlev

1 "gc" 100

2 "mn" 4

2 "DMI" nlev

1 "gc" 21

Random effects nlev

1 "animal" 5330 NRM

======== end of file ============================10-02-2015==========12:46====

======= Version 30-08-2013 ======================================= **KM** ====

Program WOMBAT: Estimates of covariance components

==============================================================================

Analysis type : "muv 2"

Data file : "DADOS.dat"

Pedigree file : "ReducedPedFile.dat"

Parameter file : "wombat.par"

No. of traits = 2 CC DMI

No. of records = 4831 3876 955

No. of parameters = 6

Maximum log L = -8910.760

-1/2 AIC & AICC = -8916.760 -8916.769

-1/2 BIC = -8936.127 "Penalty factor" = 4.228

Parameter estimates with approx. sampling erors

1 CHOL Z 1 1 4.97847 0.119101

2 CHOL Z 1 2 0.279539 0.376286E-01

3 CHOL Z 2 2 0.595725 0.289774E-01

4 CHOL A 1 1 1.22979 0.681143E-01

5 CHOL A 1 2 0.461268 0.596572E-01

6 CHOL A 2 2 -1.02318 0.167842

Convergence criteria for last 3 iterates

Change in log likelihood = 0.134892 0.002884 0.000094

Change in parameter vector = 0.006726 0.001495 0.000280

Norm of gradient vector = 12.2522 1.1789 0.1834

Newton decrement = -0.2443 -0.0049 -0.0002

***** Estimates of residual covariances ************************************

Order of fit = 2

Covariance matrix

1 24.785

2 1.3917 0.43303

Eigenvalues of covariance matrix

Value 24.86 0.35

(%) 98.60 1.40

Trace 25.22

Matrix of correlations and variance ratios

1 0.6793

2 0.4248 0.5587

Covariances & correlations with approximate sampling errors

1 COVS Z 1 1 24.7852 1.18588 vrat 0.679 0.038

2 COVS Z 1 2 1.39168 0.200830 corr 0.425 0.047

3 COVS Z 2 2 0.433031 0.430947E-01 vrat 0.559 0.061

***** Estimates for RE 1 "animal" ***************************************

No. of levels = 5330

Covariance structure = NRM

Order of fit = 2

Covariance matrix

1 11.700

2 1.5778 0.34197

Eigenvalues of covariance matrix

Value 11.92 0.13

(%) 98.95 1.05

Trace 12.04

Matrix of correlations and variance ratios

1 0.3207

2 0.7888 0.4413

Covariances & correlations with approximate sampling errors

4 COVS A 1 1 11.6999 1.59386 vrat 0.321 0.038

5 COVS A 1 2 1.57777 0.252836 corr 0.789 0.073

6 COVS A 2 2 0.341972 0.563263E-01 vrat 0.441 0.061

***** Estimates of phenotypic covariances ***********************************

Covariance matrix

1 36.485

2 2.9694 0.77500

Eigenvalues of covariance matrix

Value 36.73 0.53

(%) 98.58 1.42

Trace 37.26

Correlation matrix

1 1.0000

2 0.5584 1.0000

Covariances & correlations with approximate sampling errors

7 COVS T 1 1 36.4851 0.994190

8 COVS T 1 2 2.96945 0.179836 corr 0.558 0.023

9 COVS T 2 2 0.775003 0.382396E-01

======== end of file ============================10-02-2015==========12:46====

======= Version 30-08-2013 ======================================= **KM** ====

Program WOMBAT: Summary of Pedigree Information

==============================================================================

Two traits analysis (LEA x DMI)

Analysis type : "muv 2"

Data file : "DADOS.dat"

Pedigree file : "ReducedPedFile.dat"

Parameter file : "wombat.par"

No. of animal IDs in data file = = 2396

No. of animal IDs in total = = 3756

*****Pedigree Structure for random effect : 1 ****************************

Original no. of animals = 3756

No. of animals after pruning = 3665

... proportion (%) remaining = 97.6

No. of levels w/out records = 1269

No. of levels with records = 2396 100.0%

... 1 record(s) = 1554 64.9%

... 2 record(s) = 842 35.1%

No. of animals w/out offspring = 1913 52.2%

No. of animals with offspring = 1752 47.8%

... and records = 483 13.2%

No. of animals with unknown sire = 64

No. of animals with unknown dam = 161

No. of animals with both parents unknown = 64

No. of animals with records =

... and unknown sire = 0

... and unknown dam = 0

... and both parents unknown = 0

No. of sires = 313

... with progeny in the data = 180

... with records & progeny in data = 85

No. of dams = 1439

... with progeny in the data = 1042

... with records & progeny in data = 392

No. of animals with known/unpruned grand-parents

... with paternal grandsire = 3520

... with paternal granddam = 3382

... with maternal grandsire = 3391

... with maternal granddam = 3267

Inbreeding coefficients for random effect 1 computed

No. of inbred animals = 3134

Average inbreeding coefficient = 2.3793 (in %)

... amongst inbred animals = 2.7824 (in %)

random effect no. = 1 NRM

no. of elements in NRM/GIN inverse 14038

log determinant = -2522.5521618264124

======== end of file ============================10-02-2015==========12:49====

======= Version 30-08-2013 ======================================= **KM** ====

Program WOMBAT: Summary of information from Set-up step

==============================================================================

Analysis type : "muv 2"

Data file : "DADOS.dat"

Pedigree file : "ReducedPedFile.dat"

Parameter file : "wombat.par"

No. of traits = 2

nrec mean sdev min. max.

1 "LEA" 2283 51.4305 8.89390 21.4000 83.4000

2 "DMI" 955 6.83104 1.33500 2.15500 12.6400

Numbers of individuals/records for pairs of traits

1 2

1 "LEA" 2283 842

2 "DMI" 842 955

Covariables

1"LEA" nrec mean sdev min. max.

1 "idv(2)" 2283 6.11082 2.92852 2.00000 17.0000

2 "idade(1)" 2283 448.951 98.8881 306.000 609.000

2"DMI" nrec mean sdev min. max.

1 "idv(2)" 955 6.15079 3.05304 3.00000 17.0000

2 "idade(1)" 955 369.980 37.3543 267.000 511.000

Fixed effects

1 "LEA" nlev

1 "gc" 65

2 "mn" 4

2 "DMI" nlev

1 "gc" 21

Random effects nlev

1 "animal" 3665 NRM

======== end of file ============================10-02-2015==========12:49====

======= Version 30-08-2013 ======================================= **KM** ====

Program WOMBAT: Estimates of covariance components

==============================================================================

Analysis type : "muv 2"

Data file : "DADOS.dat"

Pedigree file : "ReducedPedFile.dat"

Parameter file : "wombat.par"

No. of traits = 2 LEA DMI

No. of records = 3238 2283 955

No. of parameters = 6

Maximum log L = -5359.010

-1/2 AIC & AICC = -5365.010 -5365.023

-1/2 BIC = -5383.168 "Penalty factor" = 4.026

Parameter estimates with approx. sampling erors

1 CHOL Z 1 1 4.41891 0.175518

2 CHOL Z 1 2 0.232155 0.488244E-01

3 CHOL Z 2 2 0.602986 0.336757E-01

4 CHOL A 1 1 1.42717 0.665879E-01

5 CHOL A 1 2 0.289813 0.697580E-01

6 CHOL A 2 2 -0.628927 0.978025E-01

Convergence criteria for last 3 iterates

Change in log likelihood = 0.052870 0.000506 0.000023

Change in parameter vector = 0.004681 0.000861 0.000156

Norm of gradient vector = 13.7109 0.4405 0.1332

Newton decrement = -0.1057 -0.0012 -0.0001

***** Estimates of residual covariances ************************************

Order of fit = 2

Covariance matrix

1 19.527

2 1.0259 0.41749

Eigenvalues of covariance matrix

Value 19.58 0.36

(%) 98.18 1.82

Trace 19.94

Matrix of correlations and variance ratios

1 0.5293

2 0.3593 0.5313

Covariances & correlations with approximate sampling errors

1 COVS Z 1 1 19.5268 1.55120 vrat 0.529 0.051

2 COVS Z 1 2 1.02587 0.228930 corr 0.359 0.068

3 COVS Z 2 2 0.417487 0.460325E-01 vrat 0.531 0.066

***** Estimates for RE 1 "animal" ***************************************

No. of levels = 3665

Covariance structure = NRM

Order of fit = 2

Covariance matrix

1 17.363

2 1.2076 0.36825

Eigenvalues of covariance matrix

Value 17.45 0.28

(%) 98.40 1.60

Trace 17.73

Matrix of correlations and variance ratios

1 0.4707

2 0.4776 0.4687

Covariances & correlations with approximate sampling errors

4 COVS A 1 1 17.3630 2.31233 vrat 0.471 0.051

5 COVS A 1 2 1.20762 0.319075 corr 0.478 0.101

6 COVS A 2 2 0.368255 0.627377E-01 vrat 0.469 0.066

***** Estimates of phenotypic covariances ***********************************

Covariance matrix

1 36.890

2 2.2335 0.78574

Eigenvalues of covariance matrix

Value 37.03 0.65

(%) 98.28 1.72

Trace 37.68

Correlation matrix

1 1.0000

2 0.4148 1.0000

Covariances & correlations with approximate sampling errors

7 COVS T 1 1 36.8898 1.37759

8 COVS T 1 2 2.23349 0.208649 corr 0.415 0.032

9 COVS T 2 2 0.785742 0.407205E-01

======== end of file ============================10-02-2015==========12:49====

======= Version 30-08-2013 ======================================= **KM** ====

Program WOMBAT: Summary of Pedigree Information

==============================================================================

Two traits analysis (BF x DMI)

Analysis type : "muv 2"

Data file : "DADOS.dat"

Pedigree file : "ReducedPedFile.dat"

Parameter file : "wombat.par"

No. of animal IDs in data file = = 2396

No. of animal IDs in total = = 3756

*****Pedigree Structure for random effect : 1 ****************************

Original no. of animals = 3756

No. of animals after pruning = 3665

... proportion (%) remaining = 97.6

No. of levels w/out records = 1269

No. of levels with records = 2396 100.0%

... 1 record(s) = 1552 64.8%

... 2 record(s) = 844 35.2%

No. of animals w/out offspring = 1913 52.2%

No. of animals with offspring = 1752 47.8%

... and records = 483 13.2%

No. of animals with unknown sire = 64

No. of animals with unknown dam = 161

No. of animals with both parents unknown = 64

No. of animals with records =

... and unknown sire = 0

... and unknown dam = 0

... and both parents unknown = 0

No. of sires = 313

... with progeny in the data = 180

... with records & progeny in data = 85

No. of dams = 1439

... with progeny in the data = 1042

... with records & progeny in data = 392

No. of animals with known/unpruned grand-parents

... with paternal grandsire = 3520

... with paternal granddam = 3382

... with maternal grandsire = 3391

... with maternal granddam = 3267

random effect no. = 1 NRM

no. of elements in NRM/GIN inverse 14038

log determinant = -2522.5521618264124

======== end of file ============================10-02-2015==========12:53====

======= Version 30-08-2013 ======================================= **KM** ====

Program WOMBAT: Summary of information from Set-up step

==============================================================================

Analysis type : "muv 2"

Data file : "DADOS.dat"

Pedigree file : "ReducedPedFile.dat"

Parameter file : "wombat.par"

No. of traits = 2

nrec mean sdev min. max.

1 "BF" 2285 1.75922 1.42798 0.00000 10.5000

2 "DMI" 955 6.83104 1.33500 2.15500 12.6400

Numbers of individuals/records for pairs of traits

1 2

1 "BF" 2285 844

2 "DMI" 844 955

Covariables

1"BF" nrec mean sdev min. max.

1 "idv(2)" 2285 6.10810 2.92868 2.00000 17.0000

2 "idade(1)" 2285 449.081 98.9421 306.000 609.000

2"DMI" nrec mean sdev min. max.

1 "idv(2)" 955 6.15079 3.05304 3.00000 17.0000

2 "idade(1)" 955 369.980 37.3543 267.000 511.000

Fixed effects

1 "BF" nlev

1 "gc" 65

2 "mn" 4

2 "DMI" nlev

1 "gc" 21

Random effects nlev

1 "animal" 3665 NRM

======== end of file ============================10-02-2015==========12:53====

======= Version 30-08-2013 ======================================= **KM** ====

Program WOMBAT: Estimates of covariance components

==============================================================================

Analysis type : "muv 2"

Data file : "DADOS.dat"

Pedigree file : "ReducedPedFile.dat"

Parameter file : "wombat.par"

No. of traits = 2 BF DMI

No. of records = 3240 2285 955

No. of parameters = 6

Maximum log L = -1398.389

-1/2 AIC & AICC = -1404.389 -1404.402

-1/2 BIC = -1422.550 "Penalty factor" = 4.027

Parameter estimates with approx. sampling erors

1 CHOL Z 1 1 0.797853 0.235940E-01

2 CHOL Z 1 2 0.123862 0.442625E-01

3 CHOL Z 2 2 0.628926 0.365955E-01

4 CHOL A 1 1 -0.499424 0.877193E-01

5 CHOL A 1 2 0.158470 0.716292E-01

6 CHOL A 2 2 -0.670110 0.917396E-01

Convergence criteria for last 3 iterates

Change in log likelihood = 18.005710 0.148475 0.000173

Change in parameter vector = 0.117468 0.017511 0.001377

Norm of gradient vector = 421.8041 27.7254 0.2601

Newton decrement = -34.1868 -0.2957 -0.0004

***** Estimates of residual covariances ************************************

Order of fit = 2

Covariance matrix

1 0.63657

2 0.98824E-01 0.41089

Eigenvalues of covariance matrix

Value 0.67 0.37

(%) 64.32 35.68

Trace 1.05

Matrix of correlations and variance ratios

1 0.6893

2 0.1932 0.5273

Covariances & correlations with approximate sampling errors

1 COVS Z 1 1 0.636570 0.376492E-01 vrat 0.689 0.046

2 COVS Z 1 2 0.988237E-01 0.357428E-01 corr 0.193 0.068

3 COVS Z 2 2 0.410890 0.471194E-01 vrat 0.527 0.068

***** Estimates for RE 1 "animal" ***************************************

No. of levels = 3665

Covariance structure = NRM

Order of fit = 2

Covariance matrix

1 0.28690

2 0.96172E-01 0.36830

Eigenvalues of covariance matrix

Value 0.43 0.22

(%) 65.94 34.06

Trace 0.66

Matrix of correlations and variance ratios

1 0.3107

2 0.2959 0.4727

Covariances & correlations with approximate sampling errors

4 COVS A 1 1 0.286901 0.480816E-01 vrat 0.311 0.046

5 COVS A 1 2 0.961721E-01 0.445355E-01 corr 0.296 0.130

6 COVS A 2 2 0.368304 0.646147E-01 vrat 0.473 0.068

***** Estimates of phenotypic covariances ***********************************

Covariance matrix

1 0.92347

2 0.19500 0.77919

Eigenvalues of covariance matrix

Value 1.06 0.64

(%) 62.21 37.79

Trace 1.70

Correlation matrix

1 1.0000

2 0.2299 1.0000

Covariances & correlations with approximate sampling errors

7 COVS T 1 1 0.923470 0.314975E-01

8 COVS T 1 2 0.194996 0.310140E-01 corr 0.230 0.035

9 COVS T 2 2 0.779193 0.408149E-01

======== end of file ============================10-02-2015==========12:53====

======= Version 30-08-2013 ======================================= **KM** ====

Program WOMBAT: Summary of Pedigree Information

==============================================================================

Two traits analysis (RF x DMI)

Analysis type : "muv 2"

Data file : "DADOS.dat"

Pedigree file : "ReducedPedFile.dat"

Parameter file : "wombat.par"

No. of animal IDs in data file = = 1930

No. of animal IDs in total = = 3174

*****Pedigree Structure for random effect : 1 ****************************

Original no. of animals = 3174

No. of animals after pruning = 3089

... proportion (%) remaining = 97.3

No. of levels w/out records = 1159

No. of levels with records = 1930 100.0%

... 1 record(s) = 1088 56.4%

... 2 record(s) = 842 43.6%

No. of animals w/out offspring = 1497 48.5%

No. of animals with offspring = 1592 51.5%

... and records = 433 14.0%

No. of animals with unknown sire = 60

No. of animals with unknown dam = 147

No. of animals with both parents unknown = 59

No. of animals with records =

... and unknown sire = 0

... and unknown dam = 0

... and both parents unknown = 0

No. of sires = 307

... with progeny in the data = 114

... with records & progeny in data = 44

No. of dams = 1285

... with progeny in the data = 792

... with records & progeny in data = 389

No. of animals with known/unpruned grand-parents

... with paternal grandsire = 2965

... with paternal granddam = 2839

... with maternal grandsire = 2840

... with maternal granddam = 2751

random effect no. = 1 NRM

no. of elements in NRM/GIN inverse 11801

log determinant = -2124.3928089367182

======== end of file ============================10-02-2015==========12:57====

======= Version 30-08-2013 ======================================= **KM** ====

Program WOMBAT: Summary of information from Set-up step

==============================================================================

Analysis type : "muv 2"

Data file : "DADOS.dat"

Pedigree file : "ReducedPedFile.dat"

Parameter file : "wombat.par"

No. of traits = 2

nrec mean sdev min. max.

1 "RF" 1817 5.08663 2.54341 0.00000 19.2000

2 "DMI" 955 6.83104 1.33500 2.15500 12.6400

Numbers of individuals/records for pairs of traits

1 2

1 "RF" 1817 842

2 "DMI" 842 955

Covariables

1"RF" nrec mean sdev min. max.

1 "idv(2)" 1817 6.09191 3.03138 2.00000 17.0000

2 "idade(1)" 1817 469.100 100.703 306.000 609.000

2"DMI" nrec mean sdev min. max.

1 "idv(2)" 955 6.15079 3.05304 3.00000 17.0000

2 "idade(1)" 955 369.980 37.3543 267.000 511.000

Fixed effects

1 "RF" nlev

1 "gc" 51

2 "mn" 3

2 "DMI" nlev

1 "gc" 21

Random effects nlev

1 "animal" 3089 NRM

======== end of file ============================10-02-2015==========12:57====

======= Version 30-08-2013 ======================================= **KM** ====

Program WOMBAT: Estimates of covariance components

==============================================================================

Analysis type : "muv 2"

Data file : "DADOS.dat"

Pedigree file : "ReducedPedFile.dat"

Parameter file : "wombat.par"

No. of traits = 2 RF DMI

No. of records = 2772 1817 955

No. of parameters = 6

Maximum log L = -2069.778

-1/2 AIC & AICC = -2075.778 -2075.794

-1/2 BIC = -2093.473 "Penalty factor" = 3.949

Parameter estimates with approx. sampling erors

1 CHOL Z 1 1 1.25983 0.463007E-01

2 CHOL Z 1 2 0.911604E-01 0.476723E-01

3 CHOL Z 2 2 0.632878 0.363023E-01

4 CHOL A 1 1 0.133986E-01 0.790020E-01

5 CHOL A 1 2 0.239262 0.754566E-01

6 CHOL A 2 2 -0.576069 0.101678

Convergence criteria for last 3 iterates

Change in log likelihood = 12.159858 0.105421 0.000040

Change in parameter vector = 0.099994 0.013453 0.000451

Norm of gradient vector = 195.7083 16.0833 0.2450

Newton decrement = -23.1630 -0.2082 -0.0001

***** Estimates of residual covariances ************************************

Order of fit = 2

Covariance matrix

1 1.5872

2 0.11485 0.40884

Eigenvalues of covariance matrix

Value 1.60 0.40

(%) 80.07 19.93

Trace 2.00

Matrix of correlations and variance ratios

1 0.6071

2 0.1426 0.5228

Covariances & correlations with approximate sampling errors

1 COVS Z 1 1 1.58717 0.116662 vrat 0.607 0.052

2 COVS Z 1 2 0.114847 0.609293E-01 corr 0.143 0.073

3 COVS Z 2 2 0.408844 0.473165E-01 vrat 0.523 0.068

***** Estimates for RE 1 "animal" ***************************************

No. of levels = 3089

Covariance structure = NRM

Order of fit = 2

Covariance matrix

1 1.0272

2 0.24249 0.37321

Eigenvalues of covariance matrix

Value 1.11 0.29

(%) 79.07 20.93

Trace 1.40

Matrix of correlations and variance ratios

1 0.3929

2 0.3917 0.4772

Covariances & correlations with approximate sampling errors

4 COVS A 1 1 1.02716 0.162295 vrat 0.393 0.052

5 COVS A 1 2 0.242490 0.807629E-01 corr 0.392 0.118

6 COVS A 2 2 0.373207 0.651595E-01 vrat 0.477 0.068

***** Estimates of phenotypic covariances ***********************************

Covariance matrix

1 2.6143

2 0.35734 0.78205

Eigenvalues of covariance matrix

Value 2.68 0.71

(%) 78.95 21.05

Trace 3.40

Correlation matrix

1 1.0000

2 0.2499 1.0000

Covariances & correlations with approximate sampling errors

7 COVS T 1 1 2.61433 0.103839

8 COVS T 1 2 0.357336 0.546234E-01 corr 0.250 0.035

9 COVS T 2 2 0.782051 0.410790E-01

======== end of file ============================10-02-2015==========12:57====

======= Version 30-08-2013 ======================================= **KM** ====

Program WOMBAT: Summary of Pedigree Information

==============================================================================

Two traits analysis (BW0.75 x ADG)

Analysis type : "muv 2"

Data file : "DADOS.dat"

Pedigree file : "ReducedPedFile.dat"

Parameter file : "wombat.par"

No. of animal IDs in data file = = 955

No. of animal IDs in total = = 2288

*****Pedigree Structure for random effect : 1 ****************************

Original no. of animals = 2288

No. of animals after pruning = 2204

... proportion (%) remaining = 96.3

No. of levels w/out records = 1249

No. of levels with records = 955 100.0%

... 2 record(s) = 955 100.0%

No. of animals w/out offspring = 828 37.6%

No. of animals with offspring = 1376 62.4%

... and records = 127 5.8%

No. of animals with unknown sire = 59

No. of animals with unknown dam = 145

No. of animals with both parents unknown = 58

No. of animals with records =

... and unknown sire = 0

... and unknown dam = 0

... and both parents unknown = 0

No. of sires = 296

... with progeny in the data = 78

... with records & progeny in data = 18

No. of dams = 1080

... with progeny in the data = 542

... with records & progeny in data = 98

No. of animals with known/unpruned grand-parents

... with paternal grandsire = 2083

... with paternal granddam = 1957

... with maternal grandsire = 1959

... with maternal granddam = 1872

random effect no. = 1 NRM

no. of elements in NRM/GIN inverse 8375

log determinant = -1487.8684515369519

======== end of file ============================10-02-2015==========13:26====

======= Version 30-08-2013 ======================================= **KM** ====

Program WOMBAT: Summary of information from Set-up step

==============================================================================

Analysis type : "muv 2"

Data file : "DADOS.dat"

Pedigree file : "ReducedPedFile.dat"

Parameter file : "wombat.par"

No. of traits = 2

nrec mean sdev min. max.

1 "BW0.75" 955 68.1510 10.1292 37.4020 94.5940

2 "ADG" 955 1.00846 0.260202 0.176000 1.71800

Numbers of individuals/records for pairs of traits

1 2

1 "BW0.75" 955 955

2 "ADG" 955 955

Covariables

1"BW0.75" nrec mean sdev min. max.

1 "idv(2)" 955 6.15079 3.05304 3.00000 17.0000

2 "idade(1)" 955 369.980 37.3543 267.000 511.000

2"ADG" nrec mean sdev min. max.

1 "idv(2)" 955 6.15079 3.05304 3.00000 17.0000

2 "idade(1)" 955 369.980 37.3543 267.000 511.000

Fixed effects

1 "BW0.75" nlev

1 "gc" 21

2 "mn" 4

2 "ADG" nlev

1 "gc" 21

Random effects nlev

1 "animal" 2204 NRM

======== end of file ============================10-02-2015==========13:26====

======= Version 30-08-2013 ======================================= **KM** ====

Program WOMBAT: Estimates of covariance components

==============================================================================

Analysis type : "muv 2"

Data file : "DADOS.dat"

Pedigree file : "ReducedPedFile.dat"

Parameter file : "wombat.par"

No. of traits = 2 BW0.75 ADG

No. of records = 1910 955 955

No. of parameters = 6

Maximum log L = -723.861

-1/2 AIC & AICC = -729.861 -729.883

-1/2 BIC = -746.444 "Penalty factor" = 3.764

Parameter estimates with approx. sampling erors

1 CHOL Z 1 1 1.31003 0.751468E-01

2 CHOL Z 1 2 0.166164E-01 0.108306E-01

3 CHOL Z 2 2 -2.13758 0.473234E-01

4 CHOL A 1 1 1.42180 0.876198E-01

5 CHOL A 1 2 0.755308E-01 0.118545E-01

6 CHOL A 2 2 -2.67117 0.157732

Convergence criteria for last 3 iterates

Change in log likelihood = 0.097781 0.002250 0.000150

Change in parameter vector = 0.009598 0.002184 0.000539

Norm of gradient vector = 38.4965 4.2578 1.1500

Newton decrement = -0.2174 -0.0061 -0.0004

***** Estimates of residual covariances ************************************

Order of fit = 2

Covariance matrix

1 13.736

2 0.61585E-01 0.14186E-01

Eigenvalues of covariance matrix

Value 13.74 0.01

(%) 99.90 0.10

Trace 13.75

Matrix of correlations and variance ratios

1 0.4443

2 0.1395 0.5749

Covariances & correlations with approximate sampling errors

1 COVS Z 1 1 13.7365 2.06450 vrat 0.444 0.077

2 COVS Z 1 2 0.615848E-01 0.423136E-01 corr 0.140 0.087

3 COVS Z 2 2 0.141861E-01 0.147236E-02 vrat 0.575 0.066

***** Estimates for RE 1 "animal" ***************************************

No. of levels = 2204

Covariance structure = NRM

Order of fit = 2

Covariance matrix

1 17.177

2 0.31304 0.10490E-01

Eigenvalues of covariance matrix

Value 17.18 0.00

(%) 99.97 0.03

Trace 17.19

Matrix of correlations and variance ratios

1 0.5557

2 0.7375 0.4251

Covariances & correlations with approximate sampling errors

4 COVS A 1 1 17.1774 3.01016 vrat 0.556 0.077

5 COVS A 1 2 0.313042 0.615591E-01 corr 0.737 0.086

6 COVS A 2 2 0.104896E-01 0.192907E-02 vrat 0.425 0.066

***** Estimates of phenotypic covariances ***********************************

Covariance matrix

1 30.914

2 0.37463 0.24676E-01

Eigenvalues of covariance matrix

Value 30.92 0.02

(%) 99.93 0.07

Trace 30.94

Correlation matrix

1 1.0000

2 0.4289 1.0000

Covariances & correlations with approximate sampling errors

7 COVS T 1 1 30.9138 1.71445

8 COVS T 1 2 0.374627 0.367750E-01 corr 0.429 0.030

9 COVS T 2 2 0.246756E-01 0.126924E-02

======== end of file ============================10-02-2015==========13:26====

======= Version 30-08-2013 ======================================= **KM** ====

Program WOMBAT: Summary of Pedigree Information

==============================================================================

Two traits analysis (WS X ADG)

Analysis type : "muv 2"

Data file : "DADOS.dat"

Pedigree file : "ReducedPedFile.dat"

Parameter file : "wombat.par"

No. of animal IDs in data file = = 8091

No. of animal IDs in total = = 8490

*****Pedigree Structure for random effect : 1 ****************************

Original no. of animals = 8490

No. of animals after pruning = 8433

... proportion (%) remaining = 99.3

No. of levels w/out records = 342

No. of levels with records = 8091 100.0%

... 1 record(s) = 7149 88.4%

... 2 record(s) = 942 11.6%

No. of animals w/out offspring = 6052 71.8%

No. of animals with offspring = 2381 28.2%

... and records = 2039 24.2%

No. of animals with unknown sire = 313

No. of animals with unknown dam = 384

No. of animals with both parents unknown = 312

No. of animals with records =

... and unknown sire = 1

... and unknown dam = 53

... and both parents unknown = 0

No. of sires = 325

... with progeny in the data = 320

... with records & progeny in data = 288

No. of dams = 2056

... with progeny in the data = 2056

... with records & progeny in data = 1751

No. of animals with known/unpruned grand-parents

... with paternal grandsire = 7358

... with paternal granddam = 7056

... with maternal grandsire = 6535

... with maternal granddam = 6468

random effect no. = 1 NRM

no. of elements in NRM/GIN inverse 31838

log determinant = -5688.7609053187680

random effect no. = 2 IDE

no. of elements in NRM/GIN inverse 0

log determinant = 0.0000000000000000

======== end of file ============================10-02-2015==========13:13====

======= Version 30-08-2013 ======================================= **KM** ====

Program WOMBAT: Summary of information from Set-up step

==============================================================================

Analysis type : "muv 2"

Data file : "DADOS.dat"

Pedigree file : "ReducedPedFile.dat"

Parameter file : "wombat.par"

No. of traits = 2

nrec mean sdev min. max.

1 "WS" 8078 299.703 49.6462 160.030 489.760

2 "ADG" 955 1.00846 0.260202 0.176000 1.71800

Numbers of individuals/records for pairs of traits

1 2

1 "WS" 8078 942

2 "ADG" 942 955

Covariables

1"WS" nrec mean sdev min. max.

1 "idv(2)" 8078 6.63568 3.07616 2.00000 18.0000

2 "idade(1)" 8078 473.414 105.028 293.000 645.000

2"ADG" nrec mean sdev min. max.

1 "idv(2)" 955 6.15079 3.05304 3.00000 17.0000

2 "idade(1)" 955 369.980 37.3543 267.000 511.000

Fixed effects

1 "WS" nlev

1 "gc" 201

2 "mn" 4

2 "ADG" nlev

1 "gc" 21

Random effects nlev

1 "animal" 8433 NRM

2 "peanim" 2107 IDE

======== end of file ============================10-02-2015==========13:13====

======= Version 30-08-2013 ======================================= **KM** ====

Program WOMBAT: Estimates of covariance components

==============================================================================

Analysis type : "muv 2"

Data file : "DADOS.dat"

Pedigree file : "ReducedPedFile.dat"

Parameter file : "wombat.par"

No. of traits = 2 WS ADG

No. of records = 9033 8078 955

No. of parameters = 7

Maximum log L = -28668.087

-1/2 AIC & AICC = -28675.087 -28675.094

-1/2 BIC = -28699.877 "Penalty factor" = 4.541

Parameter estimates with approx. sampling erors

1 CHOL Z 1 1 3.01764 0.213193E-01

2 CHOL Z 1 2 0.636308E-01 0.593073E-02

3 CHOL Z 2 2 -2.30089 0.414059E-01

4 CHOL A 1 1 2.90101 0.460319E-01

5 CHOL A 1 2 0.760086E-01 0.792423E-02

6 CHOL A 2 2 -3.02678 0.192864

7 CHOL B 1 1 2.33878 0.474784E-01

Convergence criteria for last 3 iterates

Change in log likelihood = 0.234479 0.005661 0.000284

Change in parameter vector = 0.003164 0.001150 0.000303

Norm of gradient vector = 94.4932 12.2173 2.6729

Newton decrement = -0.5180 -0.0129 -0.0006

***** Estimates of residual covariances ************************************

Order of fit = 2

Covariance matrix

1 417.91

2 1.3008 0.14083E-01

Eigenvalues of covariance matrix

Value 417.92 0.01

(%) 100.00 0.00

Trace 417.93

Matrix of correlations and variance ratios

1 0.4880

2 0.5362 0.6341

Covariances & correlations with approximate sampling errors

1 COVS Z 1 1 417.914 17.8193 vrat 0.488 0.027

2 COVS Z 1 2 1.30080 0.130317 corr 0.536 0.039

3 COVS Z 2 2 0.140828E-01 0.113596E-02 vrat 0.634 0.053

***** Estimates for RE 1 "animal" ***************************************

No. of levels = 8433

Covariance structure = NRM

Order of fit = 2

Covariance matrix

1 330.97

2 1.3828 0.81268E-02

Eigenvalues of covariance matrix

Value 330.97 0.00

(%) 100.00 0.00

Trace 330.97

Matrix of correlations and variance ratios

1 0.3865

2 0.8431 0.3659

Covariances & correlations with approximate sampling errors

4 COVS A 1 1 330.965 30.4699 vrat 0.386 0.030

5 COVS A 1 2 1.38278 0.169729 corr 0.843 0.058

6 COVS A 2 2 0.812679E-02 0.135652E-02 vrat 0.366 0.053

***** Estimates for RE 2 "peanim" ***************************************

No. of levels = 2107

Covariance structure = IDE

Order of fit = 1

Covariance matrix

1 107.51

Matrix of correlations and variance ratios

1 0.1255

Covariances & correlations with approximate sampling errors

7 COVS B 1 1 107.507 10.2085 vrat 0.126 0.012

***** Estimates of phenotypic covariances ***********************************

Covariance matrix

1 856.39

2 2.6836 0.22210E-01

Eigenvalues of covariance matrix

Value 856.39 0.01

(%) 100.00 0.00

Trace 856.41

Correlation matrix

1 1.0000

2 0.6153 1.0000

Covariances & correlations with approximate sampling errors

8 COVS T 1 1 856.386 18.7337

9 COVS T 1 2 2.68358 0.125604 corr 0.615 0.019

10 COVS T 2 2 0.222096E-01 0.100037E-02

======== end of file ============================10-02-2015==========13:13====

======= Version 30-08-2013 ======================================= **KM** ====

Program WOMBAT: Summary of Pedigree Information

==============================================================================

Two traits analysis (HH x ADG)

Analysis type : "muv 2"

Data file : "DADOS.dat"

Pedigree file : "ReducedPedFile.dat"

Parameter file : "wombat.par"

No. of animal IDs in data file = = 6560

No. of animal IDs in total = = 7100

*****Pedigree Structure for random effect : 1 ****************************

Original no. of animals = 7100

No. of animals after pruning = 7017

... proportion (%) remaining = 98.8

No. of levels w/out records = 457

No. of levels with records = 6560 100.0%

... 1 record(s) = 5617 85.6%

... 2 record(s) = 943 14.4%

No. of animals w/out offspring = 4829 68.8%

No. of animals with offspring = 2188 31.2%

... and records = 1731 24.7%

No. of animals with unknown sire = 148

No. of animals with unknown dam = 237

No. of animals with both parents unknown = 148

No. of animals with records =

... and unknown sire = 0

... and unknown dam = 18

... and both parents unknown = 0

No. of sires = 320

... with progeny in the data = 283

... with records & progeny in data = 240

No. of dams = 1868

... with progeny in the data = 1837

... with records & progeny in data = 1491

No. of animals with known/unpruned grand-parents

... with paternal grandsire = 6738

... with paternal granddam = 6484

... with maternal grandsire = 6339

... with maternal granddam = 6111

random effect no. = 1 NRM

no. of elements in NRM/GIN inverse 26822

log determinant = -4814.8039049931958

======== end of file ============================10-02-2015==========13:16====

======= Version 30-08-2013 ======================================= **KM** ====

Program WOMBAT: Summary of information from Set-up step

==============================================================================

Analysis type : "muv 2"

Data file : "DADOS.dat"

Pedigree file : "ReducedPedFile.dat"

Parameter file : "wombat.par"

No. of traits = 2

nrec mean sdev min. max.

1 "HH" 6548 132.269 5.43028 100.000 149.000

2 "ADG" 955 1.00846 0.260202 0.176000 1.71800

Numbers of individuals/records for pairs of traits

1 2

1 "HH" 6548 943

2 "ADG" 943 955

Covariables

1"HH" nrec mean sdev min. max.

1 "idv(2)" 6548 6.40043 2.93455 2.00000 17.0000

2 "idade(1)" 6548 472.701 107.333 293.000 645.000

2"ADG" nrec mean sdev min. max.

1 "idv(2)" 955 6.15079 3.05304 3.00000 17.0000

2 "idade(1)" 955 369.980 37.3543 267.000 511.000

Fixed effects

1 "HH" nlev

1 "gc" 169

2 "mn" 4

2 "ADG" nlev

1 "gc" 21

Random effects nlev

1 "animal" 7017 NRM

======== end of file ============================10-02-2015==========13:16====

======= Version 30-08-2013 ======================================= **KM** ====

Program WOMBAT: Estimates of covariance components

==============================================================================

Analysis type : "muv 2"

Data file : "DADOS.dat"

Pedigree file : "ReducedPedFile.dat"

Parameter file : "wombat.par"

No. of traits = 2 HH ADG

No. of records = 7503 6548 955

No. of parameters = 6

Maximum log L = -10109.855

-1/2 AIC & AICC = -10115.855 -10115.861

-1/2 BIC = -10136.544 "Penalty factor" = 4.448

Parameter estimates with approx. sampling erors

1 CHOL Z 1 1 0.887712 0.270507E-01

2 CHOL Z 1 2 0.122189E-01 0.912380E-02

3 CHOL Z 2 2 -2.13032 0.492178E-01

4 CHOL A 1 1 1.13154 0.311139E-01

5 CHOL A 1 2 0.631666E-01 0.976935E-02

6 CHOL A 2 2 -2.52521 0.127701

Convergence criteria for last 3 iterates

Change in log likelihood = 0.047280 0.000612 0.000014

Change in parameter vector = 0.003303 0.000423 0.000055

Norm of gradient vector = 36.3034 0.8785 0.1339

Newton decrement = -0.0874 -0.0011 -0.0000

***** Estimates of residual covariances ************************************

Order of fit = 2

Covariance matrix

1 5.9028

2 0.29687E-01 0.14263E-01

Eigenvalues of covariance matrix

Value 5.90 0.01

(%) 99.76 0.24

Trace 5.92

Matrix of correlations and variance ratios

1 0.3804

2 0.1023 0.5784

Covariances & correlations with approximate sampling errors

1 COVS Z 1 1 5.90278 0.319349 vrat 0.380 0.026

2 COVS Z 1 2 0.296866E-01 0.223283E-01 corr 0.102 0.075

3 COVS Z 2 2 0.142626E-01 0.145839E-02 vrat 0.578 0.065

***** Estimates for RE 1 "animal" ***************************************

No. of levels = 7017

Covariance structure = NRM

Order of fit = 2

Covariance matrix

1 9.6126

2 0.19584 0.10397E-01

Eigenvalues of covariance matrix

Value 9.62 0.01

(%) 99.93 0.07

Trace 9.62

Matrix of correlations and variance ratios

1 0.6196

2 0.6195 0.4216

Covariances & correlations with approximate sampling errors

4 COVS A 1 1 9.61260 0.598171 vrat 0.620 0.026

5 COVS A 1 2 0.195843 0.317896E-01 corr 0.619 0.082

6 COVS A 2 2 0.103967E-01 0.189779E-02 vrat 0.422 0.065

***** Estimates of phenotypic covariances ***********************************

Covariance matrix

1 15.515

2 0.22553 0.24659E-01

Eigenvalues of covariance matrix

Value 15.52 0.02

(%) 99.86 0.14

Trace 15.54

Correlation matrix

1 1.0000

2 0.3646 1.0000

Covariances & correlations with approximate sampling errors

7 COVS T 1 1 15.5154 0.386474

8 COVS T 1 2 0.225529 0.220377E-01 corr 0.365 0.031

9 COVS T 2 2 0.246592E-01 0.125643E-02

======== end of file ============================10-02-2015==========13:17====

======= Version 30-08-2013 ======================================= **KM** ====

Program WOMBAT: Summary of Pedigree Information

==============================================================================

Two traits analysis (CC x ADG)

Analysis type : "muv 2"

Data file : "DADOS.dat"

Pedigree file : "ReducedPedFile.dat"

Parameter file : "wombat.par"

No. of animal IDs in data file = = 3943

No. of animal IDs in total = = 5418

*****Pedigree Structure for random effect : 1 ****************************

Original no. of animals = 5418

No. of animals after pruning = 5330

... proportion (%) remaining = 98.4

No. of levels w/out records = 1387

No. of levels with records = 3943 100.0%

... 1 record(s) = 3055 77.5%

... 2 record(s) = 888 22.5%

No. of animals w/out offspring = 3311 62.1%

No. of animals with offspring = 2019 37.9%

... and records = 632 11.9%

No. of animals with unknown sire = 94

No. of animals with unknown dam = 188

No. of animals with both parents unknown = 94

No. of animals with records =

... and unknown sire = 0

... and unknown dam = 0

... and both parents unknown = 0

No. of sires = 318

... with progeny in the data = 255

... with records & progeny in data = 217

No. of dams = 1701

... with progeny in the data = 1508

... with records & progeny in data = 412

No. of animals with known/unpruned grand-parents

... with paternal grandsire = 5142

... with paternal granddam = 4954

... with maternal grandsire = 4953

... with maternal granddam = 4738

random effect no. = 1 NRM

no. of elements in NRM/GIN inverse 20464

log determinant = -3671.8274443710275

======== end of file ============================10-02-2015==========13:19====

======= Version 30-08-2013 ======================================= **KM** ====

Program WOMBAT: Summary of information from Set-up step

==============================================================================

Analysis type : "muv 2"

Data file : "DADOS.dat"

Pedigree file : "ReducedPedFile.dat"

Parameter file : "wombat.par"

No. of traits = 2

nrec mean sdev min. max.

1 "CC" 3876 164.140 8.72028 128.000 192.000

2 "ADG" 955 1.00846 0.260202 0.176000 1.71800

Numbers of individuals/records for pairs of traits

1 2

1 "CC" 3876 888

2 "ADG" 888 955

Covariables

1"CC" nrec mean sdev min. max.

1 "idv(2)" 3876 6.27632 2.87660 2.00000 17.0000

2 "idade(1)" 3876 431.403 109.187 293.000 725.000

2"ADG" nrec mean sdev min. max.

1 "idv(2)" 955 6.15079 3.05304 3.00000 17.0000

2 "idade(1)" 955 369.980 37.3543 267.000 511.000

Fixed effects

1 "CC" nlev

1 "gc" 100

2 "mn" 4

2 "ADG" nlev

1 "gc" 21

Random effects nlev

1 "animal" 5330 NRM

======== end of file ============================10-02-2015==========13:19====

======= Version 30-08-2013 ======================================= **KM** ====

Program WOMBAT: Estimates of covariance components

==============================================================================

Analysis type : "muv 2"

Data file : "DADOS.dat"

Pedigree file : "ReducedPedFile.dat"

Parameter file : "wombat.par"

No. of traits = 2 CC ADG

No. of records = 4831 3876 955

No. of parameters = 6

Maximum log L = -7337.391

-1/2 AIC & AICC = -7343.391 -7343.399

-1/2 BIC = -7362.757 "Penalty factor" = 4.228

Parameter estimates with approx. sampling erors

1 CHOL Z 1 1 1.60405 0.240507E-01

2 CHOL Z 1 2 0.417124E-01 0.692305E-02

3 CHOL Z 2 2 -2.18015 0.453671E-01

4 CHOL A 1 1 1.22948 0.684887E-01

5 CHOL A 1 2 0.801441E-01 0.108172E-01

6 CHOL A 2 2 -2.80624 0.189795

Convergence criteria for last 3 iterates

Change in log likelihood = 0.018944 0.000585 0.000022

Change in parameter vector = 0.002866 0.000539 0.000102

Norm of gradient vector = 7.7202 0.8151 0.1660

Newton decrement = -0.0329 -0.0010 -0.0000

***** Estimates of residual covariances ************************************

Order of fit = 2

Covariance matrix

1 24.732

2 0.20744 0.14514E-01

Eigenvalues of covariance matrix

Value 24.73 0.01

(%) 99.95 0.05

Trace 24.75

Matrix of correlations and variance ratios

1 0.6790

2 0.3462 0.5903

Covariances & correlations with approximate sampling errors

1 COVS Z 1 1 24.7323 1.18966 vrat 0.679 0.039

2 COVS Z 1 2 0.207442 0.363095E-01 corr 0.346 0.050

3 COVS Z 2 2 0.145144E-01 0.138571E-02 vrat 0.590 0.061

***** Estimates for RE 1 "animal" ***************************************

No. of levels = 5330

Covariance structure = NRM

Order of fit = 2

Covariance matrix

1 11.693

2 0.27405 0.10075E-01

Eigenvalues of covariance matrix

Value 11.70 0.00

(%) 99.97 0.03

Trace 11.70

Matrix of correlations and variance ratios

1 0.3210

2 0.7984 0.4097

Covariances & correlations with approximate sampling errors

4 COVS A 1 1 11.6926 1.60163 vrat 0.321 0.039

5 COVS A 1 2 0.274048 0.450546E-01 corr 0.798 0.079

6 COVS A 2 2 0.100751E-01 0.175924E-02 vrat 0.410 0.061

***** Estimates of phenotypic covariances ***********************************

Covariance matrix

1 36.425

2 0.48149 0.24590E-01

Eigenvalues of covariance matrix

Value 36.43 0.02

(%) 99.95 0.05

Trace 36.45

Correlation matrix

1 1.0000

2 0.5088 1.0000

Covariances & correlations with approximate sampling errors

7 COVS T 1 1 36.4249 0.994166

8 COVS T 1 2 0.481491 0.324423E-01 corr 0.509 0.025

9 COVS T 2 2 0.245895E-01 0.121673E-02

======== end of file ============================10-02-2015==========13:19====

======= Version 30-08-2013 ======================================= **KM** ====

Program WOMBAT: Summary of Pedigree Information

==============================================================================

Two traits analysis (LEA x ADG)

Analysis type : "muv 2"

Data file : "DADOS.dat"

Pedigree file : "ReducedPedFile.dat"

Parameter file : "wombat.par"

No. of animal IDs in data file = = 2396

No. of animal IDs in total = = 3756

*****Pedigree Structure for random effect : 1 ****************************

Original no. of animals = 3756

No. of animals after pruning = 3665

... proportion (%) remaining = 97.6

No. of levels w/out records = 1269

No. of levels with records = 2396 100.0%

... 1 record(s) = 1554 64.9%

... 2 record(s) = 842 35.1%

No. of animals w/out offspring = 1913 52.2%

No. of animals with offspring = 1752 47.8%

... and records = 483 13.2%

No. of animals with unknown sire = 64

No. of animals with unknown dam = 161

No. of animals with both parents unknown = 64

No. of animals with records =

... and unknown sire = 0

... and unknown dam = 0

... and both parents unknown = 0

No. of sires = 313

... with progeny in the data = 180

... with records & progeny in data = 85

No. of dams = 1439

... with progeny in the data = 1042

... with records & progeny in data = 392

No. of animals with known/unpruned grand-parents

... with paternal grandsire = 3520

... with paternal granddam = 3382

... with maternal grandsire = 3391

... with maternal granddam = 3267

random effect no. = 1 NRM

no. of elements in NRM/GIN inverse 14038

log determinant = -2522.5521618264124

======== end of file ============================10-02-2015==========13:21====

======= Version 30-08-2013 ======================================= **KM** ====

Program WOMBAT: Summary of information from Set-up step

==============================================================================

Analysis type : "muv 2"

Data file : "DADOS.dat"

Pedigree file : "ReducedPedFile.dat"

Parameter file : "wombat.par"

No. of traits = 2

nrec mean sdev min. max.

1 "LEA" 2283 51.4305 8.89390 21.4000 83.4000

2 "ADG" 955 1.00846 0.260202 0.176000 1.71800

Numbers of individuals/records for pairs of traits

1 2

1 "LEA" 2283 842

2 "ADG" 842 955

Covariables

1"LEA" nrec mean sdev min. max.

1 "idv(2)" 2283 6.11082 2.92852 2.00000 17.0000

2 "idade(1)" 2283 448.951 98.8881 306.000 609.000

2"ADG" nrec mean sdev min. max.

1 "idv(2)" 955 6.15079 3.05304 3.00000 17.0000

2 "idade(1)" 955 369.980 37.3543 267.000 511.000

Fixed effects

1 "LEA" nlev

1 "gc" 65

2 "mn" 4

2 "ADG" nlev

1 "gc" 21

Random effects nlev

1 "animal" 3665 NRM

======== end of file ============================10-02-2015==========13:21====

======= Version 30-08-2013 ======================================= **KM** ====

Program WOMBAT: Estimates of covariance components

==============================================================================

Analysis type : "muv 2"

Data file : "DADOS.dat"

Pedigree file : "ReducedPedFile.dat"

Parameter file : "wombat.par"

No. of traits = 2 LEA ADG

No. of records = 3238 2283 955

No. of parameters = 6

Maximum log L = -3772.123

-1/2 AIC & AICC = -3778.123 -3778.136

-1/2 BIC = -3796.282 "Penalty factor" = 4.026

Parameter estimates with approx. sampling erors

1 CHOL Z 1 1 1.48327 0.400334E-01

2 CHOL Z 1 2 0.397050E-01 0.892057E-02

3 CHOL Z 2 2 -2.17604 0.518761E-01

4 CHOL A 1 1 1.43023 0.665145E-01

5 CHOL A 1 2 0.417327E-01 0.126518E-01

6 CHOL A 2 2 -2.38613 0.103524

Convergence criteria for last 3 iterates

Change in log likelihood = 2.628095 0.016838 0.000293

Change in parameter vector = 0.013722 0.003032 0.000281

Norm of gradient vector = 356.5703 9.7621 1.7096

Newton decrement = -5.0715 -0.0302 -0.0005

***** Estimates of residual covariances ************************************

Order of fit = 2

Covariance matrix

1 19.425

2 0.17499 0.14456E-01

Eigenvalues of covariance matrix

Value 19.43 0.01

(%) 99.93 0.07

Trace 19.44

Matrix of correlations and variance ratios

1 0.5265

2 0.3302 0.5862

Covariances & correlations with approximate sampling errors

1 COVS Z 1 1 19.4247 1.55528 vrat 0.526 0.051

2 COVS Z 1 2 0.174994 0.411132E-01 corr 0.330 0.069

3 COVS Z 2 2 0.144564E-01 0.144248E-02 vrat 0.586 0.064

***** Estimates for RE 1 "animal" ***************************************

No. of levels = 3665

Covariance structure = NRM

Order of fit = 2

Covariance matrix

1 17.470

2 0.17443 0.10203E-01

Eigenvalues of covariance matrix

Value 17.47 0.01

(%) 99.95 0.05

Trace 17.48

Matrix of correlations and variance ratios

1 0.4735

2 0.4132 0.4138

Covariances & correlations with approximate sampling errors

4 COVS A 1 1 17.4697 2.32398 vrat 0.474 0.051

5 COVS A 1 2 0.174429 0.562652E-01 corr 0.413 0.116

6 COVS A 2 2 0.102028E-01 0.186284E-02 vrat 0.414 0.064

***** Estimates of phenotypic covariances ***********************************

Covariance matrix

1 36.894

2 0.34942 0.24659E-01

Eigenvalues of covariance matrix

Value 36.90 0.02

(%) 99.94 0.06

Trace 36.92

Correlation matrix

1 1.0000

2 0.3663 1.0000

Covariances & correlations with approximate sampling errors

7 COVS T 1 1 36.8944 1.38075

8 COVS T 1 2 0.349423 0.370375E-01 corr 0.366 0.034

9 COVS T 2 2 0.246593E-01 0.125462E-02

======== end of file ============================10-02-2015==========13:21====

======= Version 30-08-2013 ======================================= **KM** ====

Program WOMBAT: Summary of Pedigree Information

==============================================================================

Two traits analysis (BF x ADG)

Analysis type : "muv 2"

Data file : "DADOS.dat"

Pedigree file : "ReducedPedFile.dat"

Parameter file : "wombat.par"

No. of animal IDs in data file = = 2396

No. of animal IDs in total = = 3756

*****Pedigree Structure for random effect : 1 ****************************

Original no. of animals = 3756

No. of animals after pruning = 3665

... proportion (%) remaining = 97.6

No. of levels w/out records = 1269

No. of levels with records = 2396 100.0%

... 1 record(s) = 1552 64.8%

... 2 record(s) = 844 35.2%

No. of animals w/out offspring = 1913 52.2%

No. of animals with offspring = 1752 47.8%

... and records = 483 13.2%

No. of animals with unknown sire = 64

No. of animals with unknown dam = 161

No. of animals with both parents unknown = 64

No. of animals with records =

... and unknown sire = 0

... and unknown dam = 0

... and both parents unknown = 0

No. of sires = 313

... with progeny in the data = 180

... with records & progeny in data = 85

No. of dams = 1439

... with progeny in the data = 1042

... with records & progeny in data = 392

No. of animals with known/unpruned grand-parents

... with paternal grandsire = 3520

... with paternal granddam = 3382

... with maternal grandsire = 3391

... with maternal granddam = 3267

random effect no. = 1 NRM

no. of elements in NRM/GIN inverse 14038

log determinant = -2522.5521618264124

======== end of file ============================10-02-2015==========13:22====

======= Version 30-08-2013 ======================================= **KM** ====

Program WOMBAT: Summary of information from Set-up step

==============================================================================

Analysis type : "muv 2"

Data file : "DADOS.dat"

Pedigree file : "ReducedPedFile.dat"

Parameter file : "wombat.par"

No. of traits = 2

nrec mean sdev min. max.

1 "BF" 2285 1.75922 1.42798 0.00000 10.5000

2 "ADG" 955 1.00846 0.260202 0.176000 1.71800

Numbers of individuals/records for pairs of traits

1 2

1 "BF" 2285 844

2 "ADG" 844 955

Covariables

1"BF" nrec mean sdev min. max.

1 "idv(2)" 2285 6.10810 2.92868 2.00000 17.0000

2 "idade(1)" 2285 449.081 98.9421 306.000 609.000

2"ADG" nrec mean sdev min. max.

1 "idv(2)" 955 6.15079 3.05304 3.00000 17.0000

2 "idade(1)" 955 369.980 37.3543 267.000 511.000

Fixed effects

1 "BF" nlev

1 "gc" 65

2 "mn" 4

2 "ADG" nlev

1 "gc" 21

Random effects nlev

1 "animal" 3665 NRM

======== end of file ============================10-02-2015==========13:22====

======= Version 30-08-2013 ======================================= **KM** ====

Program WOMBAT: Estimates of covariance components

==============================================================================

Analysis type : "muv 2"

Data file : "DADOS.dat"

Pedigree file : "ReducedPedFile.dat"

Parameter file : "wombat.par"

No. of traits = 2 BF ADG

No. of records = 3240 2285 955

No. of parameters = 6

Maximum log L = 192.146

-1/2 AIC & AICC = 186.146 186.133

-1/2 BIC = 167.985 "Penalty factor" = 4.027

Parameter estimates with approx. sampling erors

1 CHOL Z 1 1 -0.226367 0.295890E-01

2 CHOL Z 1 2 0.188083E-01 0.791614E-02

3 CHOL Z 2 2 -2.14990 0.544742E-01

4 CHOL A 1 1 -0.623789 0.837067E-01

5 CHOL A 1 2 0.157089E-01 0.143551E-01

6 CHOL A 2 2 -2.27783 0.941444E-01

Convergence criteria for last 3 iterates

Change in log likelihood = 12.072887 0.027733 0.000014

Change in parameter vector = 0.053402 0.005021 0.000112

Norm of gradient vector = 285.9265 10.7281 0.3696

Newton decrement = -22.9874 -0.0560 -0.0000

***** Estimates of residual covariances ************************************

Order of fit = 2

Covariance matrix

1 0.63589

2 0.14998E-01 0.13925E-01

Eigenvalues of covariance matrix

Value 0.64 0.01

(%) 97.91 2.09

Trace 0.65

Matrix of correlations and variance ratios

1 0.6889

2 0.1594 0.5642

Covariances & correlations with approximate sampling errors

1 COVS Z 1 1 0.635887 0.376305E-01 vrat 0.689 0.046

2 COVS Z 1 2 0.149982E-01 0.636104E-02 corr 0.159 0.066

3 COVS Z 2 2 0.139249E-01 0.149907E-02 vrat 0.564 0.068

***** Estimates for RE 1 "animal" ***************************************

No. of levels = 3665

Covariance structure = NRM

Order of fit = 2

Covariance matrix

1 0.28720

2 0.84185E-02 0.10754E-01

Eigenvalues of covariance matrix

Value 0.29 0.01

(%) 96.48 3.52

Trace 0.30

Matrix of correlations and variance ratios

1 0.3111

2 0.1515 0.4358

Covariances & correlations with approximate sampling errors

4 COVS A 1 1 0.287199 0.480810E-01 vrat 0.311 0.046

5 COVS A 1 2 0.841853E-02 0.777597E-02 corr 0.151 0.137

6 COVS A 2 2 0.107544E-01 0.199723E-02 vrat 0.436 0.068

***** Estimates of phenotypic covariances ***********************************

Covariance matrix

1 0.92309

2 0.23417E-01 0.24679E-01

Eigenvalues of covariance matrix

Value 0.92 0.02

(%) 97.46 2.54

Trace 0.95

Correlation matrix

1 1.0000

2 0.1551 1.0000

Covariances & correlations with approximate sampling errors

7 COVS T 1 1 0.923086 0.314832E-01

8 COVS T 1 2 0.234167E-01 0.549687E-02 corr 0.155 0.036

9 COVS T 2 2 0.246793E-01 0.128168E-02

======== end of file ============================10-02-2015==========13:22====

======= Version 30-08-2013 ======================================= **KM** ====

Program WOMBAT: Summary of Pedigree Information

==============================================================================

Two traits analysis (RF x ADG)

Analysis type : "muv 2"

Data file : "DADOS.dat"

Pedigree file : "ReducedPedFile.dat"

Parameter file : "wombat.par"

No. of animal IDs in data file = = 1930

No. of animal IDs in total = = 3174

*****Pedigree Structure for random effect : 1 ****************************

Original no. of animals = 3174

No. of animals after pruning = 3089

... proportion (%) remaining = 97.3

No. of levels w/out records = 1159

No. of levels with records = 1930 100.0%

... 1 record(s) = 1088 56.4%

... 2 record(s) = 842 43.6%

No. of animals w/out offspring = 1497 48.5%

No. of animals with offspring = 1592 51.5%

... and records = 433 14.0%

No. of animals with unknown sire = 60

No. of animals with unknown dam = 147

No. of animals with both parents unknown = 59

No. of animals with records =

... and unknown sire = 0

... and unknown dam = 0

... and both parents unknown = 0

No. of sires = 307

... with progeny in the data = 114

... with records & progeny in data = 44

No. of dams = 1285

... with progeny in the data = 792

... with records & progeny in data = 389

No. of animals with known/unpruned grand-parents

... with paternal grandsire = 2965

... with paternal granddam = 2839

... with maternal grandsire = 2840

... with maternal granddam = 2751

random effect no. = 1 NRM

no. of elements in NRM/GIN inverse 11801

log determinant = -2124.3928089367182

======== end of file ============================10-02-2015==========13:24====

======= Version 30-08-2013 ======================================= **KM** ====

Program WOMBAT: Summary of information from Set-up step

==============================================================================

Analysis type : "muv 2"

Data file : "DADOS.dat"

Pedigree file : "ReducedPedFile.dat"

Parameter file : "wombat.par"

No. of traits = 2

nrec mean sdev min. max.

1 "RF" 1817 5.08663 2.54341 0.00000 19.2000

2 "ADG" 955 1.00846 0.260202 0.176000 1.71800

Numbers of individuals/records for pairs of traits

1 2

1 "RF" 1817 842

2 "ADG" 842 955

Covariables

1"RF" nrec mean sdev min. max.

1 "idv(2)" 1817 6.09191 3.03138 2.00000 17.0000

2 "idade(1)" 1817 469.100 100.703 306.000 609.000

2"ADG" nrec mean sdev min. max.

1 "idv(2)" 955 6.15079 3.05304 3.00000 17.0000

2 "idade(1)" 955 369.980 37.3543 267.000 511.000

Fixed effects

1 "RF" nlev

1 "gc" 51

2 "mn" 3

2 "ADG" nlev

1 "gc" 21

Random effects nlev

1 "animal" 3089 NRM

======== end of file ============================10-02-2015==========13:24====

======= Version 30-08-2013 ======================================= **KM** ====

Program WOMBAT: Estimates of covariance components

==============================================================================

Analysis type : "muv 2"

Data file : "DADOS.dat"

Pedigree file : "ReducedPedFile.dat"

Parameter file : "wombat.par"

No. of traits = 2 RF ADG

No. of records = 2772 1817 955

No. of parameters = 6

Maximum log L = -471.218

-1/2 AIC & AICC = -477.218 -477.233

-1/2 BIC = -494.912 "Penalty factor" = 3.949

Parameter estimates with approx. sampling erors

1 CHOL Z 1 1 0.231099 0.367901E-01

2 CHOL Z 1 2 0.231625E-01 0.847179E-02

3 CHOL Z 2 2 -2.16412 0.554473E-01

4 CHOL A 1 1 0.112243E-01 0.793802E-01

5 CHOL A 1 2 0.256062E-01 0.136334E-01

6 CHOL A 2 2 -2.28351 0.951534E-01

Convergence criteria for last 3 iterates

Change in log likelihood = 7.120894 0.023716 0.000002

Change in parameter vector = 0.031712 0.002561 0.000028

Norm of gradient vector = 250.8698 12.6060 0.0894

Newton decrement = -13.7230 -0.0472 -0.0000

***** Estimates of residual covariances ************************************

Order of fit = 2

Covariance matrix

1 1.5876

2 0.29184E-01 0.13727E-01

Eigenvalues of covariance matrix

Value 1.59 0.01

(%) 99.18 0.82

Trace 1.60

Matrix of correlations and variance ratios

1 0.6082

2 0.1977 0.5541

Covariances & correlations with approximate sampling errors

1 COVS Z 1 1 1.58756 0.116813 vrat 0.608 0.053

2 COVS Z 1 2 0.291843E-01 0.108528E-01 corr 0.198 0.071

3 COVS Z 2 2 0.137272E-01 0.150104E-02 vrat 0.554 0.068

***** Estimates for RE 1 "animal" ***************************************

No. of levels = 3089

Covariance structure = NRM

Order of fit = 2

Covariance matrix

1 1.0227

2 0.25895E-01 0.11045E-01

Eigenvalues of covariance matrix

Value 1.02 0.01

(%) 99.00 1.00

Trace 1.03

Matrix of correlations and variance ratios

1 0.3918

2 0.2437 0.4459

Covariances & correlations with approximate sampling errors

4 COVS A 1 1 1.02270 0.162365 vrat 0.392 0.053

5 COVS A 1 2 0.258952E-01 0.141697E-01 corr 0.244 0.126

6 COVS A 2 2 0.110446E-01 0.202161E-02 vrat 0.446 0.068

***** Estimates of phenotypic covariances ***********************************

Covariance matrix

1 2.6103

2 0.55080E-01 0.24772E-01

Eigenvalues of covariance matrix

Value 2.61 0.02

(%) 99.10 0.90

Trace 2.64

Correlation matrix

1 1.0000

2 0.2166 1.0000

Covariances & correlations with approximate sampling errors

7 COVS T 1 1 2.61026 0.103648

8 COVS T 1 2 0.550795E-01 0.964087E-02 corr 0.217 0.036

9 COVS T 2 2 0.247719E-01 0.129221E-02

======== end of file ============================10-02-2015==========13:24====

======= Version 30-08-2013 ======================================= **KM** ====

Program WOMBAT: Summary of Pedigree Information

==============================================================================

Two traits analysis (WS x BW0.75)

Analysis type : "muv 2"

Data file : "DADOS.dat"

Pedigree file : "ReducedPedFile.dat"

Parameter file : "wombat.par"

No. of animal IDs in data file = = 8091

No. of animal IDs in total = = 8490

*****Pedigree Structure for random effect : 1 ****************************

Original no. of animals = 8490

No. of animals after pruning = 8433

... proportion (%) remaining = 99.3

No. of levels w/out records = 342

No. of levels with records = 8091 100.0%

... 1 record(s) = 7149 88.4%

... 2 record(s) = 942 11.6%

No. of animals w/out offspring = 6052 71.8%

No. of animals with offspring = 2381 28.2%

... and records = 2039 24.2%

No. of animals with unknown sire = 313

No. of animals with unknown dam = 384

No. of animals with both parents unknown = 312

No. of animals with records =

... and unknown sire = 1

... and unknown dam = 53

... and both parents unknown = 0

No. of sires = 325

... with progeny in the data = 320

... with records & progeny in data = 288

No. of dams = 2056

... with progeny in the data = 2056

... with records & progeny in data = 1751

No. of animals with known/unpruned grand-parents

... with paternal grandsire = 7358

... with paternal granddam = 7056

... with maternal grandsire = 6535

... with maternal granddam = 6468

random effect no. = 1 NRM

no. of elements in NRM/GIN inverse 31838

log determinant = -5688.7609053187680

random effect no. = 2 IDE

no. of elements in NRM/GIN inverse 0

log determinant = 0.0000000000000000

======== end of file ============================10-02-2015==========13:35====

======= Version 30-08-2013 ======================================= **KM** ====

Program WOMBAT: Summary of information from Set-up step

==============================================================================

Analysis type : "muv 2"

Data file : "DADOS.dat"

Pedigree file : "ReducedPedFile.dat"

Parameter file : "wombat.par"

No. of traits = 2

nrec mean sdev min. max.

1 "WS" 8078 299.703 49.6462 160.030 489.760

2 "BW0.75" 955 68.1510 10.1292 37.4020 94.5940

Numbers of individuals/records for pairs of traits

1 2

1 "WS" 8078 942

2 "BW0.75" 942 955

Covariables

1"WS" nrec mean sdev min. max.

1 "idv(2)" 8078 6.63568 3.07616 2.00000 18.0000

2 "idade(1)" 8078 473.414 105.028 293.000 645.000

2"BW0.75" nrec mean sdev min. max.

1 "idv(2)" 955 6.15079 3.05304 3.00000 17.0000

2 "idade(1)" 955 369.980 37.3543 267.000 511.000

Fixed effects

1 "WS" nlev

1 "gc" 201

2 "mn" 4

2 "BW0.75" nlev

1 "gc" 21

Random effects nlev

1 "animal" 8433 NRM

2 "peanim" 2107 IDE

======== end of file ============================10-02-2015==========13:35====

======= Version 30-08-2013 ======================================= **KM** ====

Program WOMBAT: Estimates of covariance components

==============================================================================

analise bi

Analysis type : "muv 2"

Data file : "DADOS.dat"

Pedigree file : "ReducedPedFile.dat"

Parameter file : "wombat.par"

No. of traits = 2 WS BW0.75

No. of records = 9033 8078 955

No. of parameters = 7

Maximum log L = -31666.468

-1/2 AIC & AICC = -31673.468 -31673.474

-1/2 BIC = -31698.257 "Penalty factor" = 4.541

Parameter estimates with approx. sampling erors

1 CHOL Z 1 1 20.6211 0.426549

2 CHOL Z 1 2 2.75180 0.153945

3 CHOL Z 2 2 2.46470 0.994284E-01

4 CHOL A 1 1 2.98737 0.380065E-01

5 CHOL A 1 2 3.31872 0.199860

6 CHOL A 2 2 -0.302575 0.400516

7 CHOL B 1 1 1.88572 0.809032E-01

Convergence criteria for last 3 iterates

Change in log likelihood = 0.241516 0.006203 0.000247

Change in parameter vector = 0.002855 0.000592 0.000165

Norm of gradient vector = 9.6190 1.7088 0.2796

Newton decrement = -0.4763 -0.0120 -0.0004

***** Estimates of residual covariances ************************************

Order of fit = 2

Covariance matrix

1 425.23

2 56.745 13.647

Eigenvalues of covariance matrix

Value 432.91 5.97

(%) 98.64 1.36

Trace 438.88

Matrix of correlations and variance ratios

1 0.4933

2 0.7449 0.5414

Covariances & correlations with approximate sampling errors

1 COVS Z 1 1 425.230 17.5918 vrat 0.493 0.026

2 COVS Z 1 2 56.7452 3.77981 corr 0.745 0.021

3 COVS Z 2 2 13.6472 1.05544 vrat 0.541 0.046

***** Estimates for RE 1 "animal" ***************************************

No. of levels = 8433

Covariance structure = NRM

Order of fit = 2

Covariance matrix

1 393.37

2 65.822 11.560

Eigenvalues of covariance matrix

Value 404.40 0.53

(%) 99.87 0.13

Trace 404.93

Matrix of correlations and variance ratios

1 0.4563

2 0.9761 0.4586

Covariances & correlations with approximate sampling errors

4 COVS A 1 1 393.367 29.9010 vrat 0.456 0.027

5 COVS A 1 2 65.8217 5.51748 corr 0.976 0.019

6 COVS A 2 2 11.5599 1.39111 vrat 0.459 0.046

***** Estimates for RE 2 "peanim" ***************************************

No. of levels = 2107

Covariance structure = IDE

Order of fit = 1

Covariance matrix

1 43.443

Matrix of correlations and variance ratios

1 0.0504

Covariances & correlations with approximate sampling errors

7 COVS B 1 1 43.4430 7.02936 vrat 0.050 0.008

***** Estimates of phenotypic covariances ***********************************

Covariance matrix

1 862.04

2 122.57 25.207

Eigenvalues of covariance matrix

Value 879.62 7.62

(%) 99.14 0.86

Trace 887.25

Correlation matrix

1 1.0000

2 0.8315 1.0000

Covariances & correlations with approximate sampling errors

8 COVS T 1 1 862.040 18.6605

9 COVS T 1 2 122.567 3.80421 corr 0.831 0.009

10 COVS T 2 2 25.2070 0.957047

======== end of file ============================10-02-2015==========13:35====

======= Version 30-08-2013 ======================================= **KM** ====

Program WOMBAT: Summary of Pedigree Information

==============================================================================

Two traits analysis (HH x BW0.75)

Analysis type : "muv 2"

Data file : "DADOS.dat"

Pedigree file : "ReducedPedFile.dat"

Parameter file : "wombat.par"

No. of animal IDs in data file = = 6560

No. of animal IDs in total = = 7100

*****Pedigree Structure for random effect : 1 ****************************

Original no. of animals = 7100

No. of animals after pruning = 7017

... proportion (%) remaining = 98.8

No. of levels w/out records = 457

No. of levels with records = 6560 100.0%

... 1 record(s) = 5617 85.6%

... 2 record(s) = 943 14.4%

No. of animals w/out offspring = 4829 68.8%

No. of animals with offspring = 2188 31.2%

... and records = 1731 24.7%

No. of animals with unknown sire = 148

No. of animals with unknown dam = 237

No. of animals with both parents unknown = 148

No. of animals with records =

... and unknown sire = 0

... and unknown dam = 18

... and both parents unknown = 0

No. of sires = 320

... with progeny in the data = 283

... with records & progeny in data = 240

No. of dams = 1868

... with progeny in the data = 1837

... with records & progeny in data = 1491

No. of animals with known/unpruned grand-parents

... with paternal grandsire = 6738

... with paternal granddam = 6484

... with maternal grandsire = 6339

... with maternal granddam = 6111

random effect no. = 1 NRM

no. of elements in NRM/GIN inverse 26822

log determinant = -4814.8039049931958

======== end of file ============================10-02-2015==========13:38====

======= Version 30-08-2013 ======================================= **KM** ====

Program WOMBAT: Summary of information from Set-up step

==============================================================================

Analysis type : "muv 2"

Data file : "DADOS.dat"

Pedigree file : "ReducedPedFile.dat"

Parameter file : "wombat.par"

No. of traits = 2

nrec mean sdev min. max.

1 "HH" 6548 132.269 5.43028 100.000 149.000

2 "BW0.75" 955 68.1510 10.1292 37.4020 94.5940

Numbers of individuals/records for pairs of traits

1 2

1 "HH" 6548 943

2 "BW0.75" 943 955

Covariables

1"HH" nrec mean sdev min. max.

1 "idv(2)" 6548 6.40043 2.93455 2.00000 17.0000

2 "idade(1)" 6548 472.701 107.333 293.000 645.000

2"BW0.75" nrec mean sdev min. max.

1 "idv(2)" 955 6.15079 3.05304 3.00000 17.0000

2 "idade(1)" 955 369.980 37.3543 267.000 511.000

Fixed effects

1 "HH" nlev

1 "gc" 169

2 "mn" 4

2 "BW0.75" nlev

1 "gc" 21

Random effects nlev

1 "animal" 7017 NRM

======== end of file ============================10-02-2015==========13:38====

======= Version 30-08-2013 ======================================= **KM** ====

Program WOMBAT: Estimates of covariance components

==============================================================================

Analysis type : "muv 2"

Data file : "DADOS.dat"

Pedigree file : "ReducedPedFile.dat"

Parameter file : "wombat.par"

No. of traits = 2 HH BW0.75

No. of records = 7503 6548 955

No. of parameters = 6

Maximum log L = -13201.269

-1/2 AIC & AICC = -13207.269 -13207.274

-1/2 BIC = -13227.957 "Penalty factor" = 4.448

Parameter estimates with approx. sampling erors

1 CHOL Z 1 1 3.97549 0.206654

2 CHOL Z 1 2 1.39910 0.127992

3 CHOL Z 2 2 1.99082 0.862562E-01

4 CHOL A 1 1 3.85409 0.288779

5 CHOL A 1 2 2.47683 0.178673

6 CHOL A 2 2 1.86777 0.194023

Convergence criteria for last 3 iterates

Change in log likelihood = 0.349336 0.005086 0.000111

Change in parameter vector = 0.015142 0.001746 0.000270

Norm of gradient vector = 9.9348 1.0661 0.1333

Newton decrement = -0.6405 -0.0089 -0.0002

***** Estimates of residual covariances ************************************

Order of fit = 2

Covariance matrix

1 5.9209

2 5.5621 15.805

Eigenvalues of covariance matrix

Value 18.30 3.42

(%) 84.25 15.75

Trace 21.73

Matrix of correlations and variance ratios

1 0.3809

2 0.5750 0.5155

Covariances & correlations with approximate sampling errors

1 COVS Z 1 1 5.92086 0.318504 vrat 0.381 0.026

2 COVS Z 1 2 5.56212 0.667553 corr 0.575 0.047

3 COVS Z 2 2 15.8045 1.64310 vrat 0.516 0.060

***** Estimates for RE 1 "animal" ***************************************

No. of levels = 7017

Covariance structure = NRM

Order of fit = 2

Covariance matrix

1 9.6233

2 9.5459 14.854

Eigenvalues of covariance matrix

Value 22.14 2.34

(%) 90.44 9.56

Trace 24.48

Matrix of correlations and variance ratios

1 0.6191

2 0.7984 0.4845

Covariances & correlations with approximate sampling errors

4 COVS A 1 1 9.62326 0.595950 vrat 0.619 0.026

5 COVS A 1 2 9.54593 1.00602 corr 0.798 0.048

6 COVS A 2 2 14.8540 2.22596 vrat 0.484 0.060

***** Estimates of phenotypic covariances ***********************************

Covariance matrix

1 15.544

2 15.108 30.658

Eigenvalues of covariance matrix

Value 39.99 6.21

(%) 86.56 13.44

Trace 46.20

Correlation matrix

1 1.0000

2 0.6921 1.0000

Covariances & correlations with approximate sampling errors

7 COVS T 1 1 15.5441 0.385953

8 COVS T 1 2 15.1080 0.687916 corr 0.692 0.018

9 COVS T 2 2 30.6585 1.44541

======== end of file ============================10-02-2015==========13:38====

======= Version 30-08-2013 ======================================= **KM** ====

Program WOMBAT: Summary of Pedigree Information

==============================================================================

Two traits analysis (CC x BW0.75)

Analysis type : "muv 2"

Data file : "DADOS.dat"

Pedigree file : "ReducedPedFile.dat"

Parameter file : "wombat.par"

No. of animal IDs in data file = = 3943

No. of animal IDs in total = = 5418

*****Pedigree Structure for random effect : 1 ****************************

Original no. of animals = 5418

No. of animals after pruning = 5330

... proportion (%) remaining = 98.4

No. of levels w/out records = 1387

No. of levels with records = 3943 100.0%

... 1 record(s) = 3055 77.5%

... 2 record(s) = 888 22.5%

No. of animals w/out offspring = 3311 62.1%

No. of animals with offspring = 2019 37.9%

... and records = 632 11.9%

No. of animals with unknown sire = 94

No. of animals with unknown dam = 188

No. of animals with both parents unknown = 94

No. of animals with records =

... and unknown sire = 0

... and unknown dam = 0

... and both parents unknown = 0

No. of sires = 318

... with progeny in the data = 255

... with records & progeny in data = 217

No. of dams = 1701

... with progeny in the data = 1508

... with records & progeny in data = 412

No. of animals with known/unpruned grand-parents

... with paternal grandsire = 5142

... with paternal granddam = 4954

... with maternal grandsire = 4953

... with maternal granddam = 4738

random effect no. = 1 NRM

no. of elements in NRM/GIN inverse 20464

log determinant = -3671.8274443710275

======== end of file ============================10-02-2015==========13:42====

======= Version 30-08-2013 ======================================= **KM** ====

Program WOMBAT: Summary of information from Set-up step

==============================================================================

Analysis type : "muv 2"

Data file : "DADOS.dat"

Pedigree file : "ReducedPedFile.dat"

Parameter file : "wombat.par"

No. of traits = 2

nrec mean sdev min. max.

1 "CC" 3876 164.140 8.72028 128.000 192.000

2 "BW0.75" 955 68.1510 10.1292 37.4020 94.5940

Numbers of individuals/records for pairs of traits

1 2

1 "CC" 3876 888

2 "BW0.75" 888 955

Covariables

1"CC" nrec mean sdev min. max.

1 "idv(2)" 3876 6.27632 2.87660 2.00000 17.0000

2 "idade(1)" 3876 431.403 109.187 293.000 725.000

2"BW0.75" nrec mean sdev min. max.

1 "idv(2)" 955 6.15079 3.05304 3.00000 17.0000

2 "idade(1)" 955 369.980 37.3543 267.000 511.000

Fixed effects

1 "CC" nlev

1 "gc" 100

2 "mn" 4

2 "BW0.75" nlev

1 "gc" 21

Random effects nlev

1 "animal" 5330 NRM

======== end of file ============================10-02-2015==========13:42====

======= Version 30-08-2013 ======================================= **KM** ====

Program WOMBAT: Estimates of covariance components

==============================================================================

Analysis type : "muv 2"

Data file : "DADOS.dat"

Pedigree file : "ReducedPedFile.dat"

Parameter file : "wombat.par"

No. of traits = 2 CC BW0.75

No. of records = 4831 3876 955

No. of parameters = 6

Maximum log L = -10425.771

-1/2 AIC & AICC = -10431.771 -10431.779

-1/2 BIC = -10451.137 "Penalty factor" = 4.228

Parameter estimates with approx. sampling erors

1 CHOL Z 1 1 3.77531 0.208524

2 CHOL Z 1 2 3.31027 0.209626

3 CHOL Z 2 2 3.70101 0.128233

4 CHOL A 1 1 4.06780 0.278033

5 CHOL A 1 2 3.12160 0.261803

6 CHOL A 2 2 1.53396 0.269572

Convergence criteria for last 3 iterates

Change in log likelihood = 9.268096 0.027944 0.000129

Change in parameter vector = 0.067125 0.004697 0.000510

Norm of gradient vector = 55.3817 2.1562 0.0814

Newton decrement = -19.0285 -0.0553 -0.0002

***** Estimates of residual covariances ************************************

Order of fit = 2

Covariance matrix

1 24.655

2 12.497 14.253

Eigenvalues of covariance matrix

Value 32.99 5.92

(%) 84.79 15.21

Trace 38.91

Matrix of correlations and variance ratios

1 0.6708

2 0.6667 0.4628

Covariances & correlations with approximate sampling errors

1 COVS Z 1 1 24.6554 1.17454 vrat 0.671 0.038

2 COVS Z 1 2 12.4973 1.19290 corr 0.667 0.032

3 COVS Z 2 2 14.2530 1.57449 vrat 0.463 0.058

***** Estimates for RE 1 "animal" ***************************************

No. of levels = 5330

Covariance structure = NRM

Order of fit = 2

Covariance matrix

1 12.097

2 12.698 16.547

Eigenvalues of covariance matrix

Value 27.21 1.43

(%) 95.01 4.99

Trace 28.64

Matrix of correlations and variance ratios

1 0.3292

2 0.8975 0.5372

Covariances & correlations with approximate sampling errors

4 COVS A 1 1 12.0974 1.58657 vrat 0.329 0.038

5 COVS A 1 2 12.6980 1.63018 corr 0.897 0.038

6 COVS A 2 2 16.5470 2.26196 vrat 0.537 0.058

***** Estimates of phenotypic covariances ***********************************

Covariance matrix

1 36.753

2 25.195 30.800

Eigenvalues of covariance matrix

Value 59.15 8.41

(%) 87.56 12.44

Trace 67.55

Correlation matrix

1 1.0000

2 0.7489 1.0000

Covariances & correlations with approximate sampling errors

7 COVS T 1 1 36.7528 0.999648

8 COVS T 1 2 25.1953 1.07804 corr 0.749 0.014

9 COVS T 2 2 30.8000 1.44670

======== end of file ============================10-02-2015==========13:42====

======= Version 30-08-2013 ======================================= **KM** ====

Program WOMBAT: Summary of Pedigree Information

==============================================================================

Two traits analysis (LEA x BW0.75)

Analysis type : "muv 2"

Data file : "DADOS.dat"

Pedigree file : "ReducedPedFile.dat"

Parameter file : "wombat.par"

No. of animal IDs in data file = = 2396

No. of animal IDs in total = = 3756

*****Pedigree Structure for random effect : 1 ****************************

Original no. of animals = 3756

No. of animals after pruning = 3665

... proportion (%) remaining = 97.6

No. of levels w/out records = 1269

No. of levels with records = 2396 100.0%

... 1 record(s) = 1554 64.9%

... 2 record(s) = 842 35.1%

No. of animals w/out offspring = 1913 52.2%

No. of animals with offspring = 1752 47.8%

... and records = 483 13.2%

No. of animals with unknown sire = 64

No. of animals with unknown dam = 161

No. of animals with both parents unknown = 64

No. of animals with records =

... and unknown sire = 0

... and unknown dam = 0

... and both parents unknown = 0

No. of sires = 313

... with progeny in the data = 180

... with records & progeny in data = 85

No. of dams = 1439

... with progeny in the data = 1042

... with records & progeny in data = 392

No. of animals with known/unpruned grand-parents

... with paternal grandsire = 3520

... with paternal granddam = 3382

... with maternal grandsire = 3391

... with maternal granddam = 3267

random effect no. = 1 NRM

no. of elements in NRM/GIN inverse 14038

log determinant = -2522.5521618264124

======== end of file ============================10-02-2015==========13:45====

======= Version 30-08-2013 ======================================= **KM** ====

Program WOMBAT: Summary of information from Set-up step

==============================================================================

Analysis type : "muv 2"

Data file : "DADOS.dat"

Pedigree file : "ReducedPedFile.dat"

Parameter file : "wombat.par"

No. of traits = 2

nrec mean sdev min. max.

1 "LEA" 2283 51.4305 8.89390 21.4000 83.4000

2 "BW0.75" 955 68.1510 10.1292 37.4020 94.5940

Numbers of individuals/records for pairs of traits

1 2

1 "LEA" 2283 842

2 "BW0.75" 842 955

Covariables

1"LEA" nrec mean sdev min. max.

1 "idv(2)" 2283 6.11082 2.92852 2.00000 17.0000

2 "idade(1)" 2283 448.951 98.8881 306.000 609.000

2"BW0.75" nrec mean sdev min. max.

1 "idv(2)" 955 6.15079 3.05304 3.00000 17.0000

2 "idade(1)" 955 369.980 37.3543 267.000 511.000

Fixed effects

1 "LEA" nlev

1 "gc" 65

2 "mn" 4

2 "BW0.75" nlev

1 "gc" 21

Random effects nlev

1 "animal" 3665 NRM

======== end of file ============================10-02-2015==========13:45====

======= Version 30-08-2013 ======================================= **KM** ====

Program WOMBAT: Estimates of covariance components

==============================================================================

Analysis type : "muv 2"

Data file : "DADOS.dat"

Pedigree file : "ReducedPedFile.dat"

Parameter file : "wombat.par"

No. of traits = 2 LEA BW0.75

No. of records = 3238 2283 955

No. of parameters = 6

Maximum log L = -6971.610

-1/2 AIC & AICC = -6977.610 -6977.623

-1/2 BIC = -6995.769 "Penalty factor" = 4.026

Parameter estimates with approx. sampling erors

1 CHOL Z 1 1 4.47644 0.172087

2 CHOL Z 1 2 1.99846 0.281480

3 CHOL Z 2 2 3.05466 0.235291

4 CHOL A 1 1 4.30028 0.324588

5 CHOL A 1 2 2.62078 0.390877

6 CHOL A 2 2 3.15971 0.281710

Convergence criteria for last 3 iterates

Change in log likelihood = 5.110887 0.080301 0.000276

Change in parameter vector = 0.111287 0.012495 0.001084

Norm of gradient vector = 20.5020 3.3025 0.0731

Newton decrement = -10.0772 -0.1666 -0.0006

***** Estimates of residual covariances ************************************

Order of fit = 2

Covariance matrix

1 20.039

2 8.9460 13.325

Eigenvalues of covariance matrix

Value 26.24 7.13

(%) 78.64 21.36

Trace 33.36

Matrix of correlations and variance ratios

1 0.5432

2 0.5475 0.4188

Covariances & correlations with approximate sampling errors

1 COVS Z 1 1 20.0386 1.54067 vrat 0.543 0.050

2 COVS Z 1 2 8.94600 1.42493 corr 0.547 0.060

3 COVS Z 2 2 13.3248 1.86493 vrat 0.419 0.067

***** Estimates for RE 1 "animal" ***************************************

No. of levels = 3665

Covariance structure = NRM

Order of fit = 2

Covariance matrix

1 16.852

2 11.270 18.492

Eigenvalues of covariance matrix

Value 28.97 6.37

(%) 81.97 18.03

Trace 35.34

Matrix of correlations and variance ratios

1 0.4568

2 0.6384 0.5812

Covariances & correlations with approximate sampling errors

4 COVS A 1 1 16.8522 2.26763 vrat 0.457 0.050

5 COVS A 1 2 11.2701 2.07844 corr 0.638 0.074

6 COVS A 2 2 18.4924 2.79163 vrat 0.581 0.067

***** Estimates of phenotypic covariances ***********************************

Covariance matrix

1 36.891

2 20.216 31.817

Eigenvalues of covariance matrix

Value 54.73 13.98

(%) 79.65 20.35

Trace 68.71

Correlation matrix

1 1.0000

2 0.5901 1.0000

Covariances & correlations with approximate sampling errors

7 COVS T 1 1 36.8908 1.36341

8 COVS T 1 2 20.2161 1.31262 corr 0.590 0.025

9 COVS T 2 2 31.8172 1.69100

======== end of file ============================10-02-2015==========13:45====

======= Version 30-08-2013 ======================================= **KM** ====

Program WOMBAT: Summary of Pedigree Information

==============================================================================

Two traits analysis (BF x BW0.75)

Analysis type : "muv 2"

Data file : "DADOS.dat"

Pedigree file : "ReducedPedFile.dat"

Parameter file : "wombat.par"

No. of animal IDs in data file = = 2396

No. of animal IDs in total = = 3756

*****Pedigree Structure for random effect : 1 ****************************

Original no. of animals = 3756

No. of animals after pruning = 3665

... proportion (%) remaining = 97.6

No. of levels w/out records = 1269

No. of levels with records = 2396 100.0%

... 1 record(s) = 1552 64.8%

... 2 record(s) = 844 35.2%

No. of animals w/out offspring = 1913 52.2%

No. of animals with offspring = 1752 47.8%

... and records = 483 13.2%

No. of animals with unknown sire = 64

No. of animals with unknown dam = 161

No. of animals with both parents unknown = 64

No. of animals with records =

... and unknown sire = 0

... and unknown dam = 0

... and both parents unknown = 0

No. of sires = 313

... with progeny in the data = 180

... with records & progeny in data = 85

No. of dams = 1439

... with progeny in the data = 1042

... with records & progeny in data = 392

No. of animals with known/unpruned grand-parents

... with paternal grandsire = 3520

... with paternal granddam = 3382

... with maternal grandsire = 3391

... with maternal granddam = 3267

random effect no. = 1 NRM

no. of elements in NRM/GIN inverse 14038

log determinant = -2522.5521618264124

======== end of file ============================10-02-2015==========13:46====

======= Version 30-08-2013 ======================================= **KM** ====

Program WOMBAT: Summary of information from Set-up step

==============================================================================

Analysis type : "muv 2"

Data file : "DADOS.dat"

Pedigree file : "ReducedPedFile.dat"

Parameter file : "wombat.par"

No. of traits = 2

nrec mean sdev min. max.

1 "BF" 2285 1.75922 1.42798 0.00000 10.5000

2 "BW0.75" 955 68.1510 10.1292 37.4020 94.5940

Numbers of individuals/records for pairs of traits

1 2

1 "BF" 2285 844

2 "BW0.75" 844 955

Covariables

1"BF" nrec mean sdev min. max.

1 "idv(2)" 2285 6.10810 2.92868 2.00000 17.0000

2 "idade(1)" 2285 449.081 98.9421 306.000 609.000

2"BW0.75" nrec mean sdev min. max.

1 "idv(2)" 955 6.15079 3.05304 3.00000 17.0000

2 "idade(1)" 955 369.980 37.3543 267.000 511.000

Fixed effects

1 "BF" nlev

1 "gc" 65

2 "mn" 4

2 "BW0.75" nlev

1 "gc" 21

Random effects nlev

1 "animal" 3665 NRM

======== end of file ============================10-02-2015==========13:46====

======= Version 30-08-2013 ======================================= **KM** ====

Program WOMBAT: Estimates of covariance components

==============================================================================

Analysis type : "muv 2"

Data file : "DADOS.dat"

Pedigree file : "ReducedPedFile.dat"

Parameter file : "wombat.par"

No. of traits = 2 BF BW0.75

No. of records = 3240 2285 955

No. of parameters = 6

Maximum log L = -3074.715

-1/2 AIC & AICC = -3080.715 -3080.728

-1/2 BIC = -3098.876 "Penalty factor" = 4.027

Parameter estimates with approx. sampling erors

1 CHOL Z 1 1 3.56218 0.286842

2 CHOL Z 1 2 0.356362 0.623812E-01

3 CHOL Z 2 2 0.718051 0.349454E-01

4 CHOL A 1 1 1.46756 0.820281E-01

5 CHOL A 1 2 0.739714E-01 0.677294E-01

6 CHOL A 2 2 -0.646954 0.847179E-01

Convergence criteria for last 3 iterates

Change in log likelihood = 14.959532 0.189604 0.000213

Change in parameter vector = 0.076830 0.010871 0.000350

Norm of gradient vector = 259.8693 30.2450 0.5614

Newton decrement = -29.0586 -0.3860 -0.0004

***** Estimates of residual covariances ************************************

Order of fit = 2

Covariance matrix

1 0.64259

2 1.2694 12.689

Eigenvalues of covariance matrix

Value 12.82 0.51

(%) 96.17 3.83

Trace 13.33

Matrix of correlations and variance ratios

1 0.6968

2 0.4446 0.4027

Covariances & correlations with approximate sampling errors

1 COVS Z 1 1 0.642591 0.376914E-01 vrat 0.697 0.046

2 COVS Z 1 2 1.26943 0.229530 corr 0.445 0.075

3 COVS Z 2 2 12.6891 2.04357 vrat 0.403 0.075

***** Estimates for RE 1 "animal" ***************************************

No. of levels = 3665

Covariance structure = NRM

Order of fit = 2

Covariance matrix

1 0.27967

2 0.32093 18.824

Eigenvalues of covariance matrix

Value 18.83 0.27

(%) 98.57 1.43

Trace 19.10

Matrix of correlations and variance ratios

1 0.3032

2 0.1399 0.5973

Covariances & correlations with approximate sampling errors

4 COVS A 1 1 0.279669 0.477153E-01 vrat 0.303 0.046

5 COVS A 1 2 0.320934 0.298470 corr 0.140 0.126

6 COVS A 2 2 18.8237 3.08814 vrat 0.597 0.075

***** Estimates of phenotypic covariances ***********************************

Covariance matrix

1 0.92226

2 1.5904 31.513

Eigenvalues of covariance matrix

Value 31.60 0.84

(%) 97.41 2.59

Trace 32.44

Correlation matrix

1 1.0000

2 0.2950 1.0000

Covariances & correlations with approximate sampling errors

7 COVS T 1 1 0.922260 0.313464E-01

8 COVS T 1 2 1.59036 0.196727 corr 0.295 0.034

9 COVS T 2 2 31.5128 1.76483

======== end of file ============================10-02-2015==========13:46====

======= Version 30-08-2013 ======================================= **KM** ====

Program WOMBAT: Summary of Pedigree Information

==============================================================================

Two traits analysis (RF x BW0.75)

Analysis type : "muv 2"

Data file : "DADOS.dat"

Pedigree file : "ReducedPedFile.dat"

Parameter file : "wombat.par"

No. of animal IDs in data file = = 1930

No. of animal IDs in total = = 3174

*****Pedigree Structure for random effect : 1 ****************************

Original no. of animals = 3174

No. of animals after pruning = 3089

... proportion (%) remaining = 97.3

No. of levels w/out records = 1159

No. of levels with records = 1930 100.0%

... 1 record(s) = 1088 56.4%

... 2 record(s) = 842 43.6%

No. of animals w/out offspring = 1497 48.5%

No. of animals with offspring = 1592 51.5%

... and records = 433 14.0%

No. of animals with unknown sire = 60

No. of animals with unknown dam = 147

No. of animals with both parents unknown = 59

No. of animals with records =

... and unknown sire = 0

... and unknown dam = 0

... and both parents unknown = 0

No. of sires = 307

... with progeny in the data = 114

... with records & progeny in data = 44

No. of dams = 1285

... with progeny in the data = 792

... with records & progeny in data = 389

No. of animals with known/unpruned grand-parents

... with paternal grandsire = 2965

... with paternal granddam = 2839

... with maternal grandsire = 2840

... with maternal granddam = 2751

random effect no. = 1 NRM

no. of elements in NRM/GIN inverse 11801

log determinant = -2124.3928089367182

======== end of file ============================10-02-2015==========13:47====

======= Version 30-08-2013 ======================================= **KM** ====

Program WOMBAT: Summary of information from Set-up step

==============================================================================

Analysis type : "muv 2"

Data file : "DADOS.dat"

Pedigree file : "ReducedPedFile.dat"

Parameter file : "wombat.par"

No. of traits = 2

nrec mean sdev min. max.

1 "RF" 1817 5.08663 2.54341 0.00000 19.2000

2 "BW0.75" 955 68.1510 10.1292 37.4020 94.5940

Numbers of individuals/records for pairs of traits

1 2

1 "RF" 1817 842

2 "BW0.75" 842 955

Covariables

1"RF" nrec mean sdev min. max.

1 "idv(2)" 1817 6.09191 3.03138 2.00000 17.0000

2 "idade(1)" 1817 469.100 100.703 306.000 609.000

2"BW0.75" nrec mean sdev min. max.

1 "idv(2)" 955 6.15079 3.05304 3.00000 17.0000

2 "idade(1)" 955 369.980 37.3543 267.000 511.000

Fixed effects

1 "RF" nlev

1 "gc" 51

2 "mn" 3

2 "BW0.75" nlev

1 "gc" 21

Random effects nlev

1 "animal" 3089 NRM

======== end of file ============================10-02-2015==========13:47====

======= Version 30-08-2013 ======================================= **KM** ====

Program WOMBAT: Estimates of covariance components

==============================================================================

Analysis type : "muv 2"

Data file : "DADOS.dat"

Pedigree file : "ReducedPedFile.dat"

Parameter file : "wombat.par"

No. of traits = 2 RF BW0.75

No. of records = 2772 1817 955

No. of parameters = 6

Maximum log L = -3757.175

-1/2 AIC & AICC = -3763.175 -3763.191

-1/2 BIC = -3780.869 "Penalty factor" = 3.949

Parameter estimates with approx. sampling erors

1 CHOL Z 1 1 3.48409 0.298160

2 CHOL Z 1 2 0.387192 0.109970

3 CHOL Z 2 2 1.20929 0.512505E-01

4 CHOL A 1 1 4.45452 0.358511

5 CHOL A 1 2 0.301677 0.118414

6 CHOL A 2 2 0.949592 0.810834E-01

Convergence criteria for last 3 iterates

Change in log likelihood = 13.595896 0.140865 0.000065

Change in parameter vector = 0.043740 0.009667 0.000256

Norm of gradient vector = 172.7792 9.7028 0.1035

Newton decrement = -25.8714 -0.2802 -0.0001

***** Estimates of residual covariances ************************************

Order of fit = 2

Covariance matrix

1 1.6123

2 1.3490 12.139

Eigenvalues of covariance matrix

Value 12.31 1.44

(%) 89.51 10.49

Trace 13.75

Matrix of correlations and variance ratios

1 0.6189

2 0.3049 0.3796

Covariances & correlations with approximate sampling errors

1 COVS Z 1 1 1.61231 0.116955 vrat 0.619 0.052

2 COVS Z 1 2 1.34901 0.395966 corr 0.305 0.084

3 COVS Z 2 2 12.1389 2.07763 vrat 0.380 0.075

***** Estimates for RE 1 "animal" ***************************************

No. of levels = 3089

Covariance structure = NRM

Order of fit = 2

Covariance matrix

1 0.99273

2 1.3438 19.843

Eigenvalues of covariance matrix

Value 19.94 0.90

(%) 95.69 4.31

Trace 20.84

Matrix of correlations and variance ratios

1 0.3811

2 0.3028 0.6204

Covariances & correlations with approximate sampling errors

4 COVS A 1 1 0.992734 0.160582 vrat 0.381 0.052

5 COVS A 1 2 1.34383 0.545628 corr 0.303 0.114

6 COVS A 2 2 19.8428 3.19399 vrat 0.620 0.075

***** Estimates of phenotypic covariances ***********************************

Covariance matrix

1 2.6050

2 2.6928 31.982

Eigenvalues of covariance matrix

Value 32.23 2.36

(%) 93.18 6.82

Trace 34.59

Correlation matrix

1 1.0000

2 0.2950 1.0000

Covariances & correlations with approximate sampling errors

7 COVS T 1 1 2.60504 0.102876

8 COVS T 1 2 2.69284 0.351619 corr 0.295 0.035

9 COVS T 2 2 31.9816 1.81750

======== end of file ============================10-02-2015==========13:47====

======= Version 30-08-2013 ======================================= **KM** ====

Program WOMBAT: Summary of Pedigree Information

==============================================================================

Two traits analysis (WS x HH)

Analysis type : "muv 2"

Data file : "DADOS.dat"

Pedigree file : "ReducedPedFile.dat"

Parameter file : "wombat.par"

No. of animal IDs in data file = = 8109

No. of animal IDs in total = = 8506

*****Pedigree Structure for random effect : 1 ****************************

Original no. of animals = 8506

No. of animals after pruning = 8449

... proportion (%) remaining = 99.3

No. of levels w/out records = 340

No. of levels with records = 8109 100.0%

... 1 record(s) = 1592 19.6%

... 2 record(s) = 6517 80.4%

No. of animals w/out offspring = 6069 71.8%

No. of animals with offspring = 2380 28.2%

... and records = 2040 24.1%

No. of animals with unknown sire = 313

No. of animals with unknown dam = 384

No. of animals with both parents unknown = 312

No. of animals with records =

... and unknown sire = 1

... and unknown dam = 53

... and both parents unknown = 0

No. of sires = 325

... with progeny in the data = 320

... with records & progeny in data = 288

No. of dams = 2055

... with progeny in the data = 2055

... with records & progeny in data = 1752

No. of animals with known/unpruned grand-parents

... with paternal grandsire = 7374

... with paternal granddam = 7072

... with maternal grandsire = 6550

... with maternal granddam = 6483

Inbreeding coefficients for random effect 1 computed

No. of inbred animals = 5154

Average inbreeding coefficient = 1.5406 (in %)

... amongst inbred animals = 2.5255 (in %)

random effect no. = 1 NRM

no. of elements in NRM/GIN inverse 31902

log determinant = -5699.9648087765054

random effect no. = 2 IDE

no. of elements in NRM/GIN inverse 0

log determinant = 0.0000000000000000

======== end of file ============================10-02-2015==========13:54====

======= Version 30-08-2013 ======================================= **KM** ====

Program WOMBAT: Summary of information from Set-up step

==============================================================================

Analysis type : "muv 2"

Data file : "DADOS.dat"

Pedigree file : "ReducedPedFile.dat"

Parameter file : "wombat.par"

No. of traits = 2

nrec mean sdev min. max.

1 "WS" 8078 299.703 49.6462 160.030 489.760

2 "HH" 6548 132.269 5.43028 100.000 149.000

Numbers of individuals/records for pairs of traits

1 2

1 "WS" 8078 6517

2 "HH" 6517 6548

Covariables

1"WS" nrec mean sdev min. max.

1 "idv(2)" 8078 6.63568 3.07616 2.00000 18.0000

2 "idade(1)" 8078 473.414 105.028 293.000 645.000

2"HH" nrec mean sdev min. max.

1 "idv(2)" 6548 6.40043 2.93455 2.00000 17.0000

2 "idade(1)" 6548 472.701 107.333 293.000 645.000

Fixed effects

1 "WS" nlev

1 "gc" 201

2 "mn" 4

2 "HH" nlev

1 "gc" 169

2 "mn" 4

Random effects nlev

1 "animal" 8449 NRM

2 "peanim" 2107 IDE

======== end of file ============================10-02-2015==========13:54====

======= Version 30-08-2013 ======================================= **KM** ====

Program WOMBAT: Estimates of covariance components

==============================================================================

Analysis type : "muv 2"

Data file : "DADOS.dat"

Pedigree file : "ReducedPedFile.dat"

Parameter file : "wombat.par"

No. of traits = 2 WS HH

No. of records = 14626 8078 6548

No. of parameters = 7

Maximum log L = -39787.326

-1/2 AIC & AICC = -39794.326 -39794.330

-1/2 BIC = -39820.800 "Penalty factor" = 4.782

Parameter estimates with approx. sampling erors

1 CHOL Z 1 1 20.8950 0.428779

2 CHOL Z 1 2 1.55462 0.739183E-01

3 CHOL Z 2 2 1.90091 0.467090E-01

4 CHOL A 1 1 19.9683 0.760366

5 CHOL A 1 2 2.26411 0.122015

6 CHOL A 2 2 1.99579 0.802012E-01

7 CHOL B 1 1 1.79977 0.758415E-01

Convergence criteria for last 3 iterates

Change in log likelihood = 0.017730 0.000744 0.000035

Change in parameter vector = 0.003069 0.000743 0.000175

Norm of gradient vector = 2.3679 0.3846 0.0674

Newton decrement = -0.0297 -0.0012 -0.0001

***** Estimates of residual covariances ************************************

Order of fit = 2

Covariance matrix

1 436.60

2 32.484 6.0303

Eigenvalues of covariance matrix

Value 439.04 3.59

(%) 99.19 0.81

Trace 442.63

Matrix of correlations and variance ratios

1 0.5007

2 0.6331 0.3983

Covariances & correlations with approximate sampling errors

1 COVS Z 1 1 436.601 17.9187 vrat 0.501 0.026

2 COVS Z 1 2 32.4838 1.99294 corr 0.633 0.019

3 COVS Z 2 2 6.03030 0.307653 vrat 0.398 0.026

***** Estimates for RE 1 "animal" ***************************************

No. of levels = 8449

Covariance structure = NRM

Order of fit = 2

Covariance matrix

1 398.73

2 45.210 9.1094

Eigenvalues of covariance matrix

Value 403.91 3.93

(%) 99.04 0.96

Trace 407.84

Matrix of correlations and variance ratios

1 0.4573

2 0.7502 0.6017

Covariances & correlations with approximate sampling errors

4 COVS A 1 1 398.733 30.3664 vrat 0.457 0.027

5 COVS A 1 2 45.2104 3.52956 corr 0.750 0.025

6 COVS A 2 2 9.10935 0.565954 vrat 0.602 0.026

***** Estimates for RE 2 "peanim" ***************************************

No. of levels = 2107

Covariance structure = IDE

Order of fit = 1

Covariance matrix

1 36.582

Matrix of correlations and variance ratios

1 0.0420

Covariances & correlations with approximate sampling errors

7 COVS B 1 1 36.5815 5.54879 vrat 0.042 0.006

***** Estimates of phenotypic covariances ***********************************

Covariance matrix

1 871.92

2 77.694 15.140

Eigenvalues of covariance matrix

Value 878.90 8.15

(%) 99.08 0.92

Trace 887.06

Correlation matrix

1 1.0000

2 0.6762 1.0000

Covariances & correlations with approximate sampling errors

8 COVS T 1 1 871.916 18.9569

9 COVS T 1 2 77.6943 2.25490 corr 0.676 0.009

10 COVS T 2 2 15.1397 0.365149

======== end of file ============================10-02-2015==========13:54====

======= Version 30-08-2013 ======================================= **KM** ====

Program WOMBAT: Summary of Pedigree Information

==============================================================================

Two traits analysis (WS x CC)

Analysis type : "muv 2"

Data file : "DADOS.dat"

Pedigree file : "ReducedPedFile.dat"

Parameter file : "wombat.par"

No. of animal IDs in data file = = 8090

No. of animal IDs in total = = 8490

*****Pedigree Structure for random effect : 1 ****************************

Original no. of animals = 8490

No. of animals after pruning = 8433

... proportion (%) remaining = 99.3

No. of levels w/out records = 343

No. of levels with records = 8090 100.0%

... 1 record(s) = 4226 52.2%

... 2 record(s) = 3864 47.8%

No. of animals w/out offspring = 6052 71.8%

No. of animals with offspring = 2381 28.2%

... and records = 2038 24.2%

No. of animals with unknown sire = 313

No. of animals with unknown dam = 384

No. of animals with both parents unknown = 312

No. of animals with records =

... and unknown sire = 1

... and unknown dam = 53

... and both parents unknown = 0

No. of sires = 325

... with progeny in the data = 320

... with records & progeny in data = 288

No. of dams = 2056

... with progeny in the data = 2056

... with records & progeny in data = 1750

No. of animals with known/unpruned grand-parents

... with paternal grandsire = 7358

... with paternal granddam = 7056

... with maternal grandsire = 6535

... with maternal granddam = 6468

random effect no. = 1 NRM

no. of elements in NRM/GIN inverse 31838

log determinant = -5688.7609053187680

random effect no. = 2 IDE

no. of elements in NRM/GIN inverse 0

log determinant = 0.0000000000000000

======== end of file ============================10-02-2015==========14:21====

======= Version 30-08-2013 ======================================= **KM** ====

Program WOMBAT: Summary of information from Set-up step

==============================================================================

Analysis type : "muv 2"

Data file : "DADOS.dat"

Pedigree file : "ReducedPedFile.dat"

Parameter file : "wombat.par"

No. of traits = 2

nrec mean sdev min. max.

1 "WS" 8078 299.703 49.6462 160.030 489.760

2 "CC" 3876 164.140 8.72028 128.000 192.000

Numbers of individuals/records for pairs of traits

1 2

1 "WC" 8078 3864

2 "CC" 3864 3876

Covariables

1"WC" nrec mean sdev min. max.

1 "idv(2)" 8078 6.63568 3.07616 2.00000 18.0000

2 "idade(1)" 8078 473.414 105.028 293.000 645.000

2"CC" nrec mean sdev min. max.

1 "idv(2)" 3876 6.27632 2.87660 2.00000 17.0000

2 "idade(1)" 3876 431.403 109.187 293.000 725.000

Fixed effects

1 "WS" nlev

1 "gc" 201

2 "mn" 4

2 "CC" nlev

1 "gc" 100

2 "mn" 4

Random effects nlev

1 "animal" 8433 NRM

2 "peanim" 2107 IDE

======== end of file ============================10-02-2015==========14:21====

======= Version 30-08-2013 ======================================= **KM** ====

Program WOMBAT: Estimates of covariance components

==============================================================================

Analysis type : "muv 2"

Data file : "DADOS.dat"

Pedigree file : "ReducedPedFile.dat"

Parameter file : "wombat.par"

No. of traits = 2 WS CC

No. of records = 11954 8078 3876

No. of parameters = 7

Maximum log L = -37576.927

-1/2 AIC & AICC = -37583.927 -37583.932

-1/2 BIC = -37609.695 "Penalty factor" = 4.681

Parameter estimates with approx. sampling erors

1 CHOL Z 1 1 24.0196 0.330365

2 CHOL Z 1 2 3.76196 0.981907E-01

3 CHOL Z 2 2 3.69745 0.589445E-01

4 CHOL A 1 1 12.4502 0.797577

5 CHOL A 1 2 1.92263 0.202897

6 CHOL A 2 2 0.978126 0.177282

7 CHOL B 1 1 2.04259 0.526928E-01

Convergence criteria for last 3 iterates

Change in log likelihood = 141.637968 4.337866 0.070692

Change in parameter vector = 0.121204 0.044367 0.008226

Norm of gradient vector = 165.0700 12.2203 3.4727

Newton decrement = -263.7216 -7.9919 -0.1218

*** "WOMBAT" : Full convergence has not been achieved

Treat results with caution !!!

***** Estimates of residual covariances ************************************

Order of fit = 2

Covariance matrix

1 576.94

2 90.361 27.823

Eigenvalues of covariance matrix

Value 591.43 13.34

(%) 97.79 2.21

Trace 604.76

Matrix of correlations and variance ratios

1 0.7290

2 0.7132 0.8567

Covariances & correlations with approximate sampling errors

1 COVS Z 1 1 576.942 15.8705 vrat 0.729 0.023

2 COVS Z 1 2 90.3608 3.14853 corr 0.713 0.011

3 COVS Z 2 2 27.8235 0.840128 vrat 0.857 0.025

***** Estimates for RE 1 "animal" ***************************************

No. of levels = 8433

Covariance structure = NRM

Order of fit = 2

Covariance matrix

1 155.01

2 23.937 4.6532

Eigenvalues of covariance matrix

Value 158.73 0.93

(%) 99.41 0.59

Trace 159.66

Matrix of correlations and variance ratios

1 0.1959

2 0.8913 0.1433

Covariances & correlations with approximate sampling errors

4 COVS A 1 1 155.007 19.8599 vrat 0.196 0.023

5 COVS A 1 2 23.9371 3.63005 corr 0.891 0.038

6 COVS A 2 2 4.65324 0.852754 vrat 0.143 0.025

***** Estimates for RE 2 "peanim" ***************************************

No. of levels = 2107

Covariance structure = IDE

Order of fit = 1

Covariance matrix

1 59.453

Matrix of correlations and variance ratios

1 0.0751

Covariances & correlations with approximate sampling errors

7 COVS B 1 1 59.4531 6.26550 vrat 0.075 0.008

***** Estimates of phenotypic covariances ***********************************

Covariance matrix

1 791.40

2 114.30 32.477

Eigenvalues of covariance matrix

Value 808.24 15.64

(%) 98.10 1.90

Trace 823.88

Correlation matrix

1 1.0000

2 0.7129 1.0000

Covariances & correlations with approximate sampling errors

8 COVS T 1 1 791.401 14.3579

9 COVS T 1 2 114.298 2.78818 corr 0.713 0.008

10 COVS T 2 2 32.4767 0.721206

======== end of file ============================10-02-2015==========14:22====

======= Version 30-08-2013 ======================================= **KM** ====

Program WOMBAT: Summary of Pedigree Information

==============================================================================

Two traits analysis (WS x LEA)

Analysis type : "muv 2"

Data file : "DADOS.dat"

Pedigree file : "ReducedPedFile.dat"

Parameter file : "wombat.par"

No. of animal IDs in data file = = 8100

No. of animal IDs in total = = 8497

*****Pedigree Structure for random effect : 1 ****************************

Original no. of animals = 8497

No. of animals after pruning = 8440

... proportion (%) remaining = 99.3

No. of levels w/out records = 340

No. of levels with records = 8100 100.0%

... 1 record(s) = 5839 72.1%

... 2 record(s) = 2261 27.9%

No. of animals w/out offspring = 6060 71.8%

No. of animals with offspring = 2380 28.2%

... and records = 2040 24.2%

No. of animals with unknown sire = 313

No. of animals with unknown dam = 384

No. of animals with both parents unknown = 312

No. of animals with records =

... and unknown sire = 1

... and unknown dam = 53

... and both parents unknown = 0

No. of sires = 325

... with progeny in the data = 320

... with records & progeny in data = 289

No. of dams = 2055

... with progeny in the data = 2055

... with records & progeny in data = 1751

No. of animals with known/unpruned grand-parents

... with paternal grandsire = 7365

... with paternal granddam = 7063

... with maternal grandsire = 6542

... with maternal granddam = 6475

random effect no. = 1 NRM

no. of elements in NRM/GIN inverse 31866

log determinant = -5693.8614393533517

random effect no. = 2 IDE

no. of elements in NRM/GIN inverse 0

log determinant = 0.0000000000000000

======== end of file ============================10-02-2015==========14:34====

======= Version 30-08-2013 ======================================= **KM** ====

Program WOMBAT: Summary of information from Set-up step

==============================================================================

Analysis type : "muv 2"

Data file : "DADOS.dat"

Pedigree file : "ReducedPedFile.dat"

Parameter file : "wombat.par"

No. of traits = 2

nrec mean sdev min. max.

1 "WS" 8078 299.703 49.6462 160.030 489.760

2 "LEA" 2283 51.4305 8.89390 21.4000 83.4000

Numbers of individuals/records for pairs of traits

1 2

1 "WS" 8078 2261

2 "LEA" 2261 2283

Covariables

1"WS" nrec mean sdev min. max.

1 "idv(2)" 8078 6.63568 3.07616 2.00000 18.0000

2 "idade(1)" 8078 473.414 105.028 293.000 645.000

2"LEA" nrec mean sdev min. max.

1 "idv(2)" 2283 6.11082 2.92852 2.00000 17.0000

2 "idade(1)" 2283 448.951 98.8881 306.000 609.000

Fixed effects

1 "WS" nlev

1 "gc" 201

2 "mn" 4

2 "LEA" nlev

1 "gc" 65

2 "mn" 4

Random effects nlev

1 "animal" 8440 NRM

2 "peanim" 2107 IDE

======== end of file ============================10-02-2015==========14:34====

======= Version 30-08-2013 ======================================= **KM** ====

Program WOMBAT: Estimates of covariance components

==============================================================================

Analysis type : "muv 2"

Data file : "DADOS.dat"

Pedigree file : "ReducedPedFile.dat"

Parameter file : "wombat.par"

No. of traits = 2 WS LEA

No. of records = 10361 8078 2283

No. of parameters = 7

Maximum log L = -34873.867

-1/2 AIC & AICC = -34880.867 -34880.872

-1/2 BIC = -34906.132 "Penalty factor" = 4.609

Parameter estimates with approx. sampling erors

1 CHOL Z 1 1 20.5414 0.439471

2 CHOL Z 1 2 2.50192 0.187144

3 CHOL Z 2 2 3.78212 0.146753

4 CHOL A 1 1 18.8984 0.819156

5 CHOL A 1 2 1.81085 0.304958

6 CHOL A 2 2 3.27739 0.242970

7 CHOL B 1 1 2.15303 0.596965E-01

Convergence criteria for last 3 iterates

Change in log likelihood = 0.065602 0.001633 0.000091

Change in parameter vector = 0.004027 0.001165 0.000340

Norm of gradient vector = 4.3823 0.4976 0.0783

Newton decrement = -0.1256 -0.0027 -0.0001

***** Estimates of residual covariances ************************************

Order of fit = 2

Covariance matrix

1 421.95

2 51.393 20.564

Eigenvalues of covariance matrix

Value 428.42 14.09

(%) 96.82 3.18

Trace 442.51

Matrix of correlations and variance ratios

1 0.4945

2 0.5517 0.5946

Covariances & correlations with approximate sampling errors

1 COVS Z 1 1 421.949 18.0547 vrat 0.495 0.027

2 COVS Z 1 2 51.3928 4.17353 corr 0.552 0.034

3 COVS Z 2 2 20.5640 1.37193 vrat 0.595 0.047

***** Estimates for RE 1 "animal" ***************************************

No. of levels = 8440

Covariance structure = NRM

Order of fit = 2

Covariance matrix

1 357.15

2 34.222 14.020

Eigenvalues of covariance matrix

Value 360.53 10.64

(%) 97.13 2.87

Trace 371.17

Matrix of correlations and variance ratios

1 0.4186

2 0.4836 0.4054

Covariances & correlations with approximate sampling errors

4 COVS A 1 1 357.149 30.9615 vrat 0.419 0.030

5 COVS A 1 2 34.2221 6.24968 corr 0.484 0.068

6 COVS A 2 2 14.0205 1.92811 vrat 0.405 0.047

***** Estimates for RE 2 "peanim" ***************************************

No. of levels = 2107

Covariance structure = IDE

Order of fit = 1

Covariance matrix

1 74.148

Matrix of correlations and variance ratios

1 0.0869

Covariances & correlations with approximate sampling errors

7 COVS B 1 1 74.1477 8.85272 vrat 0.087 0.010

***** Estimates of phenotypic covariances ***********************************

Covariance matrix

1 853.25

2 85.615 34.584

Eigenvalues of covariance matrix

Value 862.10 25.73

(%) 97.10 2.90

Trace 887.83

Correlation matrix

1 1.0000

2 0.4984 1.0000

Covariances & correlations with approximate sampling errors

8 COVS T 1 1 853.246 18.7581

9 COVS T 1 2 85.6149 4.07138 corr 0.498 0.018

10 COVS T 2 2 34.5845 1.19282

======== end of file ============================10-02-2015==========14:35====

======= Version 30-08-2013 ======================================= **KM** ====

Program WOMBAT: Summary of Pedigree Information

==============================================================================

Two traits analysis (WS x BF)

Analysis type : "muv 2"

Data file : "DADOS.dat"

Pedigree file : "ReducedPedFile.dat"

Parameter file : "wombat.par"

No. of animal IDs in data file = = 8100

No. of animal IDs in total = = 8497

*****Pedigree Structure for random effect : 1 ****************************

Original no. of animals = 8497

No. of animals after pruning = 8440

... proportion (%) remaining = 99.3

No. of levels w/out records = 340

No. of levels with records = 8100 100.0%

... 1 record(s) = 5837 72.1%

... 2 record(s) = 2263 27.9%

No. of animals w/out offspring = 6060 71.8%

No. of animals with offspring = 2380 28.2%

... and records = 2040 24.2%

No. of animals with unknown sire = 313

No. of animals with unknown dam = 384

No. of animals with both parents unknown = 312

No. of animals with records =

... and unknown sire = 1

... and unknown dam = 53

... and both parents unknown = 0

No. of sires = 325

... with progeny in the data = 320

... with records & progeny in data = 289

No. of dams = 2055

... with progeny in the data = 2055

... with records & progeny in data = 1751

No. of animals with known/unpruned grand-parents

... with paternal grandsire = 7365

... with paternal granddam = 7063

... with maternal grandsire = 6542

... with maternal granddam = 6475

Inbreeding coefficients for random effect 1 computed

No. of inbred animals = 5151

Average inbreeding coefficient = 1.5438 (in %)

... amongst inbred animals = 2.5295 (in %)

random effect no. = 1 NRM

no. of elements in NRM/GIN inverse 31866

log determinant = -5693.8614393533517

random effect no. = 2 IDE

no. of elements in NRM/GIN inverse 0

log determinant = 0.0000000000000000

======== end of file ============================10-02-2015==========14:39====

======= Version 30-08-2013 ======================================= **KM** ====

Program WOMBAT: Summary of information from Set-up step

==============================================================================

Analysis type : "muv 2"

Data file : "DADOS.dat"

Pedigree file : "ReducedPedFile.dat"

Parameter file : "wombat.par"

No. of traits = 2

nrec mean sdev min. max.

1 "WS" 8078 299.703 49.6462 160.030 489.760

2 "BF" 2285 1.75922 1.42798 0.00000 10.5000

Numbers of individuals/records for pairs of traits

1 2

1 "WS" 8078 2263

2 "BF" 2263 2285

Covariables

1"WS" nrec mean sdev min. max.

1 "idv(2)" 8078 6.63568 3.07616 2.00000 18.0000

2 "idade(1)" 8078 473.414 105.028 293.000 645.000

2"BF" nrec mean sdev min. max.

1 "idv(2)" 2285 6.10810 2.92868 2.00000 17.0000

2 "idade(1)" 2285 449.081 98.9421 306.000 609.000

Fixed effects

1 "WS" nlev

1 "gc" 201

2 "mn" 4

2 "BF" nlev

1 "gc" 65

2 "mn" 4

Random effects nlev

1 "animal" 8440 NRM

2 "peanim" 2107 IDE

======== end of file ============================10-02-2015==========14:39====

======= Version 30-08-2013 ======================================= **KM** ====

Program WOMBAT: Estimates of covariance components

==============================================================================

Analysis type : "muv 2"

Data file : "DADOS.dat"

Pedigree file : "ReducedPedFile.dat"

Parameter file : "wombat.par"

No. of traits = 2 WS BF

No. of records = 10363 8078 2285

No. of parameters = 7

Maximum log L = -31129.895

-1/2 AIC & AICC = -31136.895 -31136.900

-1/2 BIC = -31162.161 "Penalty factor" = 4.609

Parameter estimates with approx. sampling erors

1 CHOL Z 1 1 20.5974 0.435570

2 CHOL Z 1 2 0.313129 0.330681E-01

3 CHOL Z 2 2 0.729445 0.251393E-01

4 CHOL A 1 1 2.88984 0.472459E-01

5 CHOL A 1 2 -0.114174E-01 0.536867E-01

6 CHOL A 2 2 -0.631776 0.821258E-01

7 CHOL B 1 1 2.31018 0.513404E-01

Convergence criteria for last 3 iterates

Change in log likelihood = 0.008126 0.000576 0.000047

Change in parameter vector = 0.002246 0.000561 0.000154

Norm of gradient vector = 1.4637 0.4407 0.1333

Newton decrement = -0.0130 -0.0009 -0.0001

***** Estimates of residual covariances ************************************

Order of fit = 2

Covariance matrix

1 424.25

2 6.4496 0.63014

Eigenvalues of covariance matrix

Value 424.35 0.53

(%) 99.87 0.13

Trace 424.88

Matrix of correlations and variance ratios

1 0.4994

2 0.3945 0.6902

Covariances & correlations with approximate sampling errors

1 COVS Z 1 1 424.251 17.9432 vrat 0.499 0.027

2 COVS Z 1 2 6.44964 0.690367 corr 0.394 0.040

3 COVS Z 2 2 0.630140 0.363735E-01 vrat 0.690 0.045

***** Estimates for RE 1 "animal" ***************************************

No. of levels = 8440

Covariance structure = NRM

Order of fit = 2

Covariance matrix

1 323.66

2 -0.20540 0.28278

Eigenvalues of covariance matrix

Value 323.66 0.28

(%) 99.91 0.09

Trace 323.94

Matrix of correlations and variance ratios

1 0.3810

2 -0.0215 0.3098

Covariances & correlations with approximate sampling errors

4 COVS A 1 1 323.656 30.5829 vrat 0.381 0.030

5 COVS A 1 2 -0.205405 0.964808 corr -0.021 0.101

6 COVS A 2 2 0.282779 0.462647E-01 vrat 0.310 0.045

***** Estimates for RE 2 "peanim" ***************************************

No. of levels = 2107

Covariance structure = IDE

Order of fit = 1

Covariance matrix

1 101.53

Matrix of correlations and variance ratios

1 0.1195

Covariances & correlations with approximate sampling errors

7 COVS B 1 1 101.531 10.4253 vrat 0.120 0.012

***** Estimates of phenotypic covariances ***********************************

Covariance matrix

1 849.44

2 6.2442 0.91292

Eigenvalues of covariance matrix

Value 849.48 0.87

(%) 99.90 0.10

Trace 850.35

Correlation matrix

1 1.0000

2 0.2242 1.0000

Covariances & correlations with approximate sampling errors

8 COVS T 1 1 849.438 18.5657

9 COVS T 1 2 6.24424 0.654790 corr 0.224 0.023

10 COVS T 2 2 0.912918 0.308003E-01

======== end of file ============================10-02-2015==========14:40====

======= Version 30-08-2013 ======================================= **KM** ====

Program WOMBAT: Summary of Pedigree Information

==============================================================================

Two traits analysis (WS x RF)

Analysis type : "muv 2"

Data file : "DADOS.dat"

Pedigree file : "ReducedPedFile.dat"

Parameter file : "wombat.par"

No. of animal IDs in data file = = 8101

No. of animal IDs in total = = 8498

*****Pedigree Structure for random effect : 1 ****************************

Original no. of animals = 8498

No. of animals after pruning = 8441

... proportion (%) remaining = 99.3

No. of levels w/out records = 340

No. of levels with records = 8101 100.0%

... 1 record(s) = 6307 77.9%

... 2 record(s) = 1794 22.1%

No. of animals w/out offspring = 6061 71.8%

No. of animals with offspring = 2380 28.2%

... and records = 2040 24.2%

No. of animals with unknown sire = 313

No. of animals with unknown dam = 384

No. of animals with both parents unknown = 312

No. of animals with records =

... and unknown sire = 1

... and unknown dam = 53

... and both parents unknown = 0

No. of sires = 325

... with progeny in the data = 320

... with records & progeny in data = 289

No. of dams = 2055

... with progeny in the data = 2055

... with records & progeny in data = 1751

No. of animals with known/unpruned grand-parents

... with paternal grandsire = 7366

... with paternal granddam = 7064

... with maternal grandsire = 6543

... with maternal granddam = 6476

Inbreeding coefficients for random effect 1 computed

No. of inbred animals = 5152

Average inbreeding coefficient = 1.5441 (in %)

... amongst inbred animals = 2.5298 (in %)

random effect no. = 1 NRM

no. of elements in NRM/GIN inverse 31870

log determinant = -5694.5813996468823

random effect no. = 2 IDE

no. of elements in NRM/GIN inverse 0

log determinant = 0.0000000000000000

======== end of file ============================10-02-2015==========14:42====

======= Version 30-08-2013 ======================================= **KM** ====

Program WOMBAT: Summary of information from Set-up step

==============================================================================

Analysis type : "muv 2"

Data file : "DADOS.dat"

Pedigree file : "ReducedPedFile.dat"

Parameter file : "wombat.par"

No. of traits = 2

nrec mean sdev min. max.

1 "WS" 8078 299.703 49.6462 160.030 489.760

2 "RF" 1817 5.08663 2.54341 0.00000 19.2000

Numbers of individuals/records for pairs of traits

1 2

1 "WS" 8078 1794

2 "RF" 1794 1817

Covariables

1"WS" nrec mean sdev min. max.

1 "idv(2)" 8078 6.63568 3.07616 2.00000 18.0000

2 "idade(1)" 8078 473.414 105.028 293.000 645.000

2"RF" nrec mean sdev min. max.

1 "idv(2)" 1817 6.09191 3.03138 2.00000 17.0000

2 "idade(1)" 1817 469.100 100.703 306.000 609.000

Fixed effects

1 "WS" nlev

1 "gc" 201

2 "mn" 4

2 "RF" nlev

1 "gc" 51

2 "mn" 3

Random effects nlev

1 "animal" 8441 NRM

2 "peanim" 2107 IDE

======== end of file ============================10-02-2015==========14:42====

======= Version 30-08-2013 ======================================= **KM** ====

Program WOMBAT: Estimates of covariance components

==============================================================================

Analysis type : "muv 2"

Data file : "DADOS.dat"

Pedigree file : "ReducedPedFile.dat"

Parameter file : "wombat.par"

No. of traits = 2 WS RF

No. of records = 9895 8078 1817

No. of parameters = 7

Maximum log L = -31805.093

-1/2 AIC & AICC = -31812.093 -31812.099

-1/2 BIC = -31837.198 "Penalty factor" = 4.586

Parameter estimates with approx. sampling erors

1 CHOL Z 1 1 20.5742 0.437347

2 CHOL Z 1 2 0.492900 0.625026E-01

3 CHOL Z 2 2 1.16101 0.469793E-01

4 CHOL A 1 1 18.0855 0.849656

5 CHOL A 1 2 0.115492 0.104069

6 CHOL A 2 2 0.974888 0.773014E-01

7 CHOL B 1 1 2.30007 0.519678E-01

Convergence criteria for last 3 iterates

Change in log likelihood = 0.274553 0.006502 0.000352

Change in parameter vector = 0.006062 0.002196 0.000704

Norm of gradient vector = 14.2193 1.6663 0.2108

Newton decrement = -0.5001 -0.0108 -0.0006

***** Estimates of residual covariances ************************************

Order of fit = 2

Covariance matrix

1 423.30

2 10.141 1.5909

Eigenvalues of covariance matrix

Value 423.54 1.35

(%) 99.68 0.32

Trace 424.89

Matrix of correlations and variance ratios

1 0.4981

2 0.3908 0.6227

Covariances & correlations with approximate sampling errors

1 COVS Z 1 1 423.299 17.9962 vrat 0.498 0.027

2 COVS Z 1 2 10.1410 1.30735 corr 0.391 0.047

3 COVS Z 2 2 1.59089 0.112962 vrat 0.623 0.052

***** Estimates for RE 1 "animal" ***************************************

No. of levels = 8441

Covariance structure = NRM

Order of fit = 2

Covariance matrix

1 327.09

2 2.0887 0.96375

Eigenvalues of covariance matrix

Value 327.10 0.95

(%) 99.71 0.29

Trace 328.05

Matrix of correlations and variance ratios

1 0.3849

2 0.1176 0.3773

Covariances & correlations with approximate sampling errors

4 COVS A 1 1 327.086 30.7329 vrat 0.385 0.030

5 COVS A 1 2 2.08874 1.89464 corr 0.118 0.104

6 COVS A 2 2 0.963745 0.154726 vrat 0.377 0.052

***** Estimates for RE 2 "peanim" ***************************************

No. of levels = 2107

Covariance structure = IDE

Order of fit = 1

Covariance matrix

1 99.499

Matrix of correlations and variance ratios

1 0.1171

Covariances & correlations with approximate sampling errors

7 COVS B 1 1 99.4990 10.3415 vrat 0.117 0.012

***** Estimates of phenotypic covariances ***********************************

Covariance matrix

1 849.88

2 12.230 2.5546

Eigenvalues of covariance matrix

Value 850.06 2.38

(%) 99.72 0.28

Trace 852.44

Correlation matrix

1 1.0000

2 0.2625 1.0000

Covariances & correlations with approximate sampling errors

8 COVS T 1 1 849.884 18.6194

9 COVS T 1 2 12.2298 1.23599 corr 0.262 0.025

10 COVS T 2 2 2.55463 0.996584E-01

======== end of file ============================10-02-2015==========14:42====

======= Version 30-08-2013 ======================================= **KM** ====

Program WOMBAT: Summary of Pedigree Information

==============================================================================

Two traits analysis (HH x CC)

Analysis type : "muv 2"

Data file : "DADOS.dat"

Pedigree file : "ReducedPedFile.dat"

Parameter file : "wombat.par"

No. of animal IDs in data file = = 6552

No. of animal IDs in total = = 7093

*****Pedigree Structure for random effect : 1 ****************************

Original no. of animals = 7093

No. of animals after pruning = 7010

... proportion (%) remaining = 98.8

No. of levels w/out records = 458

No. of levels with records = 6552 100.0%

... 1 record(s) = 2680 40.9%

... 2 record(s) = 3872 59.1%

No. of animals w/out offspring = 4823 68.8%

No. of animals with offspring = 2187 31.2%

... and records = 1729 24.7%

No. of animals with unknown sire = 148

No. of animals with unknown dam = 237

No. of animals with both parents unknown = 148

No. of animals with records =

... and unknown sire = 0

... and unknown dam = 18

... and both parents unknown = 0

No. of sires = 320

... with progeny in the data = 283

... with records & progeny in data = 240

No. of dams = 1867

... with progeny in the data = 1836

... with records & progeny in data = 1489

No. of animals with known/unpruned grand-parents

... with paternal grandsire = 6731

... with paternal granddam = 6477

... with maternal grandsire = 6332

... with maternal granddam = 6104

Inbreeding coefficients for random effect 1 computed

No. of inbred animals = 5142

Average inbreeding coefficient = 1.8506 (in %)

... amongst inbred animals = 2.5229 (in %)

random effect no. = 1 NRM

no. of elements in NRM/GIN inverse 26799

log determinant = -4809.8566364521839

======== end of file ============================10-02-2015==========14:46====

======= Version 30-08-2013 ======================================= **KM** ====

Program WOMBAT: Summary of information from Set-up step

==============================================================================

Analysis type : "muv 2"

Data file : "DADOS.dat"

Pedigree file : "ReducedPedFile.dat"

Parameter file : "wombat.par"

No. of traits = 2

nrec mean sdev min. max.

1 "HH" 6548 132.269 5.43028 100.000 149.000

2 "CC" 3876 164.140 8.72028 128.000 192.000

Numbers of individuals/records for pairs of traits

1 2

1 "HH" 6548 3872

2 "CC" 3872 3876

Covariables

1"HH" nrec mean sdev min. max.

1 "idv(2)" 6548 6.40043 2.93455 2.00000 17.0000

2 "idade(1)" 6548 472.701 107.333 293.000 645.000

2"CC" nrec mean sdev min. max.

1 "idv(2)" 3876 6.27632 2.87660 2.00000 17.0000

2 "idade(1)" 3876 431.403 109.187 293.000 725.000

Fixed effects

1 "HH" nlev

1 "gc" 169

2 "mn" 4

2 "CC" nlev

1 "gc" 100

Random effects nlev

1 "animal" 7010 NRM

======== end of file ============================10-02-2015==========14:46====

======= Version 30-08-2013 ======================================= **KM** ====

Program WOMBAT: Estimates of covariance components

==============================================================================

Analysis type : "muv 2"

Data file : "DADOS.dat"

Pedigree file : "ReducedPedFile.dat"

Parameter file : "wombat.par"

No. of traits = 2 HH CC

No. of records = 10424 6548 3876

No. of parameters = 6

Maximum log L = -19356.018

-1/2 AIC & AICC = -19362.018 -19362.023

-1/2 BIC = -19383.693 "Penalty factor" = 4.612

Parameter estimates with approx. sampling erors

1 CHOL Z 1 1 5.12578 0.104569

2 CHOL Z 1 2 1.46202 0.777071E-01

3 CHOL Z 2 2 1.98268 0.623342E-01

4 CHOL A 1 1 3.12019 0.214443

5 CHOL A 1 2 1.95598 0.175314

6 CHOL A 2 2 2.34577 0.116018

Convergence criteria for last 3 iterates

Change in log likelihood = 0.260149 0.001992 0.000062

Change in parameter vector = 0.011479 0.001767 0.000330

Norm of gradient vector = 10.1298 0.5532 0.0551

Newton decrement = -0.4988 -0.0034 -0.0001

***** Estimates of residual covariances ************************************

Order of fit = 2

Covariance matrix

1 6.0685

2 7.4940 26.274

Eigenvalues of covariance matrix

Value 28.75 3.59

(%) 88.89 11.11

Trace 32.34

Matrix of correlations and variance ratios

1 0.3941

2 0.5935 0.7296

Covariances & correlations with approximate sampling errors

1 COVS Z 1 1 6.06852 0.318276 vrat 0.394 0.026

2 COVS Z 1 2 7.49397 0.486150 corr 0.593 0.025

3 COVS Z 2 2 26.2736 1.07199 vrat 0.730 0.033

***** Estimates for RE 1 "animal" ***************************************

No. of levels = 7010

Covariance structure = NRM

Order of fit = 2

Covariance matrix

1 9.3285

2 6.1030 9.7356

Eigenvalues of covariance matrix

Value 15.64 3.43

(%) 82.03 17.97

Trace 19.06

Matrix of correlations and variance ratios

1 0.6059

2 0.6404 0.2704

Covariances & correlations with approximate sampling errors

4 COVS A 1 1 9.32853 0.587751 vrat 0.606 0.026

5 COVS A 1 2 6.10304 0.745469 corr 0.640 0.047

6 COVS A 2 2 9.73559 1.33820 vrat 0.270 0.033

***** Estimates of phenotypic covariances ***********************************

Covariance matrix

1 15.397

2 13.597 36.009

Eigenvalues of covariance matrix

Value 42.76 8.64

(%) 83.19 16.81

Trace 51.41

Correlation matrix

1 1.0000

2 0.5775 1.0000

Covariances & correlations with approximate sampling errors

7 COVS T 1 1 15.3971 0.380023

8 COVS T 1 2 13.5970 0.489852 corr 0.577 0.013

9 COVS T 2 2 36.0092 0.917394

======== end of file ============================10-02-2015==========14:46====

======= Version 30-08-2013 ======================================= **KM** ====

Program WOMBAT: Summary of Pedigree Information

==============================================================================

Two traits analysis (HH x LEA)

Analysis type : "muv 2"

Data file : "DADOS.dat"

Pedigree file : "ReducedPedFile.dat"

Parameter file : "wombat.par"

No. of animal IDs in data file = = 6565

No. of animal IDs in total = = 7105

*****Pedigree Structure for random effect : 1 ****************************

Original no. of animals = 7105

No. of animals after pruning = 7022

... proportion (%) remaining = 98.8

No. of levels w/out records = 457

No. of levels with records = 6565 100.0%

... 1 record(s) = 4299 65.5%

... 2 record(s) = 2266 34.5%

No. of animals w/out offspring = 4835 68.9%

No. of animals with offspring = 2187 31.1%

... and records = 1730 24.6%

No. of animals with unknown sire = 148

No. of animals with unknown dam = 237

No. of animals with both parents unknown = 148

No. of animals with records =

... and unknown sire = 0

... and unknown dam = 18

... and both parents unknown = 0

No. of sires = 320

... with progeny in the data = 283

... with records & progeny in data = 241

No. of dams = 1867

... with progeny in the data = 1836

... with records & progeny in data = 1489

No. of animals with known/unpruned grand-parents

... with paternal grandsire = 6743

... with paternal granddam = 6489

... with maternal grandsire = 6344

... with maternal granddam = 6116

Inbreeding coefficients for random effect 1 computed

No. of inbred animals = 5154

Average inbreeding coefficient = 1.8525 (in %)

... amongst inbred animals = 2.5239 (in %)

random effect no. = 1 NRM

no. of elements in NRM/GIN inverse 26841

log determinant = -4818.4754531566459

======== end of file ============================10-02-2015==========14:50====

======= Version 30-08-2013 ======================================= **KM** ====

Program WOMBAT: Summary of information from Set-up step

==============================================================================

Analysis type : "muv 2"

Data file : "DADOS.dat"

Pedigree file : "ReducedPedFile.dat"

Parameter file : "wombat.par"

No. of traits = 2

nrec mean sdev min. max.

1 "HH" 6548 132.269 5.43028 100.000 149.000

2 "LEA" 2283 51.4305 8.89390 21.4000 83.4000

Numbers of individuals/records for pairs of traits

1 2

1 "HH" 6548 2266

2 "LEA" 2266 2283

Covariables

1"HH" nrec mean sdev min. max.

1 "idv(2)" 6548 6.40043 2.93455 2.00000 17.0000

2 "idade(1)" 6548 472.701 107.333 293.000 645.000

2"LEA" nrec mean sdev min. max.

1 "idv(2)" 2283 6.11082 2.92852 2.00000 17.0000

2 "idade(1)" 2283 448.951 98.8881 306.000 609.000

Fixed effects

1 "HH" nlev

1 "gc" 169

2 "mn" 4

2 "LEA" nlev

1 "gc" 65

2 "mn" 4

Random effects nlev

1 "animal" 7022 NRM

======== end of file ============================10-02-2015==========14:50====

======= Version 30-08-2013 ======================================= **KM** ====

Program WOMBAT: Estimates of covariance components

==============================================================================

Analysis type : "muv 2"

Data file : "DADOS.dat"

Pedigree file : "ReducedPedFile.dat"

Parameter file : "wombat.par"

No. of traits = 2 HH LEA

No. of records = 8831 6548 2283

No. of parameters = 6

Maximum log L = -16350.846

-1/2 AIC & AICC = -16356.846 -16356.851

-1/2 BIC = -16378.019 "Penalty factor" = 4.529

Parameter estimates with approx. sampling erors

1 CHOL Z 1 1 4.58811 0.164442

2 CHOL Z 1 2 1.11148 0.118619

3 CHOL Z 2 2 2.18309 0.806415E-01

4 CHOL A 1 1 3.89344 0.274494

5 CHOL A 1 2 0.583375 0.229414

6 CHOL A 2 2 3.01539 0.969987E-01

Convergence criteria for last 3 iterates

Change in log likelihood = 0.334577 0.001826 0.000028

Change in parameter vector = 0.016026 0.002177 0.000260

Norm of gradient vector = 14.0902 0.3390 0.0423

Newton decrement = -0.6401 -0.0033 -0.0000

***** Estimates of residual covariances ************************************

Order of fit = 2

Covariance matrix

1 6.0013

2 5.0996 21.051

Eigenvalues of covariance matrix

Value 22.62 4.44

(%) 83.60 16.40

Trace 27.05

Matrix of correlations and variance ratios

1 0.3888

2 0.4537 0.5814

Covariances & correlations with approximate sampling errors

1 COVS Z 1 1 6.00130 0.319010 vrat 0.389 0.026

2 COVS Z 1 2 5.09961 0.593651 corr 0.454 0.046

3 COVS Z 2 2 21.0508 1.50895 vrat 0.581 0.049

***** Estimates for RE 1 "animal" ***************************************

No. of levels = 7022

Covariance structure = NRM

Order of fit = 2

Covariance matrix

1 9.4329

2 2.2713 15.159

Eigenvalues of covariance matrix

Value 15.95 8.64

(%) 64.86 35.14

Trace 24.59

Matrix of correlations and variance ratios

1 0.6112

2 0.1899 0.4186

Covariances & correlations with approximate sampling errors

4 COVS A 1 1 9.43288 0.592359 vrat 0.611 0.026

5 COVS A 1 2 2.27133 0.932734 corr 0.190 0.073

6 COVS A 2 2 15.1589 2.13745 vrat 0.419 0.049

***** Estimates of phenotypic covariances ***********************************

Covariance matrix

1 15.434

2 7.3709 36.210

Eigenvalues of covariance matrix

Value 38.56 13.08

(%) 74.66 25.34

Trace 51.64

Correlation matrix

1 1.0000

2 0.3118 1.0000

Covariances & correlations with approximate sampling errors

7 COVS T 1 1 15.4342 0.382566

8 COVS T 1 2 7.37094 0.612328 corr 0.312 0.024

9 COVS T 2 2 36.2097 1.30140

======== end of file ============================10-02-2015==========14:50====

======= Version 30-08-2013 ======================================= **KM** ====

Program WOMBAT: Summary of Pedigree Information

==============================================================================

Two traits analysis (HH x BF)

Analysis type : "muv 2"

Data file : "DADOS.dat"

Pedigree file : "ReducedPedFile.dat"

Parameter file : "wombat.par"

No. of animal IDs in data file = = 6565

No. of animal IDs in total = = 7105

*****Pedigree Structure for random effect : 1 ****************************

Original no. of animals = 7105

No. of animals after pruning = 7022

... proportion (%) remaining = 98.8

No. of levels w/out records = 457

No. of levels with records = 6565 100.0%

... 1 record(s) = 4297 65.5%

... 2 record(s) = 2268 34.5%

No. of animals w/out offspring = 4835 68.9%

No. of animals with offspring = 2187 31.1%

... and records = 1730 24.6%

No. of animals with unknown sire = 148

No. of animals with unknown dam = 237

No. of animals with both parents unknown = 148

No. of animals with records =

... and unknown sire = 0

... and unknown dam = 18

... and both parents unknown = 0

No. of sires = 320

... with progeny in the data = 283

... with records & progeny in data = 241

No. of dams = 1867

... with progeny in the data = 1836

... with records & progeny in data = 1489

No. of animals with known/unpruned grand-parents

... with paternal grandsire = 6743

... with paternal granddam = 6489

... with maternal grandsire = 6344

... with maternal granddam = 6116

Inbreeding coefficients for random effect 1 computed

No. of inbred animals = 5154

Average inbreeding coefficient = 1.8525 (in %)

... amongst inbred animals = 2.5239 (in %)

random effect no. = 1 NRM

no. of elements in NRM/GIN inverse 26841

log determinant = -4818.4754531566459

======== end of file ============================10-02-2015==========14:53====

======= Version 30-08-2013 ======================================= **KM** ====

Program WOMBAT: Summary of information from Set-up step

==============================================================================

Analysis type : "muv 2"

Data file : "DADOS.dat"

Pedigree file : "ReducedPedFile.dat"

Parameter file : "wombat.par"

No. of traits = 2

nrec mean sdev min. max.

1 "HH" 6548 132.269 5.43028 100.000 149.000

2 "BF" 2285 1.75922 1.42798 0.00000 10.5000

Numbers of individuals/records for pairs of traits

1 2

1 "HH" 6548 2268

2 "BF" 2268 2285

Covariables

1"HH" nrec mean sdev min. max.

1 "idv(2)" 6548 6.40043 2.93455 2.00000 17.0000

2 "idade(1)" 6548 472.701 107.333 293.000 645.000

2"BF" nrec mean sdev min. max.

1 "idv(2)" 2285 6.10810 2.92868 2.00000 17.0000

2 "idade(1)" 2285 449.081 98.9421 306.000 609.000

Fixed effects

1 "HH" nlev

1 "gc" 169

2 "mn" 4

2 "BF" nlev

1 "gc" 65

2 "mn" 4

Random effects nlev

1 "animal" 7022 NRM

======== end of file ============================10-02-2015==========14:53====

======= Version 30-08-2013 ======================================= **KM** ====

Program WOMBAT: Estimates of covariance components

==============================================================================

Analysis type : "muv 2"

Data file : "DADOS.dat"

Pedigree file : "ReducedPedFile.dat"

Parameter file : "wombat.par"

No. of traits = 2 HH BF

No. of records = 8833 6548 2285

No. of parameters = 6

Maximum log L = -12460.673

-1/2 AIC & AICC = -12466.673 -12466.678

-1/2 BIC = -12487.847 "Penalty factor" = 4.529

Parameter estimates with approx. sampling erors

1 CHOL Z 1 1 2.46393 0.648068E-01

2 CHOL Z 1 2 0.196936 0.389808E-01

3 CHOL Z 2 2 0.777038 0.248050E-01

4 CHOL A 1 1 1.11452 0.317076E-01

5 CHOL A 1 2 -0.795365E-01 0.472351E-01

6 CHOL A 2 2 -0.650401 0.869896E-01

Convergence criteria for last 3 iterates

Change in log likelihood = 0.347709 0.009209 0.000254

Change in parameter vector = 0.017806 0.002853 0.000480

Norm of gradient vector = 16.9000 3.1035 0.4143

Newton decrement = -0.6087 -0.0158 -0.0004

***** Estimates of residual covariances ************************************

Order of fit = 2

Covariance matrix

1 6.0709

2 0.48524 0.64257

Eigenvalues of covariance matrix

Value 6.11 0.60

(%) 91.07 8.93

Trace 6.71

Matrix of correlations and variance ratios

1 0.3952

2 0.2457 0.6975

Covariances & correlations with approximate sampling errors

1 COVS Z 1 1 6.07093 0.319358 vrat 0.395 0.026

2 COVS Z 1 2 0.485237 0.962470E-01 corr 0.246 0.048

3 COVS Z 2 2 0.642572 0.372675E-01 vrat 0.698 0.045

***** Estimates for RE 1 "animal" ***************************************

No. of levels = 7022

Covariance structure = NRM

Order of fit = 2

Covariance matrix

1 9.2909

2 -0.24243 0.27864

Eigenvalues of covariance matrix

Value 9.30 0.27

(%) 97.16 2.84

Trace 9.57

Matrix of correlations and variance ratios

1 0.6048

2 -0.1507 0.3025

Covariances & correlations with approximate sampling errors

4 COVS A 1 1 9.29087 0.589183 vrat 0.605 0.026

5 COVS A 1 2 -0.242435 0.143673 corr -0.151 0.090

6 COVS A 2 2 0.278639 0.469299E-01 vrat 0.302 0.045

***** Estimates of phenotypic covariances ***********************************

Covariance matrix

1 15.362

2 0.24280 0.92121

Eigenvalues of covariance matrix

Value 15.37 0.92

(%) 94.37 5.63

Trace 16.28

Correlation matrix

1 1.0000

2 0.0645 1.0000

Covariances & correlations with approximate sampling errors

7 COVS T 1 1 15.3618 0.379859

8 COVS T 1 2 0.242802 0.964311E-01 corr 0.065 0.026

9 COVS T 2 2 0.921212 0.311742E-01

======== end of file ============================10-02-2015==========14:53====

======= Version 30-08-2013 ======================================= **KM** ====

Program WOMBAT: Summary of Pedigree Information

==============================================================================

Two traits analysis (HH x RF)

Analysis type : "muv 2"

Data file : "DADOS.dat"

Pedigree file : "ReducedPedFile.dat"

Parameter file : "wombat.par"

No. of animal IDs in data file = = 6565

No. of animal IDs in total = = 7105

*****Pedigree Structure for random effect : 1 ****************************

Original no. of animals = 7105

No. of animals after pruning = 7022

... proportion (%) remaining = 98.8

No. of levels w/out records = 457

No. of levels with records = 6565 100.0%

... 1 record(s) = 4765 72.6%

... 2 record(s) = 1800 27.4%

No. of animals w/out offspring = 4835 68.9%

No. of animals with offspring = 2187 31.1%

... and records = 1730 24.6%

No. of animals with unknown sire = 148

No. of animals with unknown dam = 237

No. of animals with both parents unknown = 148

No. of animals with records =

... and unknown sire = 0

... and unknown dam = 18

... and both parents unknown = 0

No. of sires = 320

... with progeny in the data = 283

... with records & progeny in data = 241

No. of dams = 1867

... with progeny in the data = 1836

... with records & progeny in data = 1489

No. of animals with known/unpruned grand-parents

... with paternal grandsire = 6743

... with paternal granddam = 6489

... with maternal grandsire = 6344

... with maternal granddam = 6116

Inbreeding coefficients for random effect 1 computed

No. of inbred animals = 5154

Average inbreeding coefficient = 1.8525 (in %)

... amongst inbred animals = 2.5239 (in %)

random effect no. = 1 NRM

no. of elements in NRM/GIN inverse 26841

log determinant = -4818.4754531566459

======== end of file ============================10-02-2015==========14:55====

======= Version 30-08-2013 ======================================= **KM** ====

Program WOMBAT: Summary of information from Set-up step

==============================================================================

Analysis type : "muv 2"

Data file : "DADOS.dat"

Pedigree file : "ReducedPedFile.dat"

Parameter file : "wombat.par"

No. of traits = 2

nrec mean sdev min. max.

1 "HH" 6548 132.269 5.43028 100.000 149.000

2 "RF" 1817 5.08663 2.54341 0.00000 19.2000

Numbers of individuals/records for pairs of traits

1 2

1 "HH" 6548 1800

2 "RF" 1800 1817

Covariables

1"HH" nrec mean sdev min. max.

1 "idv(2)" 6548 6.40043 2.93455 2.00000 17.0000

2 "idade(1)" 6548 472.701 107.333 293.000 645.000

2"RF" nrec mean sdev min. max.

1 "idv(2)" 1817 6.09191 3.03138 2.00000 17.0000

2 "idade(1)" 1817 469.100 100.703 306.000 609.000

Fixed effects

1 "HH" nlev

1 "gc" 169

2 "mn" 4

2 "RF" nlev

1 "gc" 51

2 "mn" 3

Random effects nlev

1 "animal" 7022 NRM

======== end of file ============================10-02-2015==========14:55====

======= Version 30-08-2013 ======================================= **KM** ====

Program WOMBAT: Estimates of covariance components

==============================================================================

Analysis type : "muv 2"

Data file : "DADOS.dat"

Pedigree file : "ReducedPedFile.dat"

Parameter file : "wombat.par"

No. of traits = 2 HH RF

No. of records = 8365 6548 1817

No. of parameters = 6

Maximum log L = -13140.056

-1/2 AIC & AICC = -13146.056 -13146.061

-1/2 BIC = -13167.067 "Penalty factor" = 4.502

Parameter estimates with approx. sampling erors

1 CHOL Z 1 1 2.45313 0.652413E-01

2 CHOL Z 1 2 0.226991 0.740709E-01

3 CHOL Z 2 2 1.24910 0.469336E-01

4 CHOL A 1 1 3.06448 0.968260E-01

5 CHOL A 1 2 -0.300186E-01 0.925413E-01

6 CHOL A 2 2 0.992199 0.807553E-01

Convergence criteria for last 3 iterates

Change in log likelihood = 0.294766 0.004217 0.000091

Change in parameter vector = 0.015288 0.002264 0.000332

Norm of gradient vector = 17.4908 0.7888 0.1168

Newton decrement = -0.5406 -0.0074 -0.0002

***** Estimates of residual covariances ************************************

Order of fit = 2

Covariance matrix

1 6.0178

2 0.55684 1.6118

Eigenvalues of covariance matrix

Value 6.09 1.54

(%) 79.78 20.22

Trace 7.63

Matrix of correlations and variance ratios

1 0.3905

2 0.1788 0.6206

Covariances & correlations with approximate sampling errors

1 COVS Z 1 1 6.01783 0.320090 vrat 0.391 0.026

2 COVS Z 1 2 0.556838 0.181984 corr 0.179 0.058

3 COVS Z 2 2 1.61178 0.116692 vrat 0.621 0.052

***** Estimates for RE 1 "animal" ***************************************

No. of levels = 7022

Covariance structure = NRM

Order of fit = 2

Covariance matrix

1 9.3910

2 -0.91991E-01 0.98536

Eigenvalues of covariance matrix

Value 9.39 0.98

(%) 90.51 9.49

Trace 10.38

Matrix of correlations and variance ratios

1 0.6095

2 -0.0302 0.3794

Covariances & correlations with approximate sampling errors

4 COVS A 1 1 9.39101 0.593442 vrat 0.609 0.026

5 COVS A 1 2 -0.919913E-01 0.283486 corr -0.030 0.093

6 COVS A 2 2 0.985359 0.159861 vrat 0.379 0.052

***** Estimates of phenotypic covariances ***********************************

Covariance matrix

1 15.409

2 0.46485 2.5971

Eigenvalues of covariance matrix

Value 15.43 2.58

(%) 85.67 14.33

Trace 18.01

Correlation matrix

1 1.0000

2 0.0735 1.0000

Covariances & correlations with approximate sampling errors

7 COVS T 1 1 15.4088 0.382420

8 COVS T 1 2 0.464846 0.185033 corr 0.073 0.029

9 COVS T 2 2 2.59714 0.102356

======== end of file ============================10-02-2015==========14:55====

======= Version 30-08-2013 ======================================= **KM** ====

Program WOMBAT: Summary of Pedigree Information

==============================================================================

Two traits analysis (CC x LEA)

Analysis type : "muv 2"

Data file : "DADOS.dat"

Pedigree file : "ReducedPedFile.dat"

Parameter file : "wombat.par"

No. of animal IDs in data file = = 4015

No. of animal IDs in total = = 5458

*****Pedigree Structure for random effect : 1 ****************************

Original no. of animals = 5458

No. of animals after pruning = 5370

... proportion (%) remaining = 98.4

No. of levels w/out records = 1355

No. of levels with records = 4015 100.0%

... 1 record(s) = 1871 46.6%

... 2 record(s) = 2144 53.4%

No. of animals w/out offspring = 3345 62.3%

No. of animals with offspring = 2025 37.7%

... and records = 670 12.5%

No. of animals with unknown sire = 94

No. of animals with unknown dam = 188

No. of animals with both parents unknown = 94

No. of animals with records =

... and unknown sire = 0

... and unknown dam = 0

... and both parents unknown = 0

No. of sires = 318

... with progeny in the data = 255

... with records & progeny in data = 218

No. of dams = 1707

... with progeny in the data = 1517

... with records & progeny in data = 452

No. of animals with known/unpruned grand-parents

... with paternal grandsire = 5182

... with paternal granddam = 4994

... with maternal grandsire = 4993

... with maternal granddam = 4778

Inbreeding coefficients for random effect 1 computed

No. of inbred animals = 4364

Average inbreeding coefficient = 2.1437 (in %)

... amongst inbred animals = 2.6379 (in %)

random effect no. = 1 NRM

no. of elements in NRM/GIN inverse 20614

log determinant = -3700.8634178252159

======== end of file ============================10-02-2015==========14:59====

======= Version 30-08-2013 ======================================= **KM** ====

Program WOMBAT: Summary of information from Set-up step

==============================================================================

Analysis type : "muv 2"

Data file : "DADOS.dat"

Pedigree file : "ReducedPedFile.dat"

Parameter file : "wombat.par"

No. of traits = 2

nrec mean sdev min. max.

1 "CC" 3876 164.140 8.72028 128.000 192.000

2 "LEA" 2283 51.4305 8.89390 21.4000 83.4000

Numbers of individuals/records for pairs of traits

1 2

1 "CC" 3876 2144

2 "LEA" 2144 2283

Covariables

1"CC" nrec mean sdev min. max.

1 "idv(2)" 3876 6.27632 2.87660 2.00000 17.0000

2 "idade(1)" 3876 431.403 109.187 293.000 725.000

2"LEA" nrec mean sdev min. max.

1 "idv(2)" 2283 6.11082 2.92852 2.00000 17.0000

2 "idade(1)" 2283 448.951 98.8881 306.000 609.000

Fixed effects

1 "CC" nlev

1 "gc" 100

2 "mn" 4

2 "LEA" nlev

1 "gc" 65

2 "mn" 4

Random effects nlev

1 "animal" 5370 NRM

======== end of file ============================10-02-2015==========14:59====

======= Version 30-08-2013 ======================================= **KM** ====

Program WOMBAT: Estimates of covariance components

==============================================================================

Analysis type : "muv 2"

Data file : "DADOS.dat"

Pedigree file : "ReducedPedFile.dat"

Parameter file : "wombat.par"

No. of traits = 2 CC LEA

No. of records = 6159 3876 2283

No. of parameters = 6

Maximum log L = -13526.599

-1/2 AIC & AICC = -13532.599 -13532.606

-1/2 BIC = -13552.689 "Penalty factor" = 4.348

Parameter estimates with approx. sampling erors

1 CHOL Z 1 1 5.00497 0.119072

2 CHOL Z 1 2 2.26880 0.188998

3 CHOL Z 2 2 3.97947 0.153629

4 CHOL A 1 1 3.87908 0.270497

5 CHOL A 1 2 1.29027 0.323291

6 CHOL A 2 2 3.08918 0.221173

Convergence criteria for last 3 iterates

Change in log likelihood = 0.194940 0.001538 0.000046

Change in parameter vector = 0.009598 0.001466 0.000259

Norm of gradient vector = 8.4199 0.2204 0.0366

Newton decrement = -0.3734 -0.0026 -0.0001

***** Estimates of residual covariances ************************************

Order of fit = 2

Covariance matrix

1 25.050

2 11.355 20.984

Eigenvalues of covariance matrix

Value 34.55 11.48

(%) 75.06 24.94

Trace 46.03

Matrix of correlations and variance ratios

1 0.6909

2 0.4953 0.5824

Covariances & correlations with approximate sampling errors

1 COVS Z 1 1 25.0497 1.19190 vrat 0.691 0.039

2 COVS Z 1 2 11.3553 1.04955 corr 0.495 0.034

3 COVS Z 2 2 20.9836 1.48491 vrat 0.582 0.049

***** Estimates for RE 1 "animal" ***************************************

No. of levels = 5370

Covariance structure = NRM

Order of fit = 2

Covariance matrix

1 11.208

2 5.0051 15.047

Eigenvalues of covariance matrix

Value 18.49 7.77

(%) 70.42 29.58

Trace 26.26

Matrix of correlations and variance ratios

1 0.3091

2 0.3854 0.4176

Covariances & correlations with approximate sampling errors

4 COVS A 1 1 11.2079 1.58663 vrat 0.309 0.039

5 COVS A 1 2 5.00508 1.39810 corr 0.385 0.086

6 COVS A 2 2 15.0472 2.09856 vrat 0.418 0.049

***** Estimates of phenotypic covariances ***********************************

Covariance matrix

1 36.258

2 16.360 36.031

Eigenvalues of covariance matrix

Value 52.50 19.78

(%) 72.63 27.37

Trace 72.29

Correlation matrix

1 1.0000

2 0.4526 1.0000

Covariances & correlations with approximate sampling errors

7 COVS T 1 1 36.2575 0.984305

8 COVS T 1 2 16.3604 0.910076 corr 0.453 0.020

9 COVS T 2 2 36.0309 1.28251

======== end of file ============================10-02-2015==========14:59====

======= Version 30-08-2013 ======================================= **KM** ====

Program WOMBAT: Summary of Pedigree Information

==============================================================================

Two traits analysis (CC x BF)

Analysis type : "muv 2"

Data file : "DADOS.dat"

Pedigree file : "ReducedPedFile.dat"

Parameter file : "wombat.par"

No. of animal IDs in data file = = 4017

No. of animal IDs in total = = 5459

*****Pedigree Structure for random effect : 1 ****************************

Original no. of animals = 5459

No. of animals after pruning = 5371

... proportion (%) remaining = 98.4

No. of levels w/out records = 1354

No. of levels with records = 4017 100.0%

... 1 record(s) = 1873 46.6%

... 2 record(s) = 2144 53.4%

No. of animals w/out offspring = 3345 62.3%

No. of animals with offspring = 2026 37.7%

... and records = 672 12.5%

No. of animals with unknown sire = 94

No. of animals with unknown dam = 188

No. of animals with both parents unknown = 94

No. of animals with records =

... and unknown sire = 0

... and unknown dam = 0

... and both parents unknown = 0

No. of sires = 318

... with progeny in the data = 255

... with records & progeny in data = 218

No. of dams = 1708

... with progeny in the data = 1518

... with records & progeny in data = 454

No. of animals with known/unpruned grand-parents

... with paternal grandsire = 5183

... with paternal granddam = 4995

... with maternal grandsire = 4994

... with maternal granddam = 4779

Inbreeding coefficients for random effect 1 computed

No. of inbred animals = 4365

Average inbreeding coefficient = 2.1440 (in %)

... amongst inbred animals = 2.6381 (in %)

random effect no. = 1 NRM

no. of elements in NRM/GIN inverse 20618

log determinant = -3701.5766085983810

======== end of file ============================10-02-2015==========15:01====

======= Version 30-08-2013 ======================================= **KM** ====

Program WOMBAT: Summary of information from Set-up step

==============================================================================

Analysis type : "muv 2"

Data file : "DADOS.dat"

Pedigree file : "ReducedPedFile.dat"

Parameter file : "wombat.par"

No. of traits = 2

nrec mean sdev min. max.

1 "CC" 3876 164.140 8.72028 128.000 192.000

2 "BF" 2285 1.75922 1.42798 0.00000 10.5000

Numbers of individuals/records for pairs of traits

1 2

1 "CC" 3876 2144

2 "BF" 2144 2285

Covariables

1"CC" nrec mean sdev min. max.

1 "idv(2)" 3876 6.27632 2.87660 2.00000 17.0000

2 "idade(1)" 3876 431.403 109.187 293.000 725.000

2"BF" nrec mean sdev min. max.

1 "idv(2)" 2285 6.10810 2.92868 2.00000 17.0000

2 "idade(1)" 2285 449.081 98.9421 306.000 609.000

Fixed effects

1 "CC" nlev

1 "gc" 100

2 "mn" 4

2 "BF" nlev

1 "gc" 65

2 "mn" 4

Random effects nlev

1 "animal" 5371 NRM

======== end of file ============================10-02-2015==========15:01====

======= Version 30-08-2013 ======================================= **KM** ====

Program WOMBAT: Estimates of covariance components

==============================================================================

Analysis type : "muv 2"

Data file : "DADOS.dat"

Pedigree file : "ReducedPedFile.dat"

Parameter file : "wombat.par"

No. of traits = 2 CC BF

No. of records = 6161 3876 2285

No. of parameters = 6

Maximum log L = -9677.170

-1/2 AIC & AICC = -9683.170 -9683.176

-1/2 BIC = -9703.260 "Penalty factor" = 4.348

Parameter estimates with approx. sampling erors

1 CHOL Z 1 1 5.03149 0.118051

2 CHOL Z 1 2 0.260681 0.313631E-01

3 CHOL Z 2 2 0.760665 0.229988E-01

4 CHOL A 1 1 1.18945 0.723968E-01

5 CHOL A 1 2 0.897630E-01 0.601563E-01

6 CHOL A 2 2 -0.666630 0.844398E-01

Convergence criteria for last 3 iterates

Change in log likelihood = 25.625707 0.279136 0.000159

Change in parameter vector = 0.037267 0.005155 0.000251

Norm of gradient vector = 442.0985 33.6485 0.2966

Newton decrement = -48.9635 -0.5575 -0.0003

***** Estimates of residual covariances ************************************

Order of fit = 2

Covariance matrix

1 25.316

2 1.3116 0.64657

Eigenvalues of covariance matrix

Value 25.39 0.58

(%) 97.78 2.22

Trace 25.96

Matrix of correlations and variance ratios

1 0.7011

2 0.3242 0.7041

Covariances & correlations with approximate sampling errors

1 COVS Z 1 1 25.3159 1.18794 vrat 0.701 0.039

2 COVS Z 1 2 1.31161 0.164393 corr 0.324 0.037

3 COVS Z 2 2 0.646566 0.370960E-01 vrat 0.704 0.045

***** Estimates for RE 1 "animal" ***************************************

No. of levels = 5371

Covariance structure = NRM

Order of fit = 2

Covariance matrix

1 10.793

2 0.29490 0.27167

Eigenvalues of covariance matrix

Value 10.80 0.26

(%) 97.62 2.38

Trace 11.06

Matrix of correlations and variance ratios

1 0.2989

2 0.1722 0.2959

Covariances & correlations with approximate sampling errors

4 COVS A 1 1 10.7931 1.56277 vrat 0.299 0.039

5 COVS A 1 2 0.294897 0.202571 corr 0.172 0.112

6 COVS A 2 2 0.271674 0.463386E-01 vrat 0.296 0.045

***** Estimates of phenotypic covariances ***********************************

Covariance matrix

1 36.109

2 1.6065 0.91824

Eigenvalues of covariance matrix

Value 36.18 0.85

(%) 97.72 2.28

Trace 37.03

Correlation matrix

1 1.0000

2 0.2790 1.0000

Covariances & correlations with approximate sampling errors

7 COVS T 1 1 36.1089 0.974096

8 COVS T 1 2 1.60651 0.139647 corr 0.279 0.022

9 COVS T 2 2 0.918240 0.309063E-01

======== end of file ============================10-02-2015==========15:01====

======= Version 30-08-2013 ======================================= **KM** ====

Program WOMBAT: Summary of Pedigree Information

==============================================================================

Two traits analysis (CC x RF)

Analysis type : "muv 2"

Data file : "DADOS.dat"

Pedigree file : "ReducedPedFile.dat"

Parameter file : "wombat.par"

No. of animal IDs in data file = = 4016

No. of animal IDs in total = = 5457

*****Pedigree Structure for random effect : 1 ****************************

Original no. of animals = 5457

No. of animals after pruning = 5369

... proportion (%) remaining = 98.4

No. of levels w/out records = 1353

No. of levels with records = 4016 100.0%

... 1 record(s) = 2339 58.2%

... 2 record(s) = 1677 41.8%

No. of animals w/out offspring = 3344 62.3%

No. of animals with offspring = 2025 37.7%

... and records = 672 12.5%

No. of animals with unknown sire = 94

No. of animals with unknown dam = 188

No. of animals with both parents unknown = 94

No. of animals with records =

... and unknown sire = 0

... and unknown dam = 0

... and both parents unknown = 0

No. of sires = 318

... with progeny in the data = 255

... with records & progeny in data = 218

No. of dams = 1707

... with progeny in the data = 1517

... with records & progeny in data = 454

No. of animals with known/unpruned grand-parents

... with paternal grandsire = 5181

... with paternal granddam = 4993

... with maternal grandsire = 4992

... with maternal granddam = 4777

Inbreeding coefficients for random effect 1 computed

No. of inbred animals = 4363

Average inbreeding coefficient = 2.1440 (in %)

... amongst inbred animals = 2.6384 (in %)

random effect no. = 1 NRM

no. of elements in NRM/GIN inverse 20610

log determinant = -3700.1515718108940

======== end of file ============================10-02-2015==========15:03====

======= Version 30-08-2013 ======================================= **KM** ====

Program WOMBAT: Summary of information from Set-up step

==============================================================================

Analysis type : "muv 2"

Data file : "DADOS.dat"

Pedigree file : "ReducedPedFile.dat"

Parameter file : "wombat.par"

No. of traits = 2

nrec mean sdev min. max.

1 "CC" 3876 164.140 8.72028 128.000 192.000

2 "RF" 1817 5.08663 2.54341 0.00000 19.2000

Numbers of individuals/records for pairs of traits

1 2

1 "CC" 3876 1677

2 "RF" 1677 1817

Covariables

1"CC" nrec mean sdev min. max.

1 "idv(2)" 3876 6.27632 2.87660 2.00000 17.0000

2 "idade(1)" 3876 431.403 109.187 293.000 725.000

2"RF" nrec mean sdev min. max.

1 "idv(2)" 1817 6.09191 3.03138 2.00000 17.0000

2 "idade(1)" 1817 469.100 100.703 306.000 609.000

Fixed effects

1 "CC" nlev

1 "gc" 100

2 "mn" 4

2 "RF" nlev

1 "gc" 51

2 "mn" 3

Random effects nlev

1 "animal" 5369 NRM

======== end of file ============================10-02-2015==========15:03====

======= Version 30-08-2013 ======================================= **KM** ====

Program WOMBAT: Estimates of covariance components

==============================================================================

Analysis type : "muv 2"

Data file : "DADOS.dat"

Pedigree file : "ReducedPedFile.dat"

Parameter file : "wombat.par"

No. of traits = 2 CC RF

No. of records = 5693 3876 1817

No. of parameters = 6

Maximum log L = -10356.265

-1/2 AIC & AICC = -10362.265 -10362.272

-1/2 BIC = -10382.119 "Penalty factor" = 4.309

Parameter estimates with approx. sampling erors

1 CHOL Z 1 1 5.02169 0.118264

2 CHOL Z 1 2 0.395081 0.581413E-01

3 CHOL Z 2 2 1.20682 0.441493E-01

4 CHOL A 1 1 3.30326 0.237346

5 CHOL A 1 2 0.291306 0.114292

6 CHOL A 2 2 0.941566 0.776501E-01

Convergence criteria for last 3 iterates

Change in log likelihood = 18.902031 0.169053 0.000302

Change in parameter vector = 0.035145 0.006235 0.000722

Norm of gradient vector = 215.5688 15.5289 0.3012

Newton decrement = -36.5835 -0.3389 -0.0006

***** Estimates of residual covariances ************************************

Order of fit = 2

Covariance matrix

1 25.217

2 1.9840 1.6125

Eigenvalues of covariance matrix

Value 25.38 1.45

(%) 94.61 5.39

Trace 26.83

Matrix of correlations and variance ratios

1 0.6980

2 0.3111 0.6241

Covariances & correlations with approximate sampling errors

1 COVS Z 1 1 25.2174 1.18777 vrat 0.698 0.039

2 COVS Z 1 2 1.98397 0.302770 corr 0.311 0.043

3 COVS Z 2 2 1.61249 0.115598 vrat 0.624 0.052

***** Estimates for RE 1 "animal" ***************************************

No. of levels = 5369

Covariance structure = NRM

Order of fit = 2

Covariance matrix

1 10.912

2 0.96226 0.97141

Eigenvalues of covariance matrix

Value 11.00 0.88

(%) 92.60 7.40

Trace 11.88

Matrix of correlations and variance ratios

1 0.3020

2 0.2956 0.3759

Covariances & correlations with approximate sampling errors

4 COVS A 1 1 10.9115 1.56803 vrat 0.302 0.039

5 COVS A 1 2 0.962259 0.393845 corr 0.296 0.109

6 COVS A 2 2 0.971405 0.157845 vrat 0.376 0.052

***** Estimates of phenotypic covariances ***********************************

Covariance matrix

1 36.129

2 2.9462 2.5839

Eigenvalues of covariance matrix

Value 36.39 2.33

(%) 93.99 6.01

Trace 38.71

Correlation matrix

1 1.0000

2 0.3049 1.0000

Covariances & correlations with approximate sampling errors

7 COVS T 1 1 36.1289 0.976245

8 COVS T 1 2 2.94623 0.264208 corr 0.305 0.025

9 COVS T 2 2 2.58390 0.101269

======== end of file ============================10-02-2015==========15:03====

======= Version 30-08-2013 ======================================= **KM** ====

Program WOMBAT: Summary of Pedigree Information

==============================================================================

Two traits analysis (LEA x BF)

Analysis type : "muv 2"

Data file : "DADOS.dat"

Pedigree file : "ReducedPedFile.dat"

Parameter file : "wombat.par"

No. of animal IDs in data file = = 2285

No. of animal IDs in total = = 3697

*****Pedigree Structure for random effect : 1 ****************************

Original no. of animals = 3697

No. of animals after pruning = 3606

... proportion (%) remaining = 97.5

No. of levels w/out records = 1321

No. of levels with records = 2285 100.0%

... 1 record(s) = 2 0.1%

... 2 record(s) = 2283 99.9%

No. of animals w/out offspring = 1863 51.7%

No. of animals with offspring = 1743 48.3%

... and records = 422 11.7%

No. of animals with unknown sire = 64

No. of animals with unknown dam = 161

No. of animals with both parents unknown = 64

No. of animals with records =

... and unknown sire = 0

... and unknown dam = 0

... and both parents unknown = 0

No. of sires = 313

... with progeny in the data = 179

... with records & progeny in data = 83

No. of dams = 1430

... with progeny in the data = 1026

... with records & progeny in data = 332

No. of animals with known/unpruned grand-parents

... with paternal grandsire = 3461

... with paternal granddam = 3323

... with maternal grandsire = 3332

... with maternal granddam = 3208

Inbreeding coefficients for random effect 1 computed

No. of inbred animals = 3075

Average inbreeding coefficient = 2.3699 (in %)

... amongst inbred animals = 2.7791 (in %)

random effect no. = 1 NRM

no. of elements in NRM/GIN inverse 13813

log determinant = -2480.3003433415274

======== end of file ============================10-02-2015==========15:19====

======= Version 30-08-2013 ======================================= **KM** ====

Program WOMBAT: Summary of information from Set-up step

==============================================================================

Analysis type : "muv 2"

Data file : "DADOS.dat"

Pedigree file : "ReducedPedFile.dat"

Parameter file : "wombat.par"

No. of traits = 2

nrec mean sdev min. max.

1 "LEA" 2283 51.4305 8.89390 21.4000 83.4000

2 "BF" 2285 1.75922 1.42798 0.00000 10.5000

Numbers of individuals/records for pairs of traits

1 2

1 "LEA" 2283 2283

2 "BF" 2283 2285

Covariables

1"LEA" nrec mean sdev min. max.

1 "idv(2)" 2283 6.11082 2.92852 2.00000 17.0000

2 "idade(1)" 2283 448.951 98.8881 306.000 609.000

2"BF" nrec mean sdev min. max.

1 "idv(2)" 2285 6.10810 2.92868 2.00000 17.0000

2 "idade(1)" 2285 449.081 98.9421 306.000 609.000

Fixed effects

1 "LEA" nlev

1 "gc" 65

2 "mn" 4

2 "BF" nlev

1 "gc" 65

2 "mn" 4

Random effects nlev

1 "animal" 3606 NRM

======== end of file ============================10-02-2015==========15:19====

======= Version 30-08-2013 ======================================= **KM** ====

Program WOMBAT: Estimates of covariance components

==============================================================================

Analysis type : "muv 2"

Data file : "DADOS.dat"

Pedigree file : "ReducedPedFile.dat"

Parameter file : "wombat.par"

No. of traits = 2 LEA BF

No. of records = 4568 2283 2285

No. of parameters = 6

Maximum log L = -6087.446

-1/2 AIC & AICC = -6093.446 -6093.455

-1/2 BIC = -6112.631 "Penalty factor" = 4.198

Parameter estimates with approx. sampling erors

1 CHOL Z 1 1 4.45129 0.174346

2 CHOL Z 1 2 0.218955 0.375786E-01

3 CHOL Z 2 2 0.767829 0.236024E-01

4 CHOL A 1 1 1.41227 0.678578E-01

5 CHOL A 1 2 0.484511E-01 0.575946E-01

6 CHOL A 2 2 -0.631821 0.828608E-01

Convergence criteria for last 3 iterates

Change in log likelihood = 21.672571 0.246674 0.000087

Change in parameter vector = 0.039581 0.006831 0.000135

Norm of gradient vector = 421.0346 31.4460 0.3942

Newton decrement = -41.2498 -0.4897 -0.0002

***** Estimates of residual covariances ************************************

Order of fit = 2

Covariance matrix

1 19.814

2 0.97463 0.63750

Eigenvalues of covariance matrix

Value 19.86 0.59

(%) 97.12 2.88

Trace 20.45

Matrix of correlations and variance ratios

1 0.5404

2 0.2742 0.6911

Covariances & correlations with approximate sampling errors

1 COVS Z 1 1 19.8140 1.55213 vrat 0.540 0.051

2 COVS Z 1 2 0.974630 0.174762 corr 0.274 0.045

3 COVS Z 2 2 0.637502 0.376129E-01 vrat 0.691 0.046

***** Estimates for RE 1 "animal" ***************************************

No. of levels = 3606

Covariance structure = NRM

Order of fit = 2

Covariance matrix

1 16.853

2 0.19890 0.28497

Eigenvalues of covariance matrix

Value 16.86 0.28

(%) 98.35 1.65

Trace 17.14

Matrix of correlations and variance ratios

1 0.4596

2 0.0908 0.3089

Covariances & correlations with approximate sampling errors

4 COVS A 1 1 16.8532 2.28724 vrat 0.460 0.051

5 COVS A 1 2 0.198905 0.239542 corr 0.091 0.106

6 COVS A 2 2 0.284970 0.479074E-01 vrat 0.309 0.046

***** Estimates of phenotypic covariances ***********************************

Covariance matrix

1 36.667

2 1.1735 0.92247

Eigenvalues of covariance matrix

Value 36.71 0.88

(%) 97.65 2.35

Trace 37.59

Correlation matrix

1 1.0000

2 0.2018 1.0000

Covariances & correlations with approximate sampling errors

7 COVS T 1 1 36.6672 1.36049

8 COVS T 1 2 1.17354 0.148578 corr 0.202 0.024

9 COVS T 2 2 0.922472 0.314141E-01

======== end of file ============================10-02-2015==========15:19====

======= Version 30-08-2013 ======================================= **KM** ====

Program WOMBAT: Summary of Pedigree Information

==============================================================================

Two traits analysis (LEA x RF)

Analysis type : "muv 2"

Data file : "DADOS.dat"

Pedigree file : "ReducedPedFile.dat"

Parameter file : "wombat.par"

No. of animal IDs in data file = = 2287

No. of animal IDs in total = = 3699

*****Pedigree Structure for random effect : 1 ****************************

Original no. of animals = 3699

No. of animals after pruning = 3608

... proportion (%) remaining = 97.5

No. of levels w/out records = 1321

No. of levels with records = 2287 100.0%

... 1 record(s) = 474 20.7%

... 2 record(s) = 1813 79.3%

No. of animals w/out offspring = 1865 51.7%

No. of animals with offspring = 1743 48.3%

... and records = 422 11.7%

No. of animals with unknown sire = 64

No. of animals with unknown dam = 161

No. of animals with both parents unknown = 64

No. of animals with records =

... and unknown sire = 0

... and unknown dam = 0

... and both parents unknown = 0

No. of sires = 313

... with progeny in the data = 179

... with records & progeny in data = 83

No. of dams = 1430

... with progeny in the data = 1026

... with records & progeny in data = 332

No. of animals with known/unpruned grand-parents

... with paternal grandsire = 3463

... with paternal granddam = 3325

... with maternal grandsire = 3334

... with maternal granddam = 3210

Inbreeding coefficients for random effect 1 computed

No. of inbred animals = 3077

Average inbreeding coefficient = 2.3711 (in %)

... amongst inbred animals = 2.7803 (in %)

random effect no. = 1 NRM

no. of elements in NRM/GIN inverse 13820

log determinant = -2481.7555421612105

======== end of file ============================10-02-2015==========15:21====

======= Version 30-08-2013 ======================================= **KM** ====

Program WOMBAT: Summary of information from Set-up step

==============================================================================

Analysis type : "muv 2"

Data file : "DADOS.dat"

Pedigree file : "ReducedPedFile.dat"

Parameter file : "wombat.par"

No. of traits = 2

nrec mean sdev min. max.

1 "LEA" 2283 51.4305 8.89390 21.4000 83.4000

2 "RF" 1817 5.08663 2.54341 0.00000 19.2000

Numbers of individuals/records for pairs of traits

1 2

1 "LEA" 2283 1813

2 "RF" 1813 1817

Covariables

1"LEA" nrec mean sdev min. max.

1 "idv(2)" 2283 6.11082 2.92852 2.00000 17.0000

2 "idade(1)" 2283 448.951 98.8881 306.000 609.000

2"RF" nrec mean sdev min. max.

1 "idv(2)" 1817 6.09191 3.03138 2.00000 17.0000

2 "idade(1)" 1817 469.100 100.703 306.000 609.000

Fixed effects

1 "LEA" nlev

1 "gc" 65

2 "mn" 4

2 "RF" nlev

1 "gc" 51

2 "mn" 3

Random effects nlev

1 "animal" 3608 NRM

======== end of file ============================10-02-2015==========15:21====

======= Version 30-08-2013 ======================================= **KM** ====

Program WOMBAT: Estimates of covariance components

==============================================================================

Analysis type : "muv 2"

Data file : "DADOS.dat"

Pedigree file : "ReducedPedFile.dat"

Parameter file : "wombat.par"

No. of traits = 2 LEA RF

No. of records = 4100 2283 1817

No. of parameters = 6

Maximum log L = -6752.450

-1/2 AIC & AICC = -6758.450 -6758.460

-1/2 BIC = -6777.311 "Penalty factor" = 4.144

Parameter estimates with approx. sampling erors

1 CHOL Z 1 1 4.44345 0.175322

2 CHOL Z 1 2 0.381084 0.683715E-01

3 CHOL Z 2 2 1.20399 0.456711E-01

4 CHOL A 1 1 4.11888 0.279482

5 CHOL A 1 2 0.185436 0.108306

6 CHOL A 2 2 0.990640 0.776990E-01

Convergence criteria for last 3 iterates

Change in log likelihood = 18.983481 0.300584 0.000335

Change in parameter vector = 0.034859 0.007160 0.000432

Norm of gradient vector = 216.8386 19.8606 0.4456

Newton decrement = -36.0323 -0.5908 -0.0006

***** Estimates of residual covariances ************************************

Order of fit = 2

Covariance matrix

1 19.744

2 1.6933 1.5948

Eigenvalues of covariance matrix

Value 19.90 1.44

(%) 93.26 6.74

Trace 21.34

Matrix of correlations and variance ratios

1 0.5379

2 0.3018 0.6109

Covariances & correlations with approximate sampling errors

1 COVS Z 1 1 19.7443 1.55807 vrat 0.538 0.051

2 COVS Z 1 2 1.69333 0.319282 corr 0.302 0.051

3 COVS Z 2 2 1.59481 0.116919 vrat 0.611 0.053

***** Estimates for RE 1 "animal" ***************************************

No. of levels = 3608

Covariance structure = NRM

Order of fit = 2

Covariance matrix

1 16.965

2 0.76379 1.0158

Eigenvalues of covariance matrix

Value 17.00 0.98

(%) 94.55 5.45

Trace 17.98

Matrix of correlations and variance ratios

1 0.4621

2 0.1840 0.3891

Covariances & correlations with approximate sampling errors

4 COVS A 1 1 16.9651 2.30231 vrat 0.462 0.051

5 COVS A 1 2 0.763787 0.458854 corr 0.184 0.104

6 COVS A 2 2 1.01575 0.161962 vrat 0.389 0.053

***** Estimates of phenotypic covariances ***********************************

Covariance matrix

1 36.709

2 2.4571 2.6106

Eigenvalues of covariance matrix

Value 36.89 2.43

(%) 93.81 6.19

Trace 39.32

Correlation matrix

1 1.0000

2 0.2510 1.0000

Covariances & correlations with approximate sampling errors

7 COVS T 1 1 36.7094 1.36590

8 COVS T 1 2 2.45712 0.287335 corr 0.251 0.027

9 COVS T 2 2 2.61056 0.103478

======== end of file ============================10-02-2015==========15:21====

======= Version 30-08-2013 ======================================= **KM** ====

Program WOMBAT: Summary of Pedigree Information

==============================================================================

Two traits analysis (BF x RF)

Analysis type : "muv 2"

Data file : "DADOS.dat"

Pedigree file : "ReducedPedFile.dat"

Parameter file : "wombat.par"

No. of animal IDs in data file = = 2287

No. of animal IDs in total = = 3699

*****Pedigree Structure for random effect : 1 ****************************

Original no. of animals = 3699

No. of animals after pruning = 3608

... proportion (%) remaining = 97.5

No. of levels w/out records = 1321

No. of levels with records = 2287 100.0%

... 1 record(s) = 472 20.6%

... 2 record(s) = 1815 79.4%

No. of animals w/out offspring = 1865 51.7%

No. of animals with offspring = 1743 48.3%

... and records = 422 11.7%

No. of animals with unknown sire = 64

No. of animals with unknown dam = 161

No. of animals with both parents unknown = 64

No. of animals with records =

... and unknown sire = 0

... and unknown dam = 0

... and both parents unknown = 0

No. of sires = 313

... with progeny in the data = 179

... with records & progeny in data = 83

No. of dams = 1430

... with progeny in the data = 1026

... with records & progeny in data = 332

No. of animals with known/unpruned grand-parents

... with paternal grandsire = 3463

... with paternal granddam = 3325

... with maternal grandsire = 3334

... with maternal granddam = 3210

random effect no. = 1 NRM

no. of elements in NRM/GIN inverse 13820

log determinant = -2481.7555421612105

======== end of file ============================10-02-2015==========15:25====

======= Version 30-08-2013 ======================================= **KM** ====

Program WOMBAT: Summary of information from Set-up step

==============================================================================

Analysis type : "muv 2"

Data file : "DADOS.dat"

Pedigree file : "ReducedPedFile.dat"

Parameter file : "wombat.par"

No. of traits = 2

nrec mean sdev min. max.

1 "BF" 2285 1.75922 1.42798 0.00000 10.5000

2 "RF" 1817 5.08663 2.54341 0.00000 19.2000

Numbers of individuals/records for pairs of traits

1 2

1 "BF" 2285 1815

2 "RF" 1815 1817

Covariables

1"BF" nrec mean sdev min. max.

1 "idv(2)" 2285 6.10810 2.92868 2.00000 17.0000

2 "idade(1)" 2285 449.081 98.9421 306.000 609.000

2"RF" nrec mean sdev min. max.

1 "idv(2)" 1817 6.09191 3.03138 2.00000 17.0000

2 "idade(1)" 1817 469.100 100.703 306.000 609.000

Fixed effects

1 "BF" nlev

1 "gc" 65

2 "mn" 4

2 "RF" nlev

1 "gc" 51

2 "mn" 3

Random effects nlev

1 "animal" 3608 NRM

======== end of file ============================10-02-2015==========15:25====

======= Version 30-08-2013 ======================================= **KM** ====

Program WOMBAT: Estimates of covariance components

==============================================================================

Analysis type : "muv 2"

Data file : "DADOS.dat"

Pedigree file : "ReducedPedFile.dat"

Parameter file : "wombat.par"

No. of traits = 2 BF RF

No. of records = 4102 2285 1817

No. of parameters = 6

Maximum log L = -2423.147

-1/2 AIC & AICC = -2429.147 -2429.157

-1/2 BIC = -2448.010 "Penalty factor" = 4.144

Parameter estimates with approx. sampling erors

1 CHOL Z 1 1 1.26824 0.446236E-01

2 CHOL Z 1 2 0.411809 0.335607E-01

3 CHOL Z 2 2 0.683214 0.179966E-01

4 CHOL A 1 1 -0.156826E-01 0.793077E-01

5 CHOL A 1 2 0.426729 0.520763E-01

6 CHOL A 2 2 -1.12926 0.131805

Convergence criteria for last 3 iterates

Change in log likelihood = 4.580935 0.007359 0.000004

Change in parameter vector = 0.057343 0.003728 0.000181

Norm of gradient vector = 109.5298 3.7662 0.0314

Newton decrement = -8.9242 -0.0147 -0.0000

***** Estimates of residual covariances ************************************

Order of fit = 2

Covariance matrix

1 0.63637

2 0.52227 1.6084

Eigenvalues of covariance matrix

Value 1.84 0.41

(%) 81.78 18.22

Trace 2.24

Matrix of correlations and variance ratios

1 0.6895

2 0.5162 0.6240

Covariances & correlations with approximate sampling errors

1 COVS Z 1 1 0.636368 0.374617E-01 vrat 0.689 0.046

2 COVS Z 1 2 0.522271 0.540673E-01 corr 0.516 0.032

3 COVS Z 2 2 1.60842 0.113187 vrat 0.624 0.051

***** Estimates for RE 1 "animal" ***************************************

No. of levels = 3608

Covariance structure = NRM

Order of fit = 2

Covariance matrix

1 0.28660

2 0.42009 0.96912

Eigenvalues of covariance matrix

Value 1.17 0.09

(%) 93.10 6.90

Trace 1.26

Matrix of correlations and variance ratios

1 0.3105

2 0.7971 0.3760

Covariances & correlations with approximate sampling errors

4 COVS A 1 1 0.286602 0.477274E-01 vrat 0.311 0.046

5 COVS A 1 2 0.420089 0.730839E-01 corr 0.797 0.057

6 COVS A 2 2 0.969122 0.153718 vrat 0.376 0.051

***** Estimates of phenotypic covariances ***********************************

Covariance matrix

1 0.92297

2 0.94236 2.5775

Eigenvalues of covariance matrix

Value 3.00 0.50

(%) 85.82 14.18

Trace 3.50

Correlation matrix

1 1.0000

2 0.6110 1.0000

Covariances & correlations with approximate sampling errors

7 COVS T 1 1 0.922969 0.314076E-01

8 COVS T 1 2 0.942360 0.471141E-01 corr 0.611 0.016

9 COVS T 2 2 2.57755 0.993613E-01

======== end of file ============================10-02-2015==========15:25====
